# Cobalt-catalysed site-selective intra- and intermolecular dehydrogenative amination of unactivated sp^3^ carbons

**DOI:** 10.1038/ncomms7462

**Published:** 2015-03-10

**Authors:** Xuesong Wu, Ke Yang, Yan Zhao, Hao Sun, Guigen Li, Haibo Ge

**Affiliations:** 1 Department of Chemistry and Chemical Biology, Indiana University-Purdue University Indianapolis, 402 N. Blackford Street, Indianapolis, Indiana 46202, USA; 2 Institute of Chemistry and BioMedical Sciences, Nanjing University, Nanjing 210093, P.R. China; 3 Department of Chemistry and Biochemistry, Texas Tech University, Lubbock, Texas 79409-1061, USA

## Abstract

Cobalt-catalysed sp^2^ C–H bond functionalization has attracted considerable attention in recent years because of the low cost of cobalt complexes and interesting modes of action in the process. In comparison, much less efforts have been devoted to the sp^3^ carbons. Here we report the cobalt-catalysed site-selective dehydrogenative cyclization of aliphatic amides via a C–H bond functionalization process on unactivated sp^3^ carbons with the assistance of a bidentate directing group. This method provides a straightforward synthesis of monocyclic and spiro β- or γ-lactams with good to excellent stereoselectivity and functional group tolerance. In addition, a new procedure has been developed to selectively remove the directing group, which enables the synthesis of free β- or γ-lactam compounds. Furthermore, the first cobalt-catalysed intermolecular dehydrogenative amination of unactivated sp^3^ carbons is also realized.

## Abstract (web summary)

Functionalizing unactivated carbon–hydrogen bonds is challenging, especially when using non-precious metals and dealing with sp^3^ hybridized carbons. Here, the authors report an intramolecular cobalt catalysed amination of C–H bonds of sp^3^ carbons, giving access to β- and γ-lactams.

## Graphical abstract

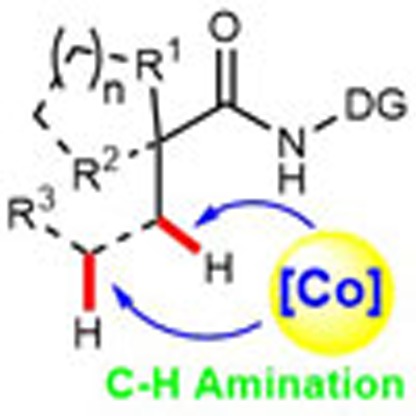

Transition metal-catalysed direct functionalization of relatively unreactive C–H bonds has emerged as a major topic of research in organic chemistry^1^,^2^,^3^,^4^,^5^,^6^,^7^,^8^,^9^,^10^,^11^,^12^,^13^. This method does not require the use of prefunctionalized materials, and thus provides an attractive alternative to traditional cross-coupling reactions. Within this reaction class, cobalt-catalysed processes have received special interest due to the low cost and toxicity of cobalt complexes, and their interesting modes of action^14^,^15^,^16^,^17^. In the 1950s, Murahashi *et al*. demonstrated the chelation-assisted C–H functionalization process on benzaldimines and azobenzenes, and it is well accepted that the catalytic cycle is initiated by oxidative addition of a low-valent cobalt species to the aromatic C–H bonds^18^,^19^,^20^,^21^. Recently, azoles, benzamides and 2-phenylpyridines were also proven to be effective substrates through a similar reaction pathway^22^,^23^,^24^,^25^,^26^. Furthermore, Co^II^ or Co^III^-catalysed direct C–H functionalization of azole, 2-phenylpyridine, indole and benzamide derivatives was also demonstrated^27^,^28^,^29^,^30^. In this case, the C–H bond activation process is believed to proceed through either an electrophilic aromatic substitution or concerted metalation-deprotonation pathway. Moreover, the cobalt-catalysed hydroacylation of olefins has also been demonstrated via an sp^2^ C–H functionalization process in the absence of chelation assistance^14^,^31^.

In comparison with the well-established cobalt-catalysed direct functionalization on sp^2^ carbons, there are only a few examples of direct functionalization on sp^3^ C–H bonds (Fig. 1). Cenini and co-workers reported the intermolecular amination of relatively reactive sp^3^ carbons with moderate yields in 1999 (refs 32, 33). Recently, more efficient intramolecular version of this transformation was developed on electron-deficient sp^3^ carbons in Zhang’s laboratory (Fig. 1a)^34^,^35^. These reactions were proposed to proceed via the outer-sphere mechanism, in which a carbon–metal bond is not involved^5^,^36^,^37^. Instead, the sp^3^ C–H bonds were indirectly activated by an inter- or intramolecular hydrogen atom transfer of the radical intermediates. In contrast to this, Brookhart and co-workers reported intramolecular hydrogen transfer of cyclic amines in 2007 via an inner-sphere mechanism, in which a carbon–cobalt bond was formed by oxidative addition of a cobalt species to the α-sp^3^ C–H bond (Fig. 1b)^36^,^37^,^38^,^39^,^40^.

Inspired by the reports of transition metal-catalysed bidentate ligand-directed sp^3^ C–H functionalization process^41^,^42^, we have explored and demonstrated here the cobalt-catalysed site-selective direct C–H functionalization on unactivated sp^3^ carbons with the aid of a bidentate directing group (Fig. 1c). In addition, a novel two-step procedure has been developed under oxidative conditions to remove the directing group, which enables an efficient access to β-lactam, γ-lactam or β-amino amide derivatives.

## Results

### Reaction condition optimization of intramolecular amidation

Synthesis of lactams via transition metal-catalysed C–H functionalization is of current research interest because of the biological importance of these molecules^43^,^44^. In the past two years, Pd-, Cu- or Ni-catalysed process for the formation of β- or γ-lactams has been achieved^45^,^46^,^47^,^48^,^49^,^50^,^51^. However, all of these approaches suffer from their own limitations on the substrate scope. To provide a complementary method and demonstrate the feasibility of cobalt catalysis on unactivated sp^3^ carbons, we carried out the study of cobalt-catalysed bidentate ligand-directed intramolecular cyclization of propanamides. Our investigation began with oxidative cyclization of 2-ethyl-2-methyl-*N*-(quinolin-8-yl)pentanamide (**1a**) in the presence of catalytic amount of CoCl_2_ by using Ag_2_CO_3_ as the oxidant (Table 1). After an initial solvent screening, chlorobenzene turned out to be optimal, producing the β-lactam compound **2a** in 33% yield (entry 1). Notably, this reaction proceeded in a highly site-selective manner, favouring the C–H bond of a β-methyl group over those of β-methylene and γ-methyl groups. Next, a screening on oxidants was carried out. It was observed that the reaction could also be performed with several other oxidants with lower efficiency (entries 2–4). Further optimization of reaction conditions showed that the reaction could be significantly improved by using Co(OAc)_2_ as the catalyst and sodium benzoate as the base (entry 15). Considering that PhCO_2_Na could potentially compete with amide **1** for coordination to the cobalt complex, we further reduced the loading of this base. Delightfully, the chemical yield of this reaction was significantly improved (entry 17).

### Substrate scope of intramolecular amination

**Figure 2:**
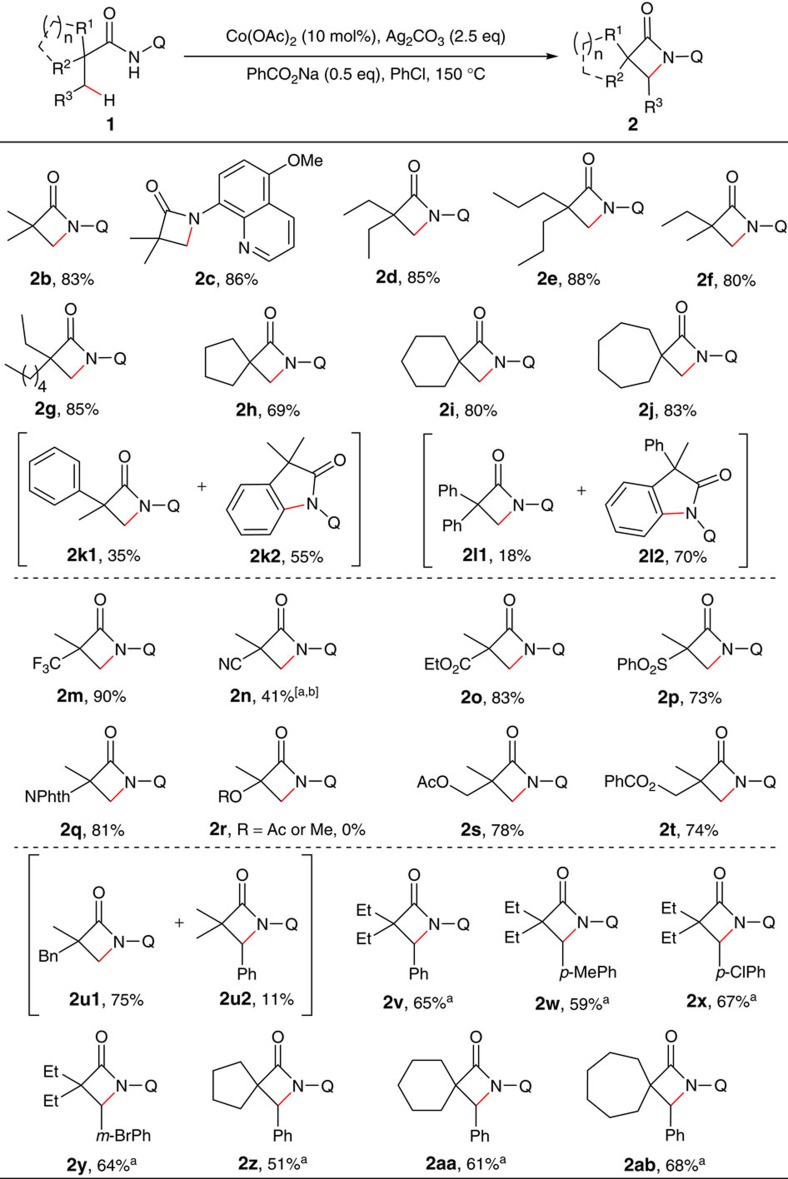
Scope of α,α-disubstituted propanamides. Reaction conditions: **1** (0.3 mmol), Co(OAc)_2_ (0.03 mmol), Ag_2_CO_3_ (0.75 mmol), PhCO_2_Na (0.15 mmol), 0.6 ml PhCl, 150 °C, 24 h. Isolated yield based on three runs of each reaction. ^a^Run for 48 h. ^b^Co(OAc)_2_ (0.06 mmol). Q=8-quinolinyl.

With optimized conditions in hand, the substrate scope studies were carried out (Fig. 2; also see Supplementary Figs 1 and 2 for the structures of substrates). Gratifyingly, the reaction showed great generality with 2,2-disubstituted propanamides bearing either linear or cyclic chains on α-carbons with predominant selectivity for C–H bonds of β-methyl groups (**2b**, **2d**–**j**). However, with α-phenyl substituted substrates, a preference of C–H bond functionalization of sp^2^ carbons was observed, providing indolin-2-one derivative as the major products (**2k** and **2l**). Noticeably, the replacement of the quinolyl group with 5-methoxyquinolyl group had no apparent effect on the reaction (**2c**). Moreover, the removability of 5-methoxyquinolyl moiety of β- or γ-lactams has been well documented^45^,^48^,^49^,^50^,^51^.

Furthermore, substrates bearing a trifluoromethyl, cyano, ethoxycarbonyl, sulfonyl or phthalimidyl group on an α-carbon showed good compatibility (**2m**–**q**). In addition, although α-methoxy and acetoxy-substituted amides failed to provide the desired products (**2r**), substrates with an acetoxy or benzenecarboxy group on β-carbons produced β-lactams **2s** and **2t** in good yields. It was also noticed that the reaction favours the C–H bond of the β-methyl over that of the more reactive benzyl group (**2u**). Moreover, the β-benzylic sp^3^ C–H bonds could also be effectively functionalized (**2v**-**ab**).

**Figure 3:**
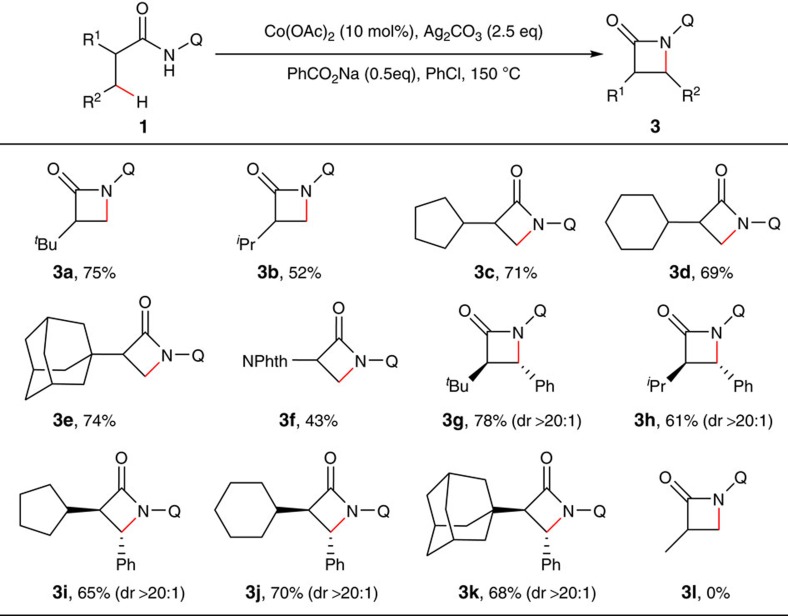
Scope of α-monosubstituted propanamides. Reaction conditions: same as in Fig. 2. Isolated yield based on three runs of each reaction.

Next, we carried out compatibility studies of α-monosubstituted propanamide derivatives (Fig. 3; also see Supplementary Figs 1 and 2 for the structures of substrates). To our delight, introduction of a relatively bulky group on the α-carbon could effectively initiate the process (**3a–k**). Furthermore, excellent diastereoselectivity was observed with β-phenyl substituted substrates (**3g**–**k**).

**Figure 4:**
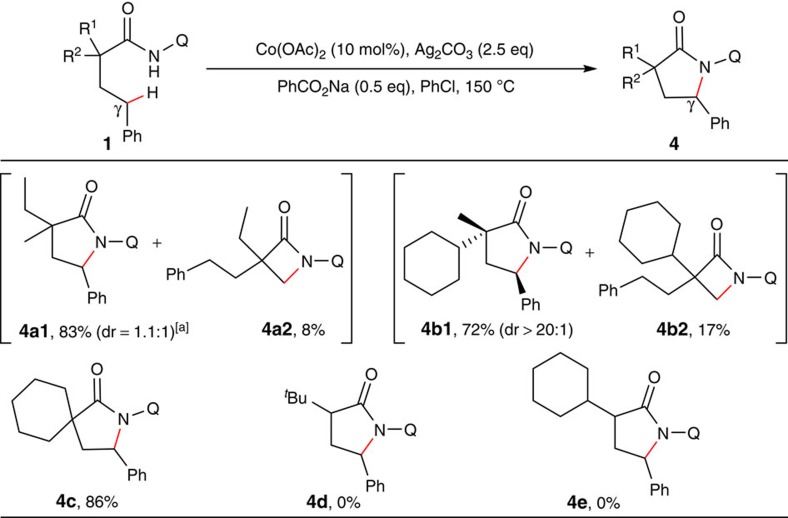
Functionalization of sp^3^ carbons. Reaction conditions: same as in Fig. 2. Isolated yield based on three runs of each reaction. ^a^Unisolated diastereoisomers.

Interestingly, a great preference of functionalizing the C–H bonds of γ-benzylic carbons over those of β-methyl carbons was observed during the course of substrate scope studies, providing γ-lactams as the major products (Fig. 4; also see Supplementary Figs 1 and 2 for the structures of substrates). However, α-monosubstituted substrates failed to provide any γ-lactams (**4d–e**).

### Mechanistic investigation

**Figure 5:**
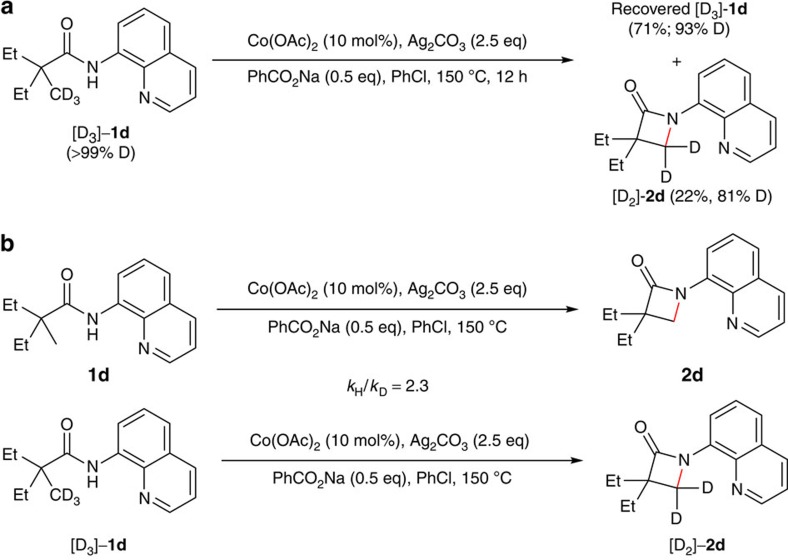
Deuterium labelling experiments. (**a**) The deuterium–proton exchange experiment. (**b**) The kinetic isotope effect experiments.

To gain some insights on the mechanism of this reaction, the deuterium-labelling experiments were carried out (Fig. 5). With the deuterium-labelled compound [D_3_]-**1d**, an apparent deuterium-proton exchange occurred with both the substrate and product (Fig. 5a). More interestingly, the product has a lower deuterium ratio compared with the recovered starting material, which is presumably due to the different reaction rates of proton- and deuterium-containing starting materials. Furthermore, a primary kinetic isotope effect was also observed for **1d** based on the early relative rate of parallel reactions (see Supplementary Methods), indicating that the sp^3^ C–H bond cleavage of amide **1d** is the rate-limiting step in the catalytic process (Fig. 5b).

We then carried out a series of control experiments with 2-ethyl-2-methyl-*N*-(quinolin-8-yl)butanamide (**1d**). As shown in Table 2, the reaction failed to provide the desired product without a cobalt catalyst under the standard or modified conditions based on Shi’s study (entries 2 and 3)^52^. It was then noticed that the oxidant, Ag_2_CO_3_, could be replaced with Ce(SO_4_)_2_, albeit with a low yield (entries 4 and 5). Furthermore, no desired product **2d** was obtained with stoichiometric amounts of commercially available Co(acac)_3_ or CoF_3_ in the absence of Ag_2_CO_3_ (entries 6 and 7). On the other hand, the reaction could be performed with a catalytic amount of Co(acac)_3_ or CoF_3_ in the presence of Ag_2_CO_3_ (entries 8 and 9). These results suggest that the C–H bond activation process could be initiated by a Co^III^ species^28^,^29^,^30^, but the product is unlikely generated from reductive elimination of a Co^III^ complex. It was also observed that addition of the radical inhibitor, TEMPO, had no significant effect on the reaction, indicating that a radical intermediate may not be involved in the catalytic cycle (entries 10 and 11).

Next, a series of control experiments with 1-phenethyl-*N*-(quinolin-8-yl)cyclohexane-1-carboxamide (**1d**) were carried out to explore the plausible reaction pathway for the formation of γ-lactams (Supplementary Table 1). It was found that neither a cobalt nor a silver species is required for this reaction (entries 2–5). However, the efficiency of the reaction was significantly decreased without these species. Furthermore, reaction yield was dramatically decreased by the addition of TEMPO, which indicates that an alkyl radical intermediate generated from a single electron oxidation process may be involved in the reaction (entries 6 and 7).

**Figure 6:**
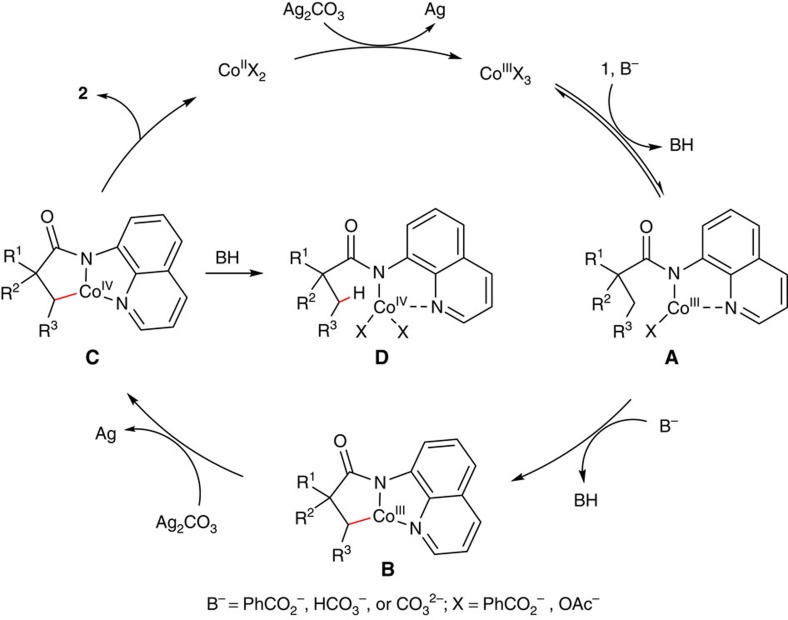
Proposed catalytic cycle of β-lactam formation. The possible mechanism involves the Co-catalysed sp^3^ C–H activation, oxidation and subsequent reductive elimination.

On the basis of the above results, a plausible catalytic cycle for the formation of β-lactams is proposed (Fig. 6)^14^,^15^,^16^,^17^,^53^,^54^. The Co^III^ complex **A** is initially generated by coordination of amide **1** to a cobalt species followed by a ligand exchange process under basic conditions. Cyclometalation of this intermediate produces the intermediate **B**, which is believed to be an irreversible step based on the kinetic isotope effect studies. In this process, benzoate might act as a ligand to coordinate to the Co^III^ complex, and subsequently facilitates the C–H bond cleavage via concerted metallation-deprotonation^7^,^55^,^56^. Oxidation of intermediate **B** with Ag_2_CO_3_ gives rise to the Co^IV^ complex **C**, which produces the β-lactam compound **2** upon reductive elimination^57^,^58^. The newly generated Co^II^ species could then be re-oxidized to the Co^III^ species to furnish the catalytic cycle. It is noteworthy that the catalytic Co^II^/Co^IV^ cycle could not be excluded, which involves cyclometalation of amide **1** with a Co^II^ species followed by oxidation to generate the intermediate **C**. It should also be mentioned that a competing side reaction, protonation of the Co^IV^ complex **C**, is also possible in the process, giving the Co^IV^ species **D**. Furthermore, although a radical-mediated process could not be excluded, the observed high selectivity of the β-methyl over the β-benzylic C–H bonds suggests that the catalytic cycle is unlikely performed with a radical intermediate. However, in the case of the formation of γ-lactam derivatives, a radical or cationic species is believed to be involved in the catalytic cycle because of the predominant preference of functionalization of the γ-benzylic over the β-methyl C–H bonds.

**Figure 7:**
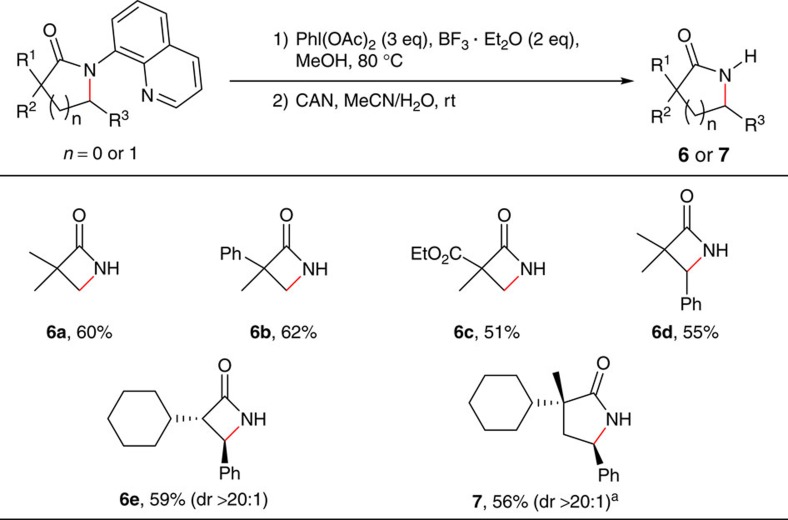
Removal of the quinolyl group. Reaction conditions: (1) amide (0.15 mmol), BF_3_·Et_2_O (0.30 mmol), PhI(OAc)_2_ (0.45 mmol), MeOH (0.5 ml), 80 °C, 3–8 h; (2) CAN (0.45 mmol), MeCN/H_2_O(2.0 ml/0.4 ml), room temperature, 6 h. Isolated yield based on three runs of each reaction. ^a^The methoxylation step was carried out at 50 °C.

To broaden the synthetic applications of this method, we carried out studies on the selective removal of the directing group (Fig. 7). It was found that the quinolyl group could be cleaved by the introduction of a methoxy group on the C5 position of this moiety under oxidative conditions followed by oxidative cleavage of the newly generated 5-methoxyquinolyl moiety with ammonium cerium(IV) nitrate (CAN). Under these conditions, α-mono and α,α-di-substituted β-lactams, and α,α-di-substituted γ-lactams were all effective substrates, which enables the efficient synthesis of free β- or γ-lactam compounds.

### Intermolecular amination

**Figure 8:**
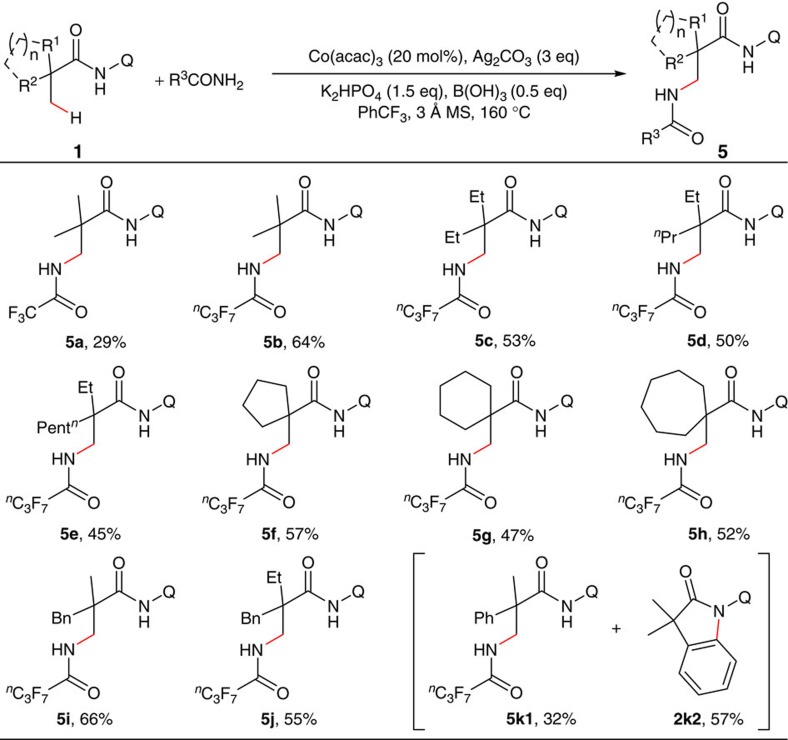
Intermolecular amination of α,α-disubstituted propanamides. Reaction conditions: **1** (0.15 mmol), amide (0.45 mmol), Co(acac)_3_ (0.03 mmol), Ag_2_CO_3_ (0.45 mmol), K_2_HPO_4_ (0.23 mmol), B(OH)_3_ (0.075 mmol), 2.0 ml PhCF_3_, 160 °C, 24 h. Isolated yield based on three runs of each reaction.

Direct intermolecular amination of sp^3^ carbons is an important research topic because of the importance of the products in pharmaceutical industry^5^,^59^,^60^,^61^. As one of the most efficient synthetic methods, the transition metal-catalysed ligand-directed approach has attracted considerable attention and significant progress has been achieved in recent years^62^,^63^,^64^,^65^,^66^,^67^. However, within this reaction category, reports of dehydrogenative aminations are rare^62^,^63^ Encouraged by the above results, we carried out the study of a cobalt-catalysed intermolecular dehydrogenative amination of *N*-(quinolin-8-yl)propanamide derivatives (Fig. 8; also see Supplementary Figs 1 and 2 for the structures of substrates). After an extensive screening, trifluoroacetamide was proved to be an effective coupling partner (**5a**), whereas many other nitrogen sources such as acetamide, benzamide, phathalimide, sulfonamide, morpholine and aniline failed to produce the desired products (for optimization of reaction conditions, see Supplementary Table 2). Furthermore, replacement of trifluoroacetamide with heptafluorobutanamide significantly improved the reaction (**5b**). As expected, 2,2-disubstituted propanamides bearing either linear or cyclic chains on α-carbons proceeded smoothly to give the corresponding products **5c**–**j** with a predominant selectivity for C–H bonds of β-methyl groups. It was also noticed that with α-phenyl-substituted substrate **1k**, C–H bond functionalization of sp^2^ carbons was favoured, providing indolin-2-one derivative as the major product (**5k1** and **2k2**).

We then carried out a series of control experiments with 2-ethyl-2-methyl-*N*-(quinolin-8-yl)butanamide (**1d**) to gain some insights on the reaction mechanism. As shown in Supplementary Table 3, the reaction failed to provide the desired product in the absence of a cobalt or silver species (entries 2–4). Furthermore, the addition of TEMPO had no apparent effect on the reaction, indicating that an alkyl radical intermediate may not be involved in this process (entries 5 and 6).

## Discussion

As described, a highly regioselective intramolecular amination of propionamide and butyramide derivatives with an 8-aminoquinolinyl group as the bidentate directing group was developed via a cobalt-catalysed sp^3^ C–H bond functionalization process. The reaction favours the C–H bonds of β-methyl groups over those of the β-methylene and γ- or δ-methyl groups, providing the β-lactam derivatives in a highly site- and diastereo-selective manner. Interestingly, a predominant preference for the functionalization of γ-benzylic over β-methyl C–H bonds was observed, producing γ-lactams as the major products. On the basis of these results, it is believed that two distinct reaction pathways are involved in the formation of these four- and five-membered ring products. As mentioned earlier, synthesis of lactams has been demonstrated recently via a Pd-, Cu- or Ni-catalysed C–H functionalization process. However, the Cu- or Ni-catalysed process is restricted to substrates with an α-quaternary carbon and the formation of β-lactams. On the other hand, Pd-catalysed synthesis of β-lactams either is restricted to α-unsubstituted or α,β-cyclic substrates, or suffers from the irremovability of the directing group, whereas synthesis of γ-lactams is limited to β-substituted substrates. Therefore, this method provides a complementary approach to access monocyclic and spiro β- or γ-lactams. Furthermore, the cobalt-catalysed ligand-directed intermolecular amination of unactivated sp^3^ carbons was realized for the first time. The detailed mechanistic investigations of these transformations are currently undergoing in our laboratory. In the meanwhile, *N*-phosphonyl and phosphinyl groups will be investigated for the present intra- and intermolecular dehydrogenative amination reactions for achieving the GAP work-up^68^,^69^.

## Methods

### General methods

For ^1^H and ^13^C NMR spectra of compounds in this manuscript and details of the synthetic procedures, see Supplementary Figs 3–162 and Supplementary Methods.

### General procedure for intramolecular amination

A 20-ml tube was charged with α,α,α-trisubstituted *N*-(quinolin-8-yl)acetamides (**1**, 0.30 mmol), Co(OAc)_2_ (5.3 mg, 0.030 mmol), Ag_2_CO_3_ (207 mg, 0.75 mmol), PhCO_2_Na (21.6 mg, 0.15 mmol) and 0.60 ml of PhCl. The reaction mixture was stirred rigorously open to the air at 150 °C for 24 h. Then, the mixture was cooled to room temperature, diluted with EtOAc (2 ml), filtered through a celite pad and concentrated *in vacuo*. The residue was purified by flash chromatography on silica gel (gradient eluent of 2–5% EtOAc in hexanes, v/v) to give the desired product.

### General procedure for intermolecular amination

A 20-ml tube was charged with α,α,α-trisubstituted *N*-(quinolin-8-yl)acetamides (**1**, 0.15 mmol), heptafluorobutyramide (95.9 mg, 0.45 mmol), Co(acac)_3_ (10.7 mg, 0.030 mmol), Ag_2_CO_3_ (75.1 mg, 0.45 mmol), K_2_HPO_4_ (39.2 mg, 0.23 mmol), B(OH)_3_ (4.6 mg, 0.075 mmol), 3 Å MS (200 mg) and 2.0 ml of PhCF_3_. Then the vial was sealed, and stirred rigorously at 160 °C for 24 h. The mixture was cooled to room temperature, diluted with CH_2_Cl_2_ (5 ml), filtered through a celite pad and concentrated *in vacuo*. The residue was purified by flash chromatography on silica gel (gradient eluent of 2–5% EtOAc in hexanes, v/v) to give the desired product.

## Author contributions

X.W. and K.Y. performed the experiments and analysed the data. All authors conceived and designed the experiments, contributed to discussions and wrote the manuscript.

## Additional information

**How to cite this article:** Wu, X. *et al*. Cobalt-catalysed site-selective intra- and intermolecular dehydrogenative amination of unactivated sp^3^ carbons. *Nat. Commun.* 6:6462 doi: 10.1038/ncomms7462 (2015).

## Supplementary Material

Supplementary Information Supplementary Figures 1-162, Supplementary Table 1-3, Supplementary Methods and Supplementary References

## Figures and Tables

**Figure 1:**
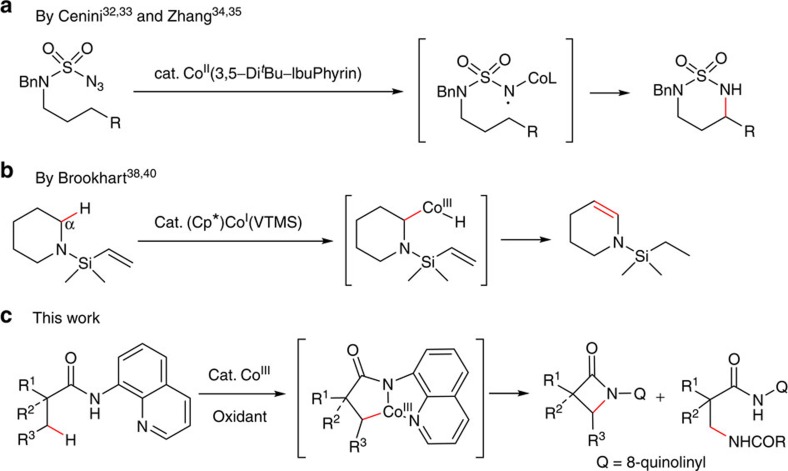
Cobalt-catalysed sp^3^ C–H bond functionalization. (**a**) Out-sphere mechanism. (**b**) Inner-sphere mechanism (via oxidative addition to a C–H bond). (**c**) Inner-sphere mechanism (via cyclometalation of an sp^3^ carbon).

**Table 1:** Optimization of reaction conditions.

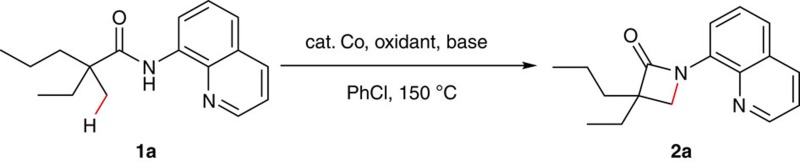				
**Entry**	**Co source (mol%)**	**Oxidant (2.5** **eq)**	**Base (eq)**	**Yield (%)**
1	CoCl_2_ (10)	Ag_2_CO_3_	K_2_HPO_4_ (1)	33
2	CoCl_2_ (10)	Ce(SO_4_)_2_	K_2_HPO_4_ (1)	7
3	CoCl_2_ (10)	AgOAc	K_2_HPO_4_ (1)	24
4	CoCl_2_ (10)	Ag_2_O	K_2_HPO_4_ (1)	<5
5	CoBr_2_ (10)	Ag_2_CO_3_	K_2_HPO_4_ (1)	12
6	CoF_2_ (10)	Ag_2_CO_3_	K_2_HPO_4_ (1)	22
7	Co(acac)_2_ (10)	Ag_2_CO_3_	K_2_HPO_4_ (1)	28
8	Co(PhCO_2_)_2_ (10)	Ag_2_CO_3_	K_2_HPO_4_ (1)	38
9	Co(OAc)_2_ (10)	Ag_2_CO_3_	K_2_HPO_4_ (1)	45
10	CoF_3_ (10)	Ag_2_CO_3_	K_2_HPO_4_ (1)	27
11	Co(acac)_3_ (10)	Ag_2_CO_3_	K_2_HPO_4_ (1)	30
12	Co(OAc)_2_ (10)	Ag_2_CO_3_	Na_2_HPO_4_ (1)	56
13	Co(OAc)_2_ (10)	Ag_2_CO_3_	Na_2_CO_3_ (1)	19
14	Co(OAc)_2_ (10)	Ag_2_CO_3_	NaOAc (1)	42
15	Co(OAc)_2_ (10)	Ag_2_CO_3_	PhCO_2_Na (1)	75
16	Co(OAc)_2_ (10)	Ag_2_CO_3_	—	33
17	Co(OAc)_2_ (10)	Ag_2_CO_3_	PhCO_2_Na (0.5)	90 (87)

Reaction conditions: **1a** (0.3 mmol), Co source (10 mol%), oxidant (2.5 eq), base, 0.6 ml of solvent, 150 °C, 24 h. Yields are based on **1a**, determined by ^1^H NMR using dibromomethane as the internal standard. Isolated yield is in brackets based on three runs.

**Table 2:** Control experiments of β-lactam formation.

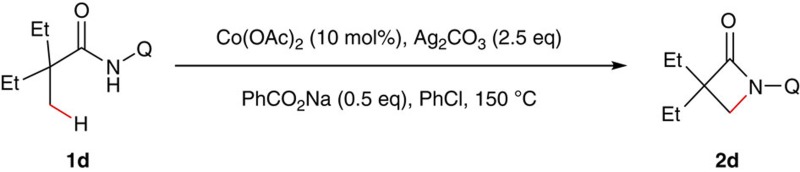		
**Entry**	Change from the ‘standard conditions’*	**Yield of 2d (%)**†
1	None	88
2	No Co(OAc)_2_	0
3	Shi’s conditions‡ instead of the standard conditions	0
4	Co(OAc)_2_ (1 eq), no Ag_2_CO_3_	0
5	Ce(SO_4_)_2_ (6 eq) instead of Ag_2_CO_3_	25
6	Co(acac)_3_ (1 eq) instead of Co(OAc)_2_, no Ag_2_CO_3_	0
7	CoF_3_ (1 eq) instead of Co(OAc)_2_, no Ag_2_CO_3_	0
8	Co(acac)_3_ (10%) instead of Co(OAc)_2_	53
9	CoF_3_ (10%) instead of Co(OAc)_2_	46
10	Addition of TEMPO (1 eq)	63
11	Addition of TEMPO (2 eq)	56

^*^Reaction conditions: **1d** (0.3 mmol), Co(OAc)_2_ (0.03 mmol), Ag_2_CO_3_ (0.75 mmol), PhCO_2_Na (0.15 mmol), 0.6 ml PhCl, 150 °C, 24 h.

^†^Yields are based on **1d**, determined by ^1^H NMR using dibromomethane as the internal standard.

^‡^Shi’s conditions: **1d** (0.25 mmol), AgOAc (20 mol%), PhI(TFA)_2_ (0.50 mmol), 4,4′-di-t-butyl-2,2′-bipyridine (20 mol%), K_2_CO_3_ (0.50 mmol), PhCl/DCE (1.5 ml/1.5 ml), 120 °C, 12 h.
