# Supplementary material for: Cobalt-catalysed site-selective intra- and intermolecular dehydrogenative amination of unactivated sp3 carbons
Source: Nat Commun. 2015 Mar 10;6:6462. doi: 10.1038/ncomms7462 (PMC4366490; doi:10.1038/ncomms7462)
Supplement: Supplementary Information — Supplementary Figures 1-162, Supplementary Table 1-3, Supplementary Methods and Supplementary References [file ncomms7462-s1.pdf]

Supplementary Figures

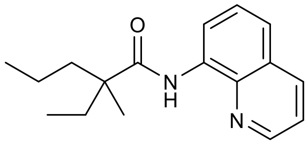

1a

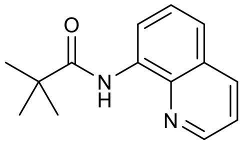

1b

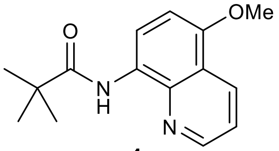

1c

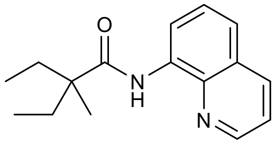

1d

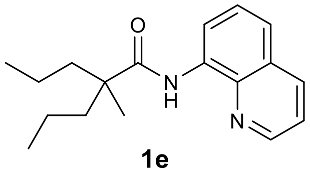

1e

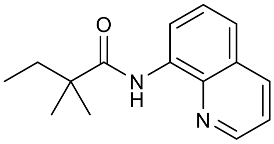

1f

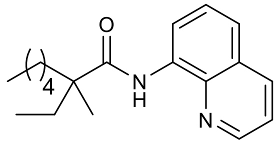

1g

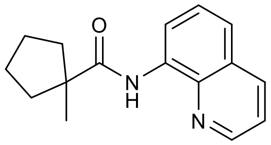

1h

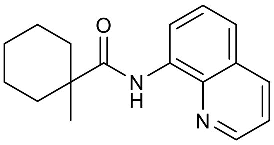

1i

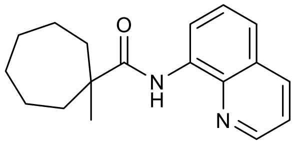

1j

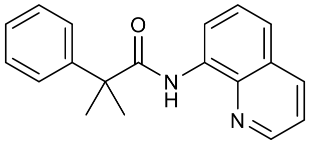

1k

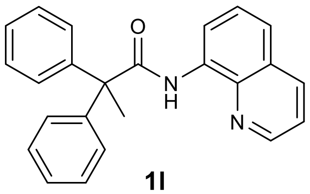

1l

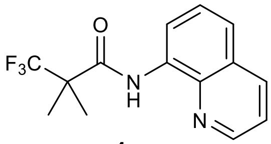

1m

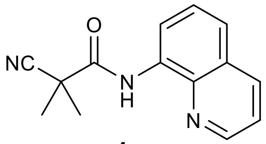

1n

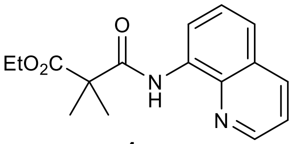

1o

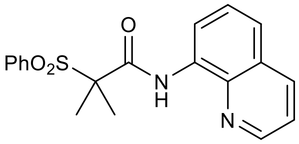

1p

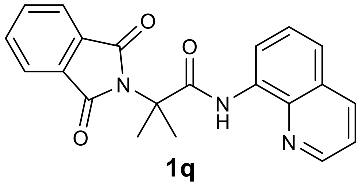

1q

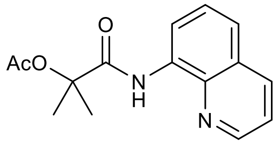

1r1

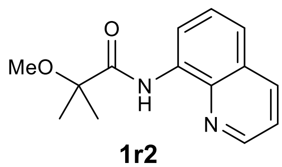

1r2

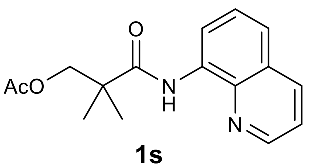

1s

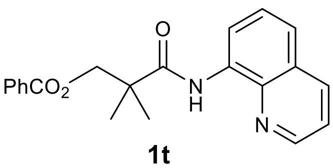

1t

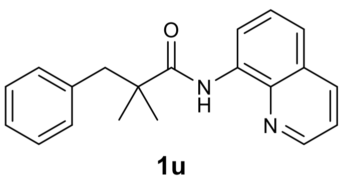

1u

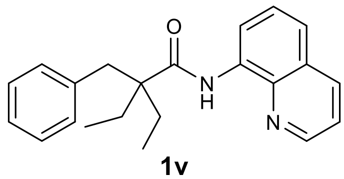

1v

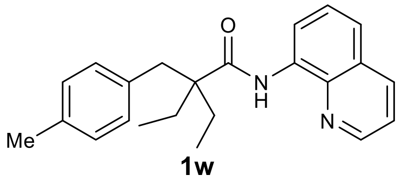

1w

Supplementary Figure 1. Structures of Starting Materials

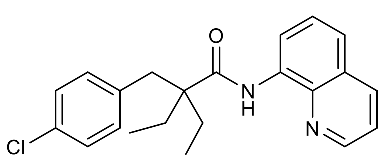
1x

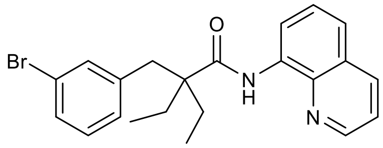
1y

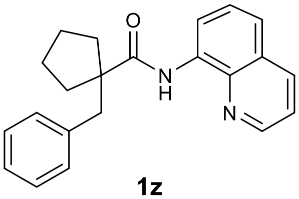
1z

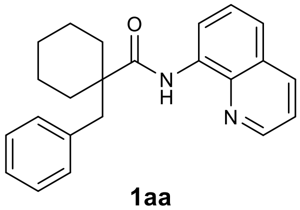
1aa

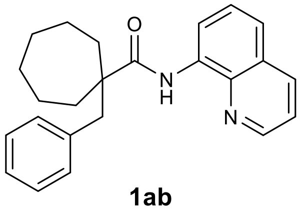
1ab

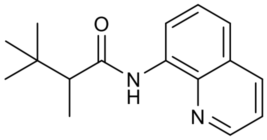
1ac

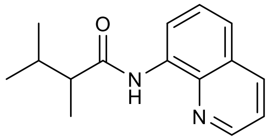
1ad

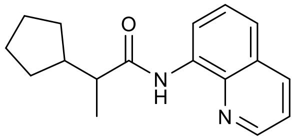
1ae

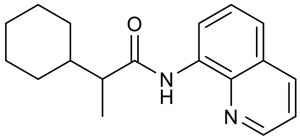
1af

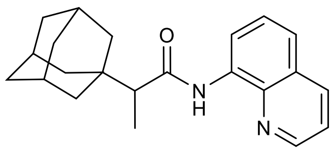
1ag

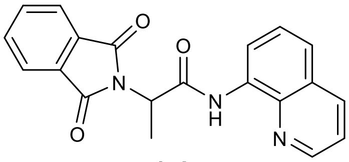
1ah

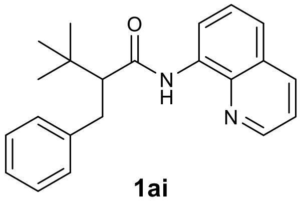
1ai

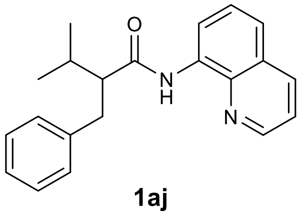
1aj

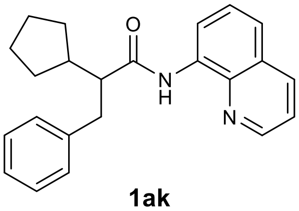
1ak

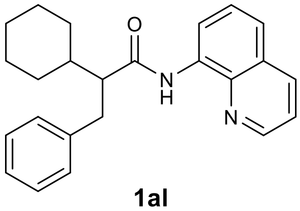
1al

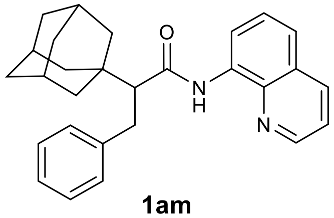
1am

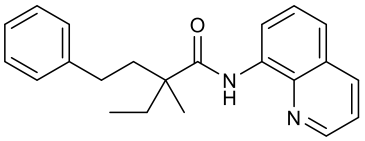
1an

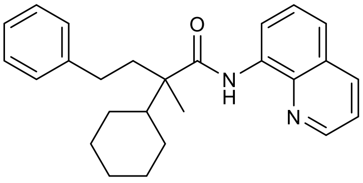
1ao

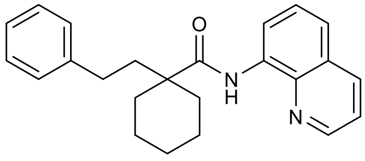
1ap

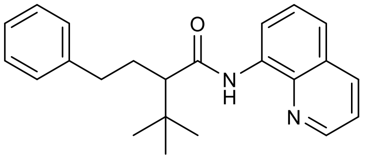
1aq

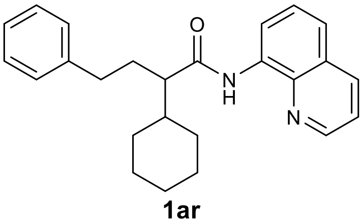
1ar

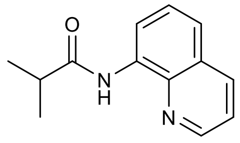
1as

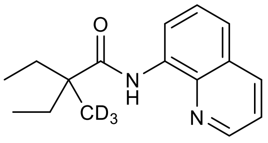
$[D_3]$-**1d**

Supplementary Figure 2. Structures of Starting Materials (continued)

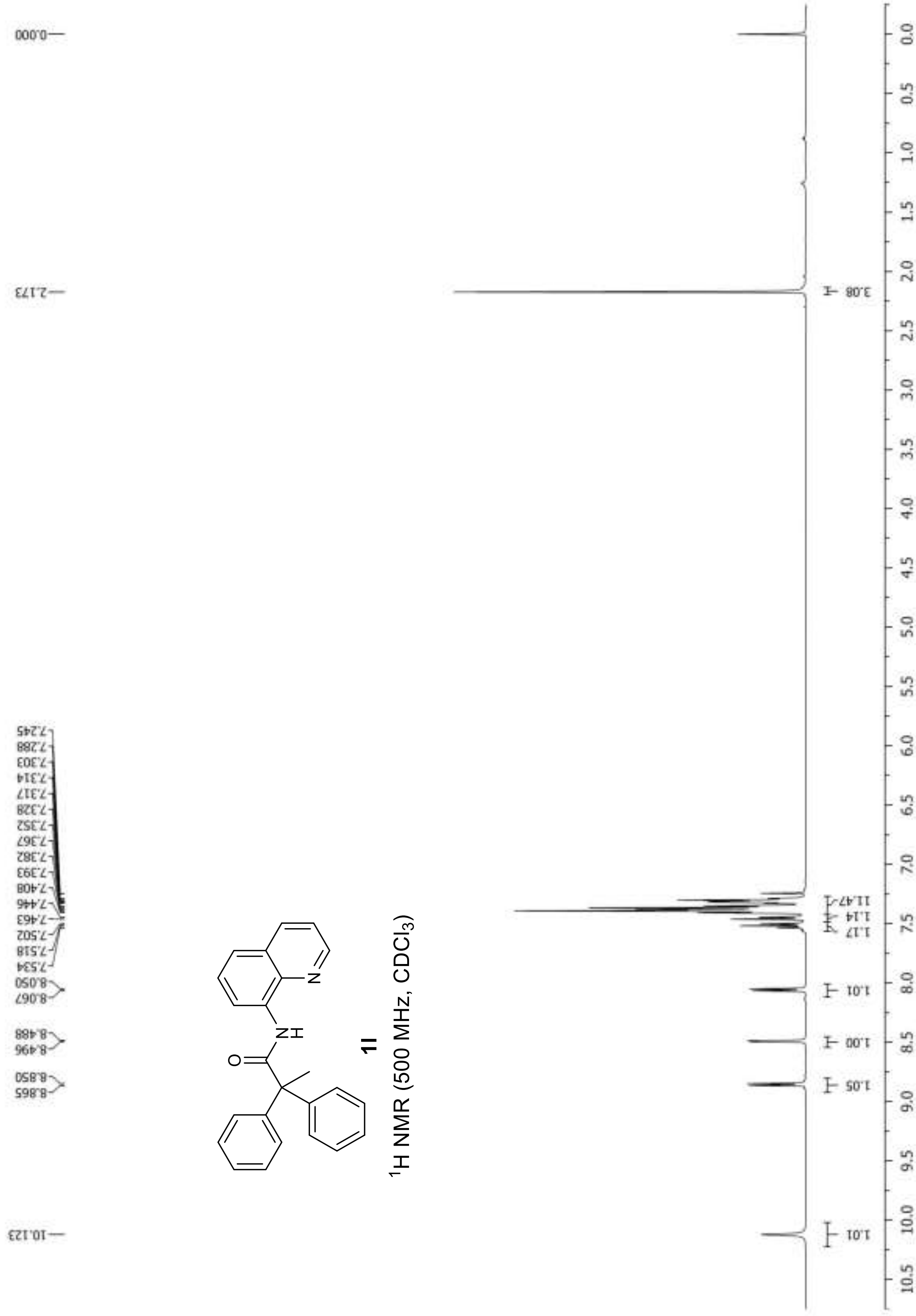

Supplementary Figure 3. ^{1}H NMR (500 MHz, $CDCl_3$) spectrum for 1l.

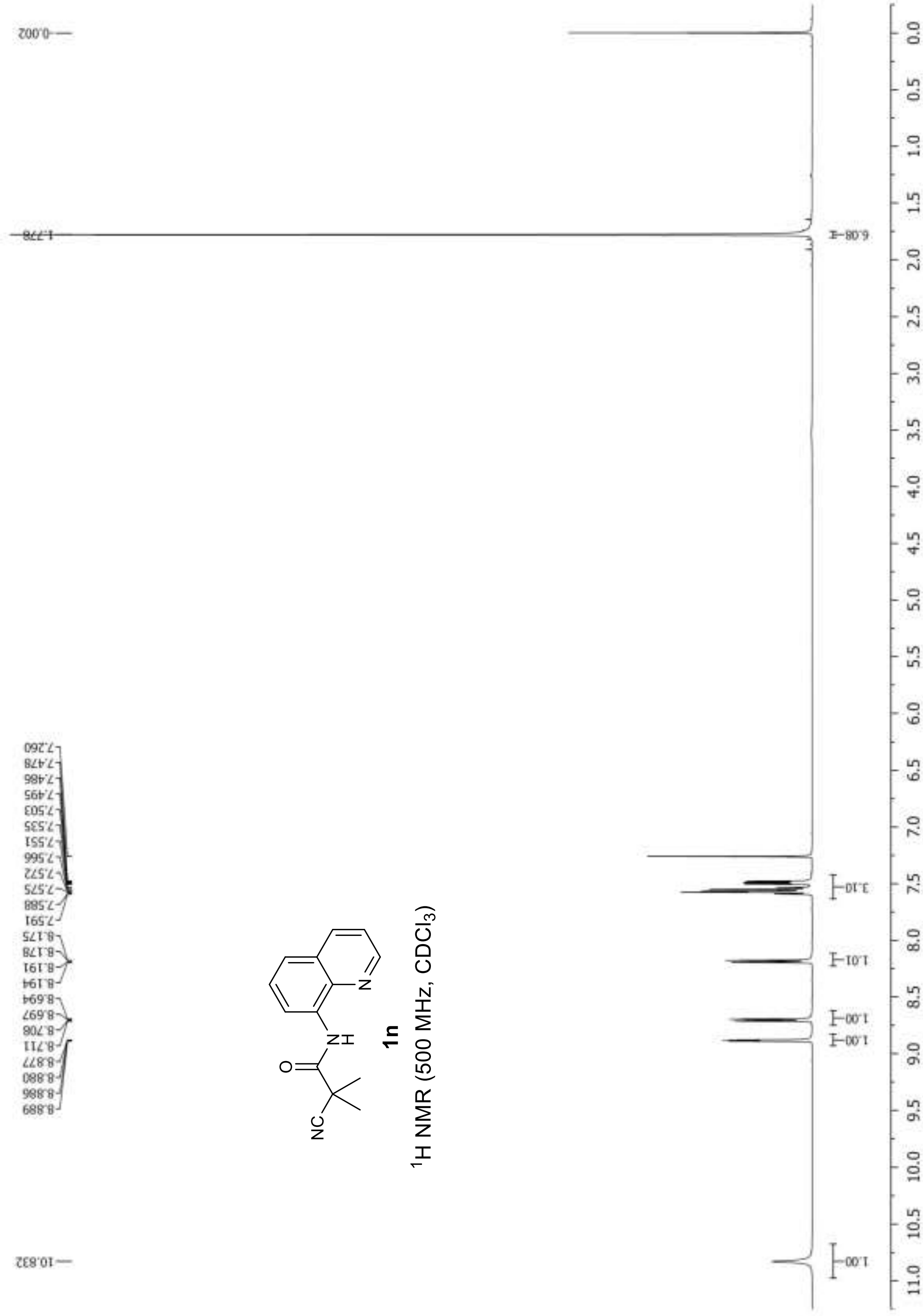

Supplementary Figure 4. ^{1}H NMR (500 MHz, CDCl$_3$) spectrum for 1n.

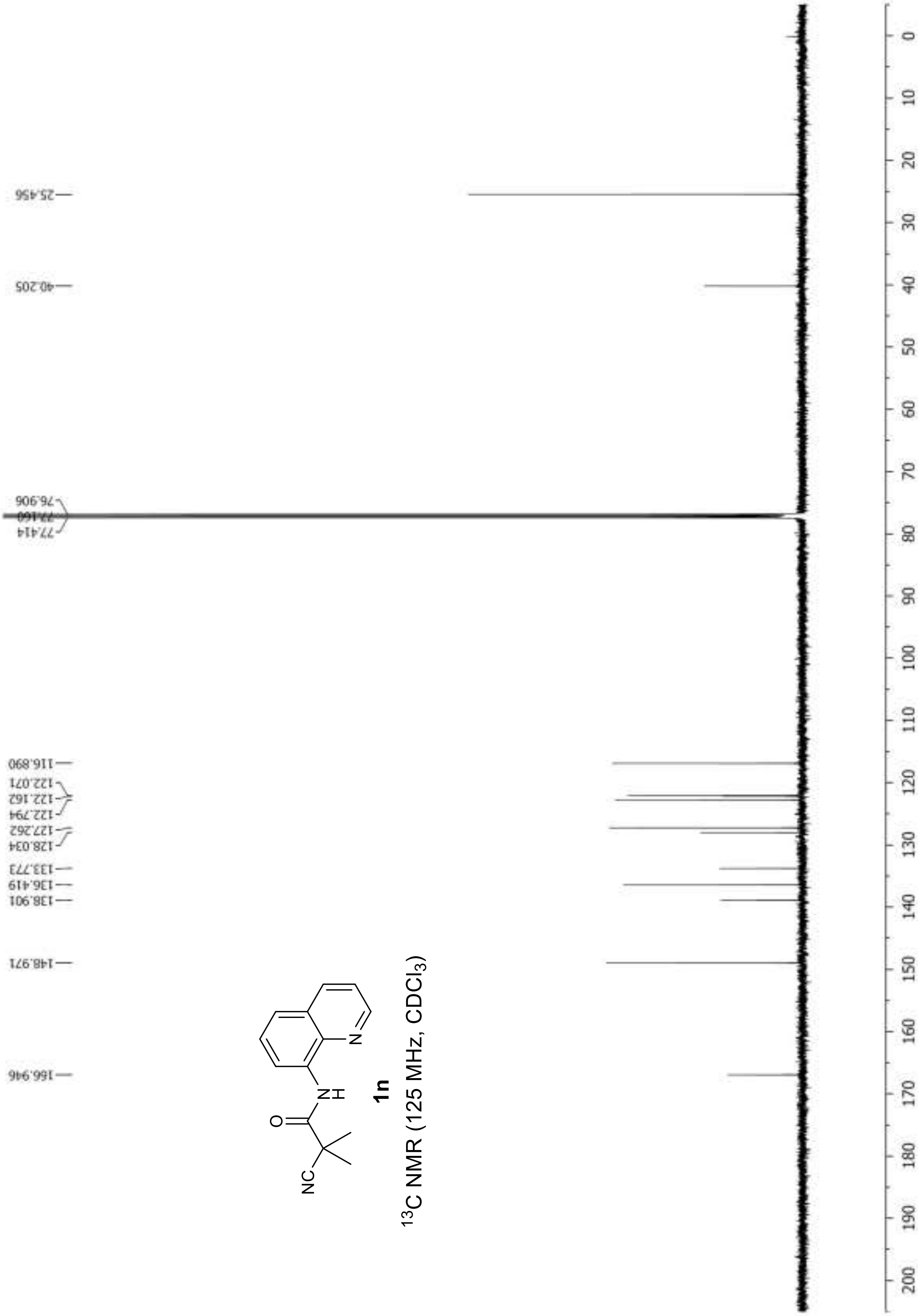

Supplementary Figure 5. ^{13}C NMR (125 MHz, $CDCl_3$) spectrum for 1n.

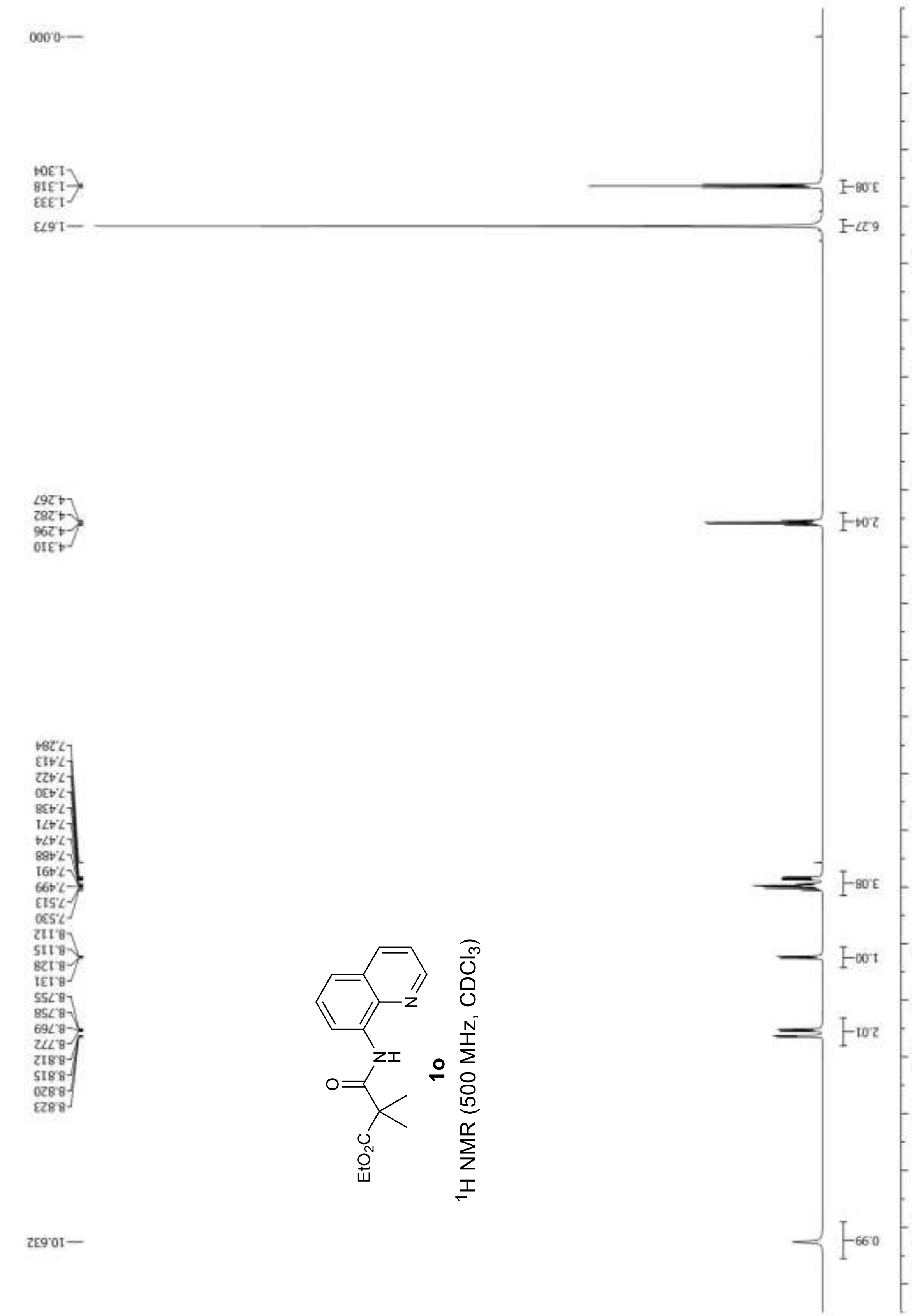

Supplementary Figure 6. ^{1}H NMR (500 MHz, $CDCl_3$) spectrum for 1o.

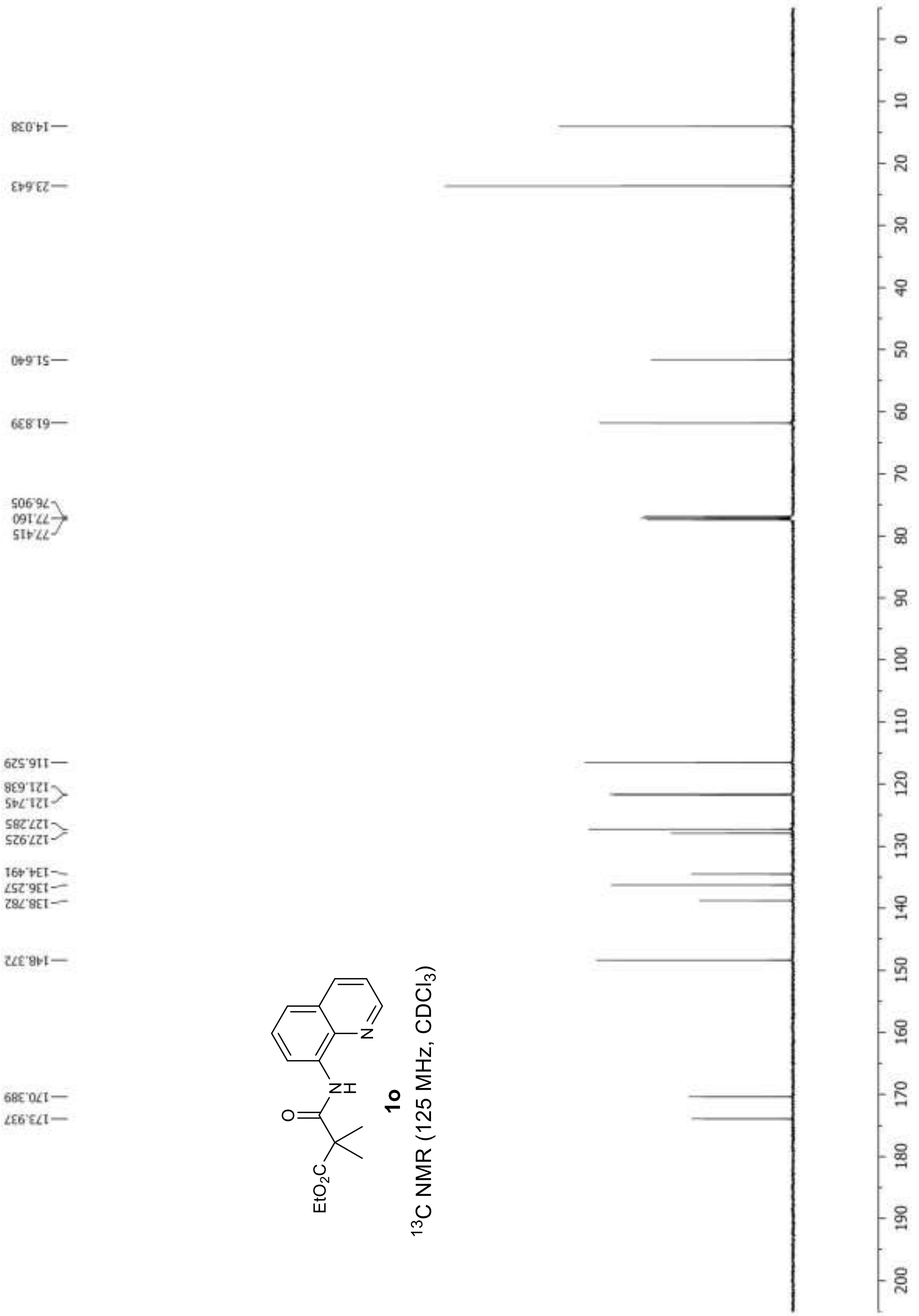

Supplementary Figure 7. ^{13}C NMR (125 MHz, $CDCl_3$) spectrum for 1o.

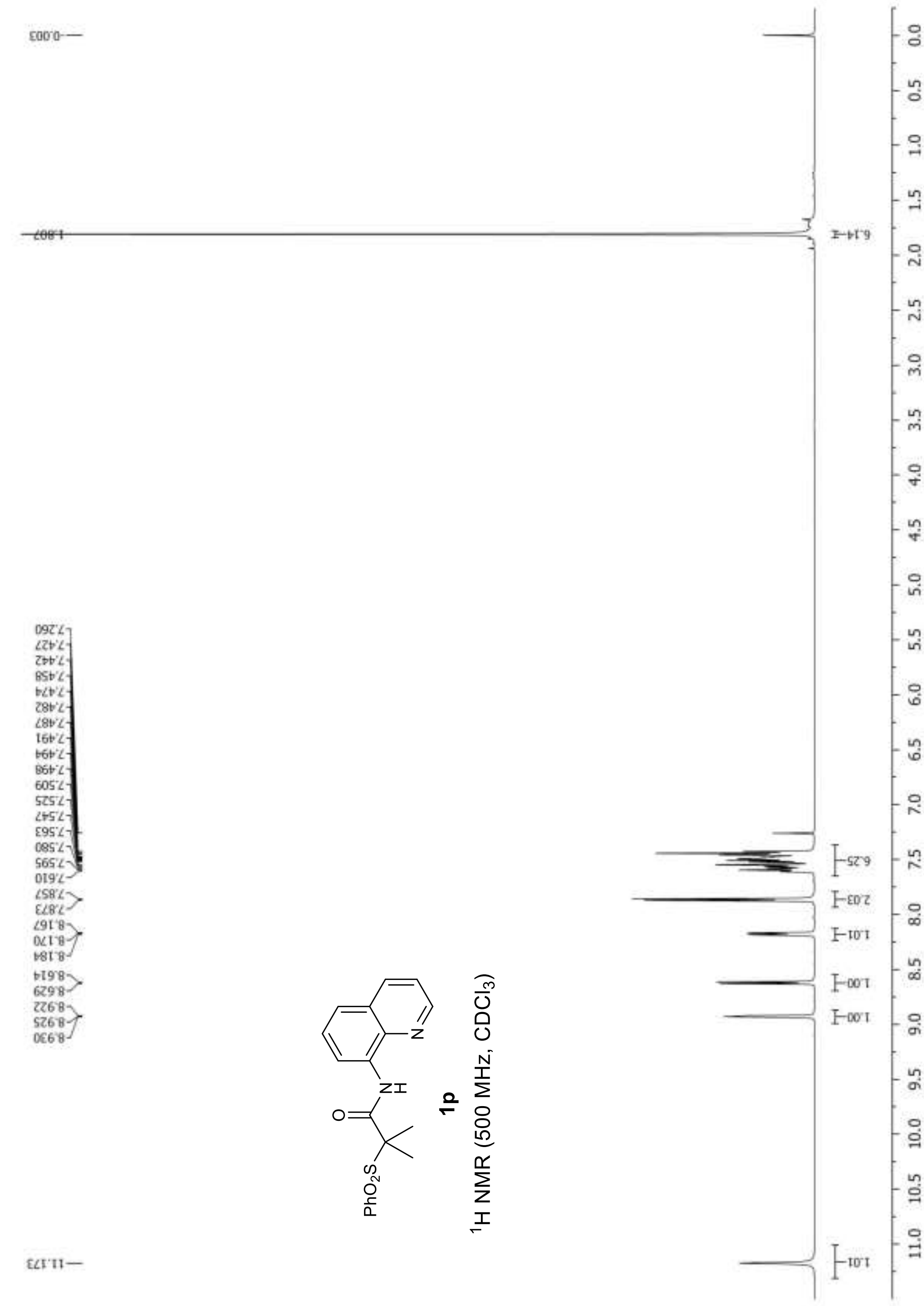

Supplementary Figure 8. ^{1}H NMR (500 MHz, $CDCl_3$) spectrum for 1p.

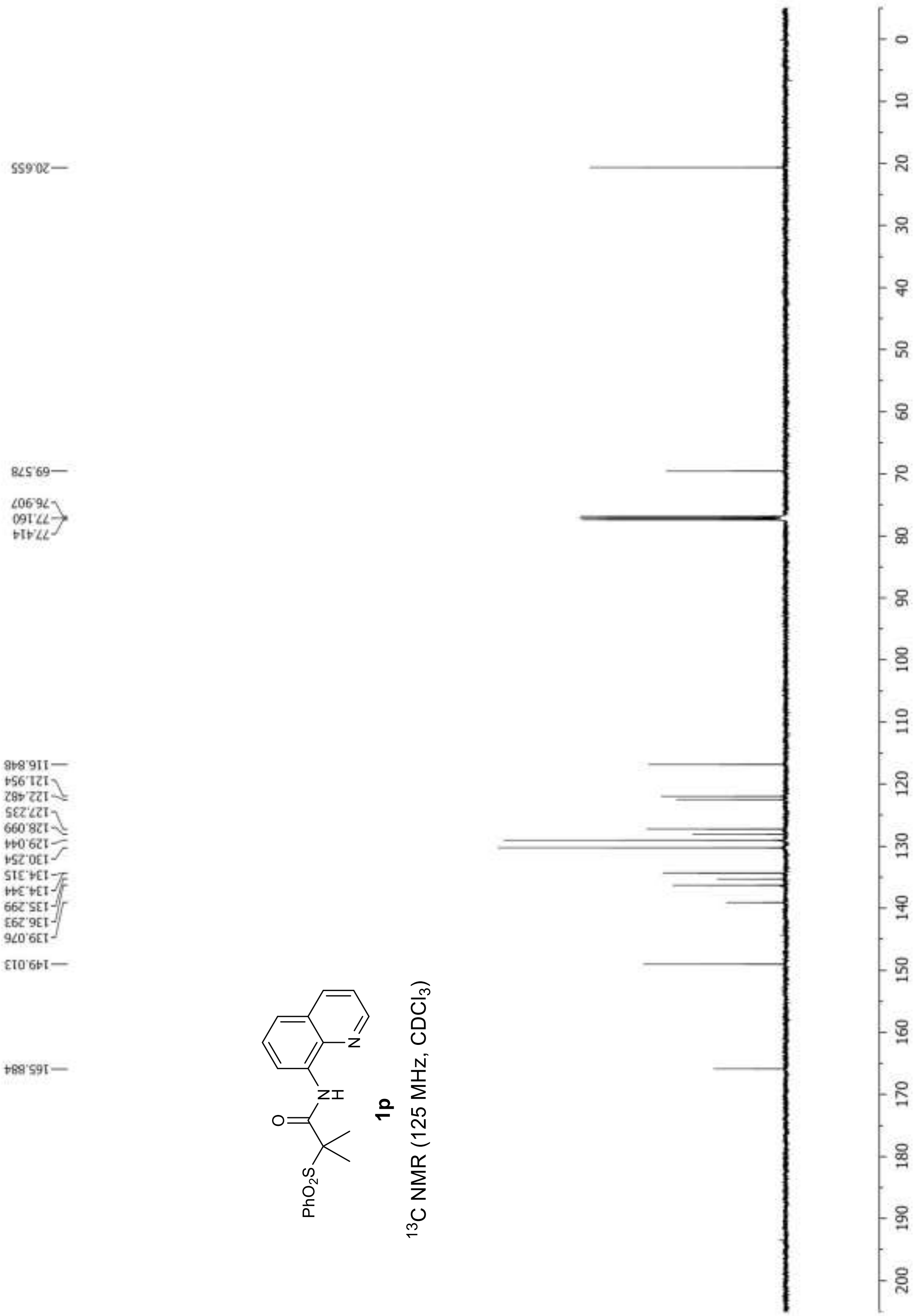

Supplementary Figure 9. ^{13}C NMR (125 MHz, $CDCl_3$) spectrum for 1p.

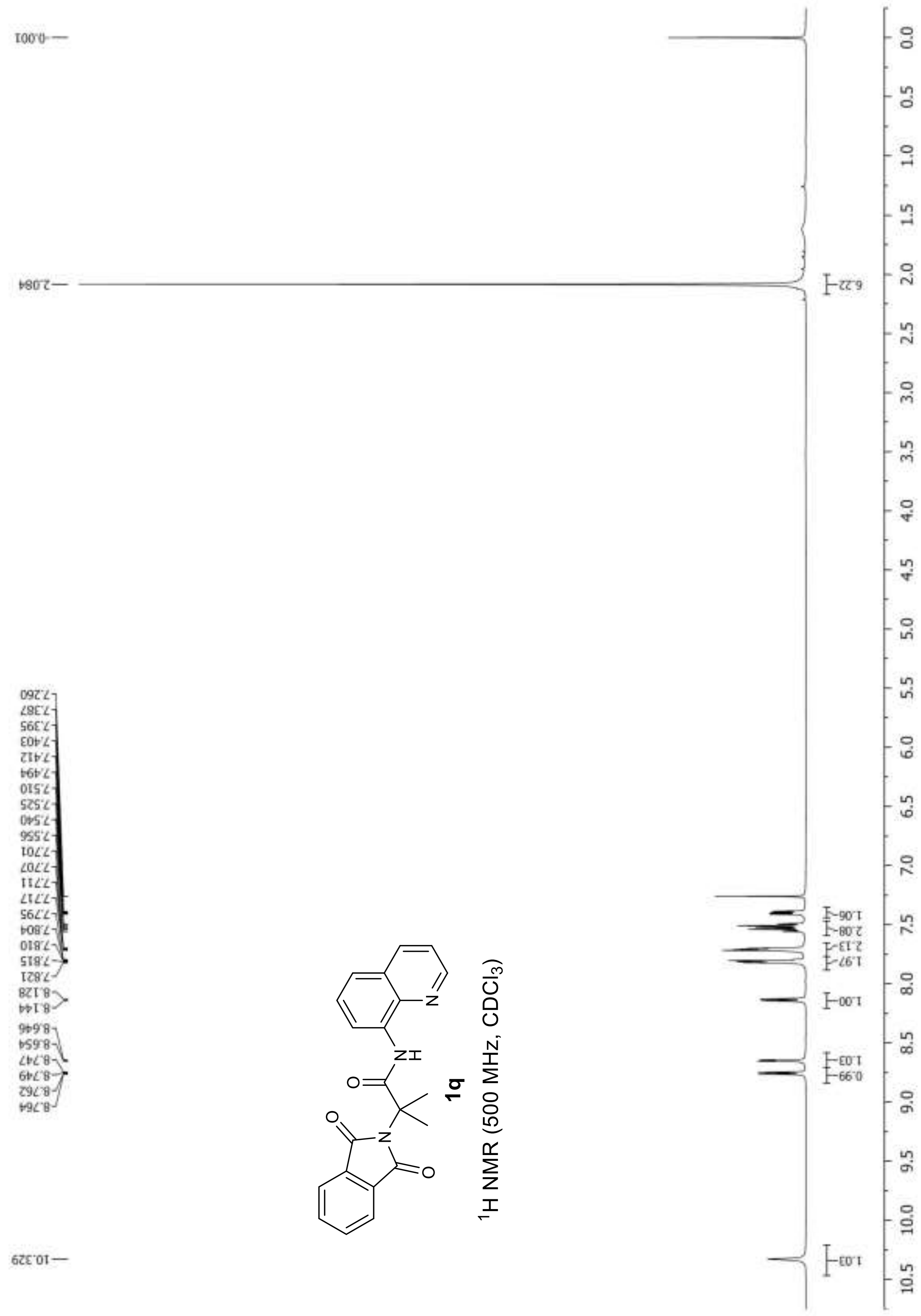

Supplementary Figure 10. ^{1}H NMR (500 MHz, $CDCl_3$) spectrum for 1q.

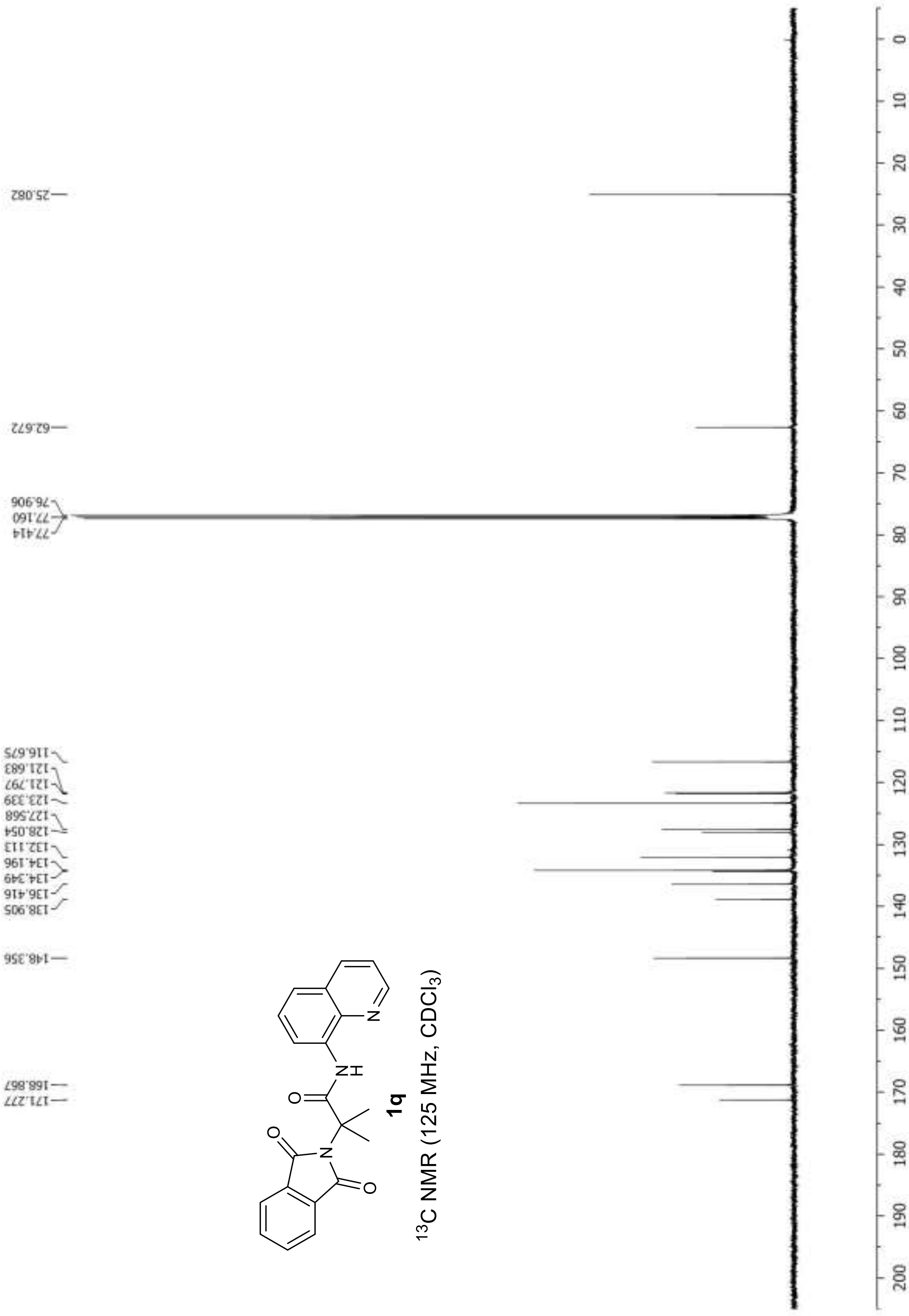

Supplementary Figure 11. ^{13}**C NMR (125 MHz, CDCl**$_3$**) spectrum for 1q.**

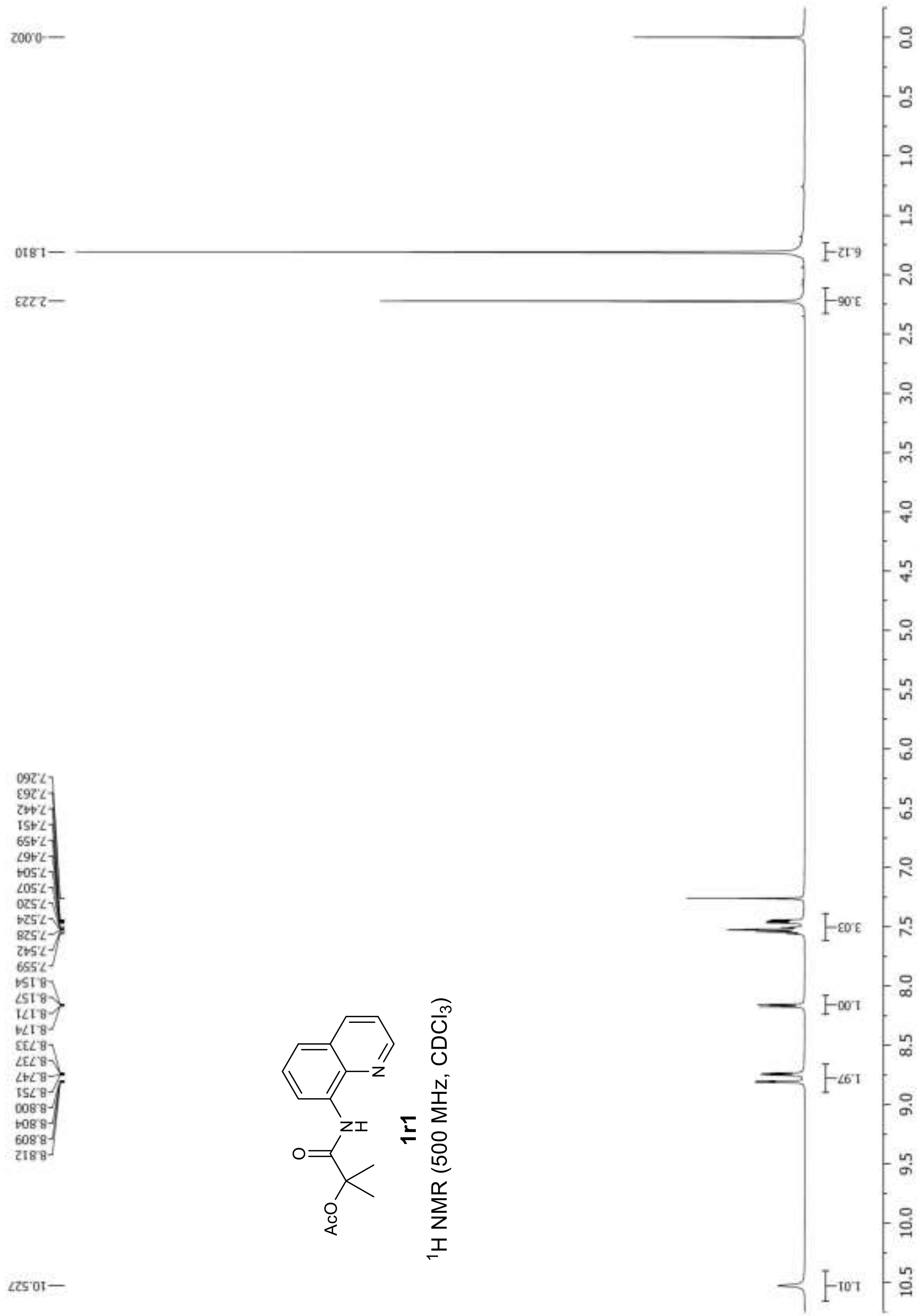

Supplementary Figure 12. ^{1}H NMR (500 MHz, $CDCl_3$) spectrum for 1r1.

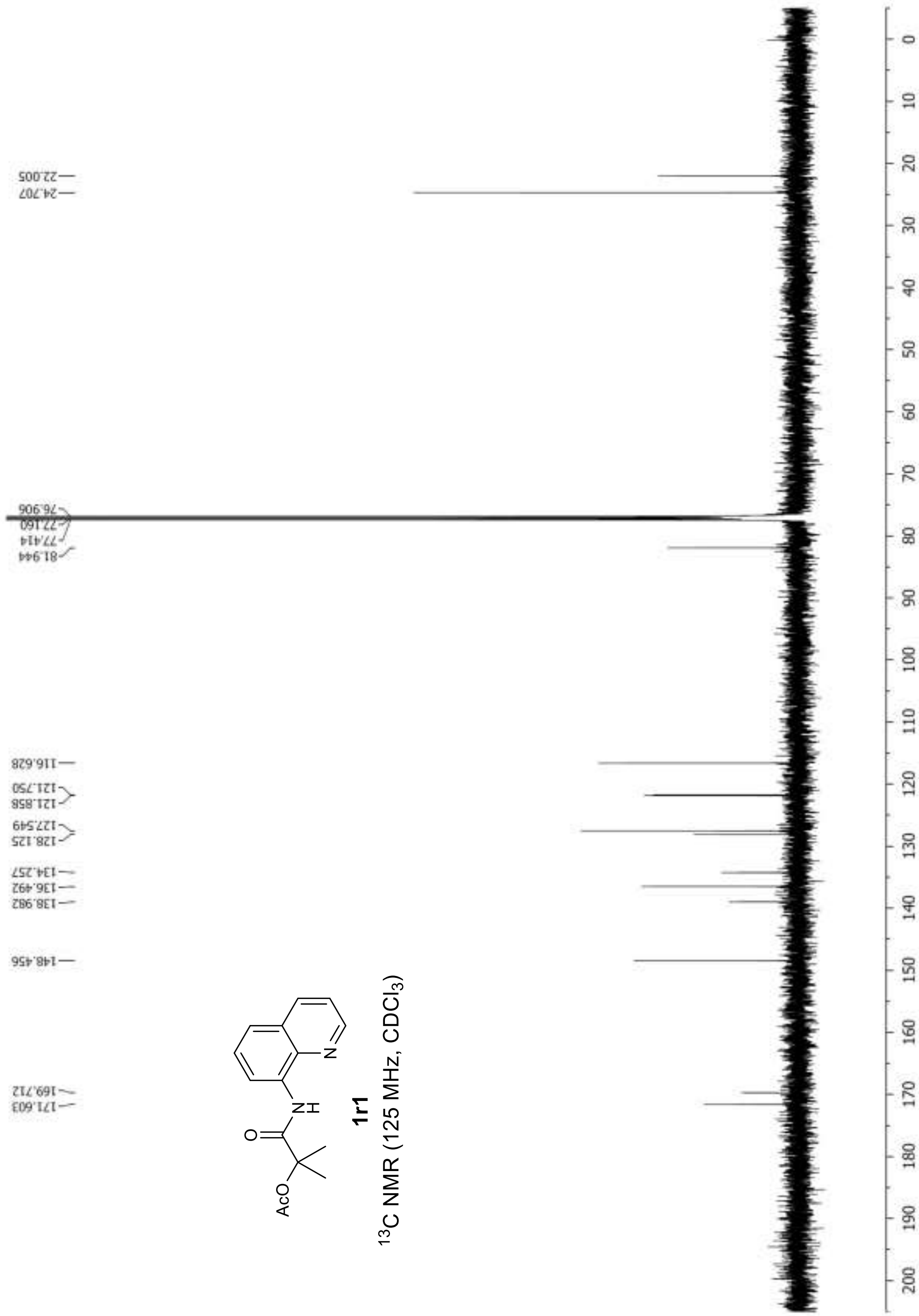

Supplementary Figure 13. ^{13}**C NMR (125 MHz,** $CDCl_3$**) spectrum for 1r1.**

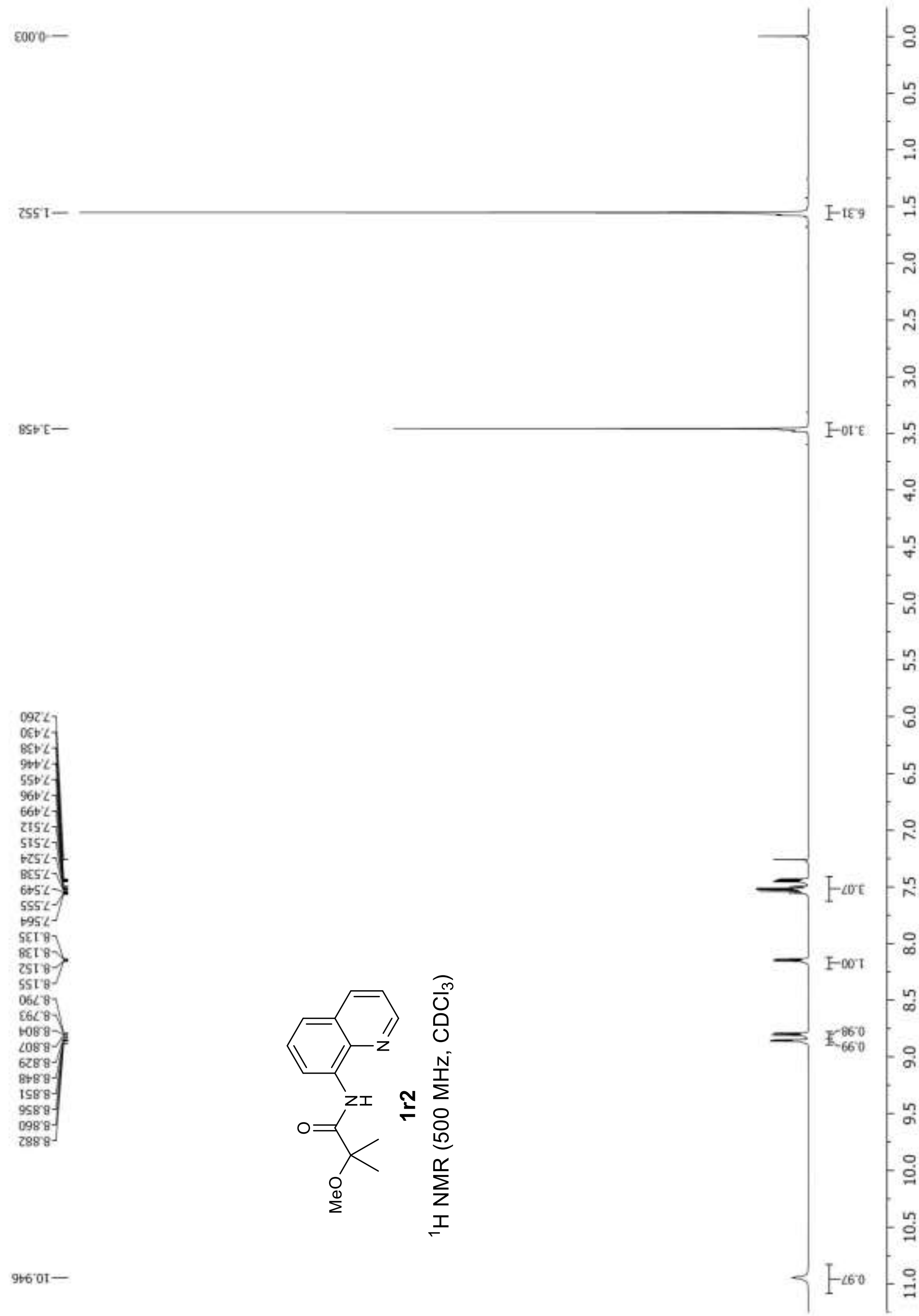

Supplementary Figure 14. ^{1}H NMR (500 MHz, $CDCl_3$) spectrum for 1r2.

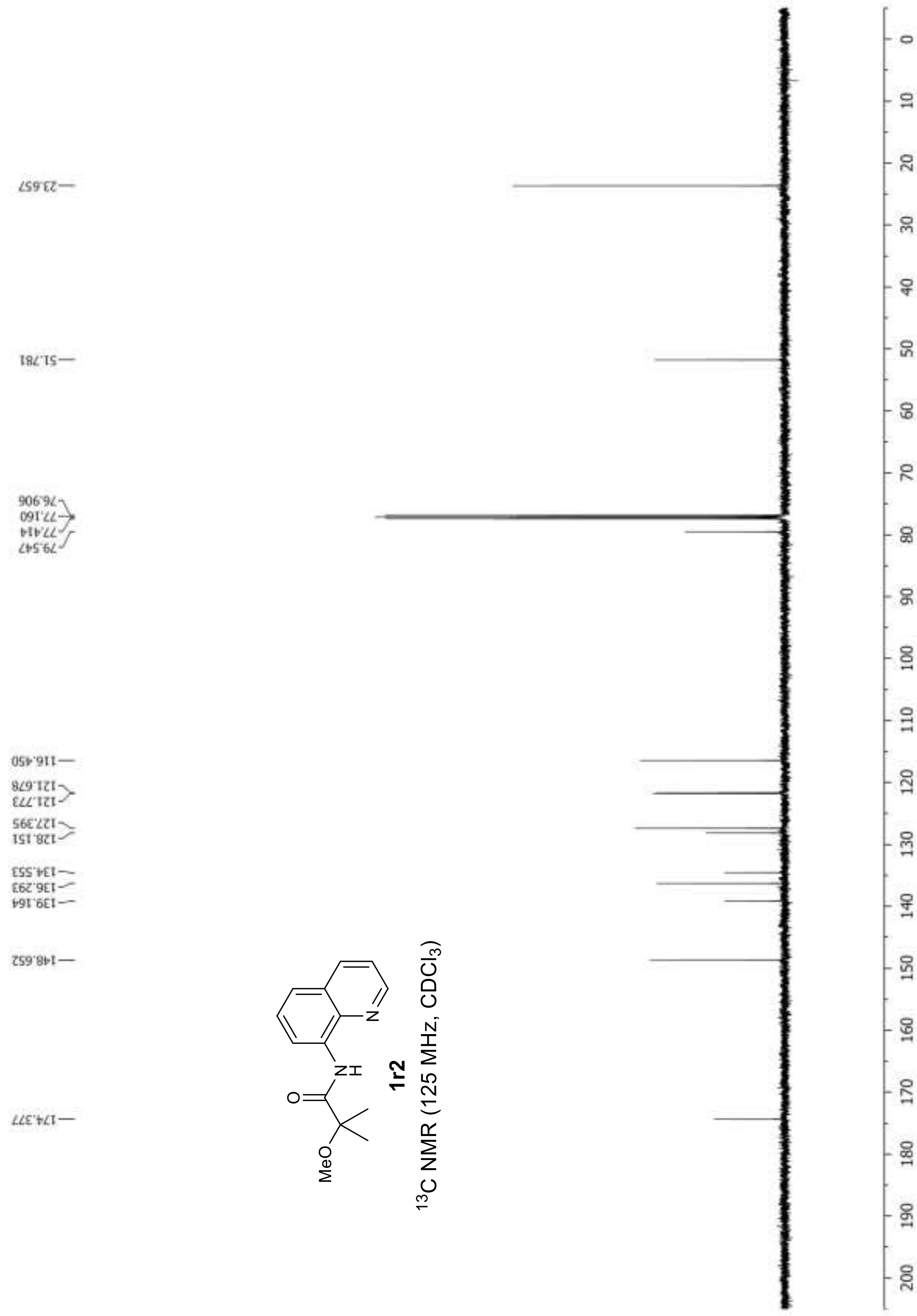

Supplementary Figure 15. ^{13}C NMR (125 MHz, $CDCl_3$) spectrum for 1r2.

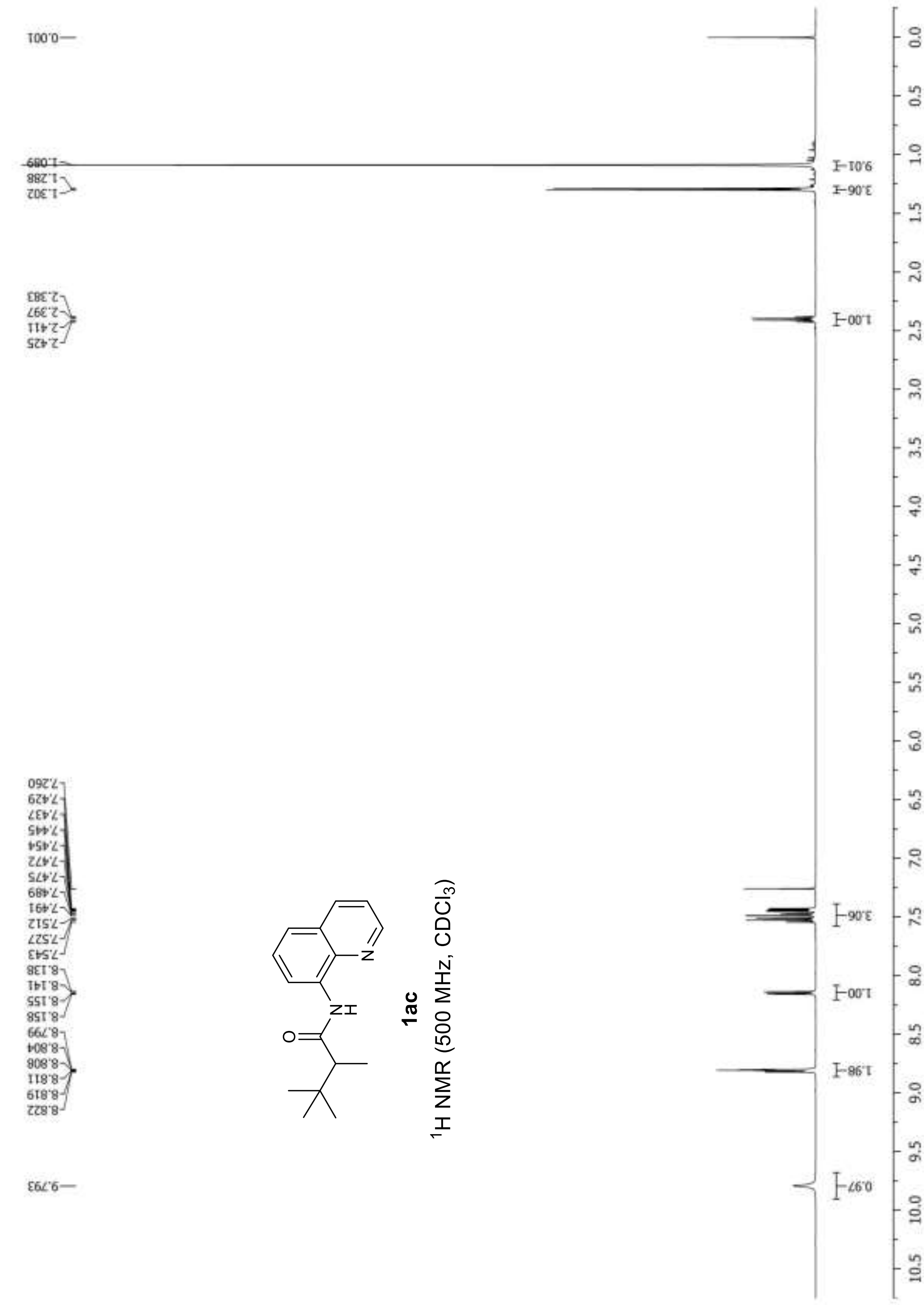

Supplementary Figure 16. ^{1}H NMR (500 MHz, $CDCl_3$) spectrum for 1ac.

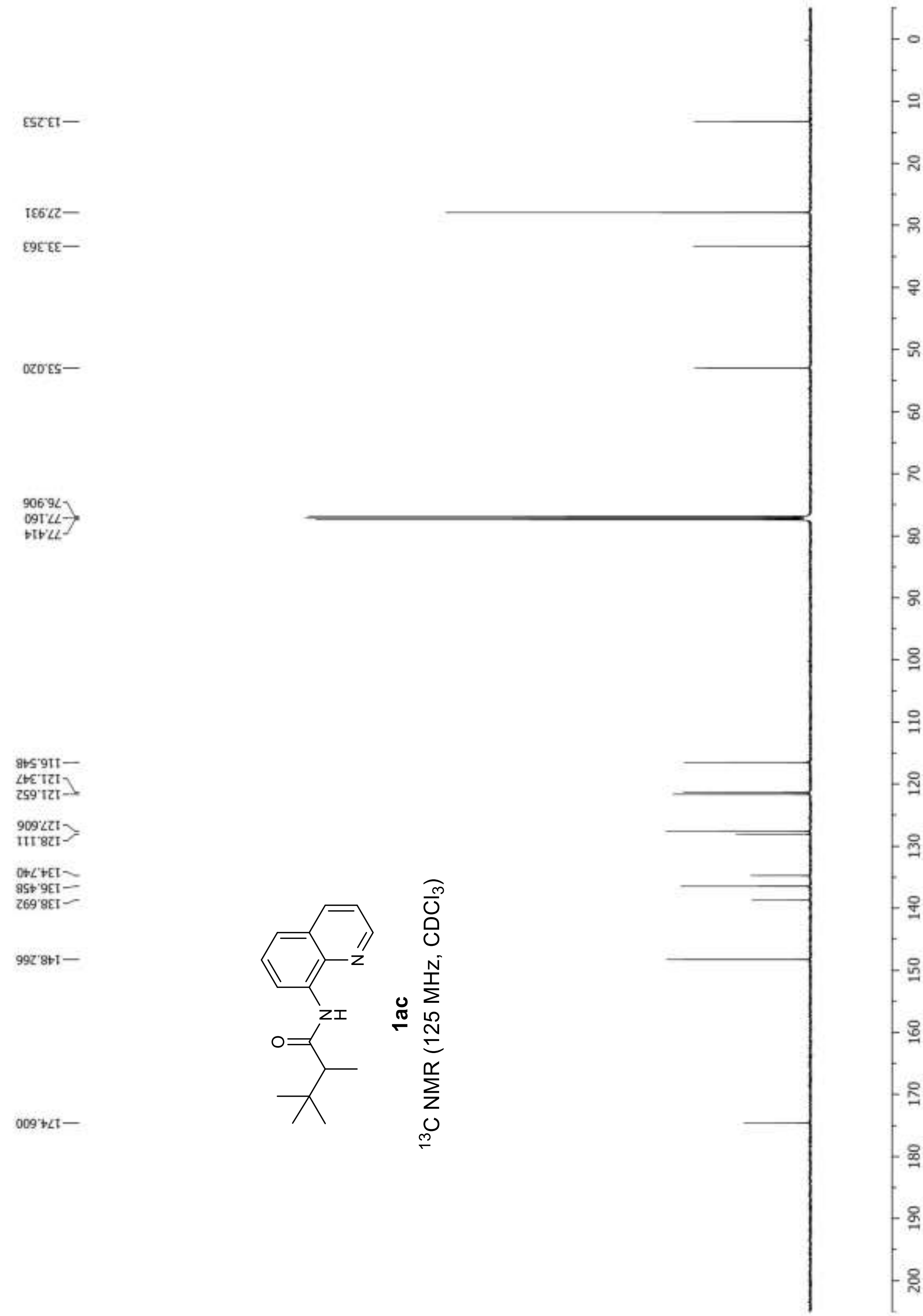

Supplementary Figure 17. ^{13}C NMR (125 MHz, $CDCl_3$) spectrum for 1ac.

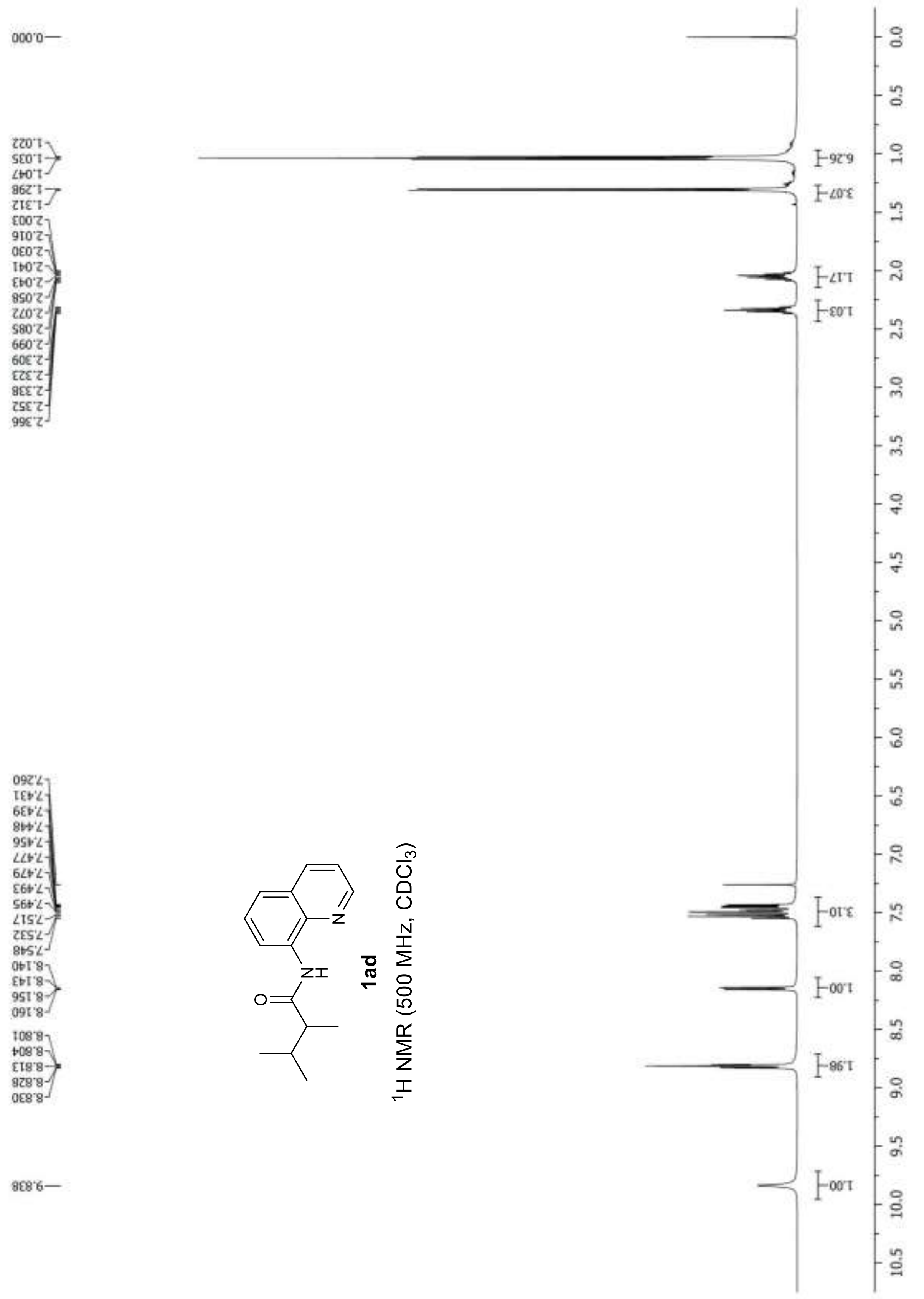

Supplementary Figure 18. ^{1}H NMR (500 MHz, $CDCl_3$) spectrum for 1ad.

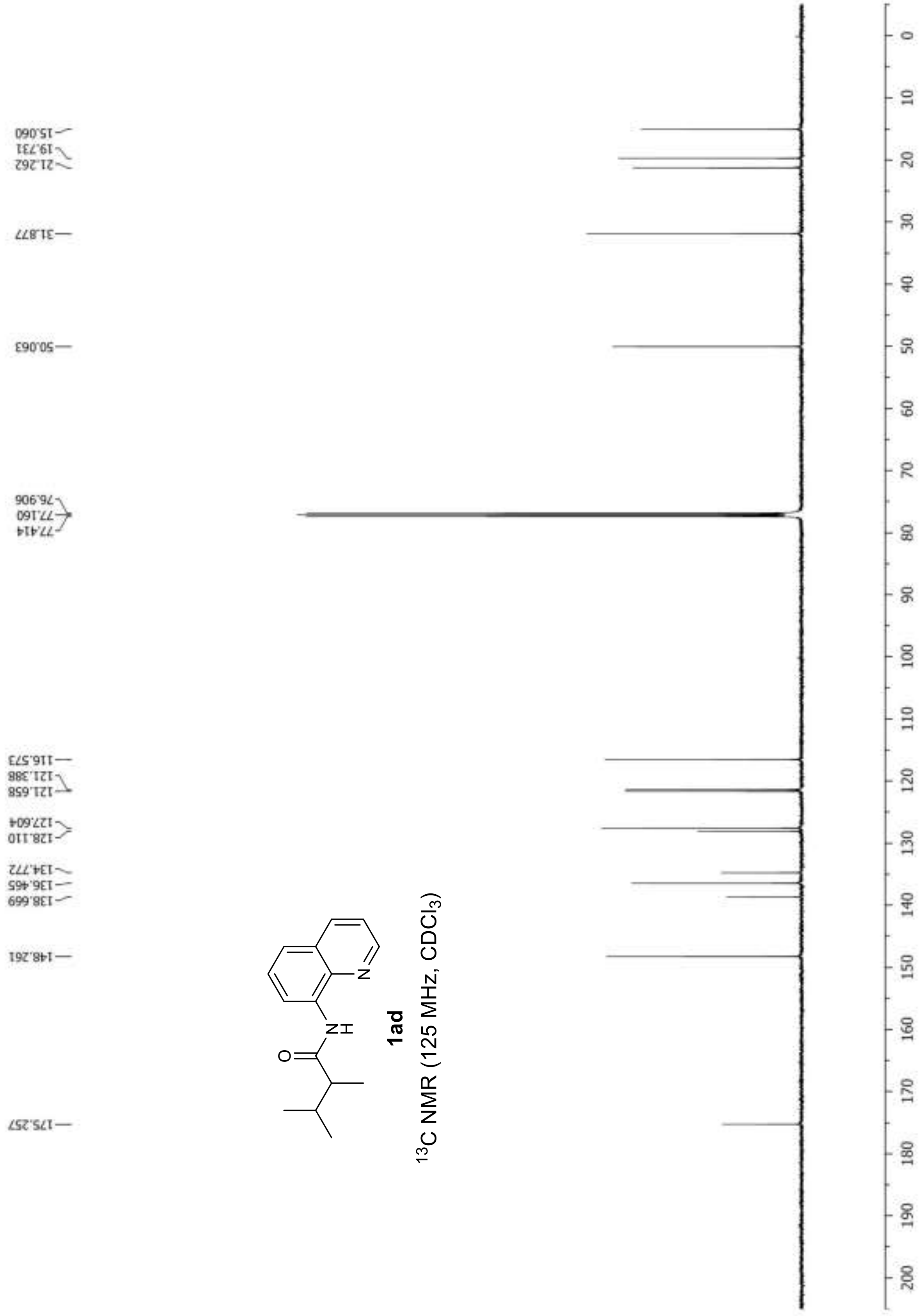

Supplementary Figure 19. ^{13}C NMR (125 MHz, $CDCl_3$) spectrum for 1ad.

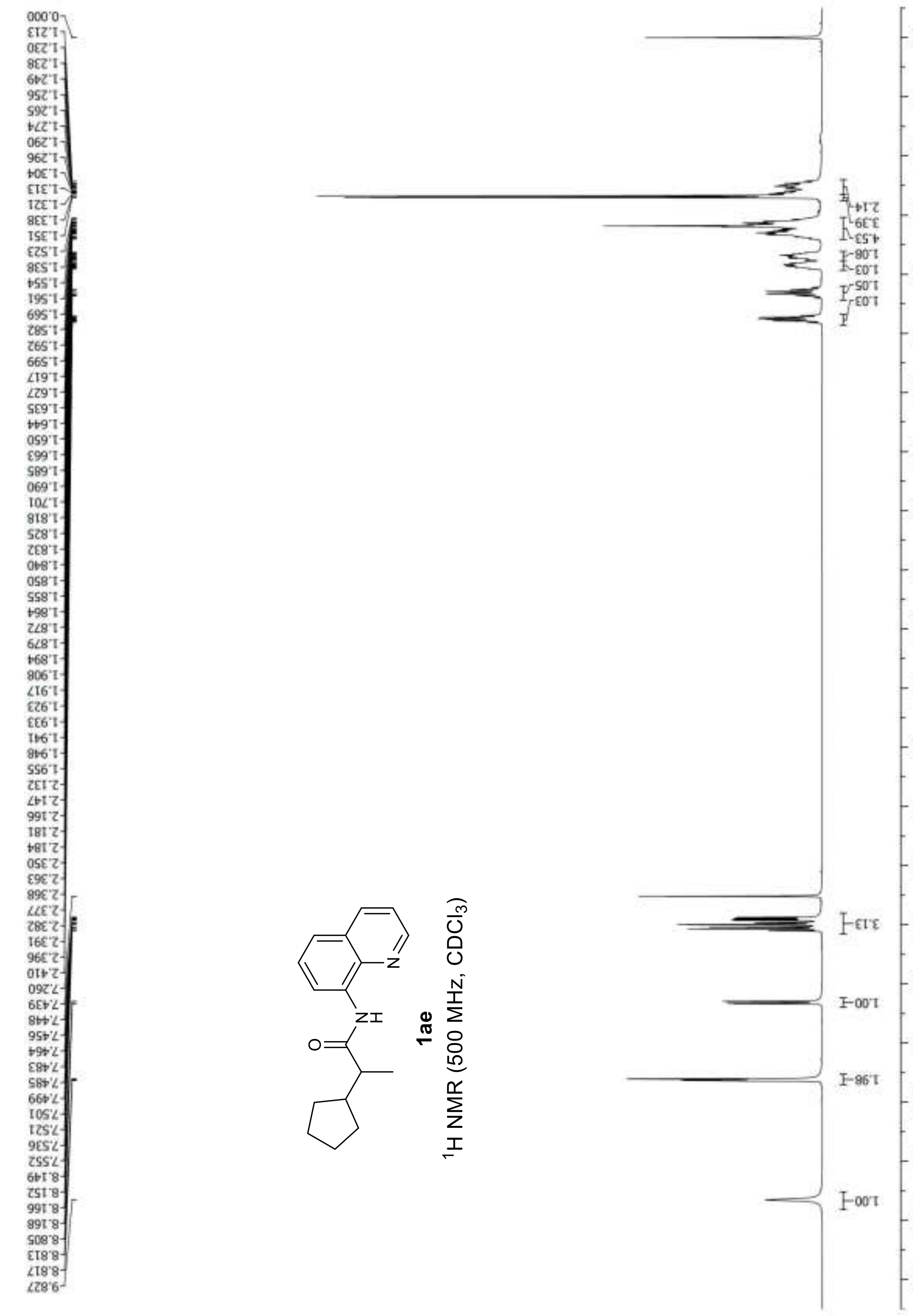

Supplementary Figure 20. ^{1}H NMR (500 MHz, $CDCl_3$) spectrum for 1ae.

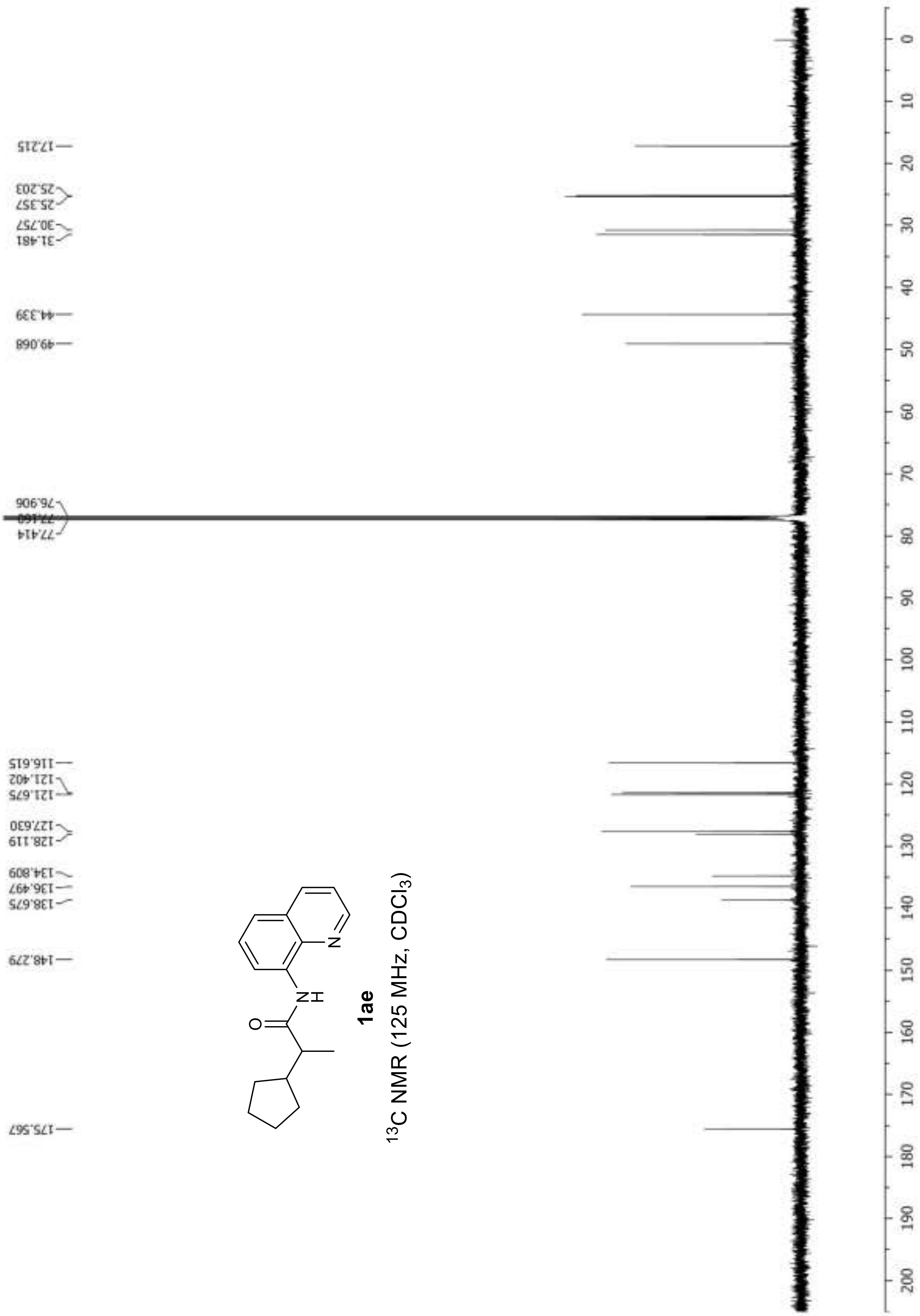

Supplementary Figure 21. **^{13}C NMR (125 MHz, $CDCl_3$) spectrum for 1ae.**

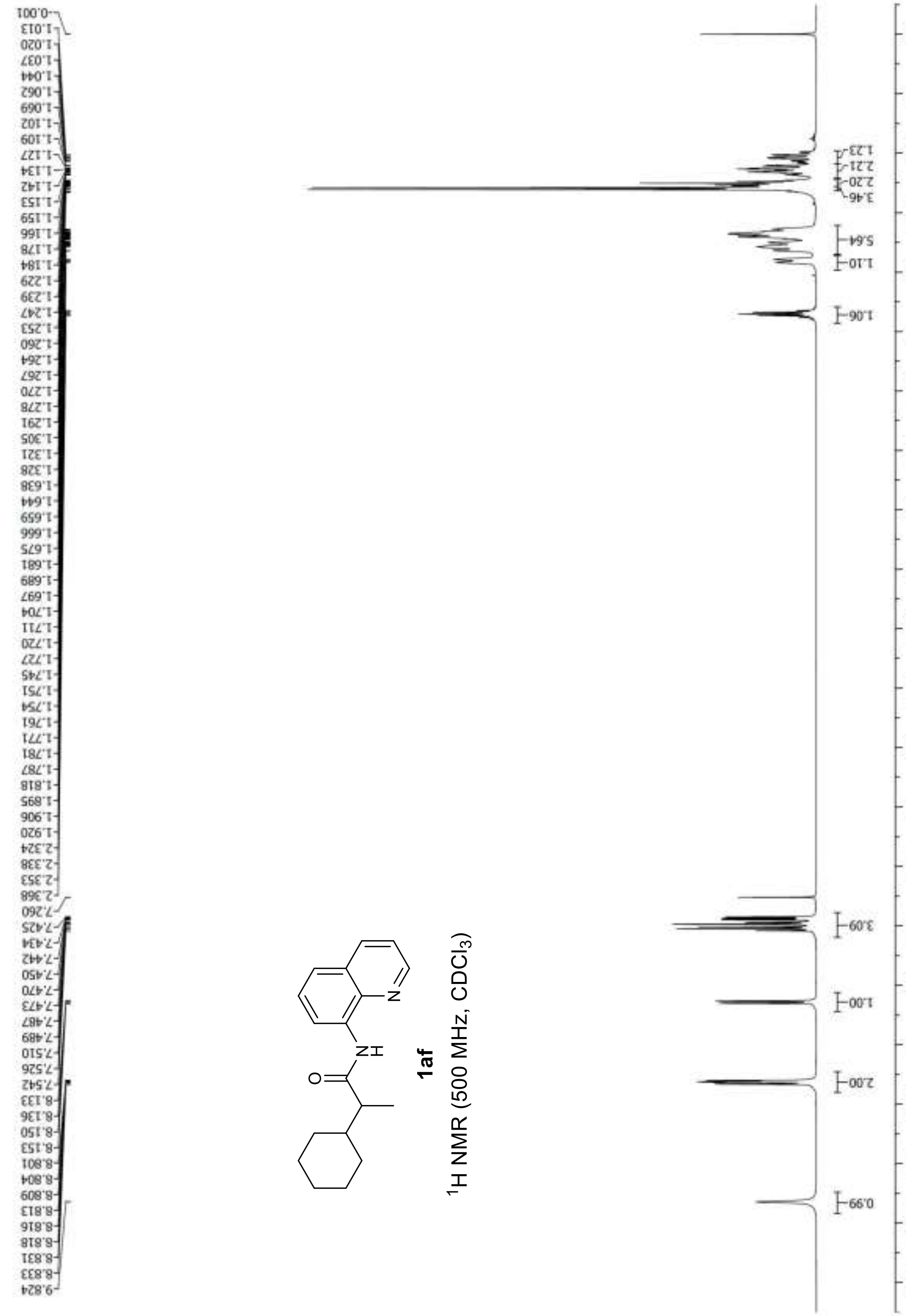

Supplementary Figure 22. ^{1}H NMR (500 MHz, $CDCl_3$) spectrum for 1af.

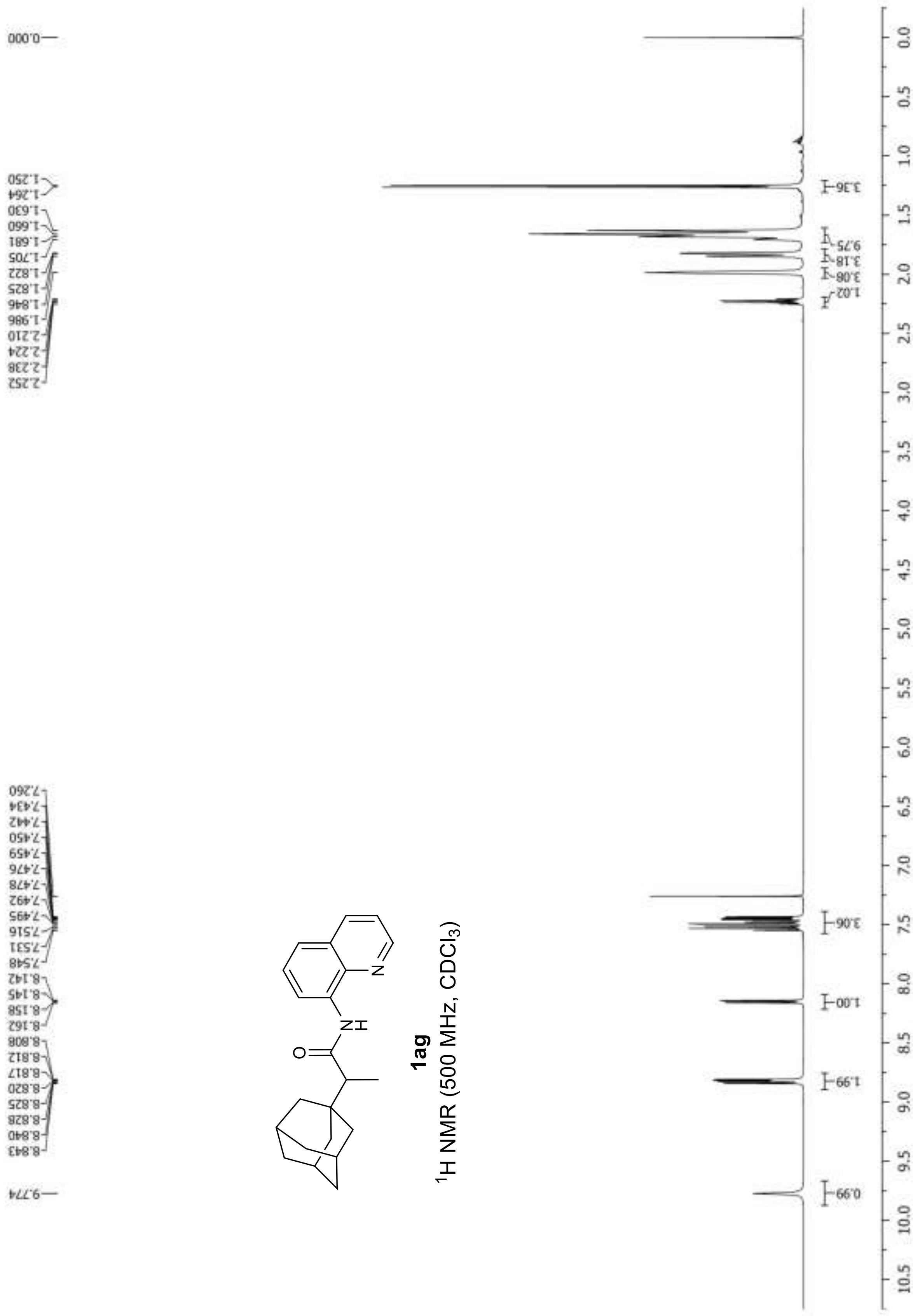

Supplementary Figure 23. ^{1}H NMR (500 MHz, $CDCl_3$) spectrum for 1ag.

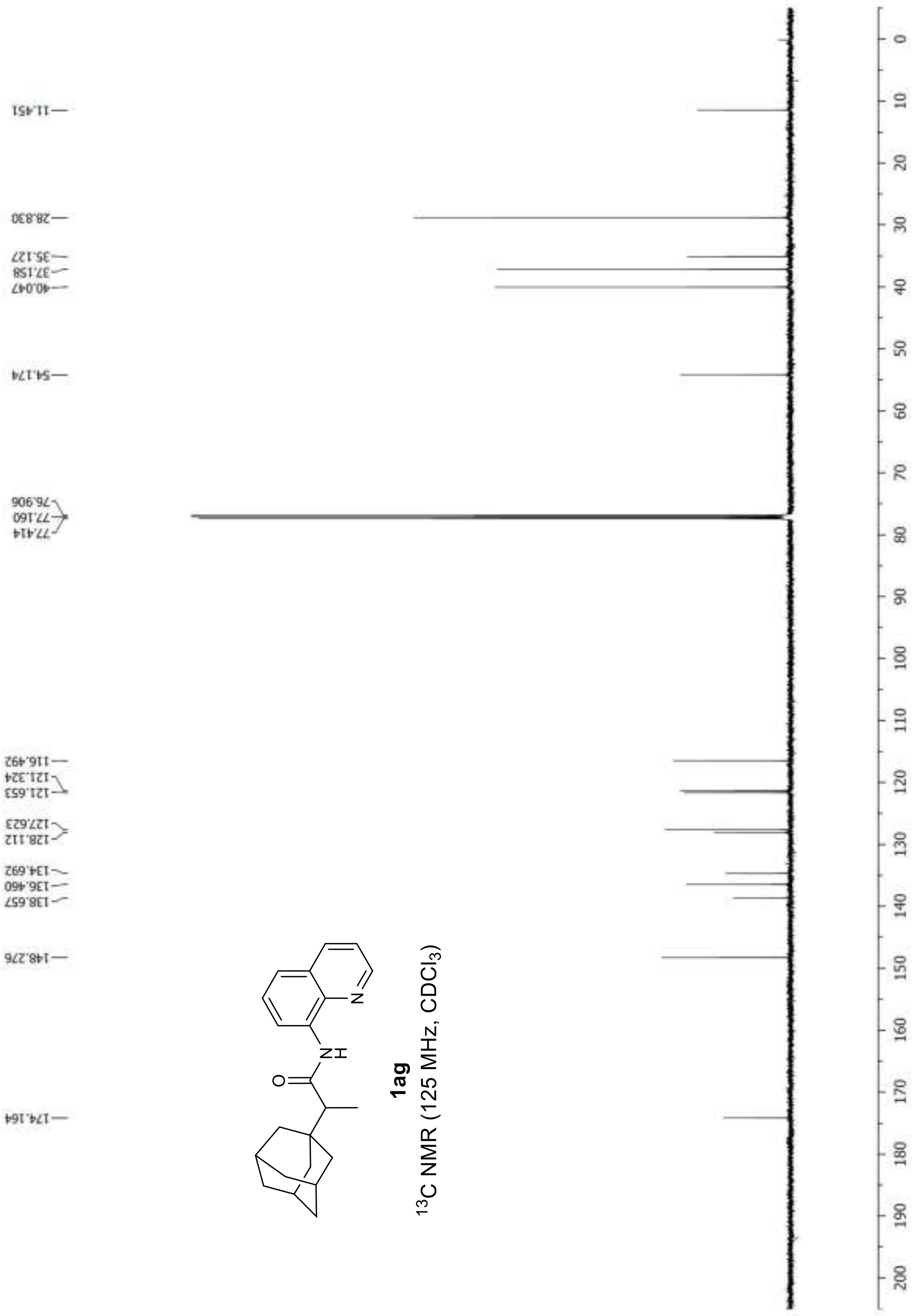

Supplementary Figure 24. ^{13}C NMR (125 MHz, $CDCl_3$) spectrum for 1ag.

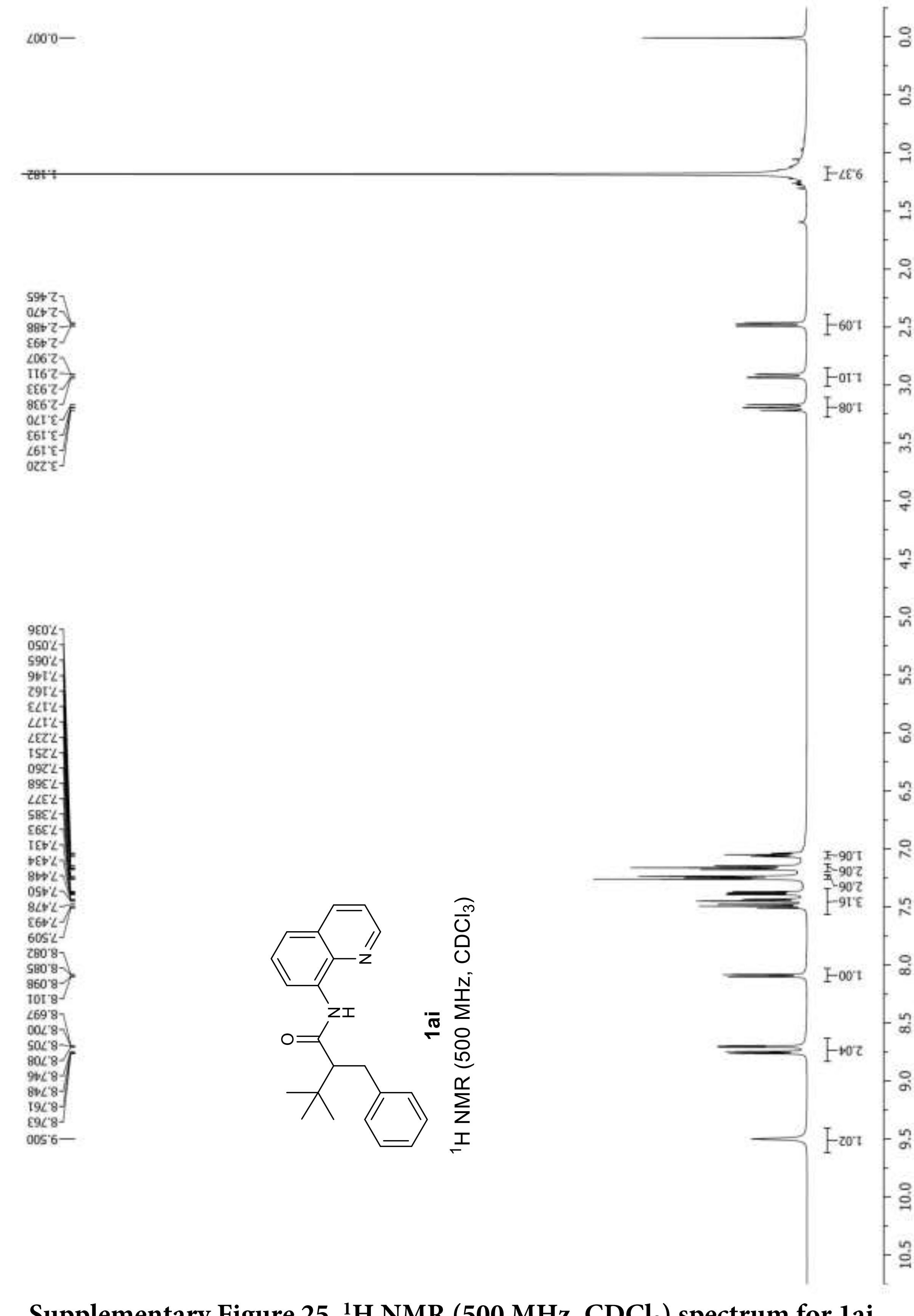

Supplementary Figure 25. ^{1}H NMR (500 MHz, $CDCl_3$) spectrum for 1ai.

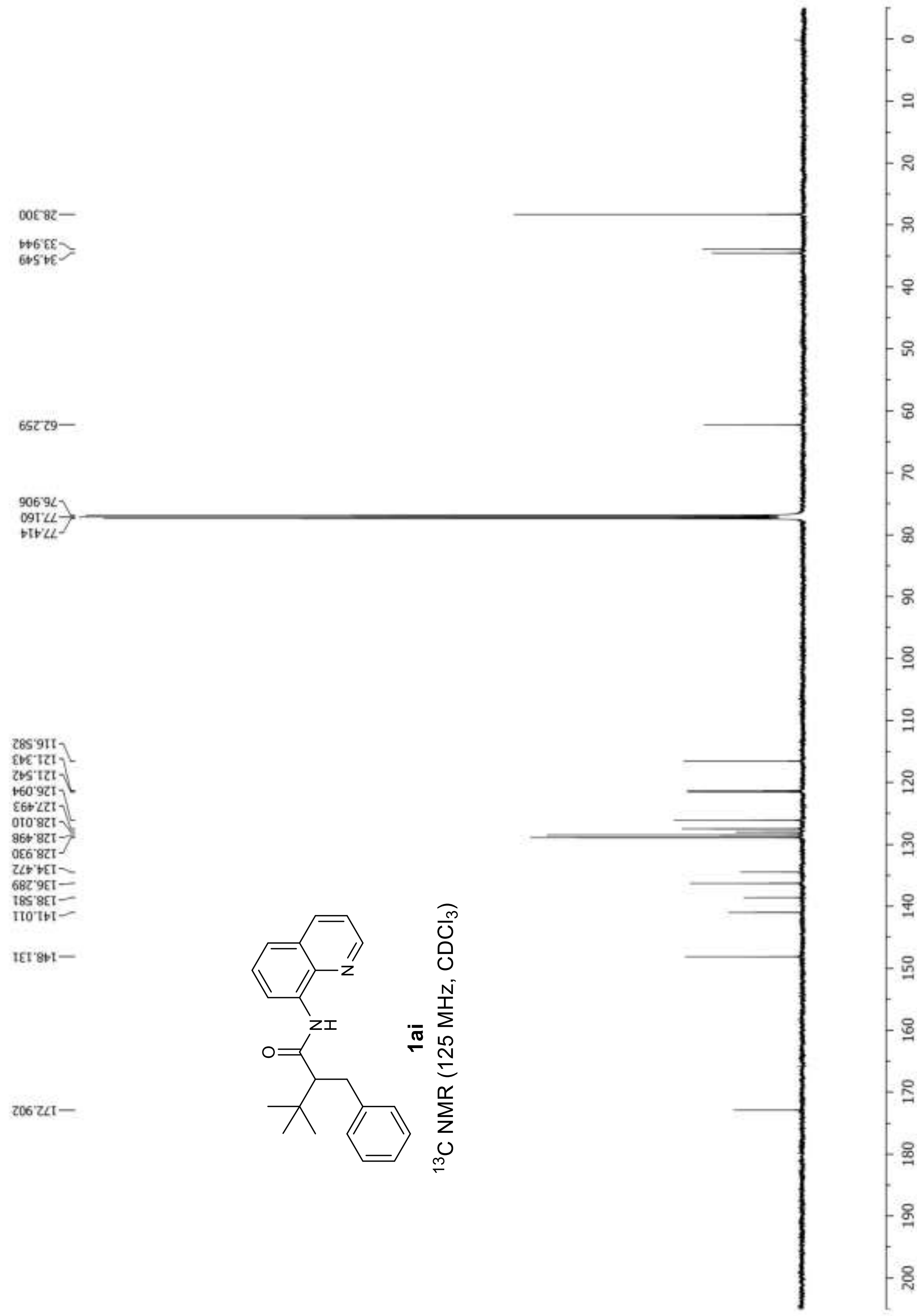

upplementary Figure 26. ^{13}C NMR (125 MHz, $CDCl_3$) spectrum for 1ai.

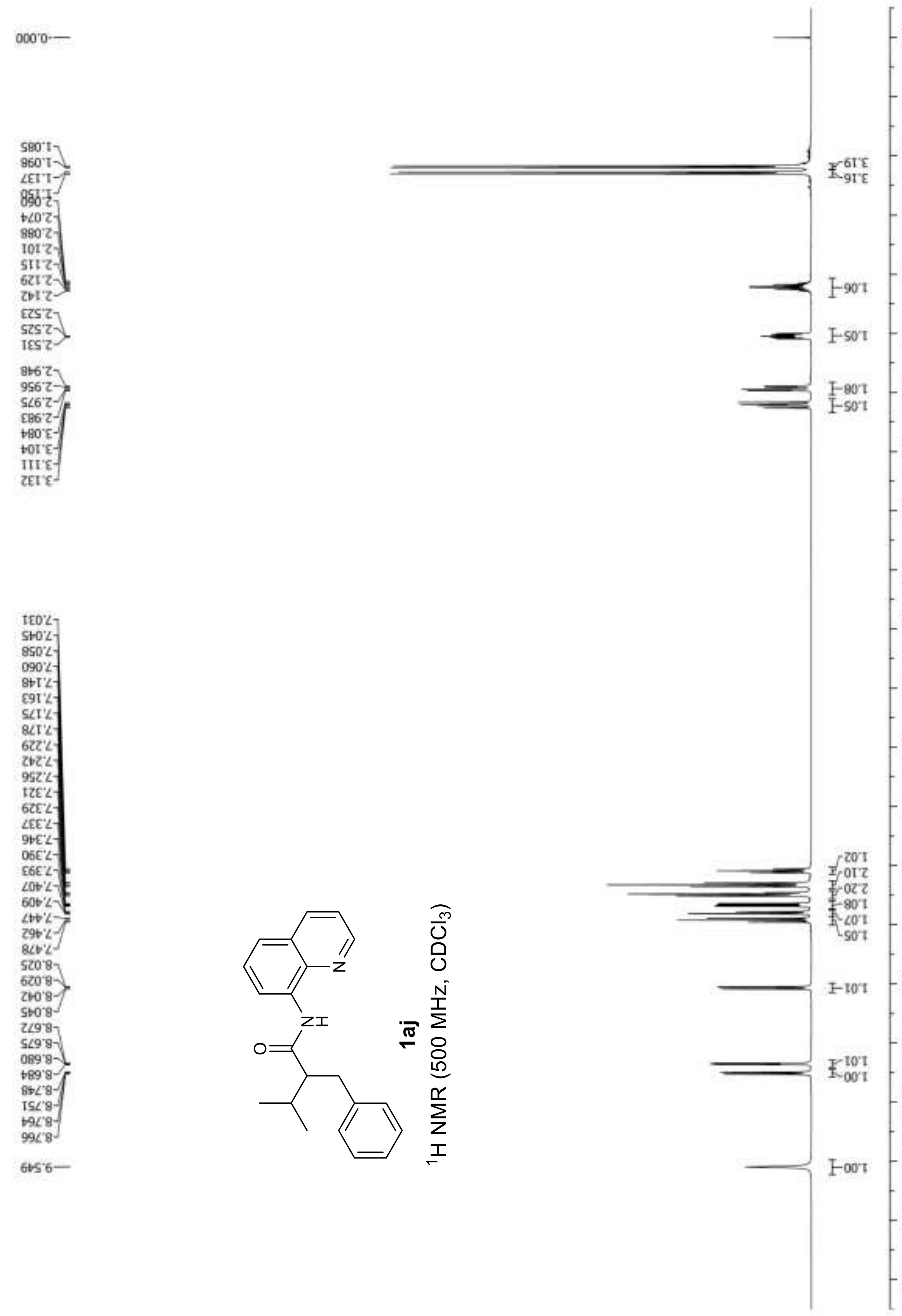

Supplementary Figure 27. ^{1}H NMR (500 MHz, $CDCl_3$) spectrum for 1aj.

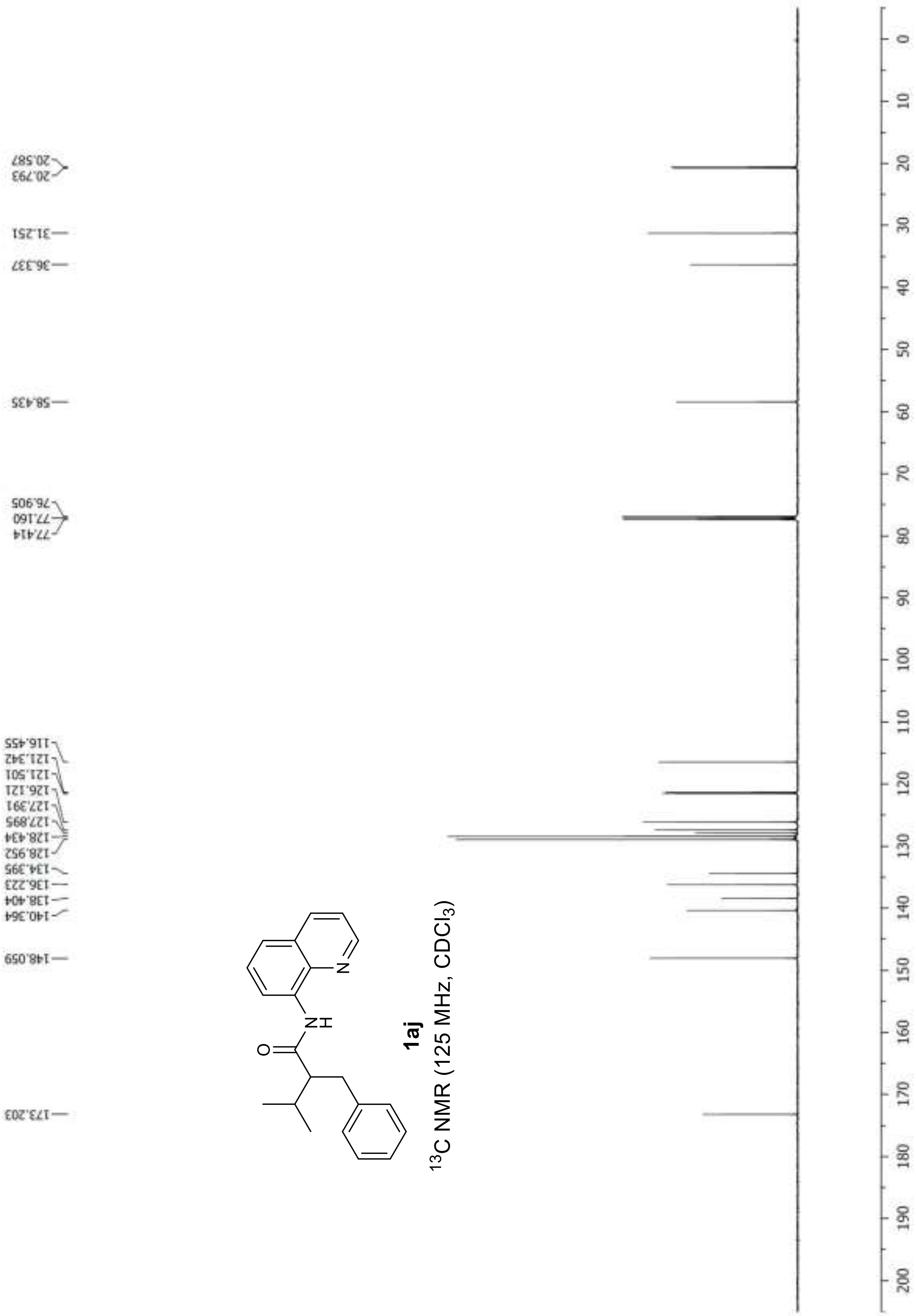

Supplementary Figure 28. ^{13}C NMR (125 MHz, $CDCl_3$) spectrum for 1aj.

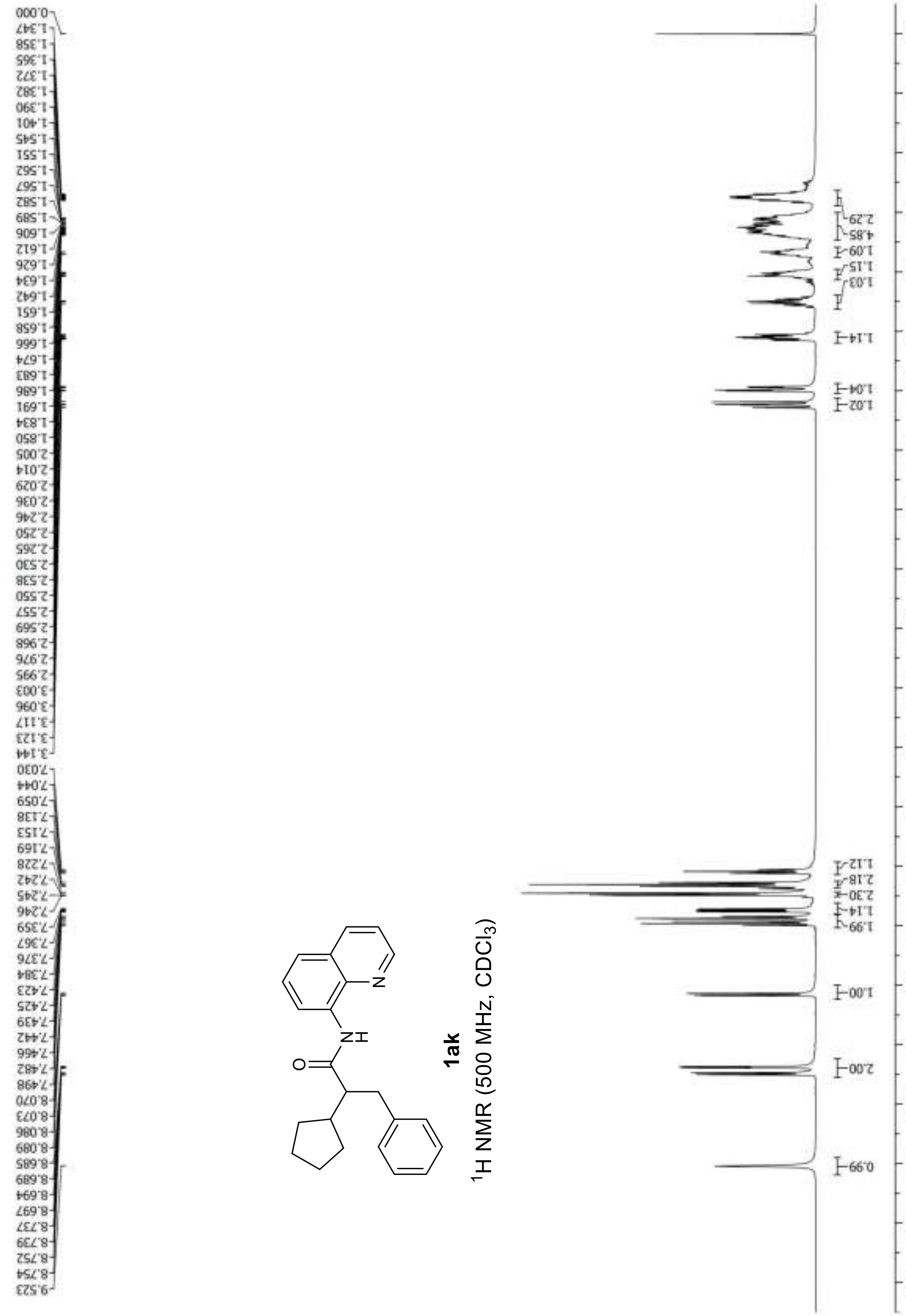

Supplementary Figure 29. ^{1}H NMR (500 MHz, $CDCl_3$) spectrum for 1ak.

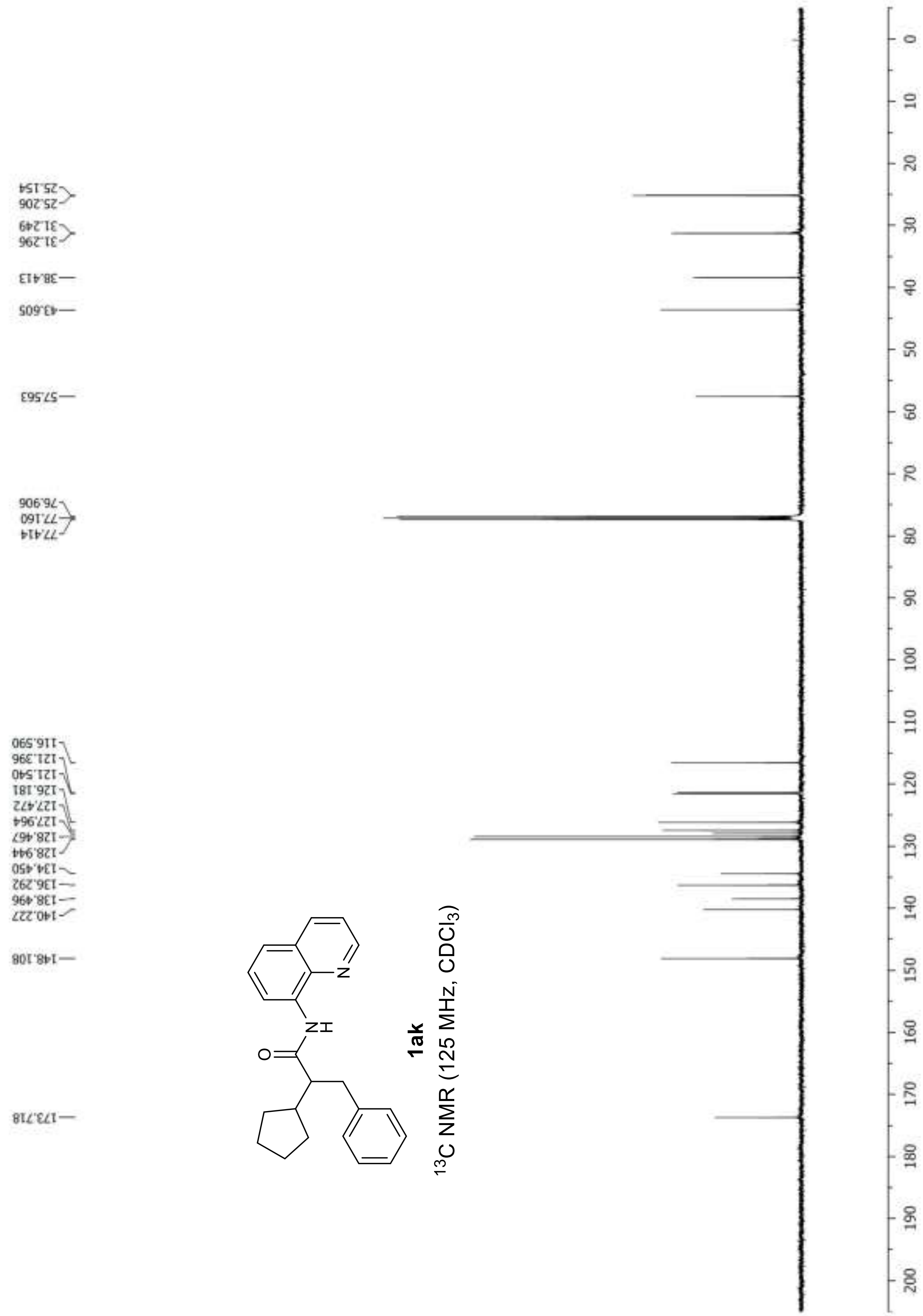

Supplementary Figure 30. ^{13}C NMR (125 MHz, $CDCl_3$) spectrum for 1ak.

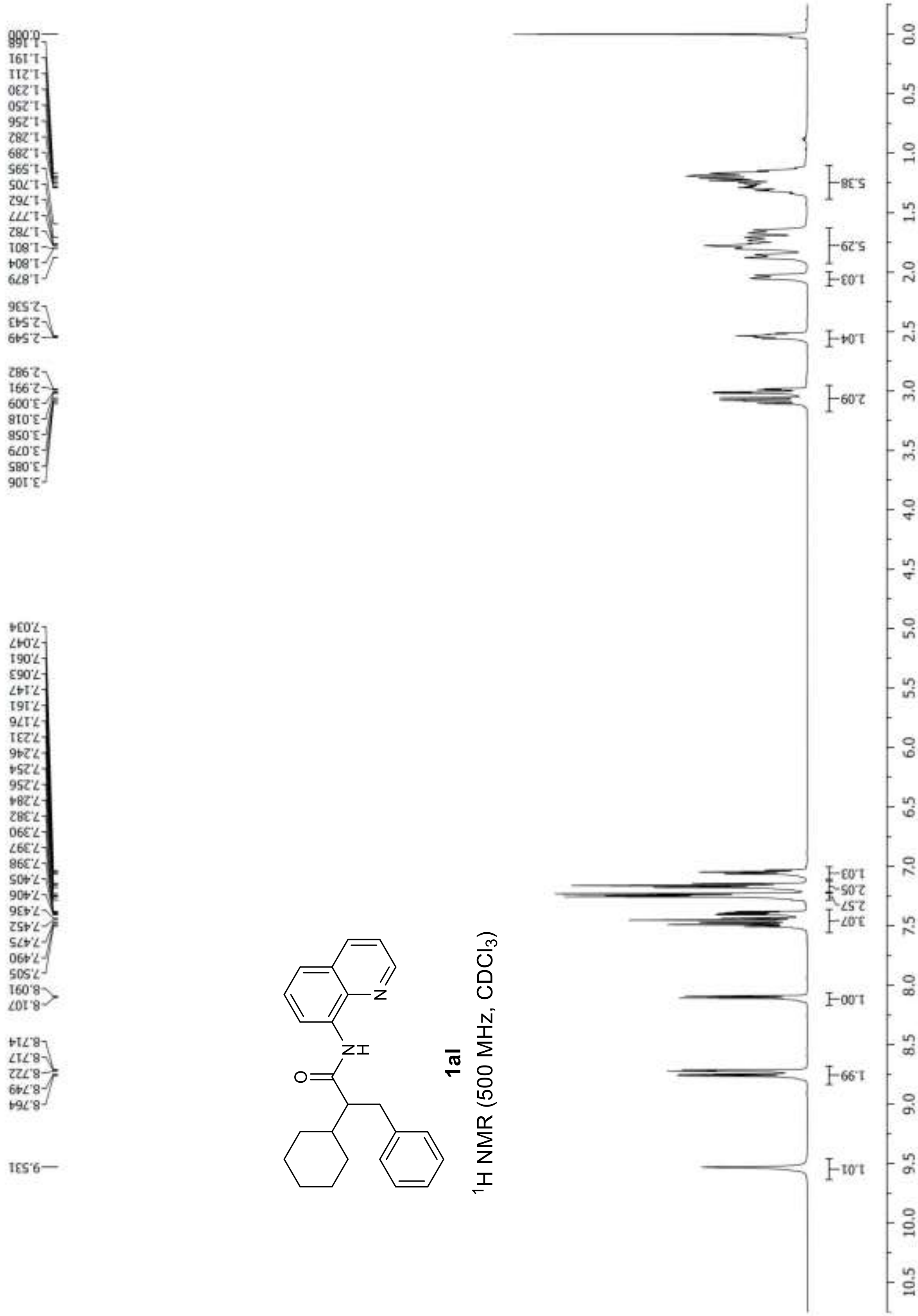

Supplementary Figure 31. ^{1}H NMR (500 MHz, $CDCl_3$) spectrum for 1al.

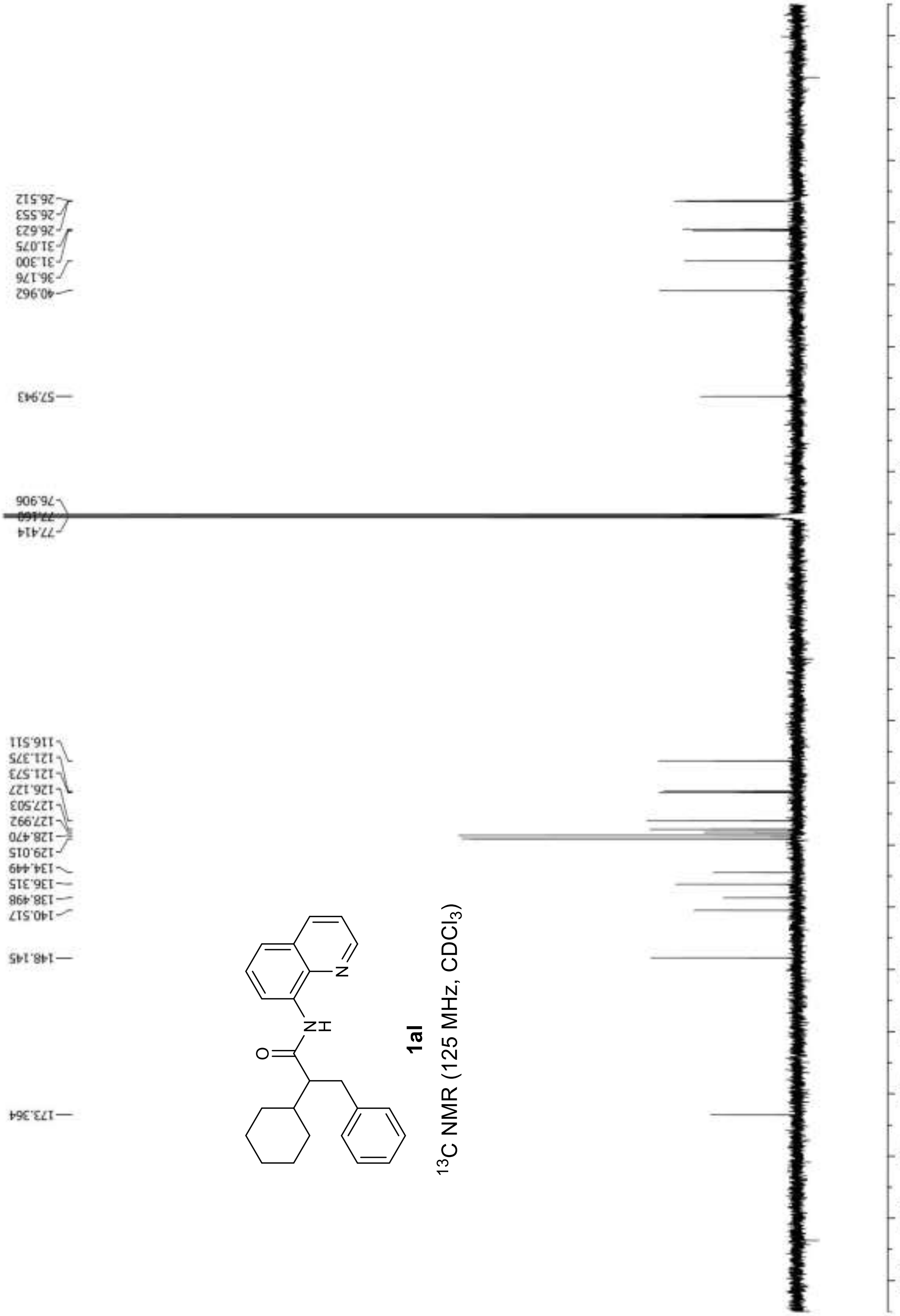

Supplementary Figure 32. ^{13}C NMR (125 MHz, $CDCl_3$) spectrum for 1al.

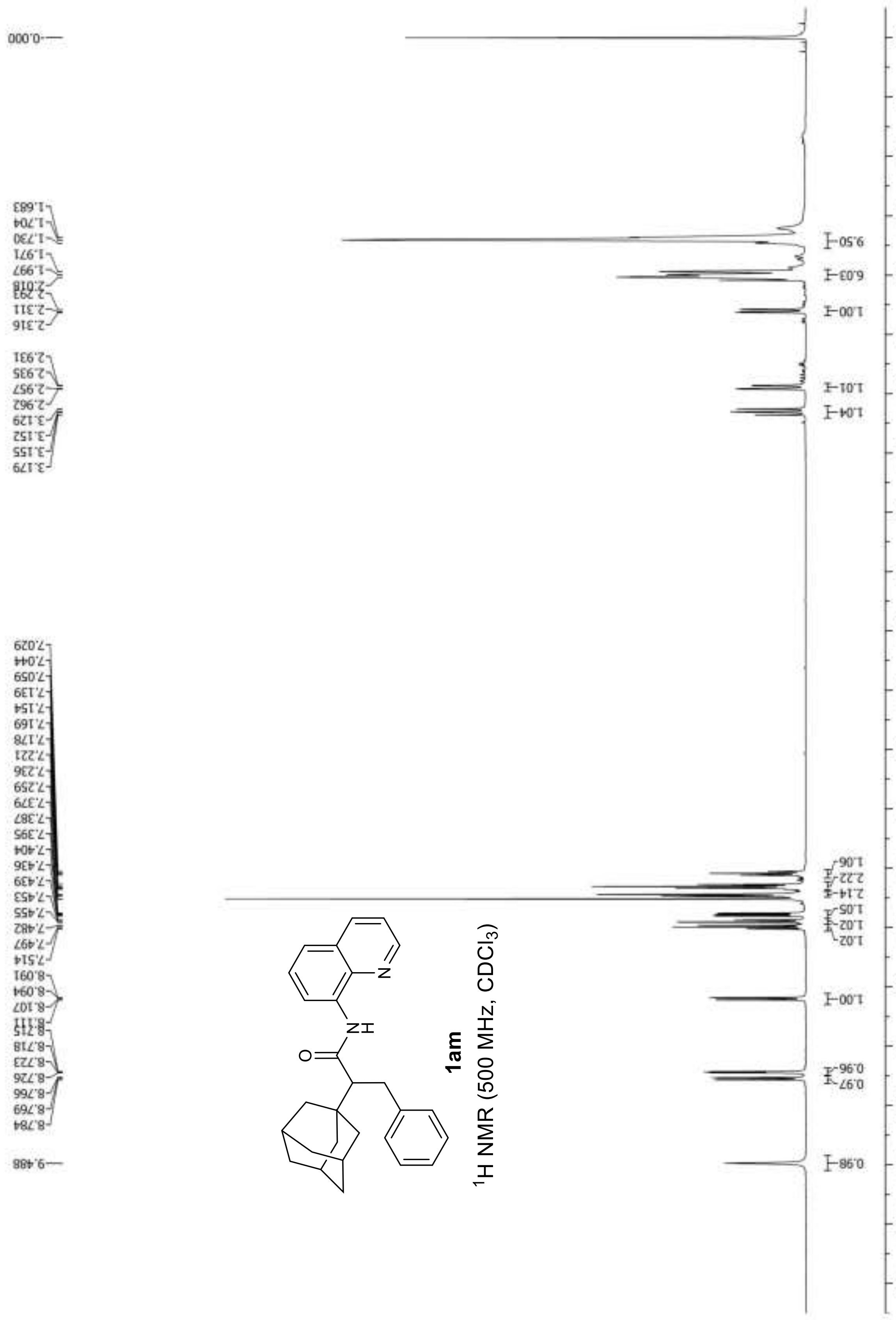

Supplementary Figure 33. ^{1}H NMR (500 MHz, $CDCl_3$) spectrum for 1am.

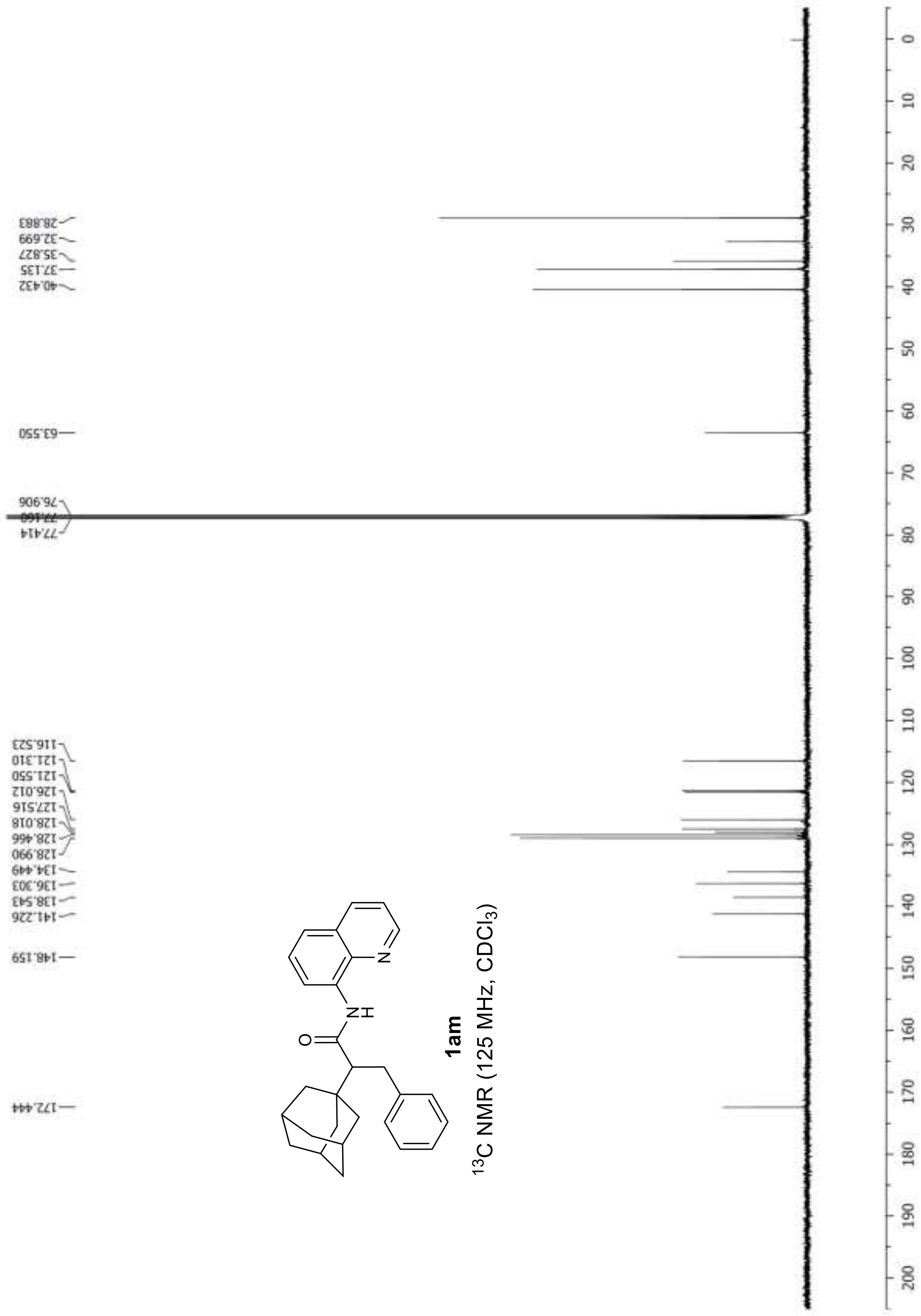

Supplementary Figure 34. ^{13}C NMR (125 MHz, $CDCl_3$) spectrum for 1am.

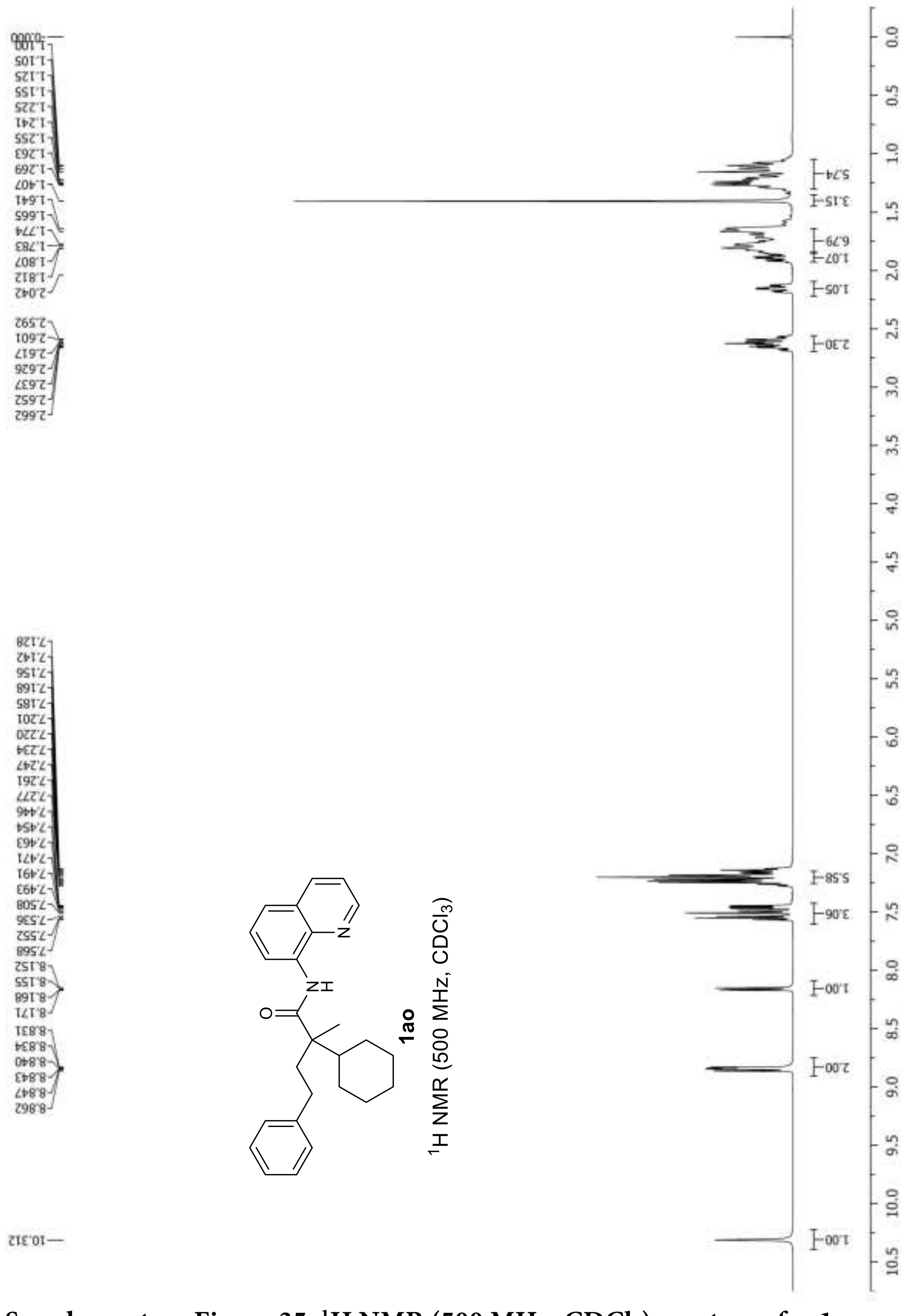

Supplementary Figure 35. ^{1}H NMR (500 MHz, $CDCl_3$) spectrum for 1ao.

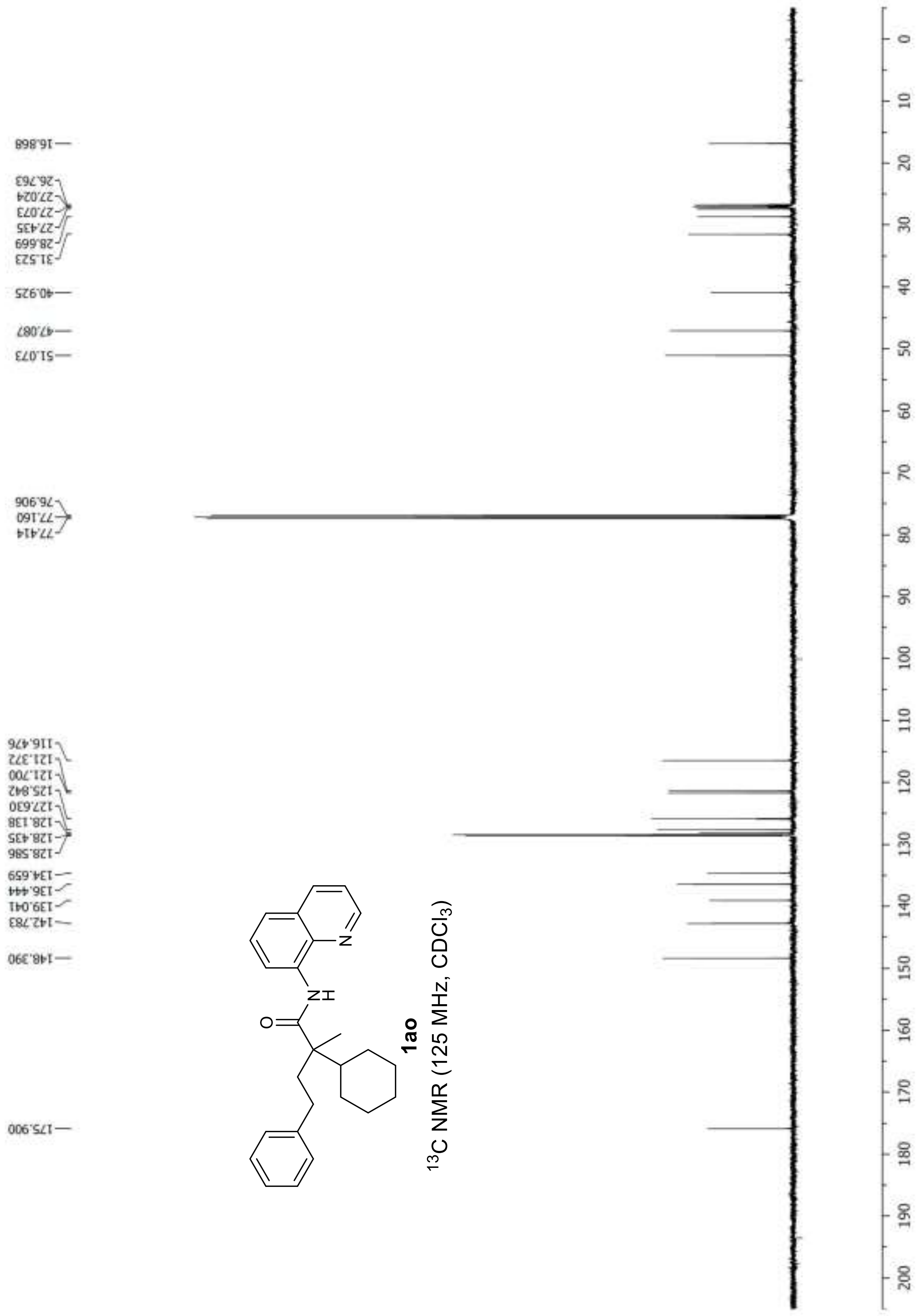

Supplementary Figure 36. ^{13}C NMR (125 MHz, $CDCl_3$) spectrum for 1ao.

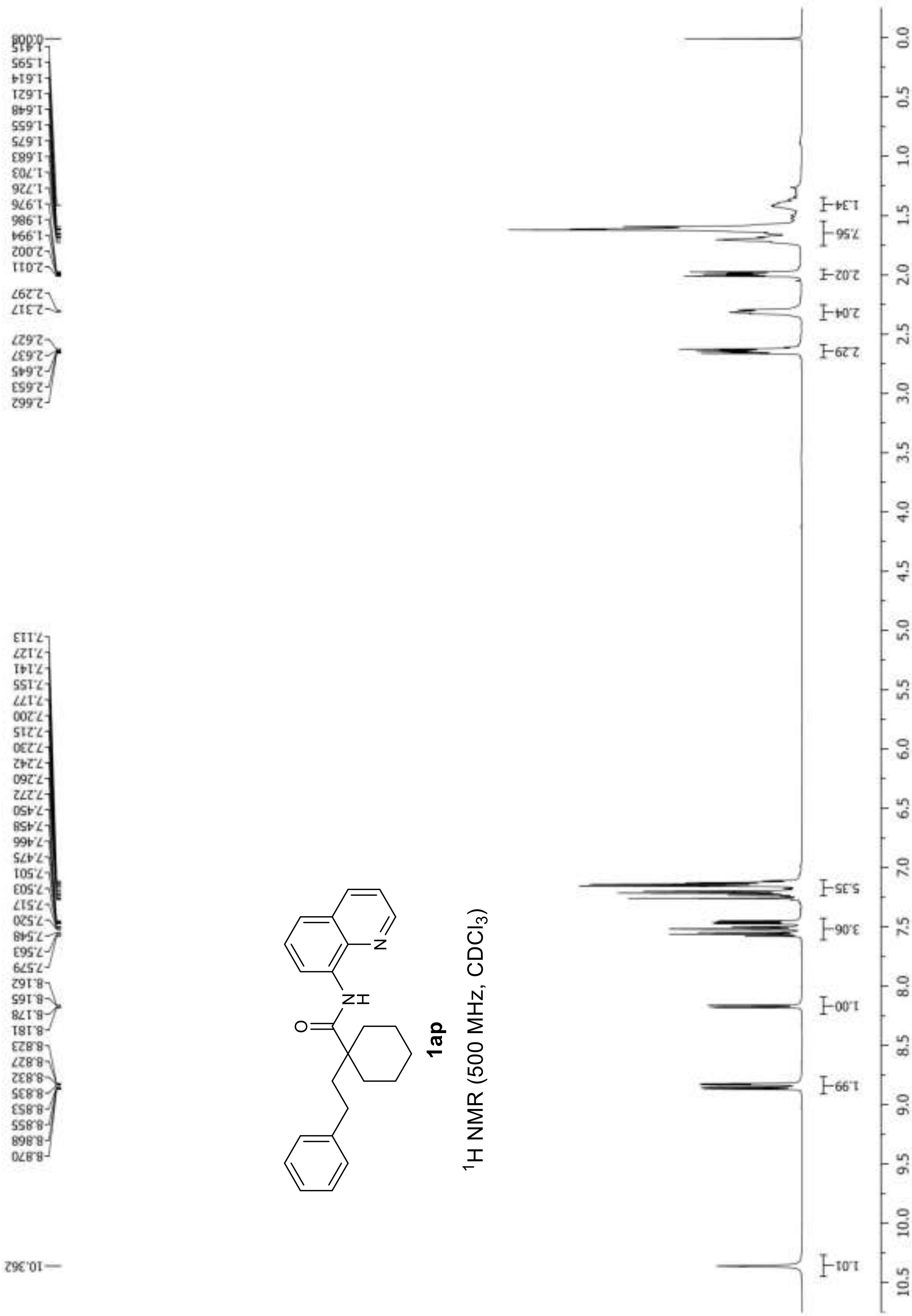

Supplementary Figure 37. ^{1}H NMR (500 MHz, CDCl$_3$) spectrum for 1ap.

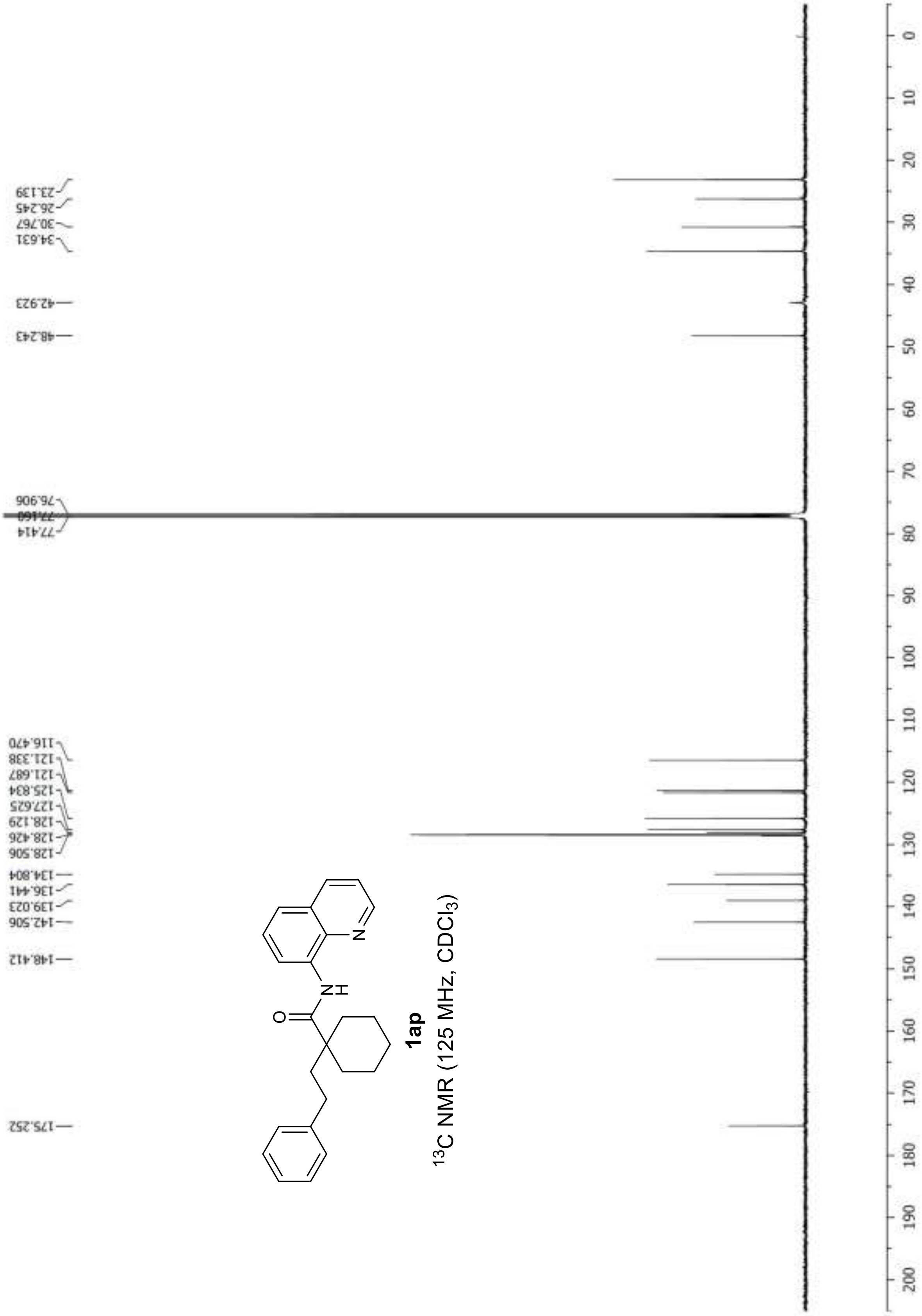

Supplementary Figure 38. ^{13}C NMR (125 MHz, $CDCl_3$) spectrum for 1ap.

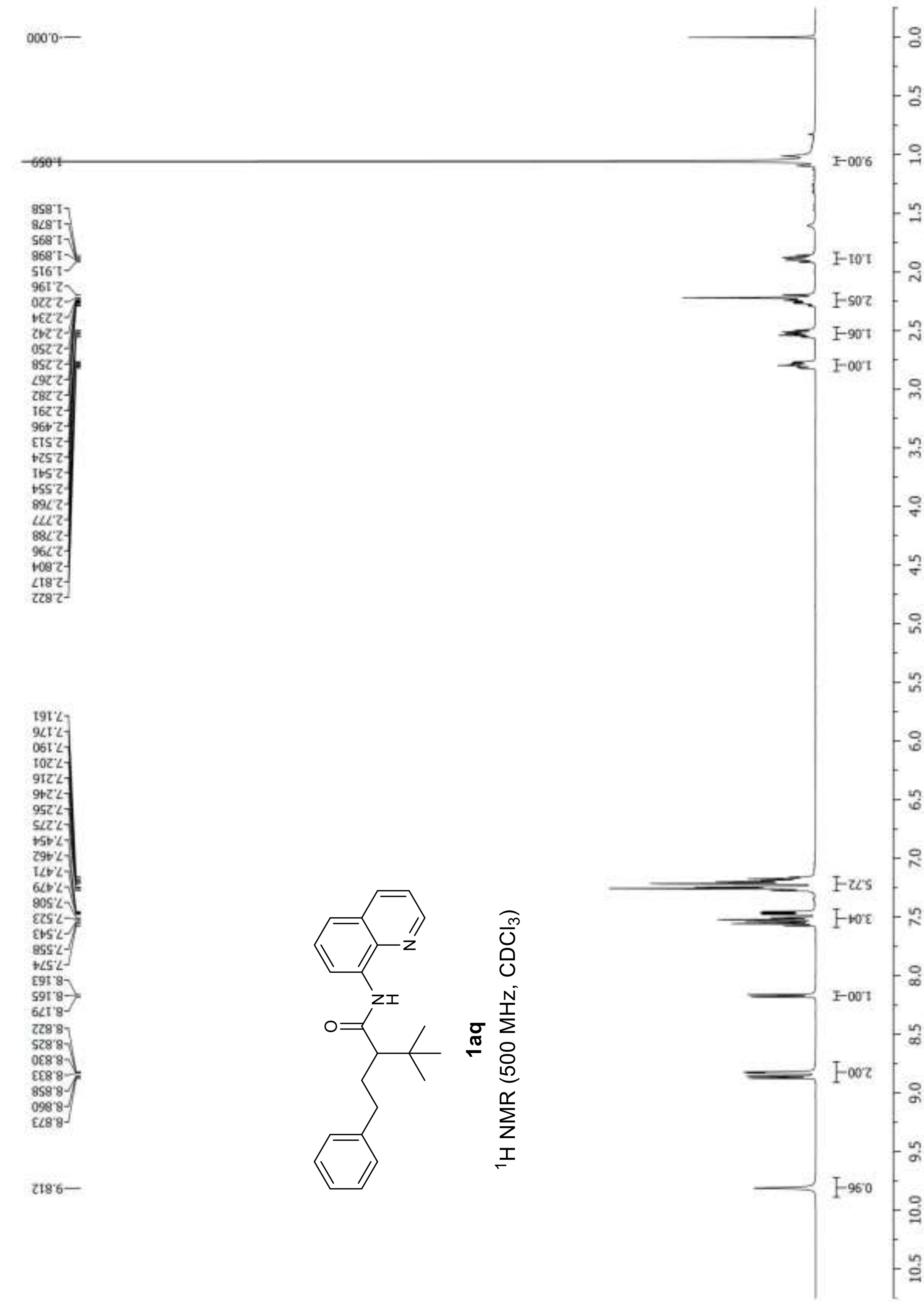

Supplementary Figure 39. **^{1}H NMR (500 MHz, $CDCl_3$) spectrum for 1aq.**

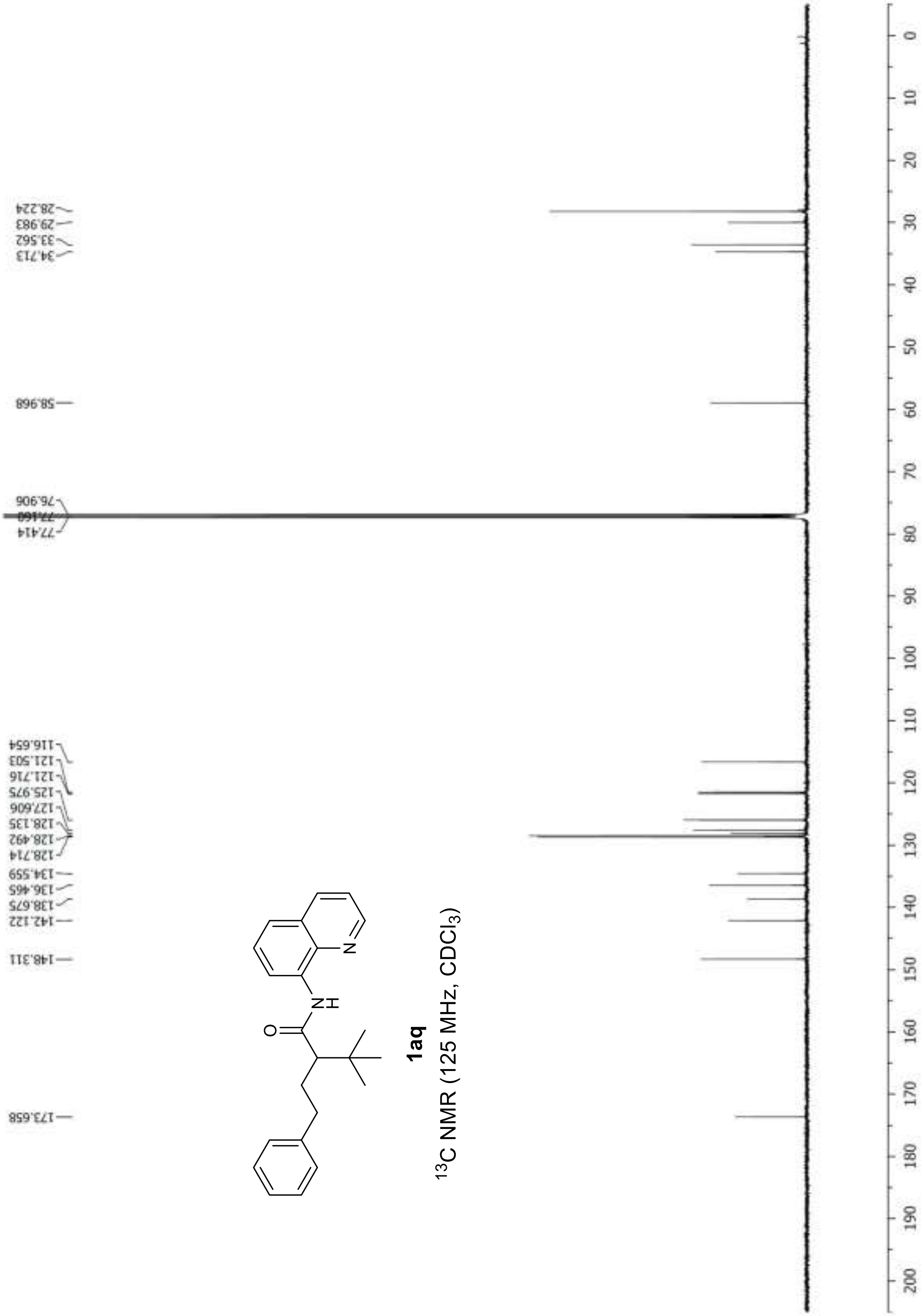

Supplementary Figure 40. ^{13}C NMR (125 MHz, $CDCl_3$) spectrum for 1aq.

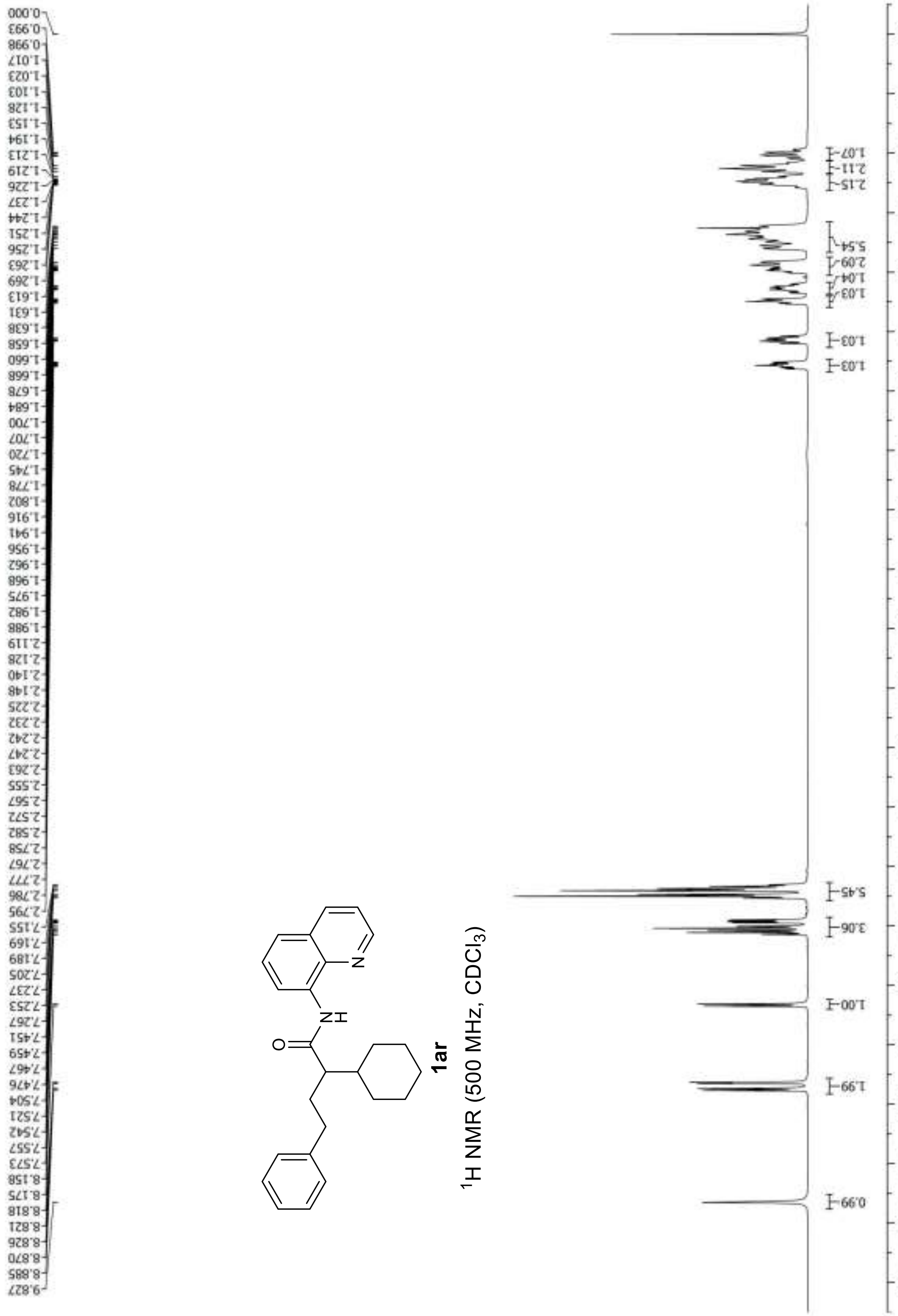

Supplementary Figure 41. ^{1}**H NMR (500 MHz,** $CDCl_3$**) spectrum for 1ar.**

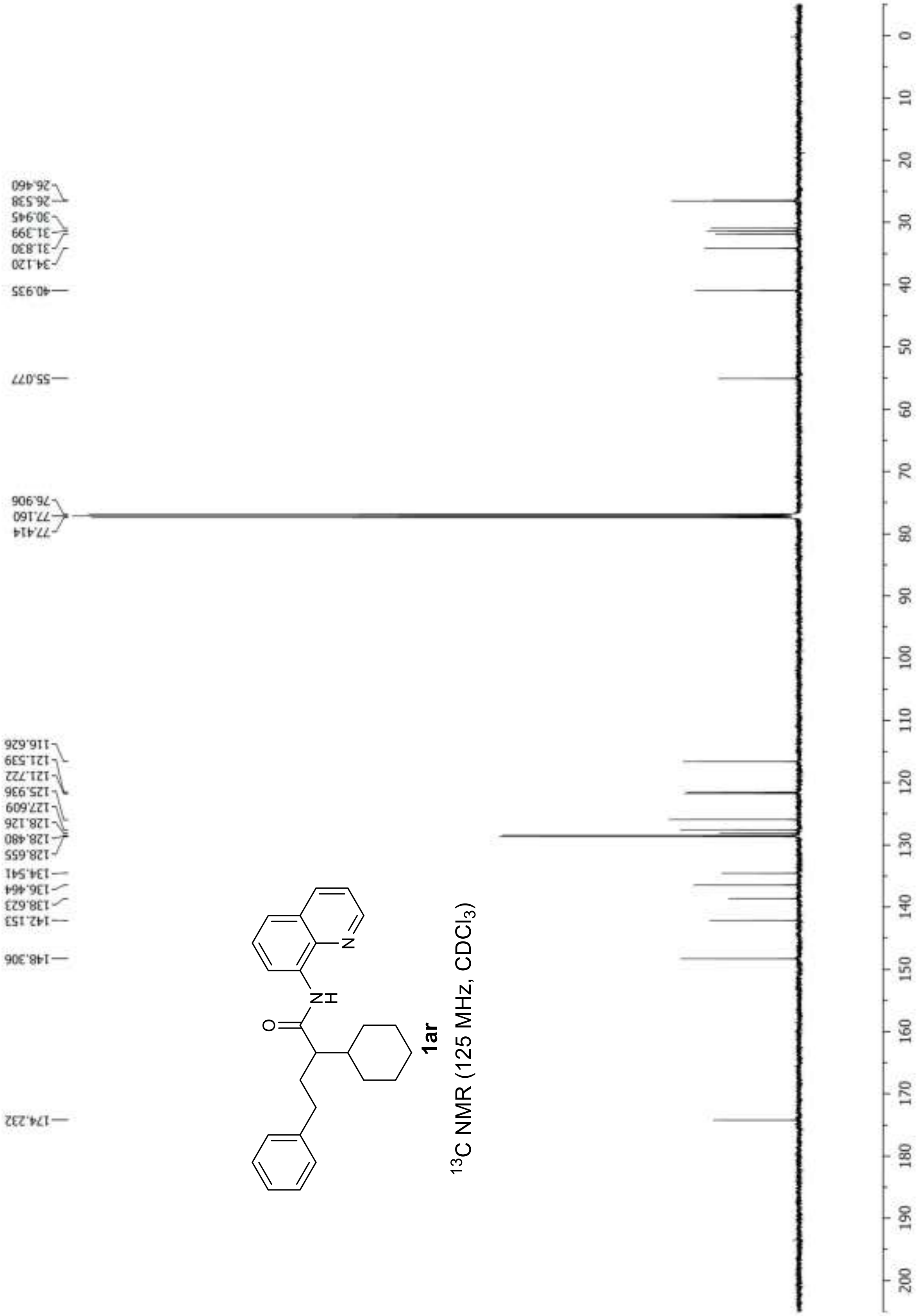

Supplementary Figure 42. ^{13}C NMR (125 MHz, $CDCl_3$) spectrum for 1ar.

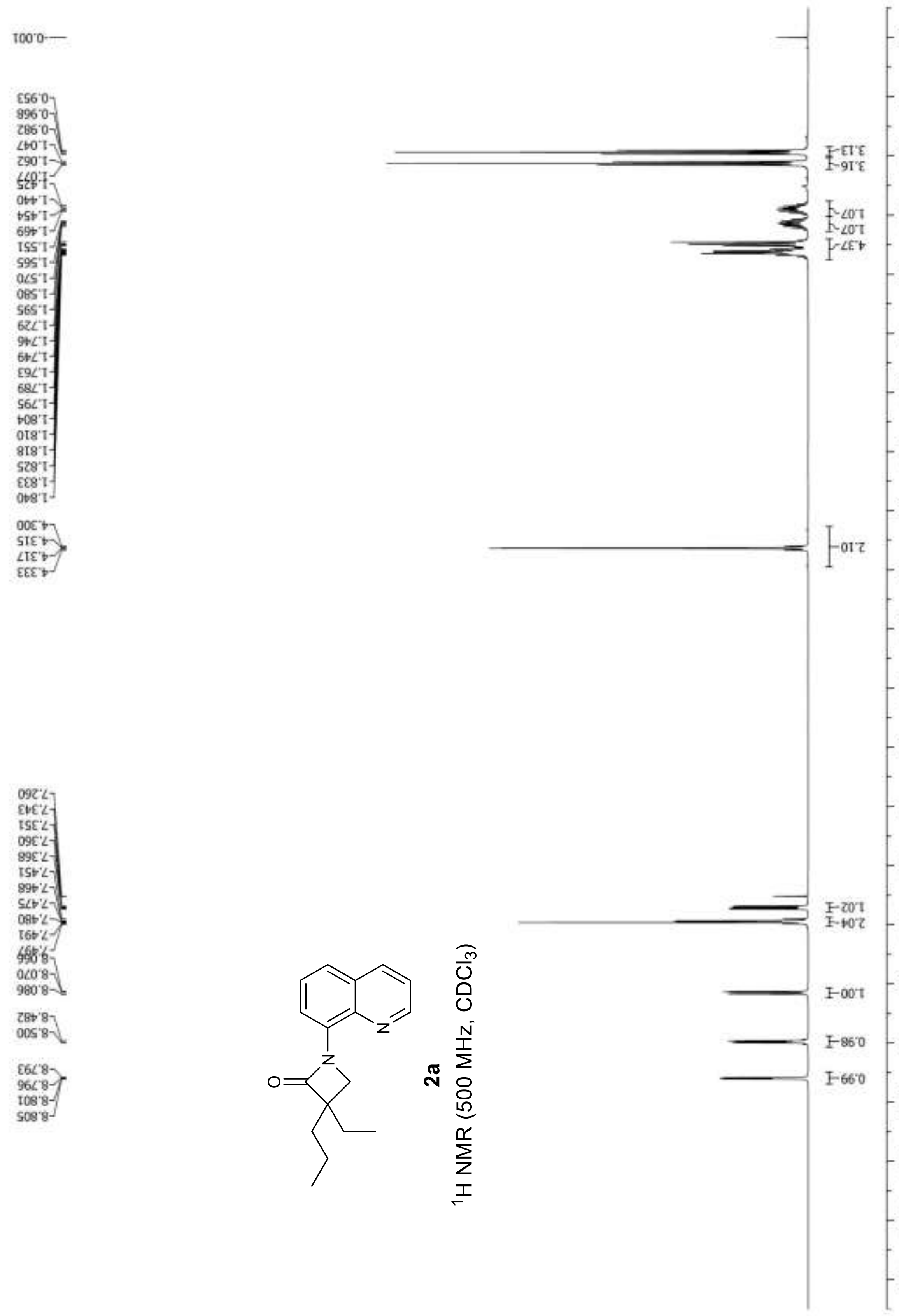

Supplementary Figure 43. ^{1}H NMR (500 MHz, $CDCl_3$) spectrum for 2a.

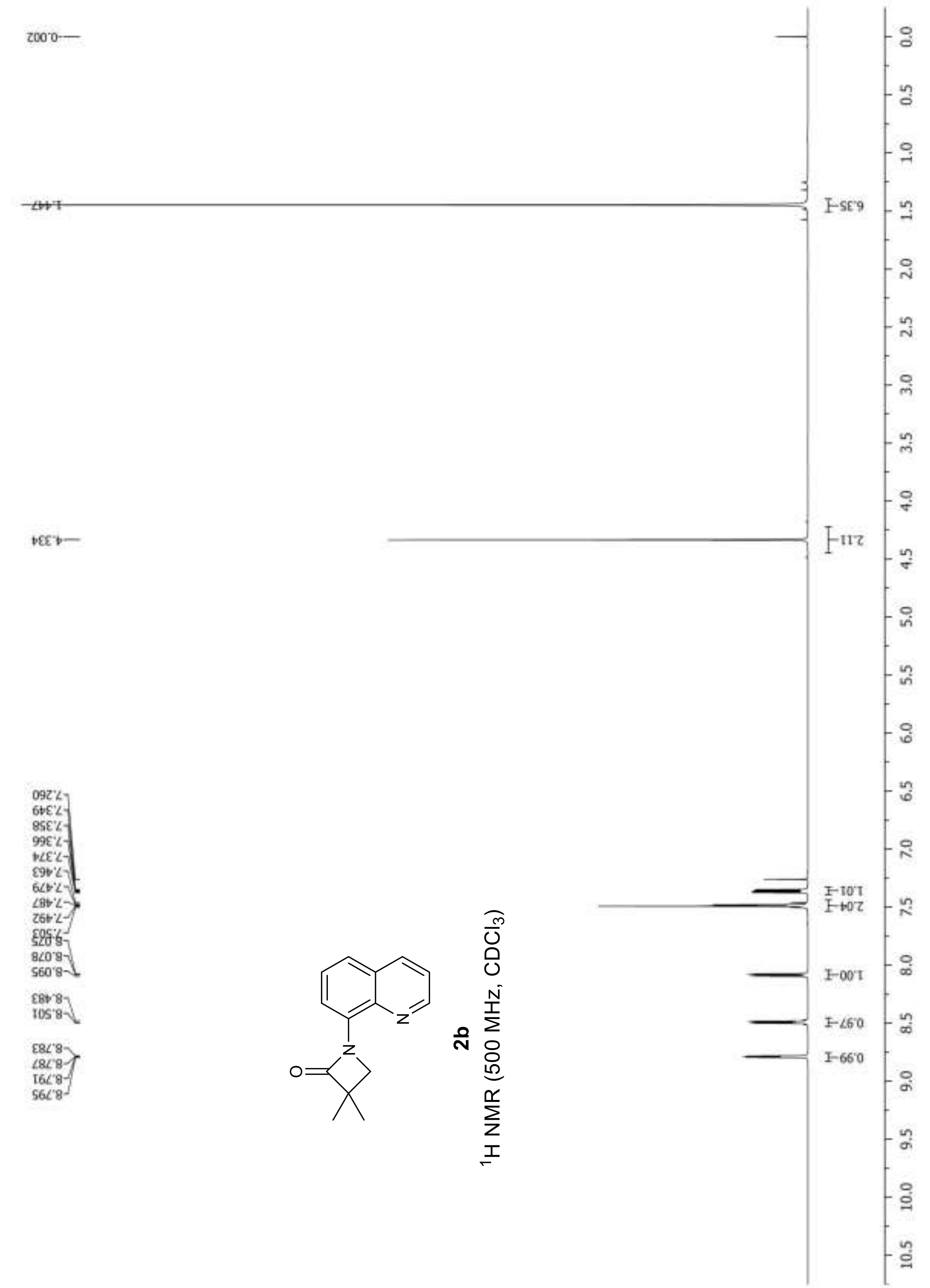

Supplementary Figure 44. ^{1}H NMR (500 MHz, $CDCl_3$) spectrum for 2b.

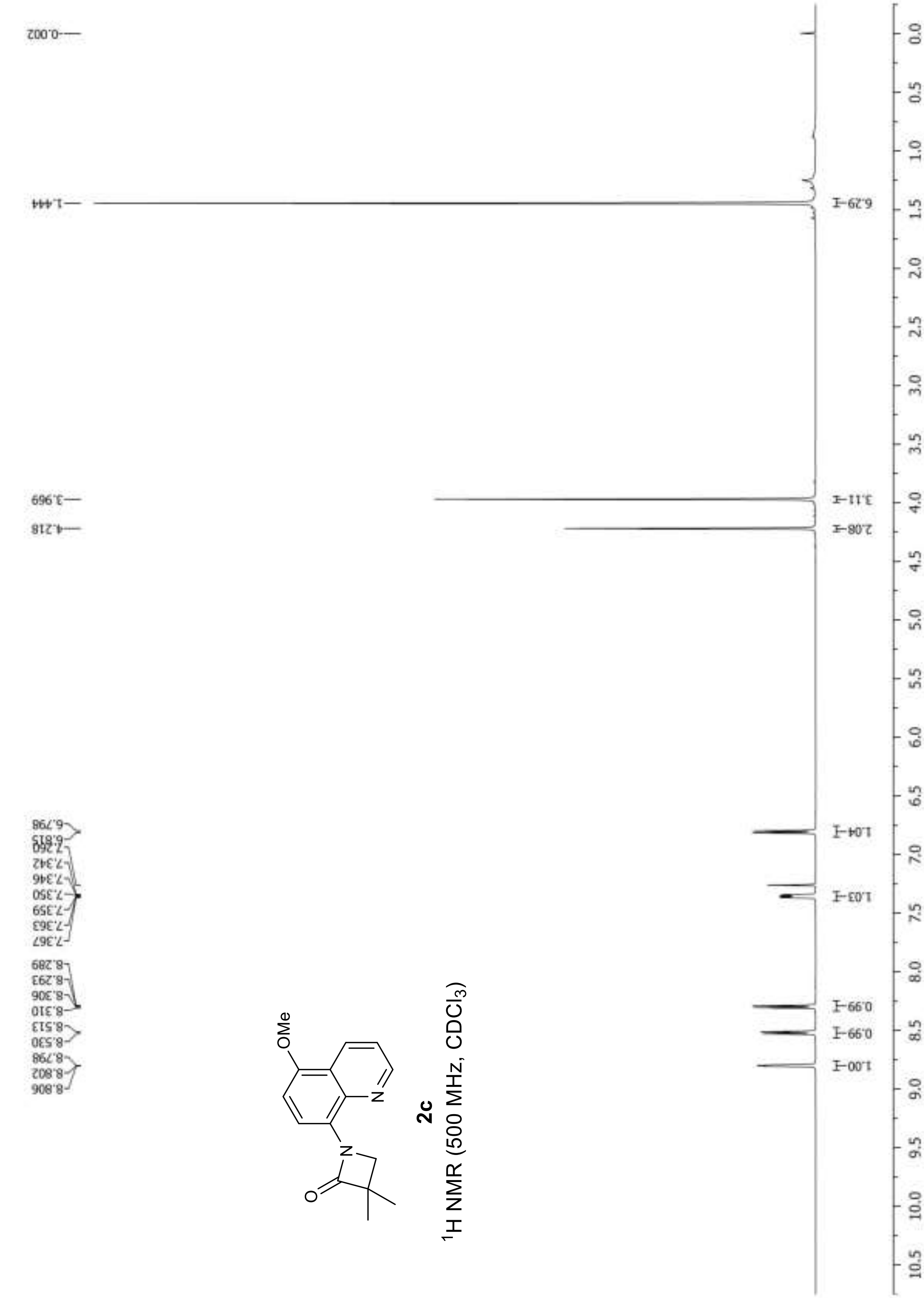

Supplementary Figure 45. ^{1}H NMR (500 MHz, $CDCl_3$) spectrum for 2c.

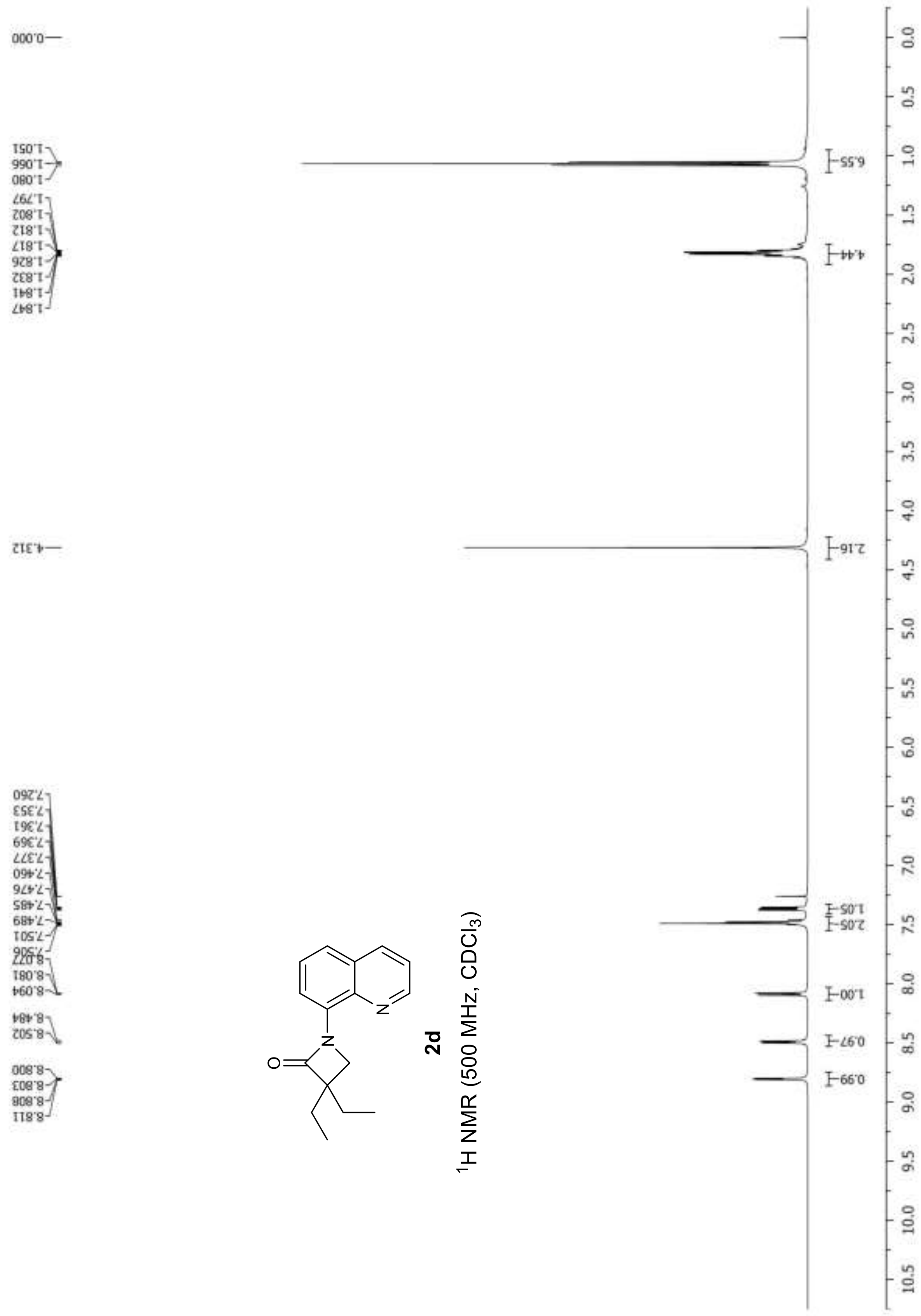

Supplementary Figure 46. ^{1}H NMR (500 MHz, $CDCl_3$) spectrum for 2d.

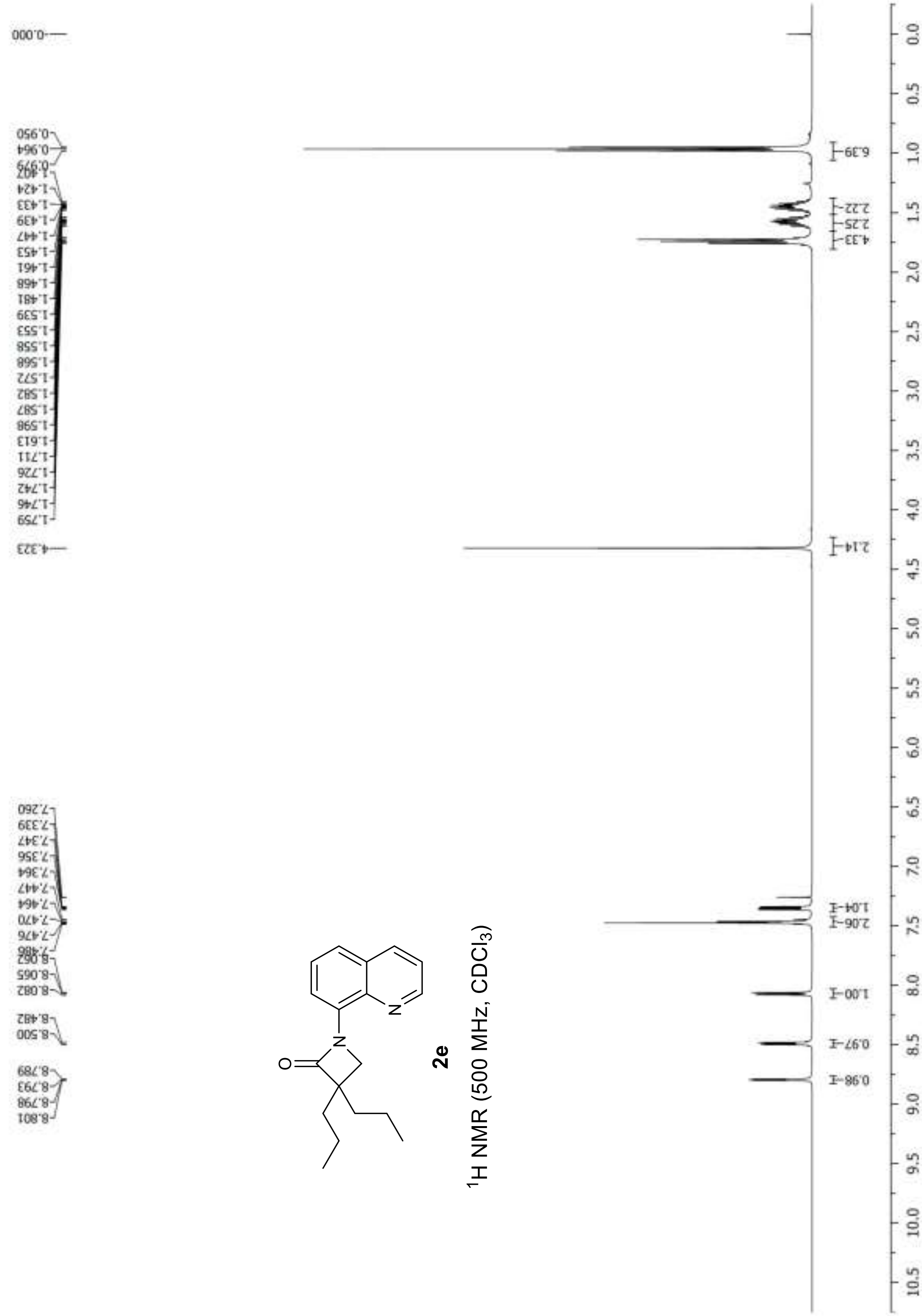

Supplementary Figure 47. ^{1}H NMR (500 MHz, $CDCl_3$) spectrum for 2e.

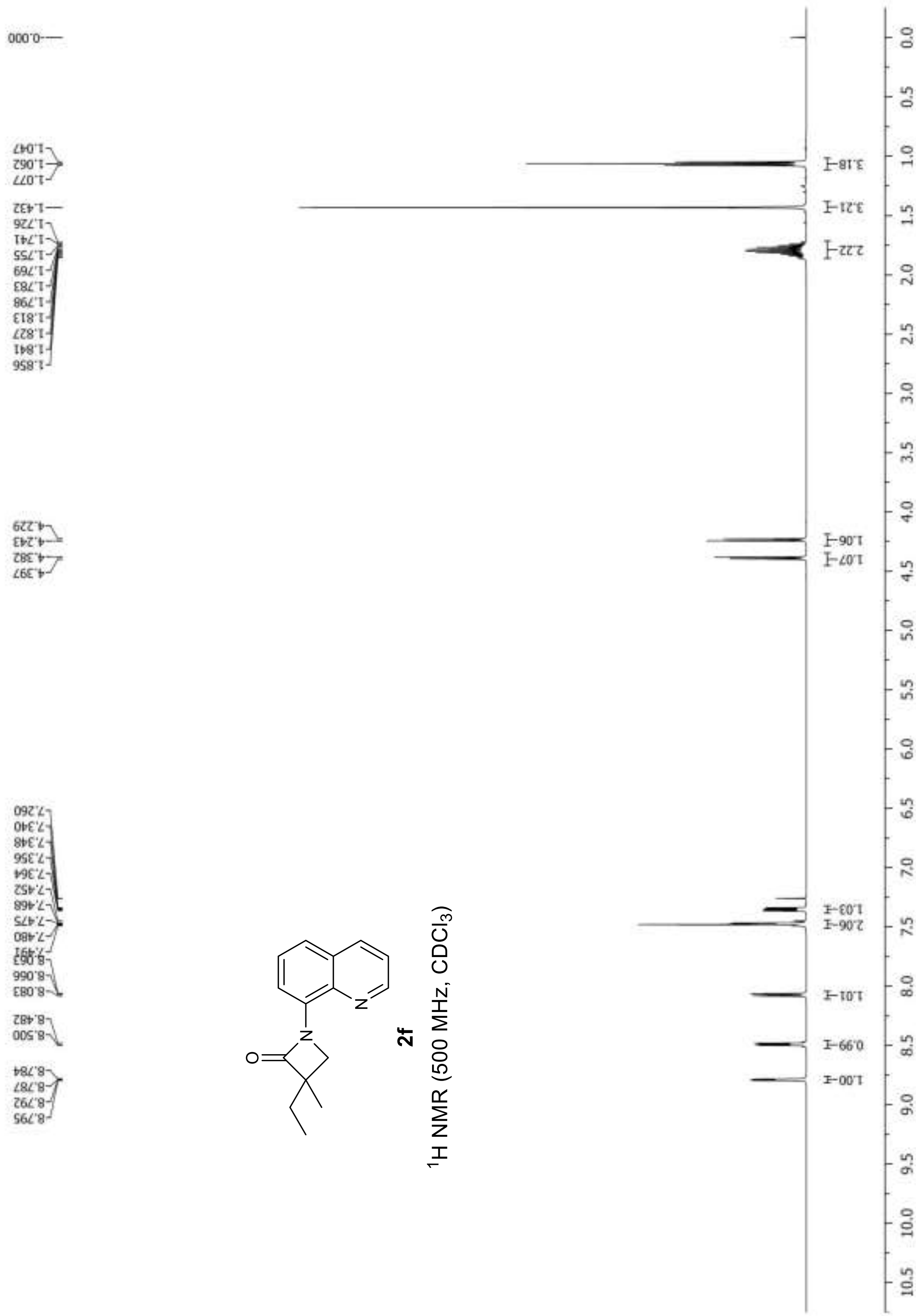

Supplementary Figure 48. ^{1}H NMR (500 MHz, $CDCl_3$) spectrum for 2f.

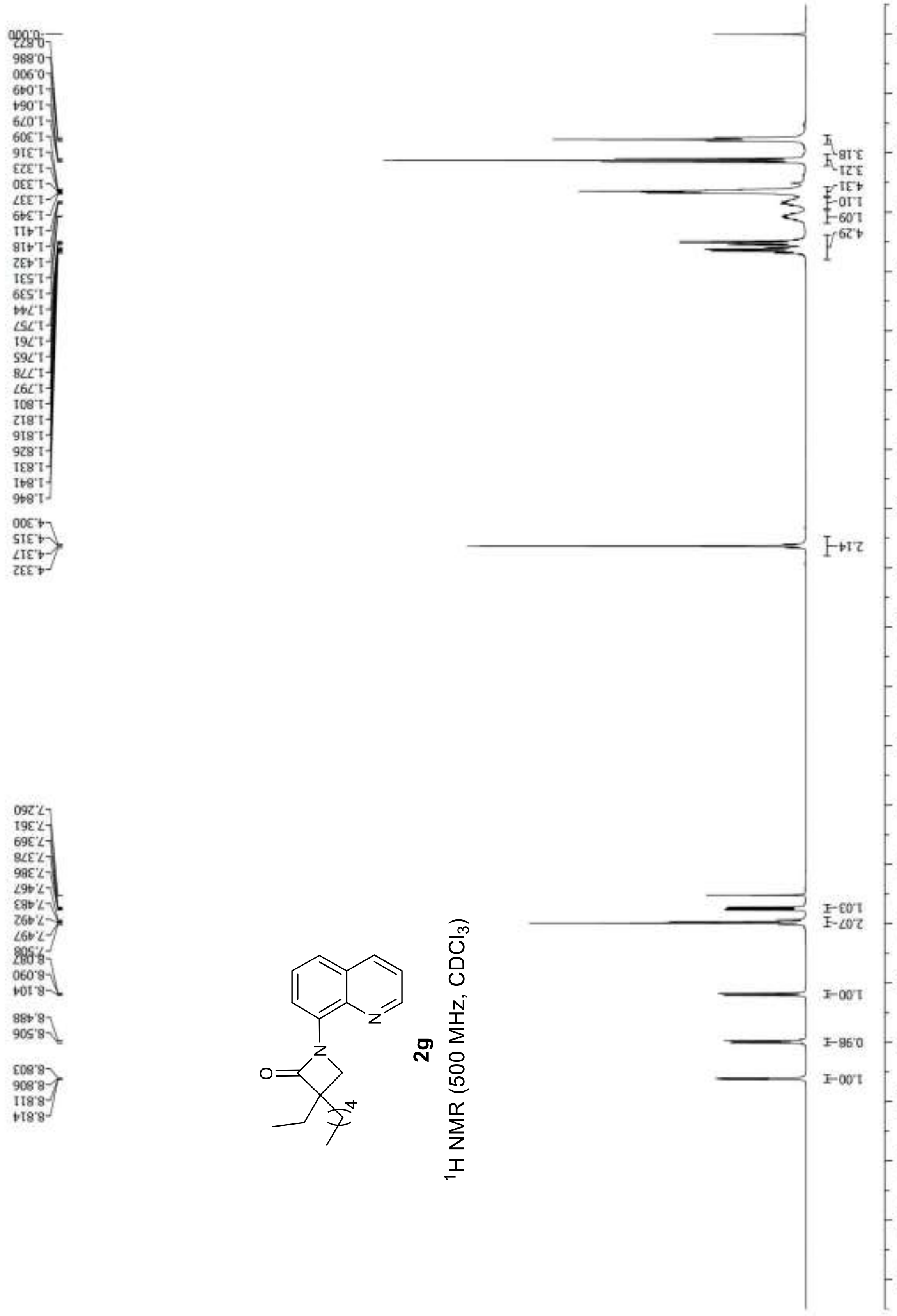

Supplementary Figure 49. ^{1}H NMR (500 MHz, $CDCl_3$) spectrum for 2g.

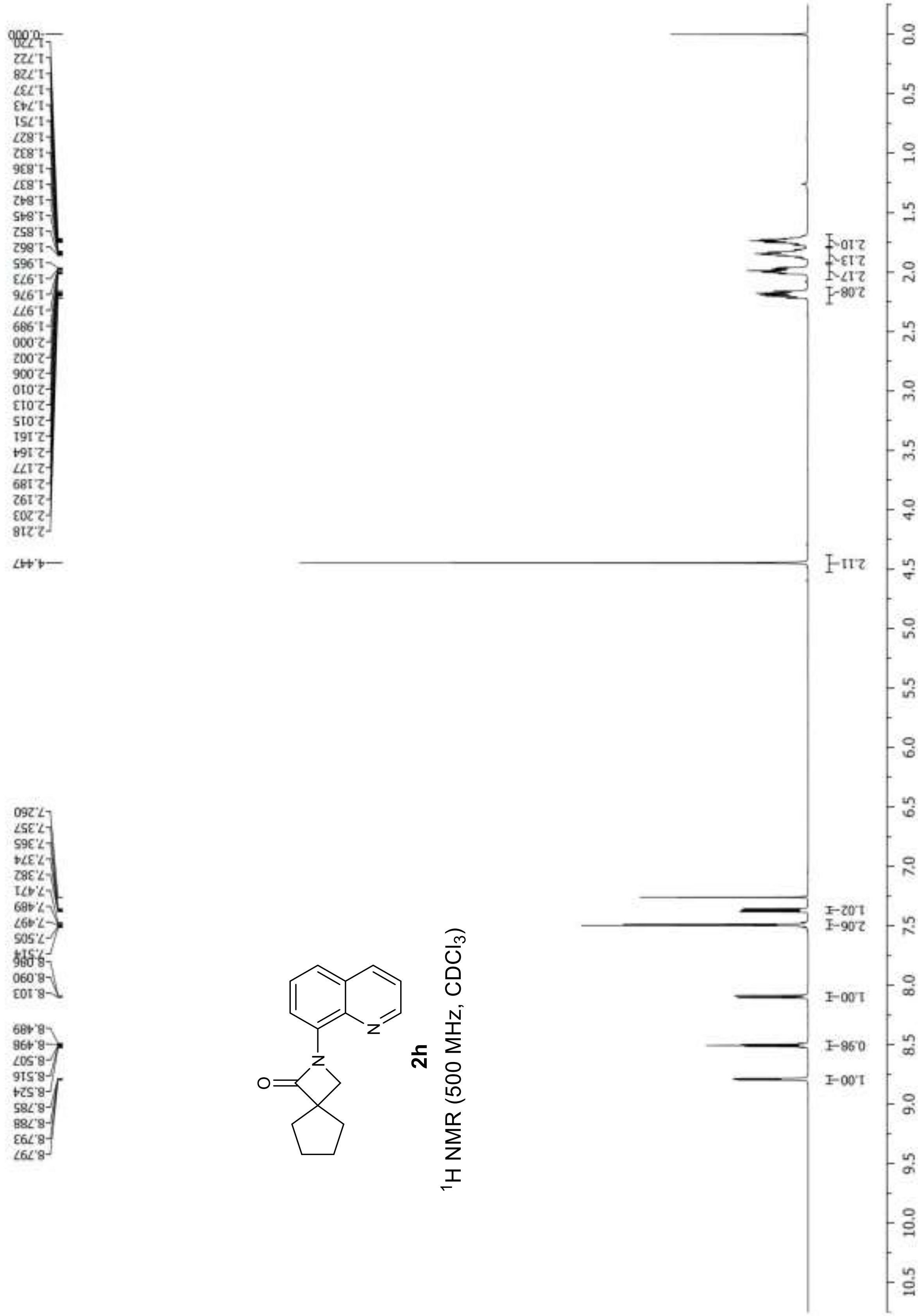

Supplementary Figure 50. ^{1}H NMR (500 MHz, $CDCl_3$) spectrum for 2h.

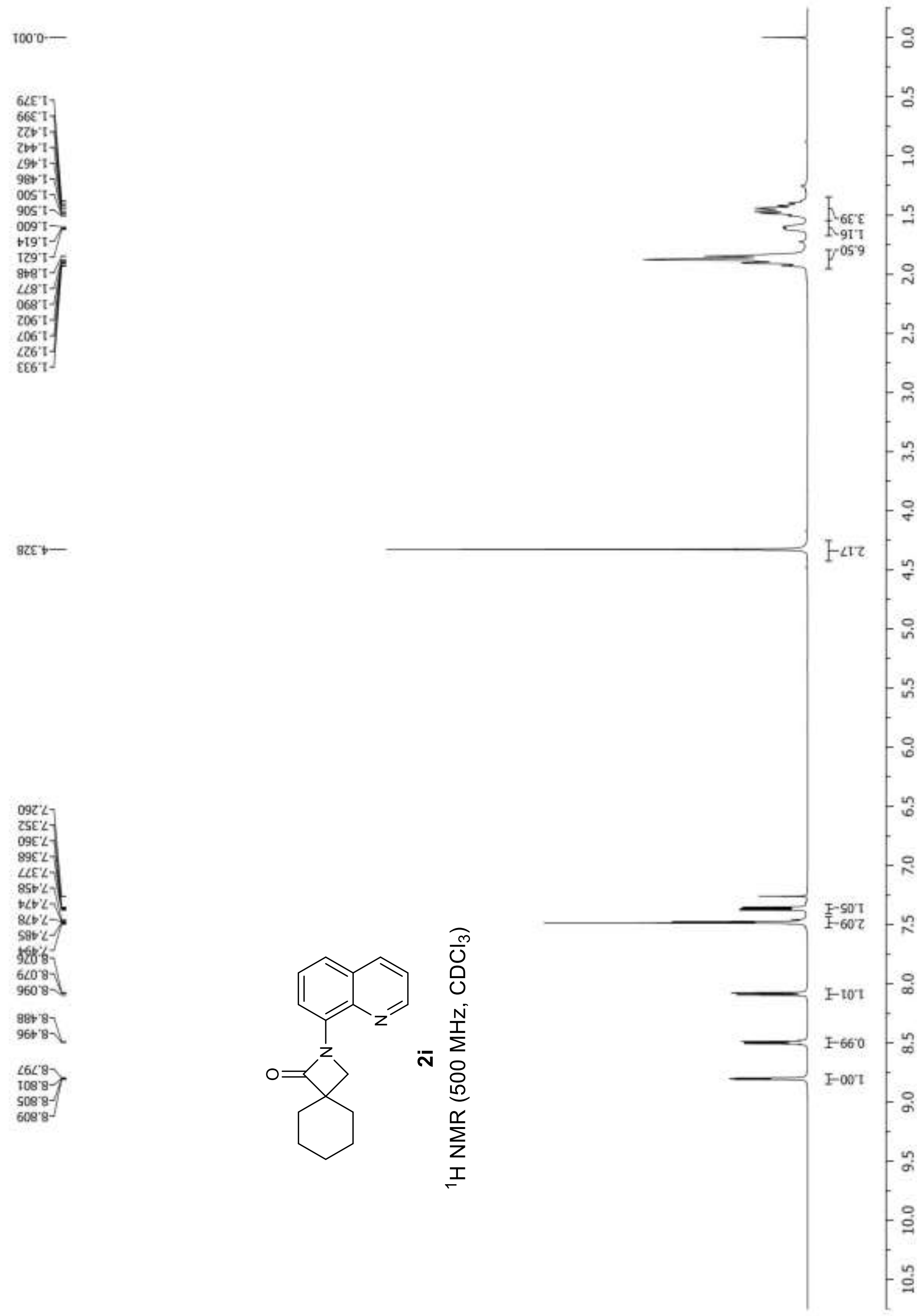

Supplementary Figure 51. ^{1}H NMR (500 MHz, $CDCl_3$) spectrum for 2i.

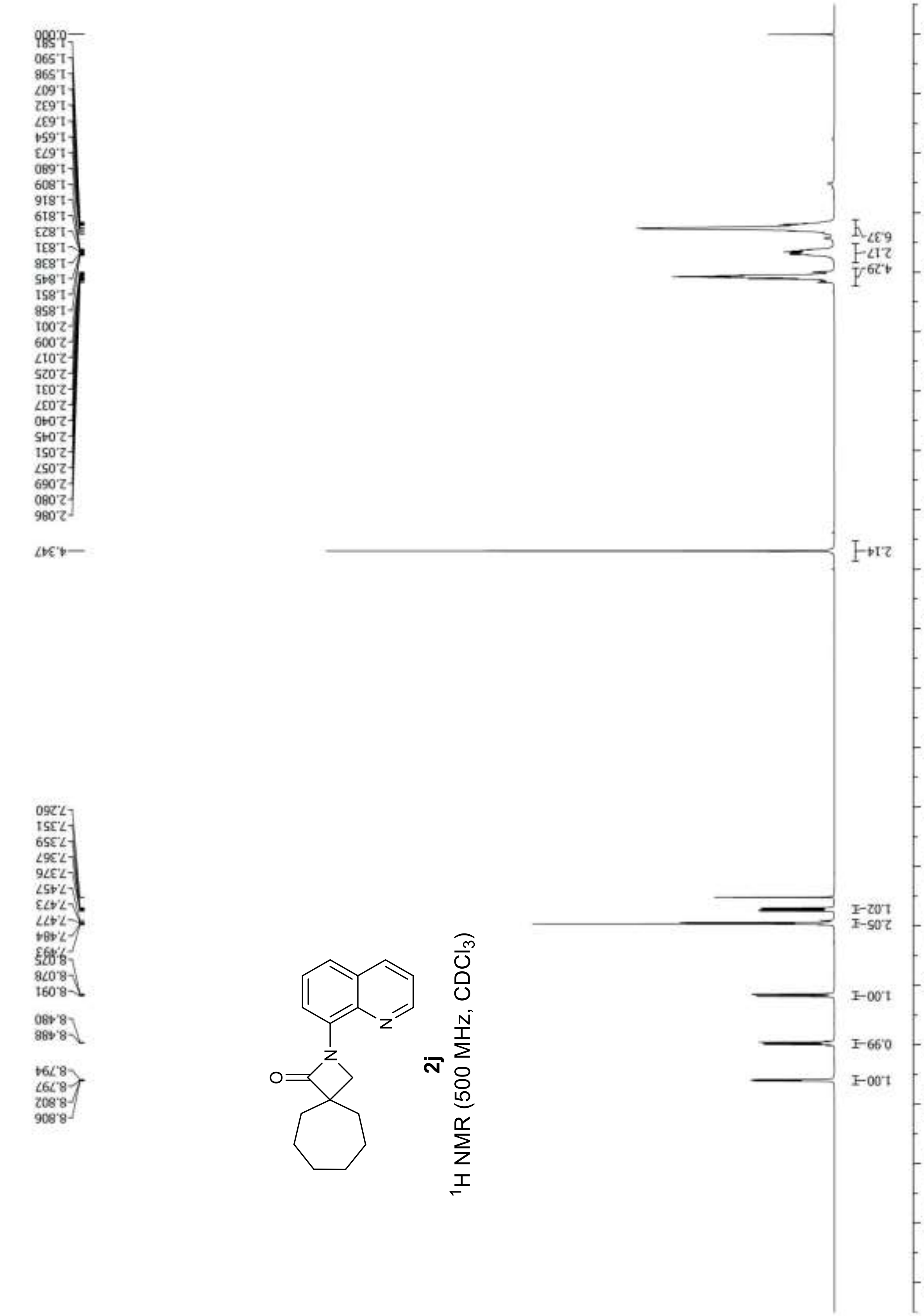

Supplementary Figure 52. ^{1}H NMR (500 MHz, $CDCl_3$) spectrum for 2j.

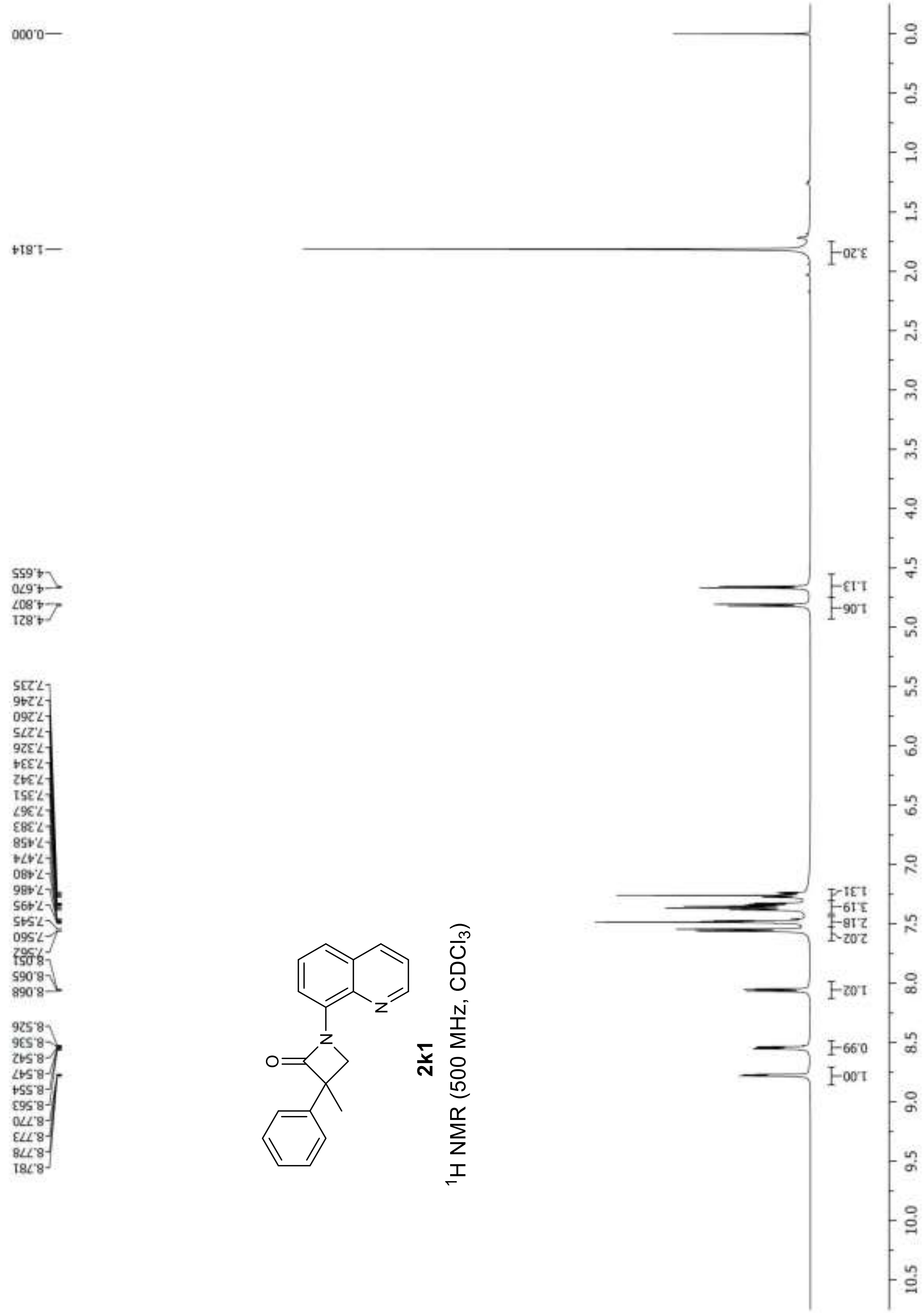

Supplementary Figure 53. ^{1}H NMR (500 MHz, $CDCl_3$) spectrum for 2k1.

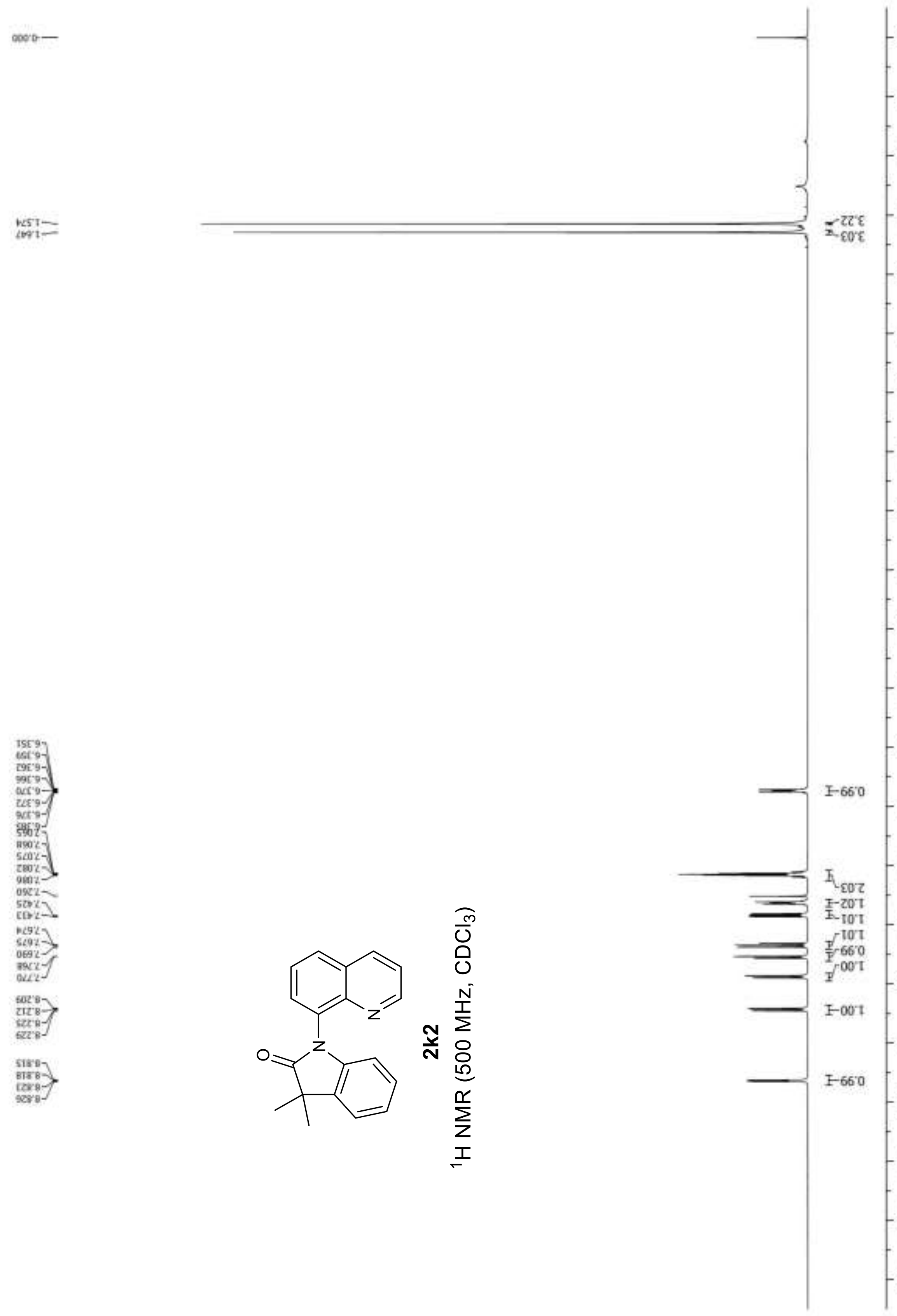

Supplementary Figure 54. ^{1}H NMR (500 MHz, $CDCl_3$) spectrum for 2k2.

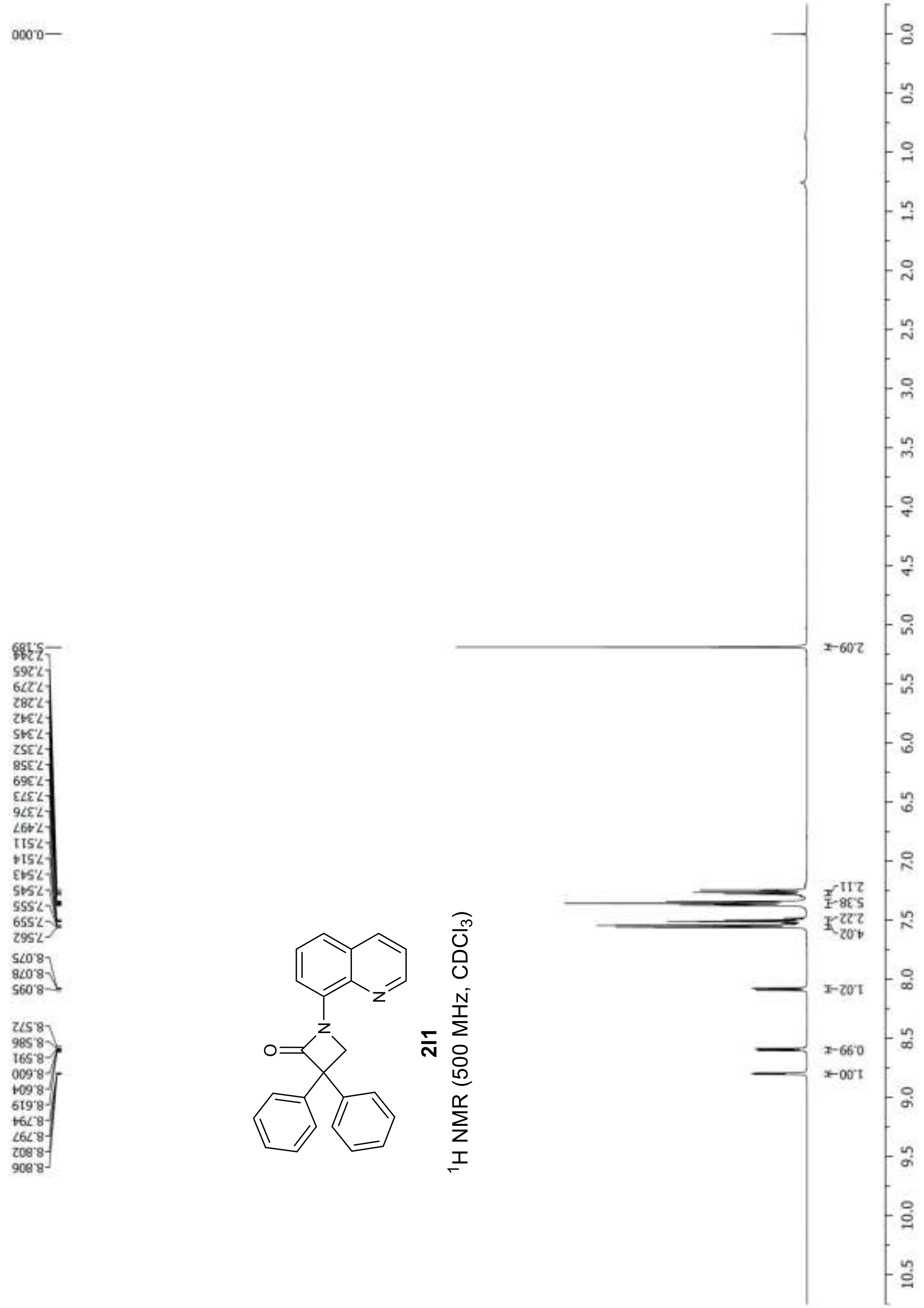

Supplementary Figure 55. ^{1}H NMR (500 MHz, $CDCl_3$) spectrum for 2l1.

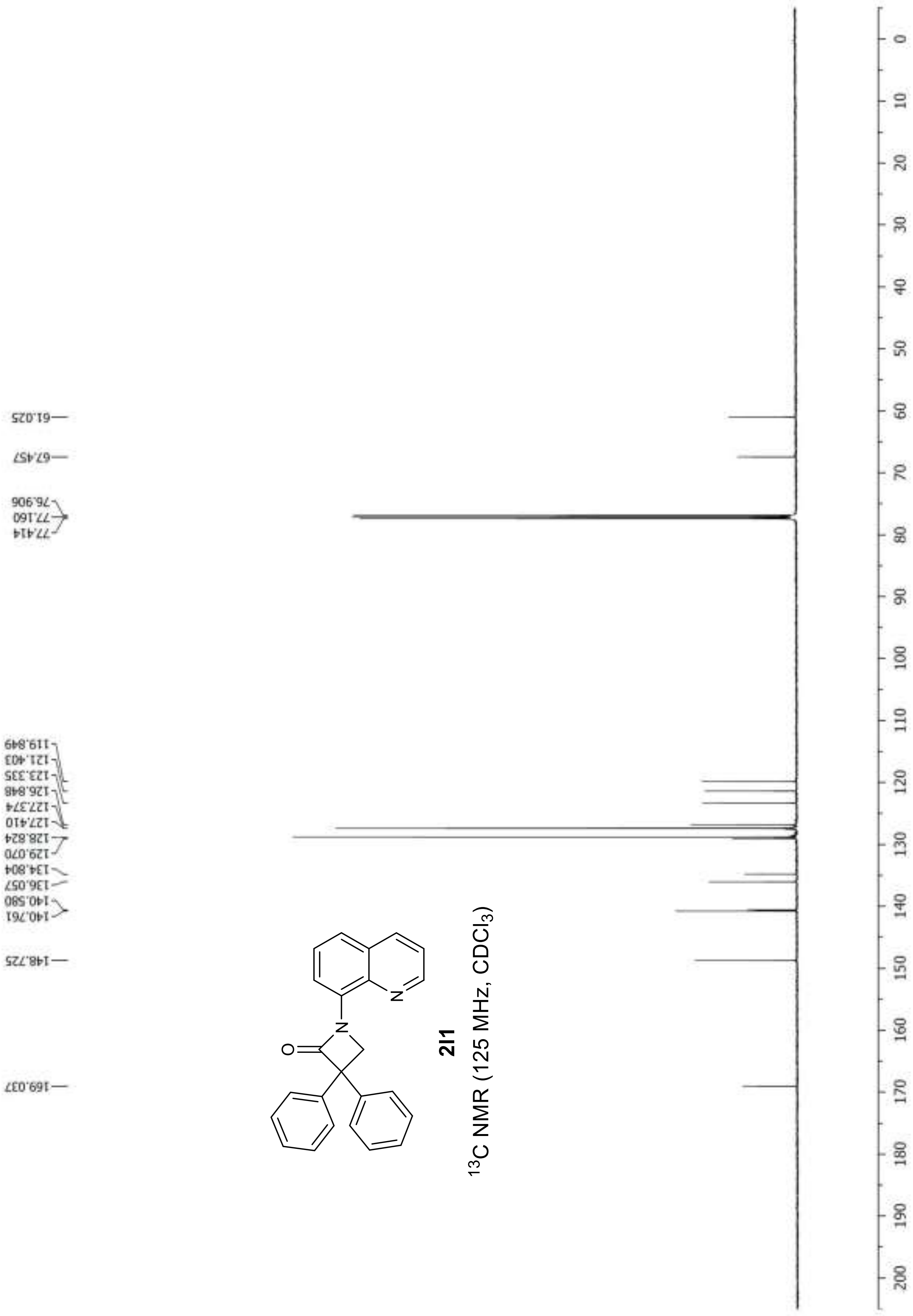

Supplementary Figure 56. ^{13}C NMR (125 MHz, $CDCl_3$) spectrum for 2l1.

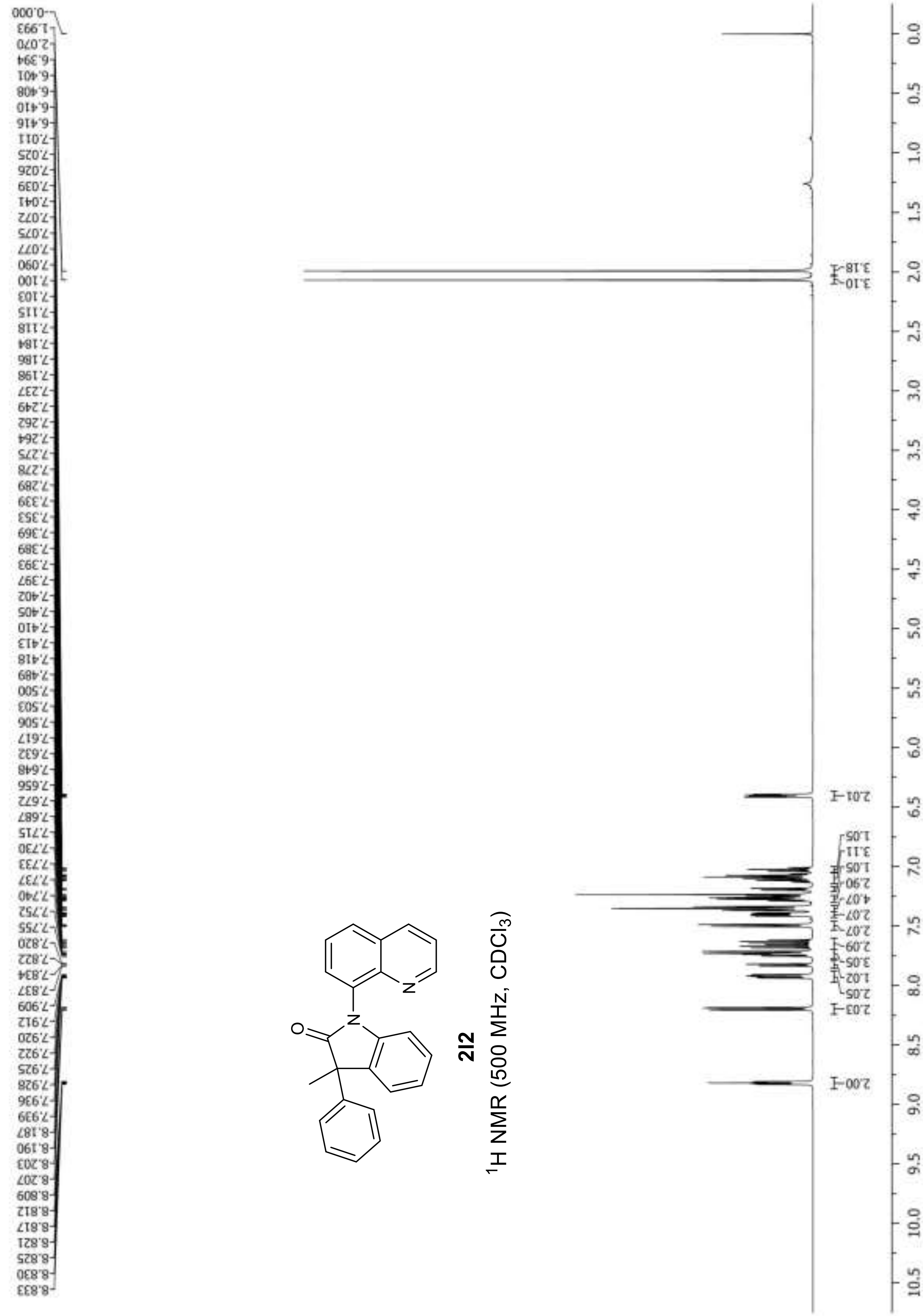

Supplementary Figure 57. ^{1}H NMR (500 MHz, $CDCl_3$) spectrum for 2l2.

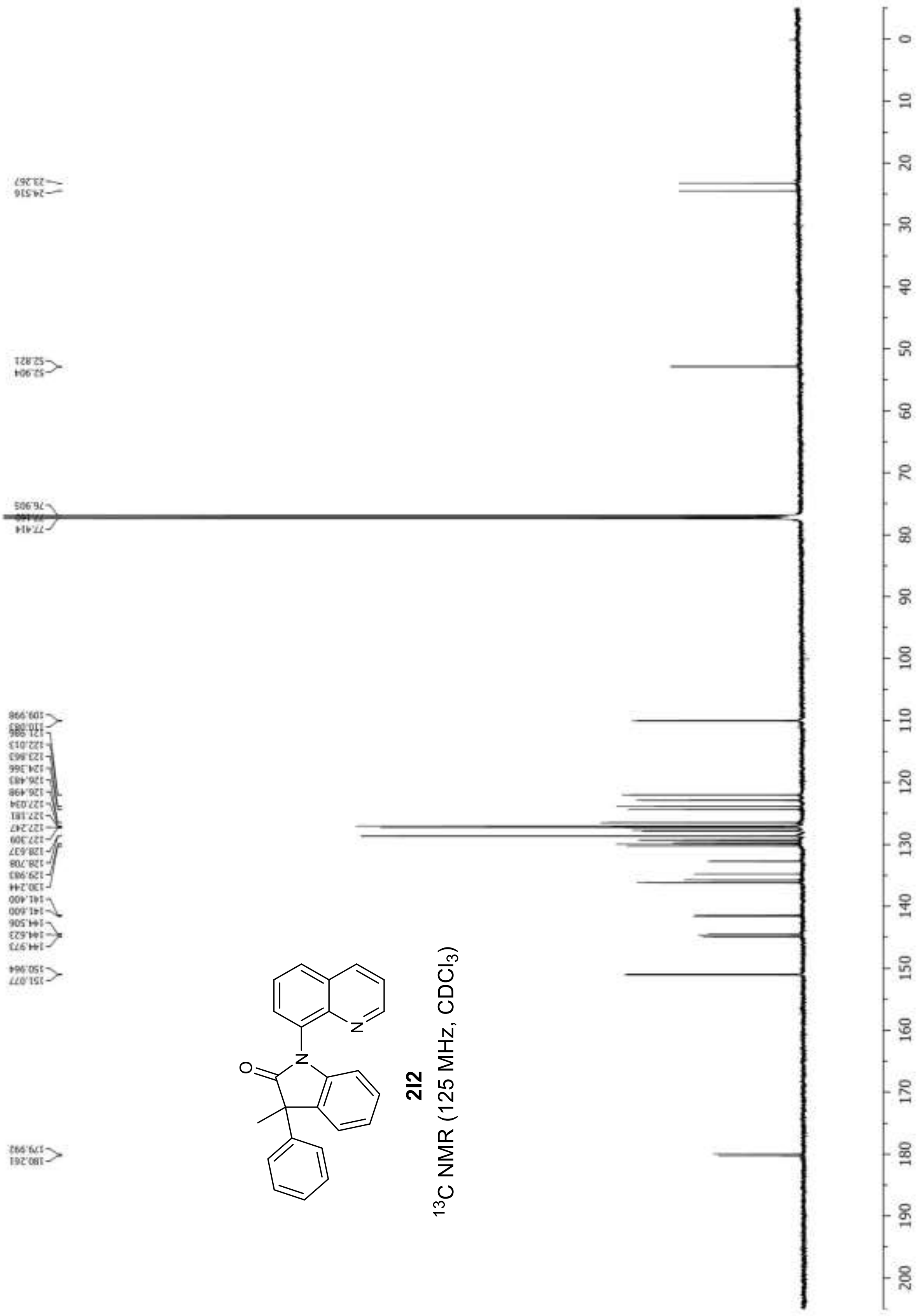

Supplementary Figure 58. ^{13}C NMR (125 MHz, $CDCl_3$) spectrum for 2l2.

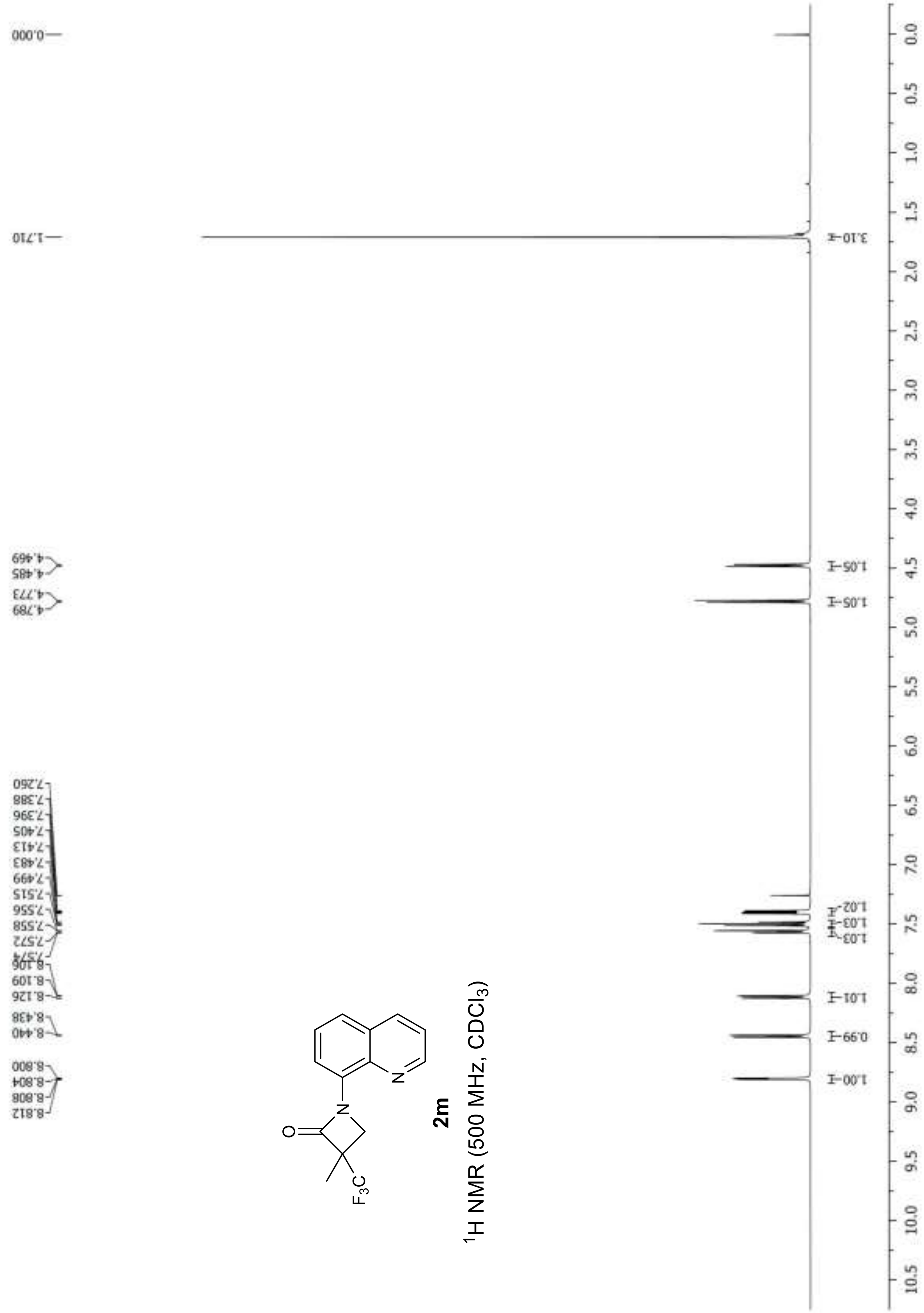

Supplementary Figure 59. ^{1}H NMR (500 MHz, CDCl$_3$) spectrum for 2m.

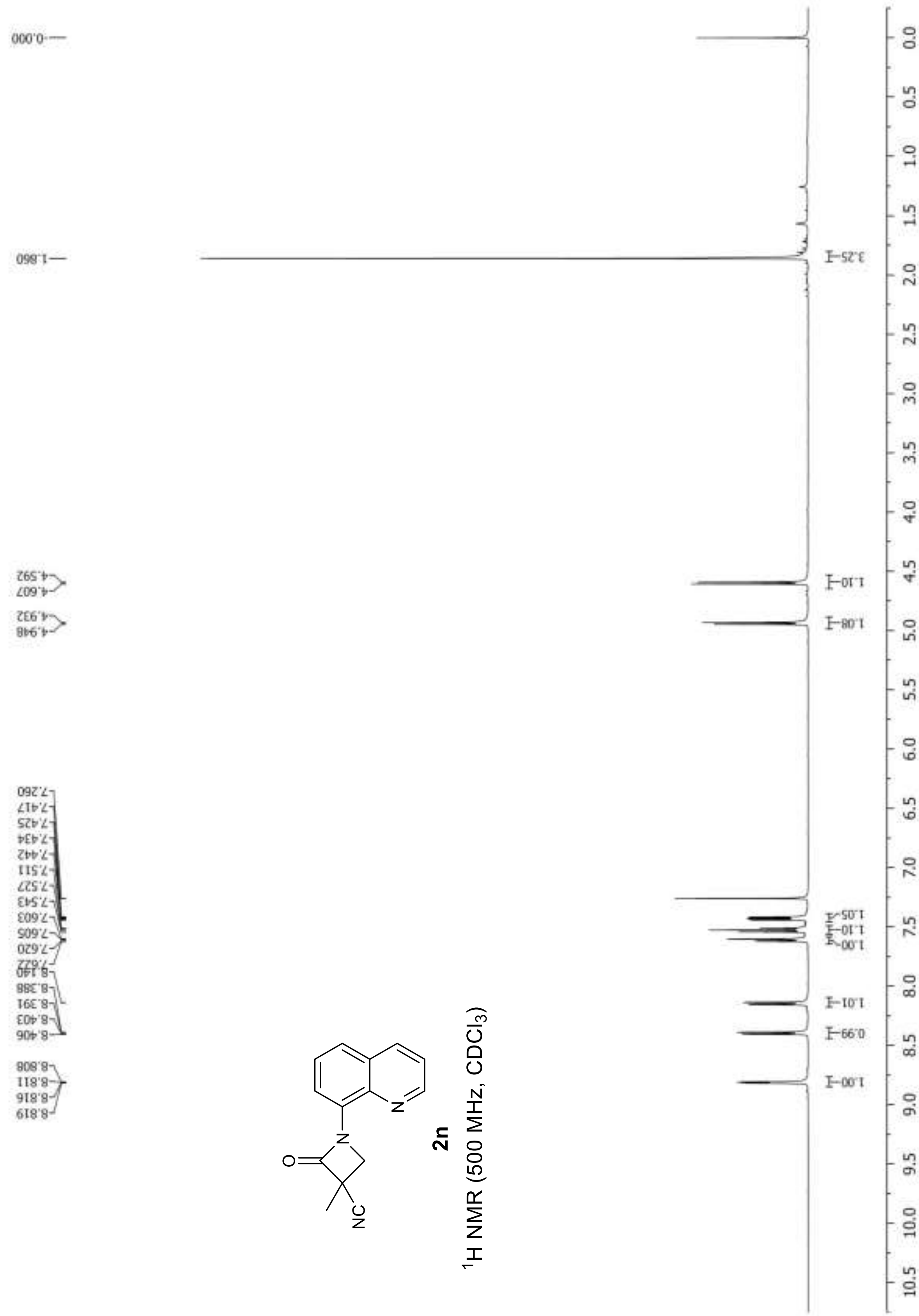

Supplementary Figure 60. ^{1}H NMR (500 MHz, $CDCl_3$) spectrum for 2n.

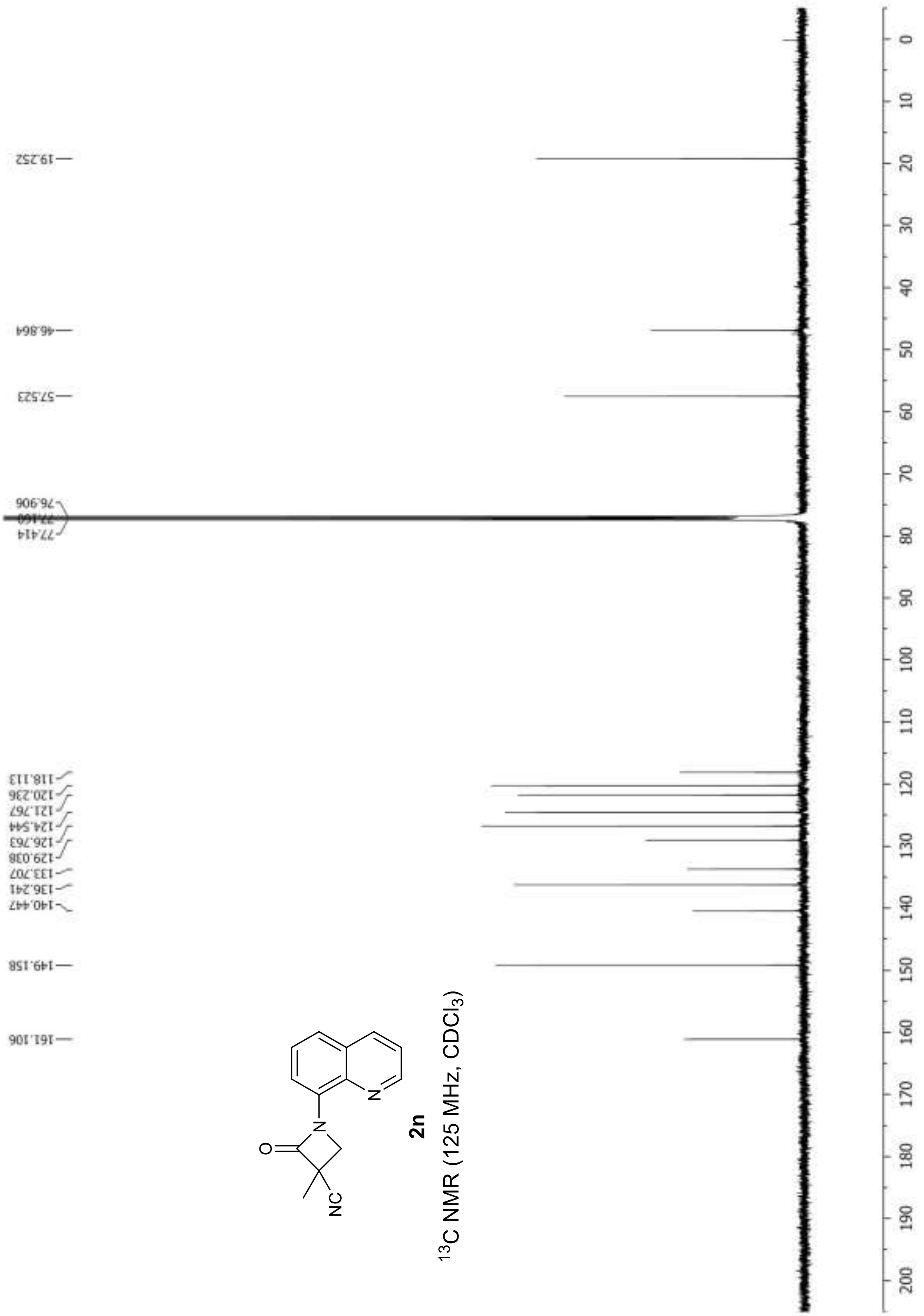

Supplementary Figure 61. ^{13}**C NMR (125 MHz, CDCl**$_3$**) spectrum for 2n.**

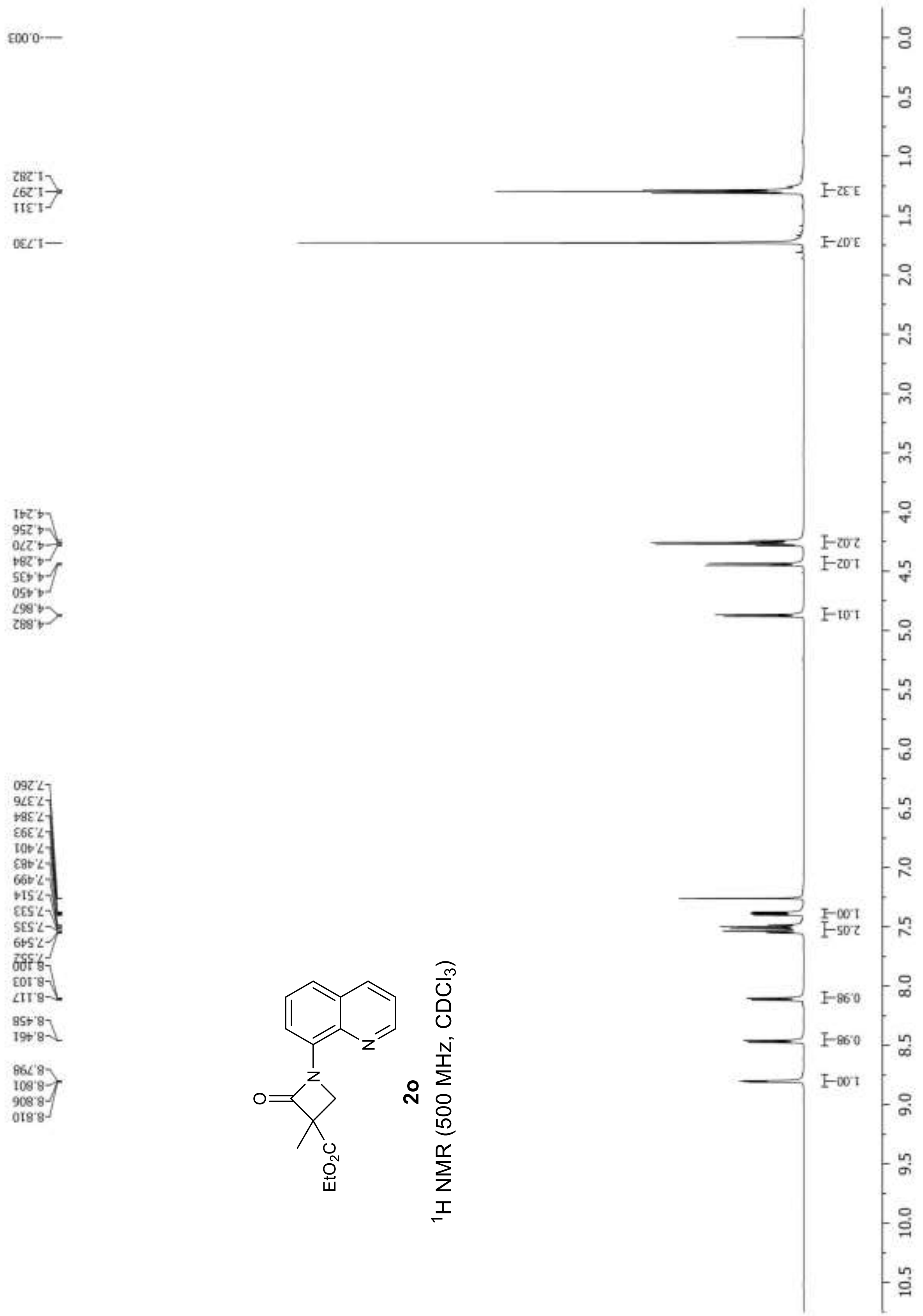

Supplementary Figure 62. **^{1}H NMR (500 MHz, $CDCl_3$) spectrum for 2o.**

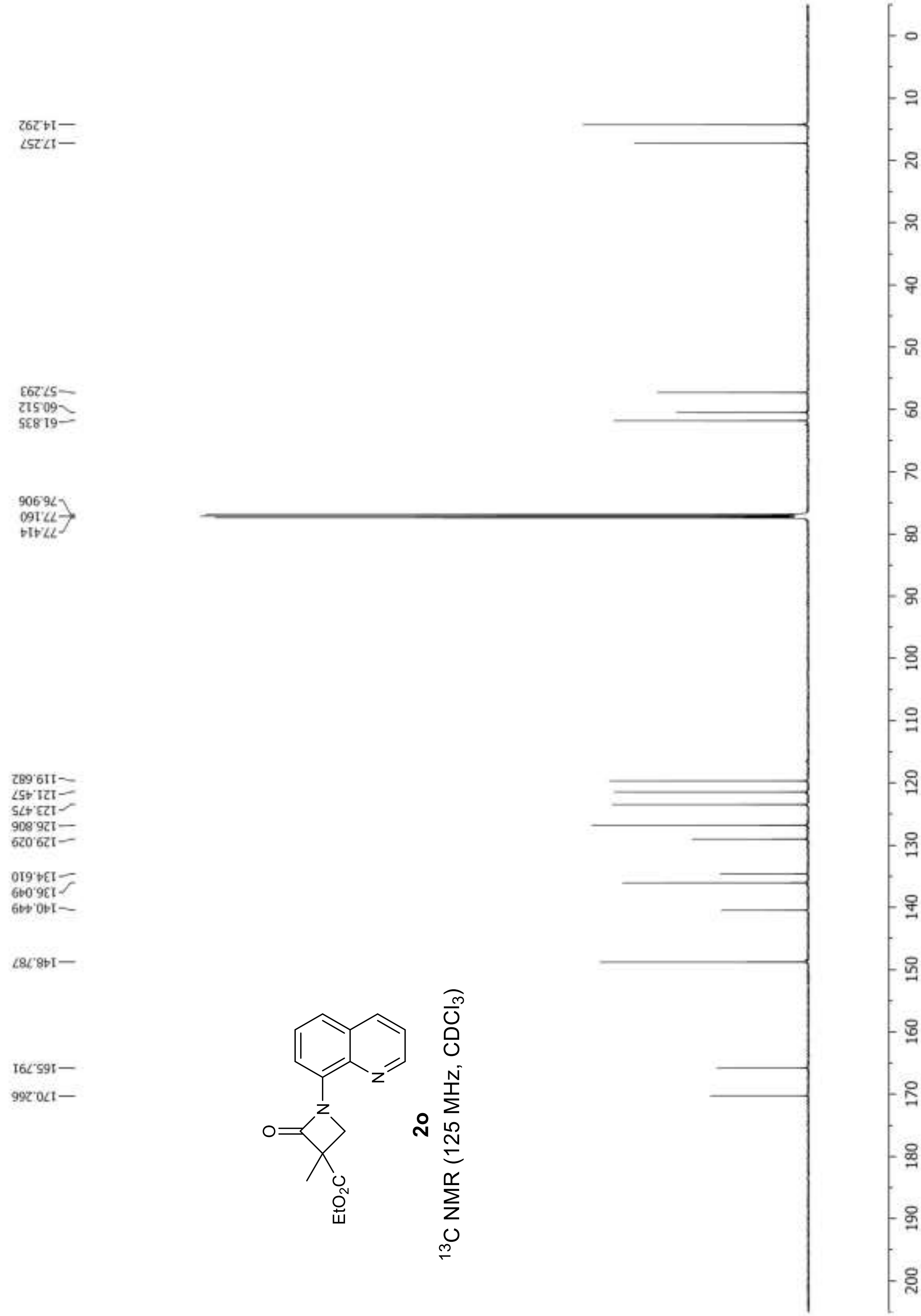

Supplementary Figure 63. ^{13}**C NMR (125 MHz, CDCl**$_3$**) spectrum for 2o.**

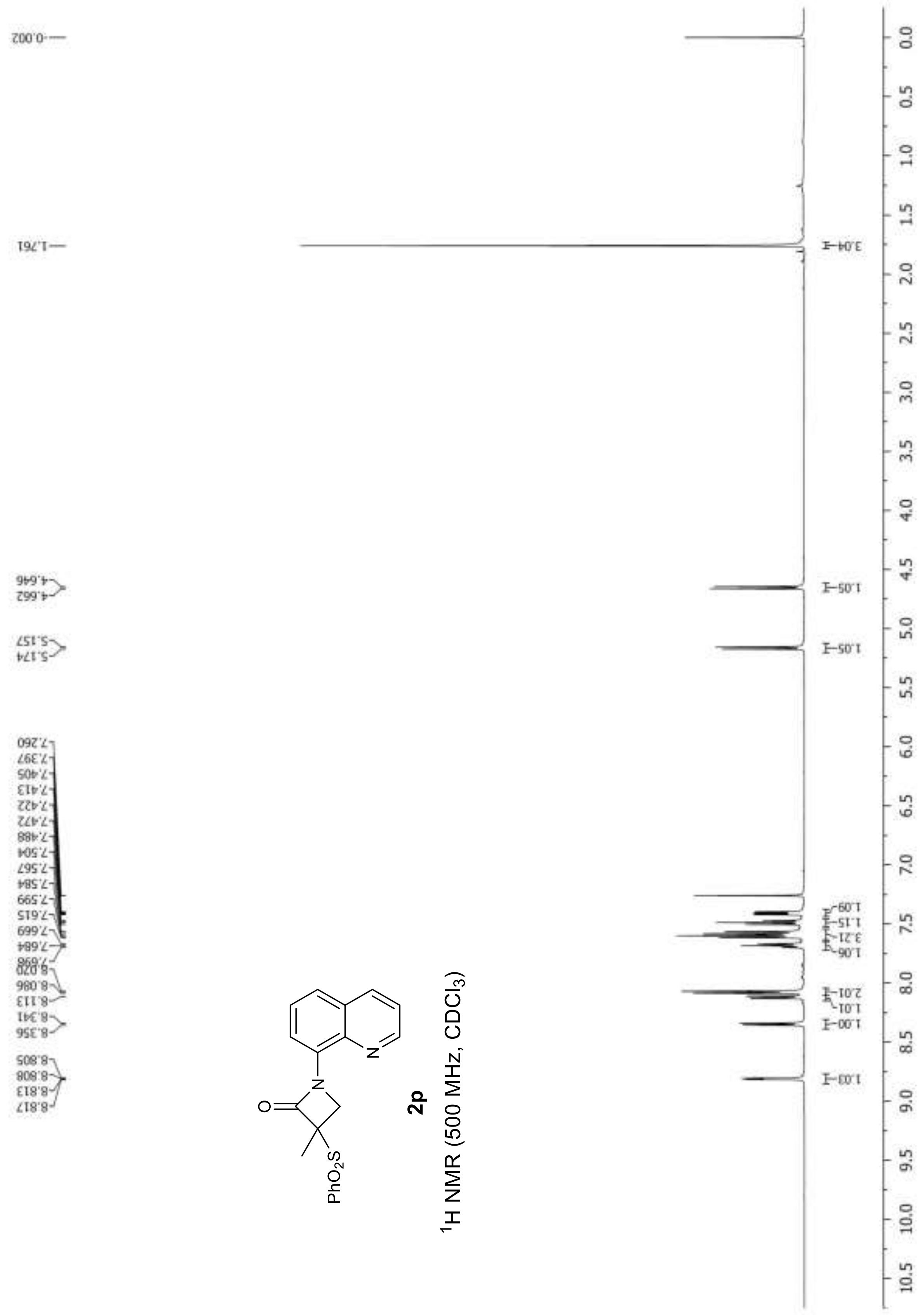

Supplementary Figure 64. ^{1}H NMR (500 MHz, $CDCl_3$) spectrum for 2p.

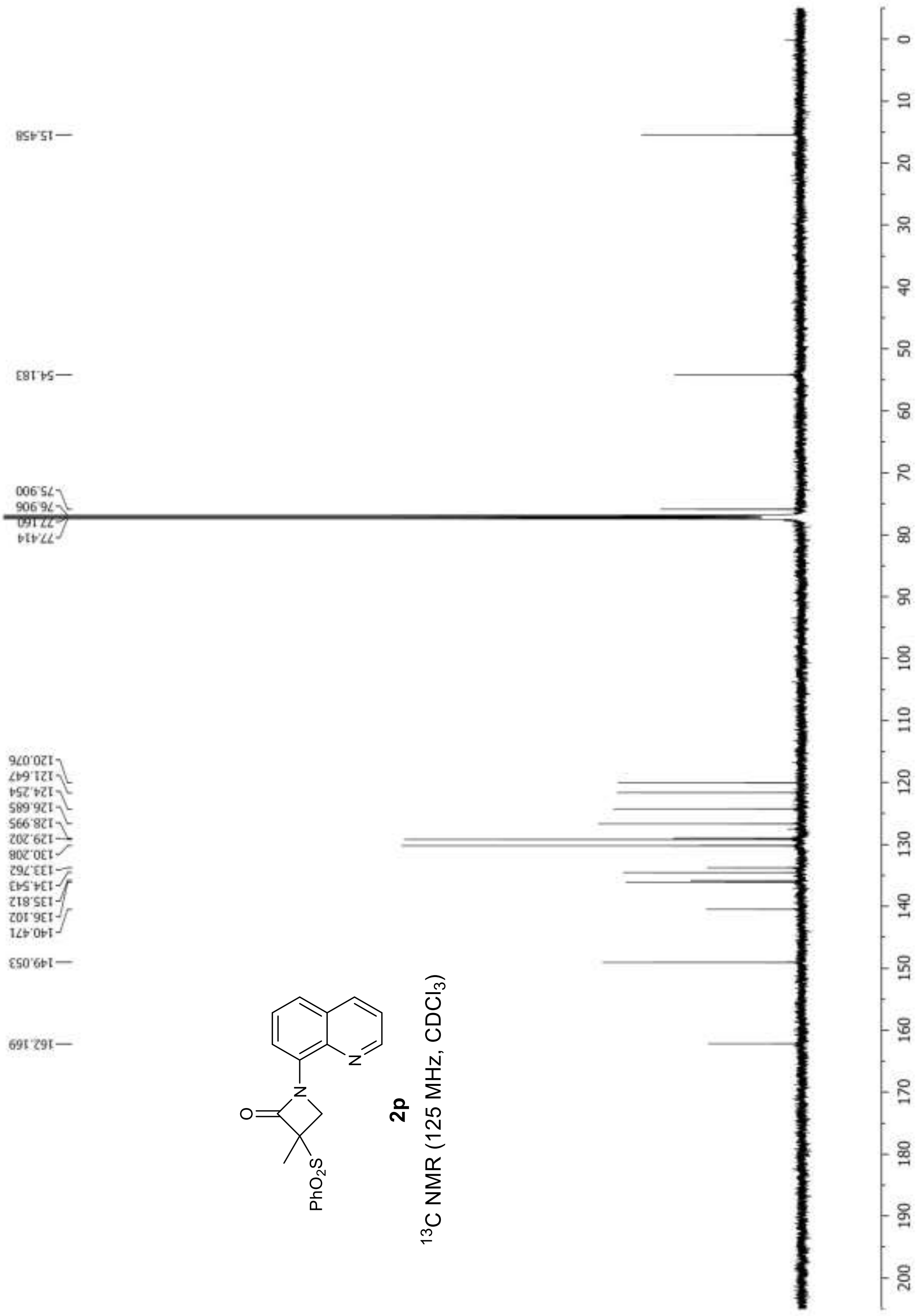

Supplementary Figure 65. ^{13}C NMR (125 MHz, $CDCl_3$) spectrum for 2p.

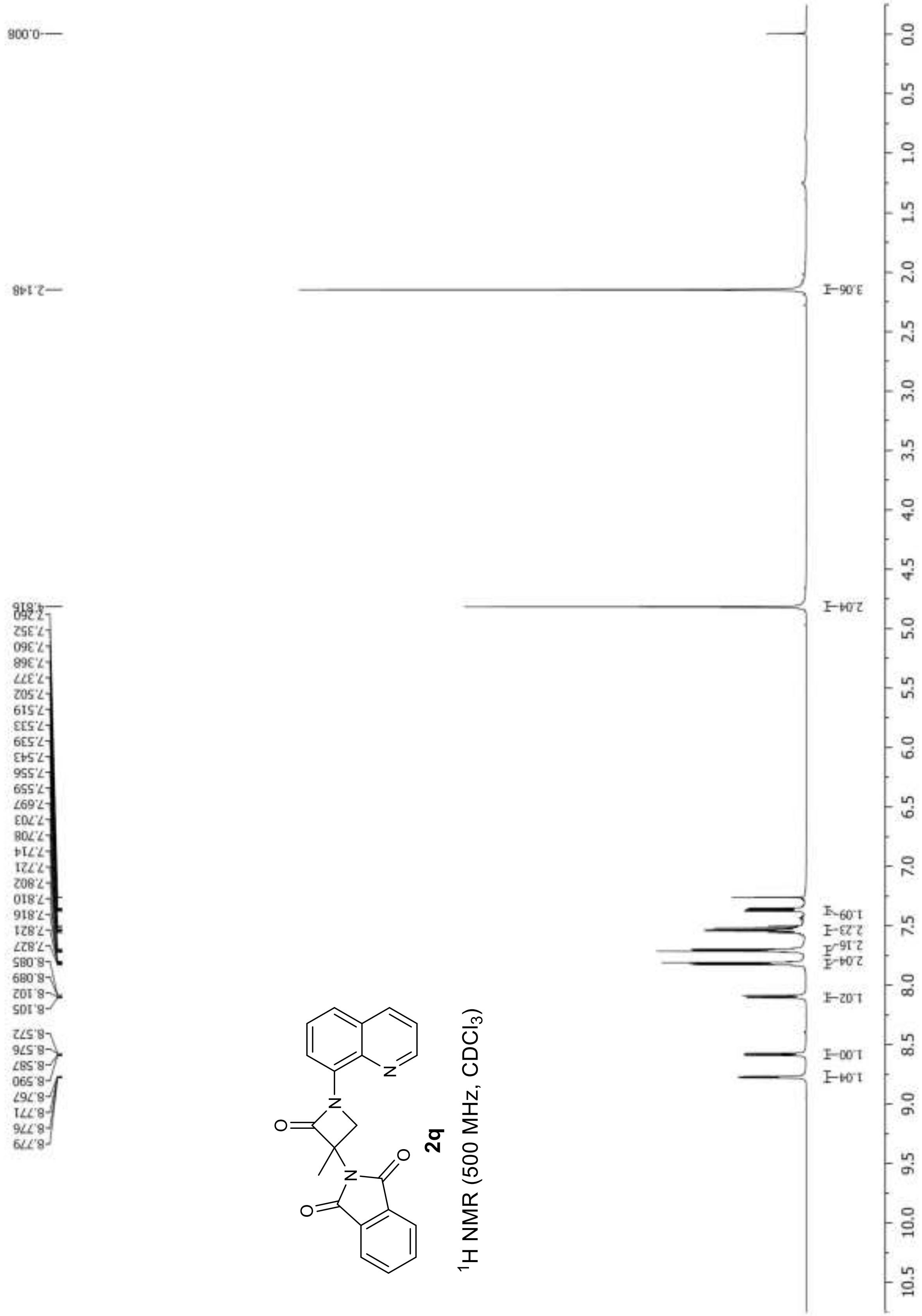

Supplementary Figure 66. ^{1}H NMR (500 MHz, $CDCl_3$) spectrum for 2q.

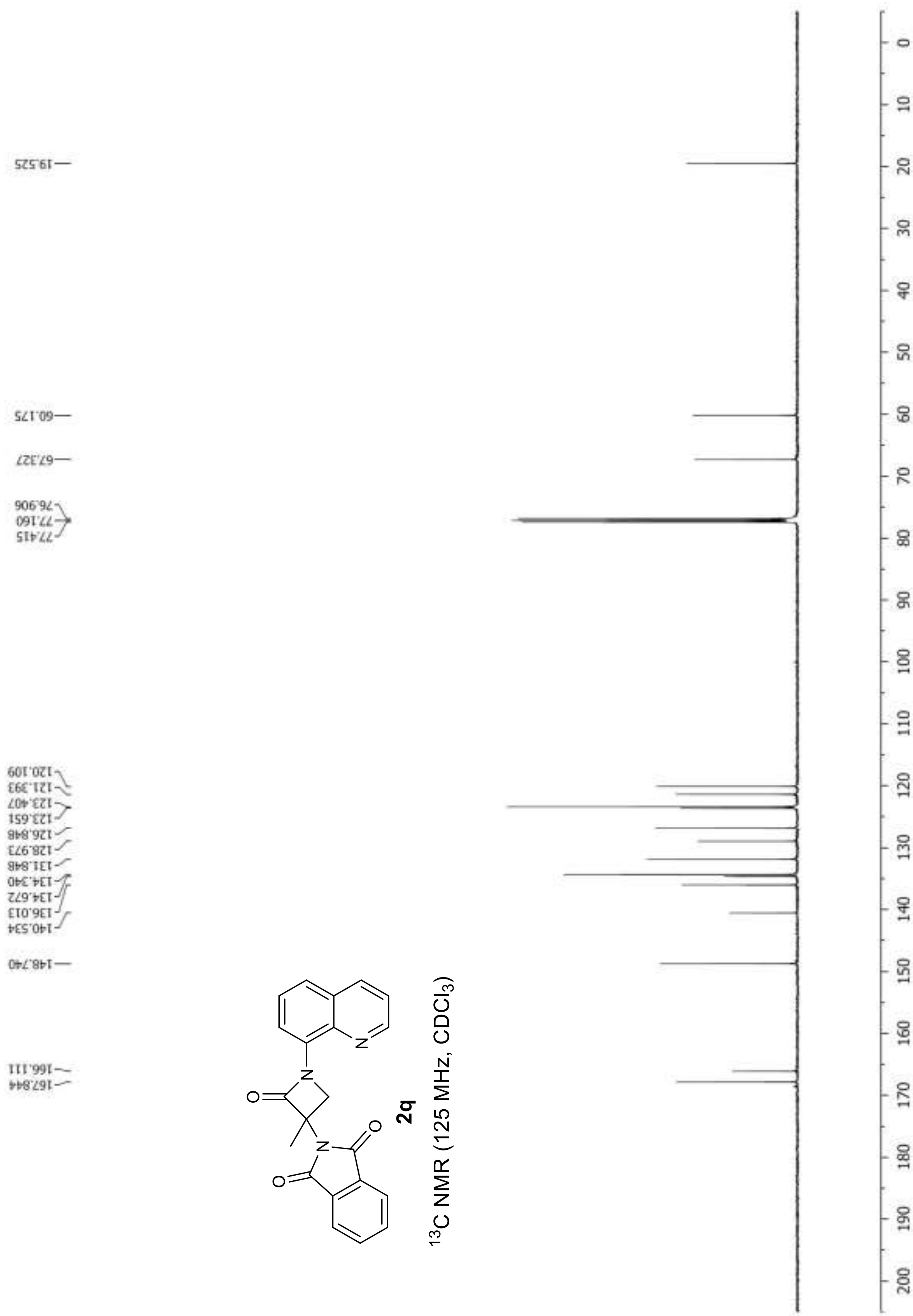

Supplementary Figure 67. ^{13}C NMR (125 MHz, $CDCl_3$) spectrum for 2q.

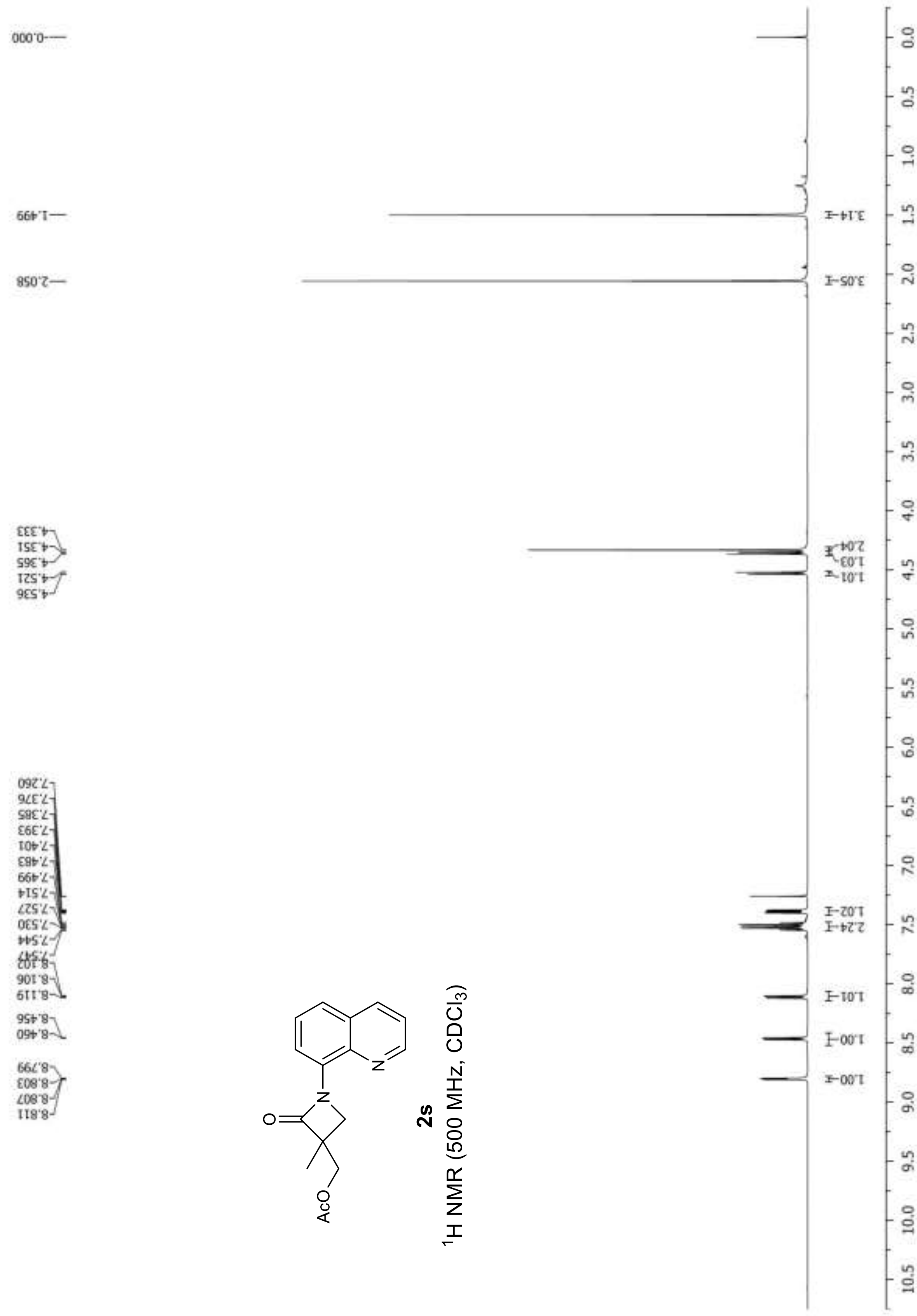

Supplementary Figure 68. ^{1}H NMR (500 MHz, $CDCl_3$) spectrum for 2s.

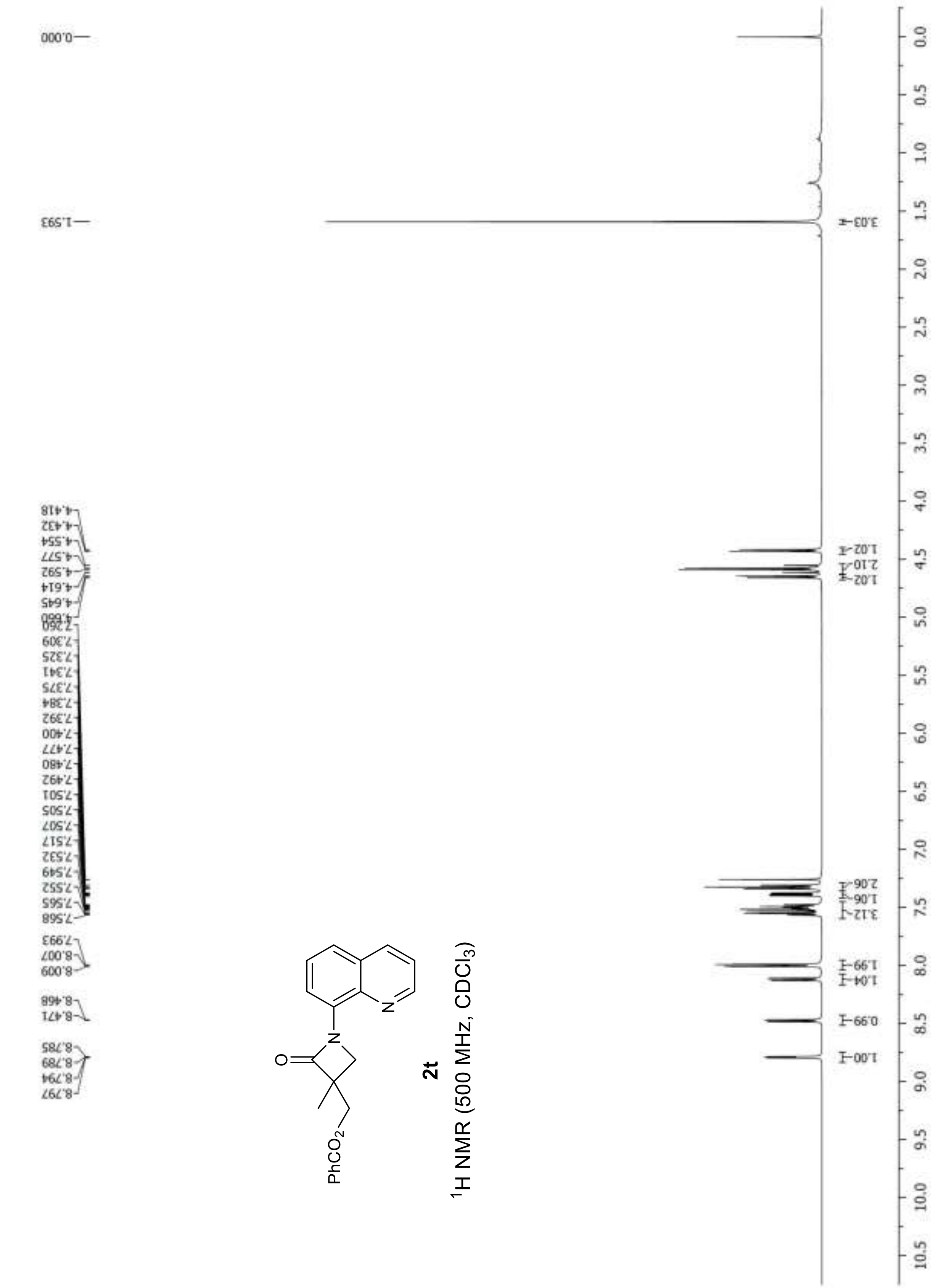

Supplementary Figure 69. ^{1}H NMR (500 MHz, $CDCl_3$) spectrum for 2t.

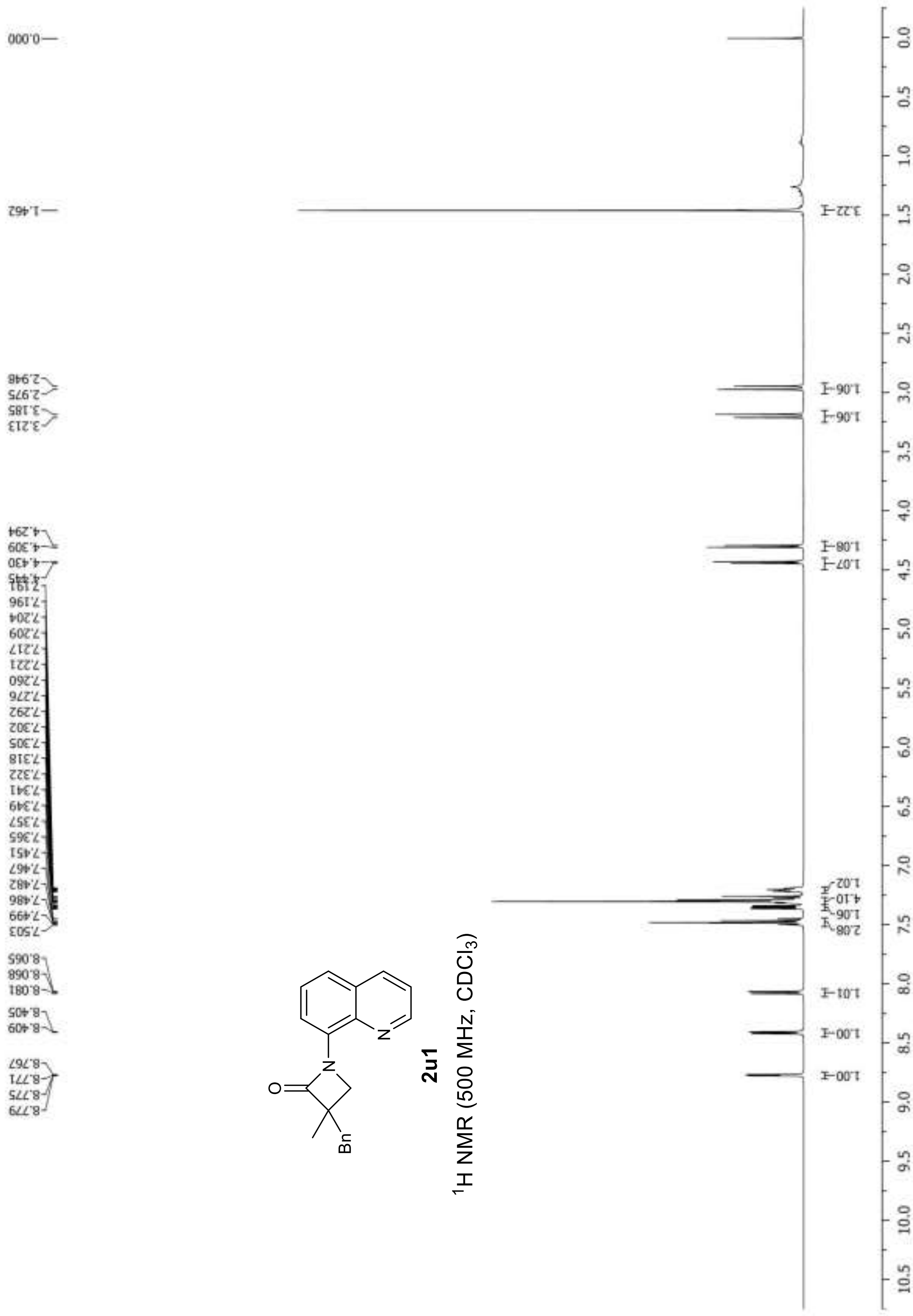

Supplementary Figure 70. ^{1}**H NMR (500 MHz,** $CDCl_3$**) spectrum for 2u1.**

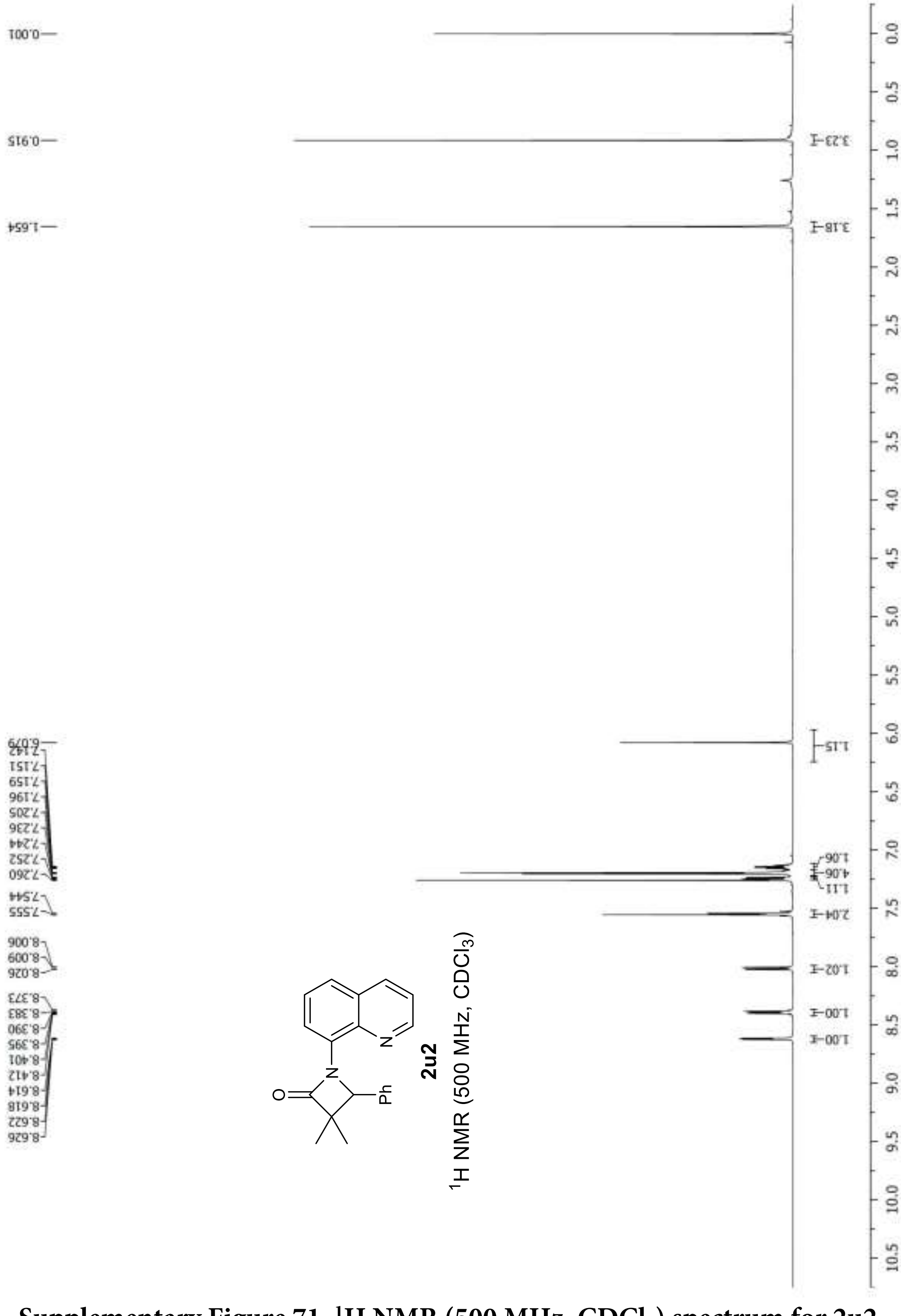

Supplementary Figure 71. ^1H NMR (500 MHz, $CDCl_3$) spectrum for 2u2.

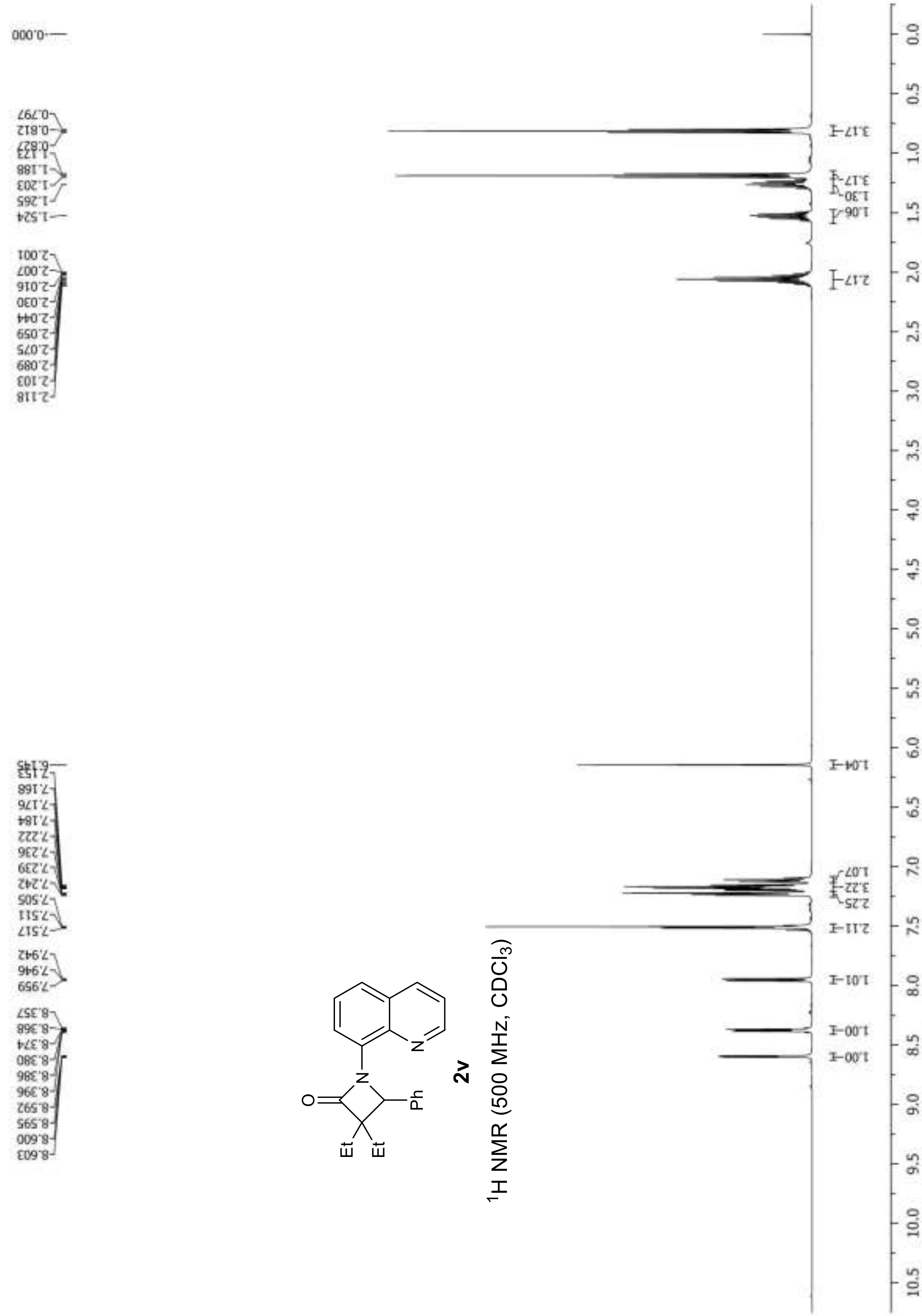

Supplementary Figure 72. ^{1}H NMR (500 MHz, $CDCl_3$) spectrum for 2v.

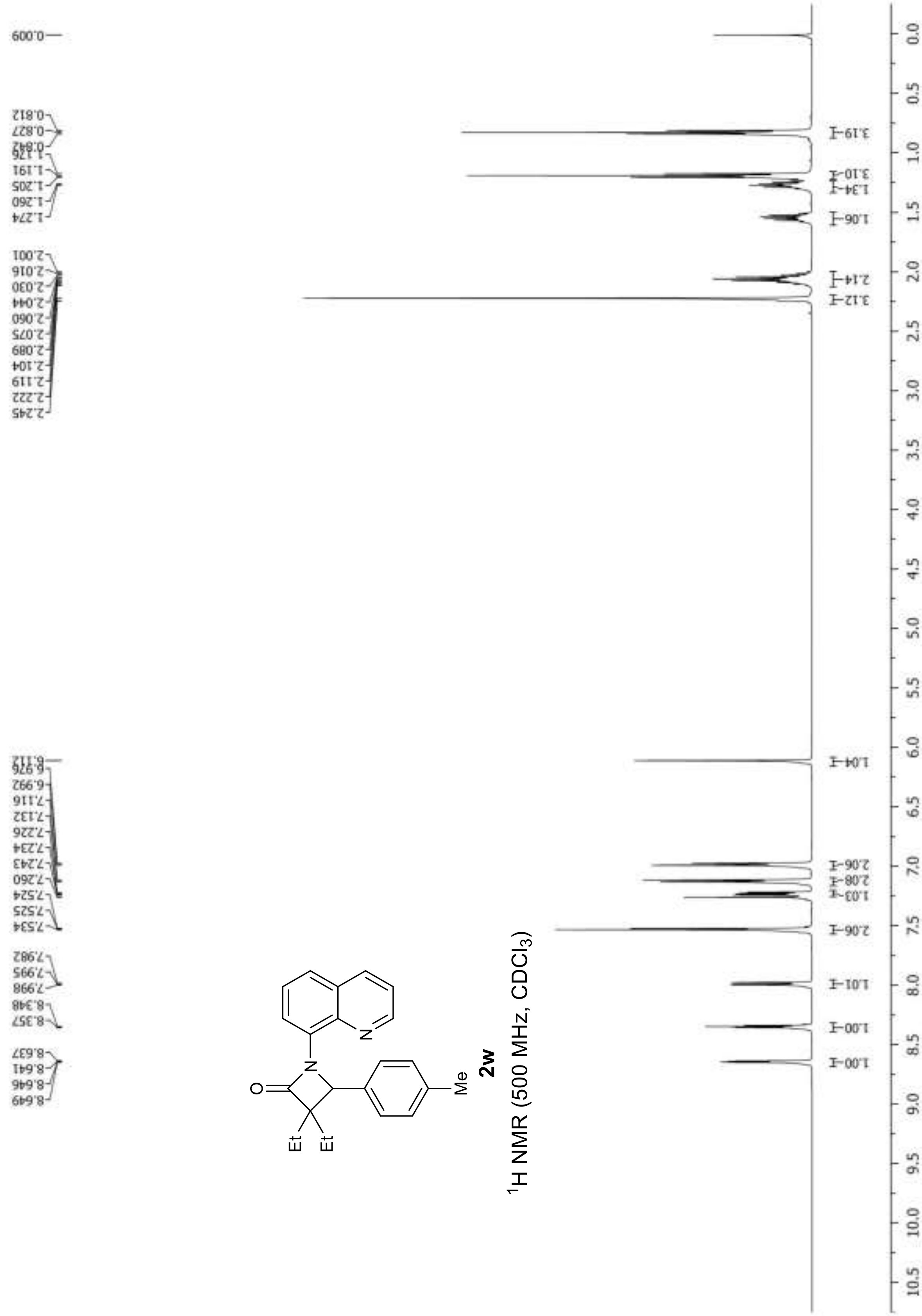

Supplementary Figure 73. ^{1}H NMR (500 MHz, $CDCl_3$) spectrum for 2w.

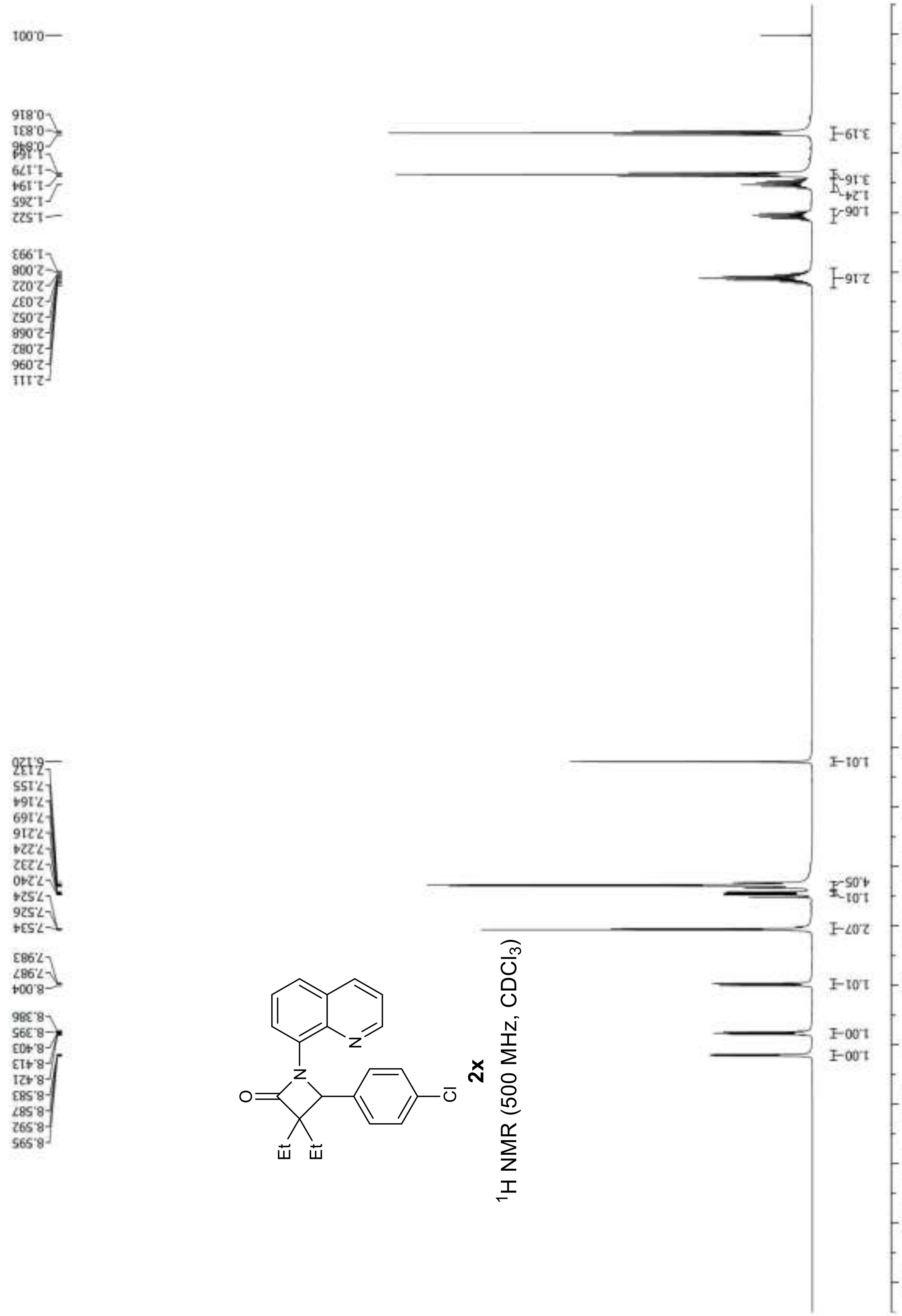

Supplementary Figure 74. ^{1}H NMR (500 MHz, $CDCl_3$) spectrum for 2x.

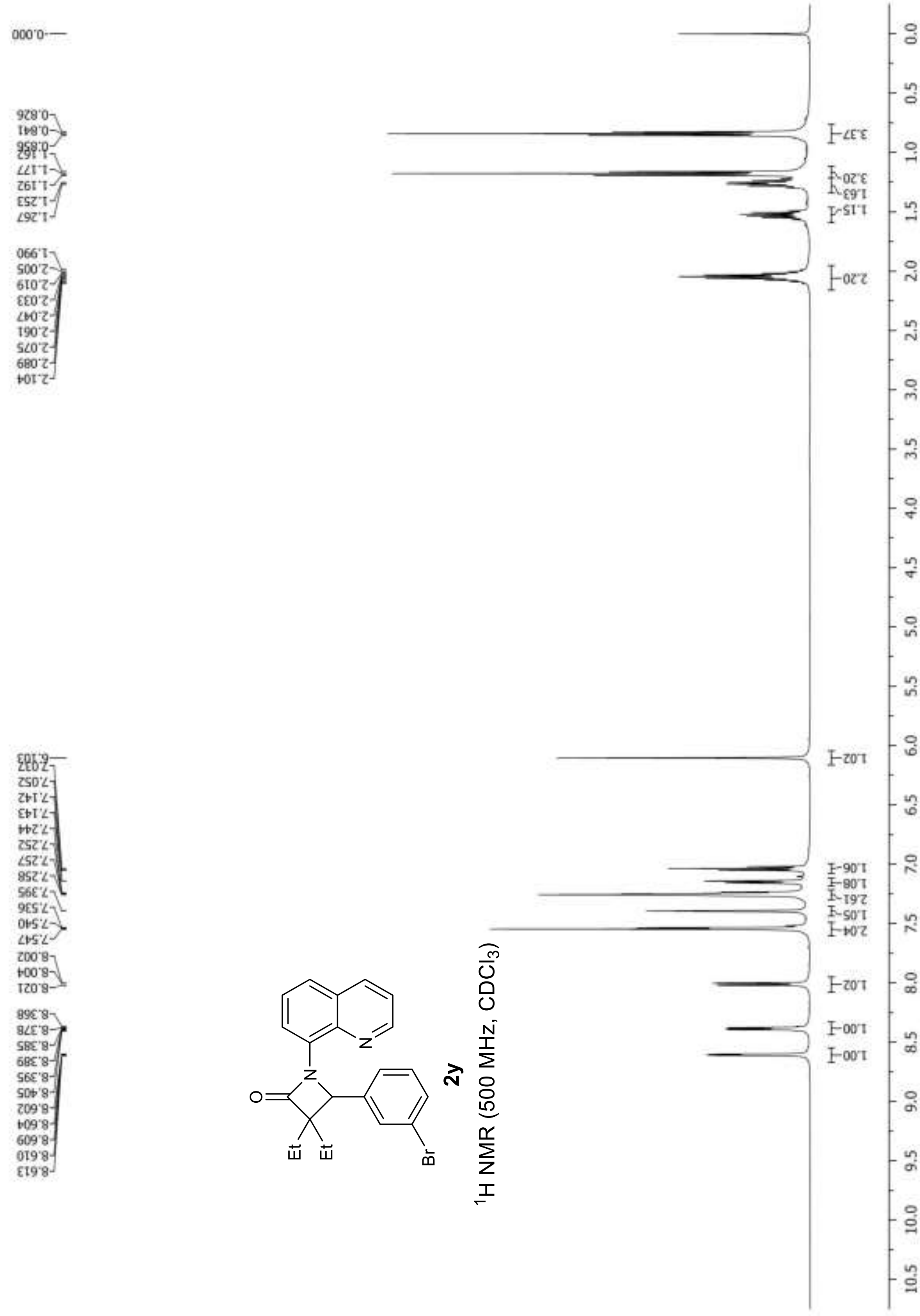

Supplementary Figure 75. ^{1}H NMR (500 MHz, $CDCl_3$) spectrum for 2y.

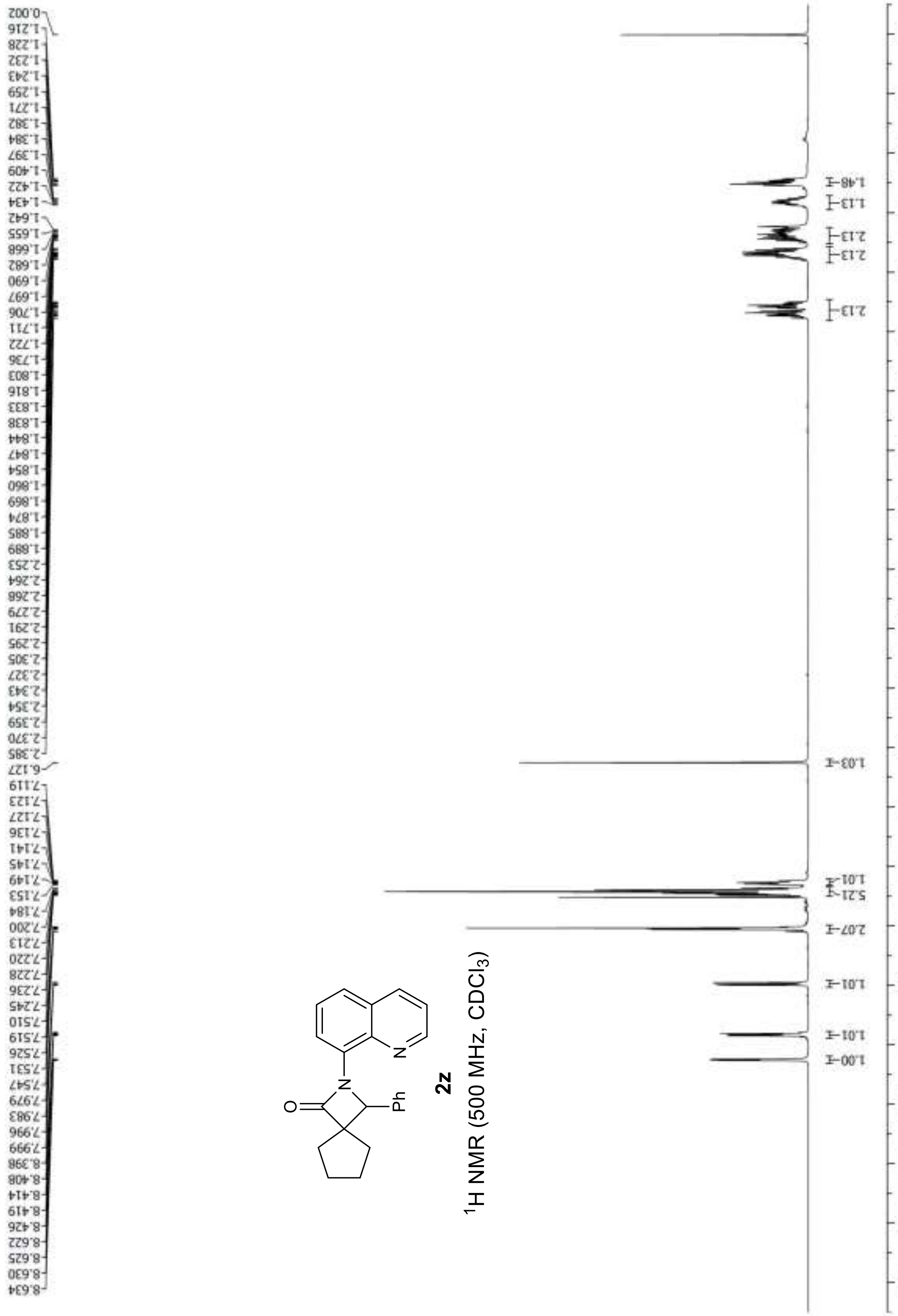

Supplementary Figure 76. ^{1}H NMR (500 MHz, $CDCl_3$) spectrum for 2z.

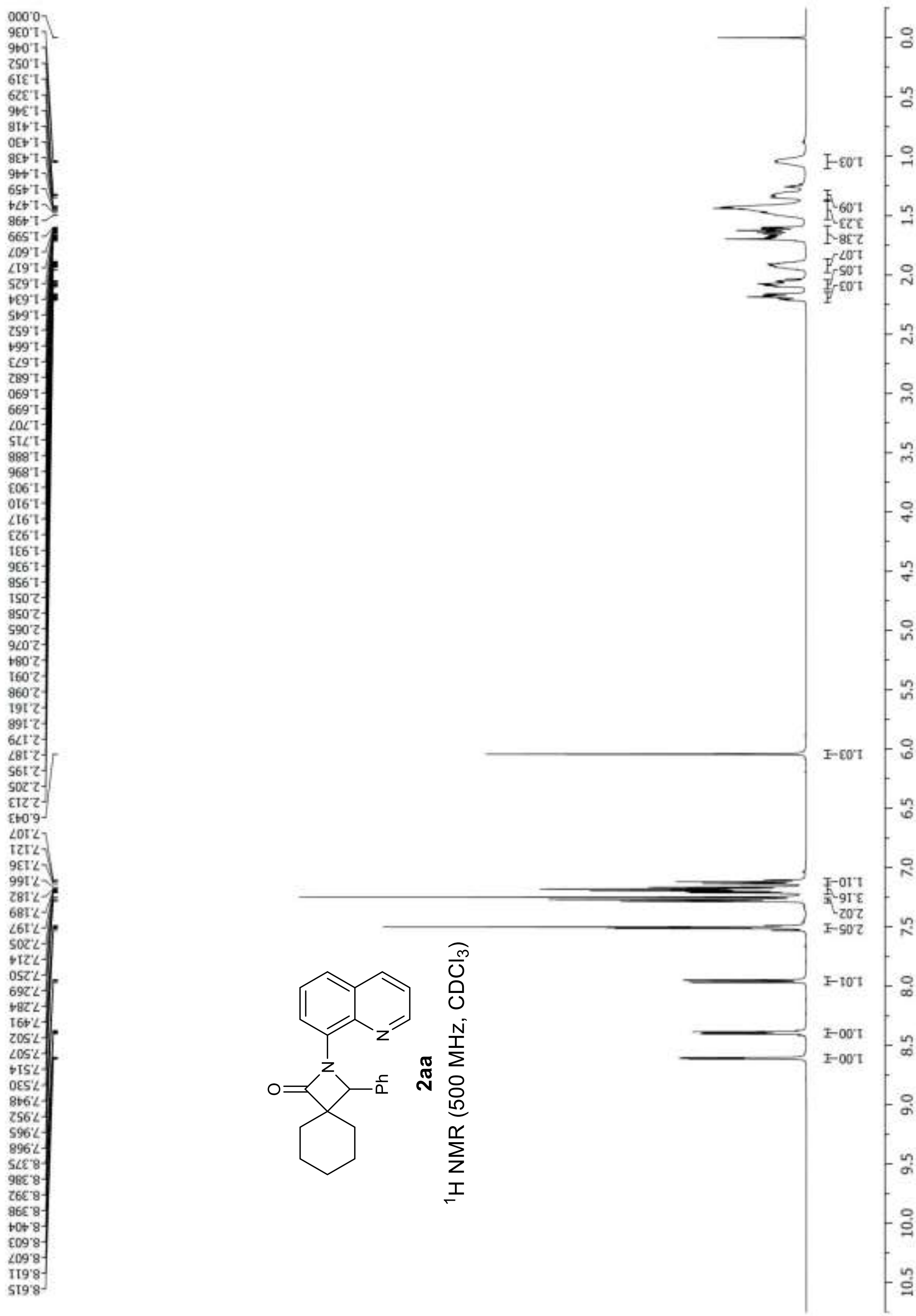

Supplementary Figure 77. ^{1}H NMR (500 MHz, $CDCl_3$) spectrum for 2aa.

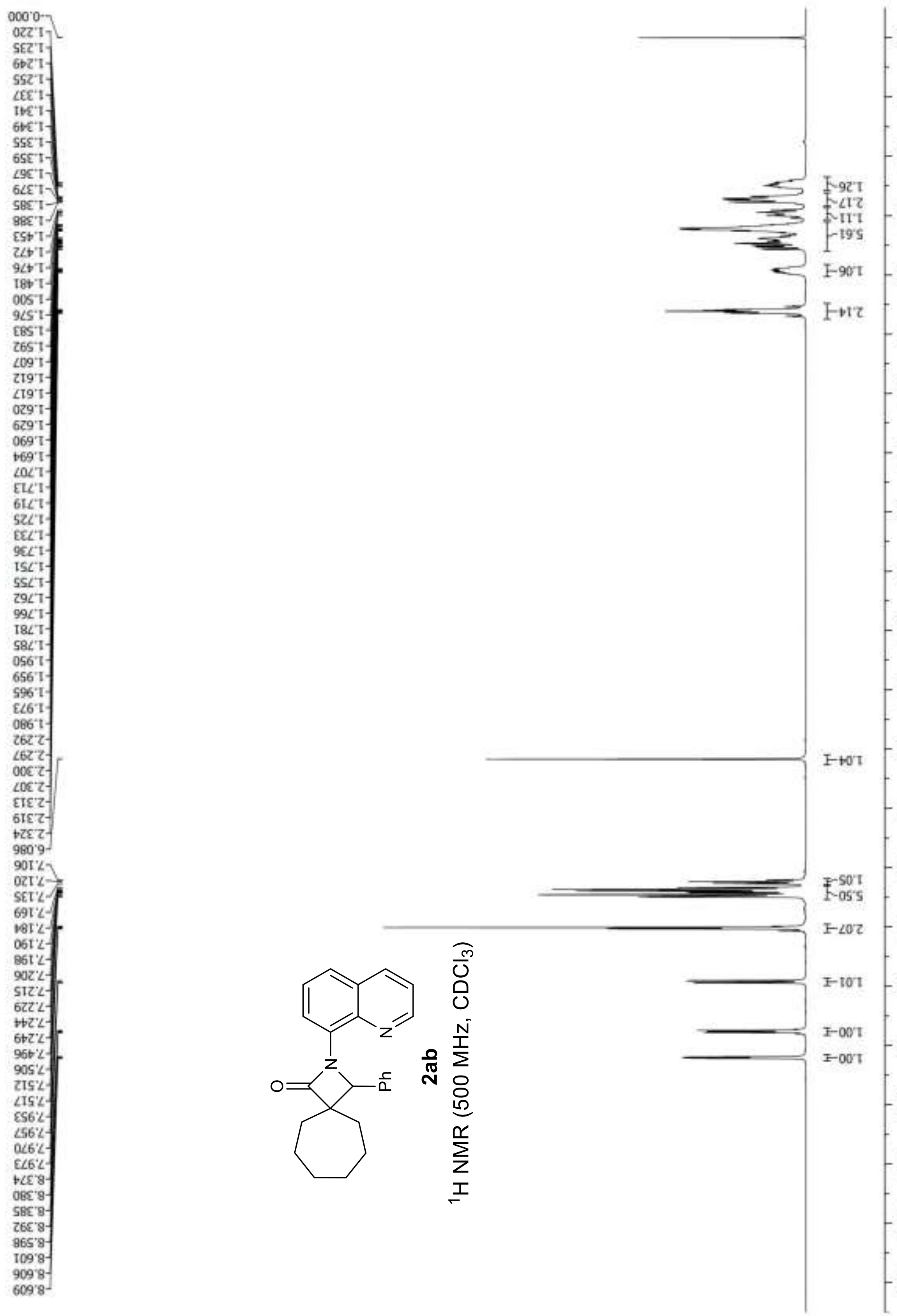

Supplementary Figure 78. ^{1}H NMR (500 MHz, $CDCl_3$) spectrum for 2ab.

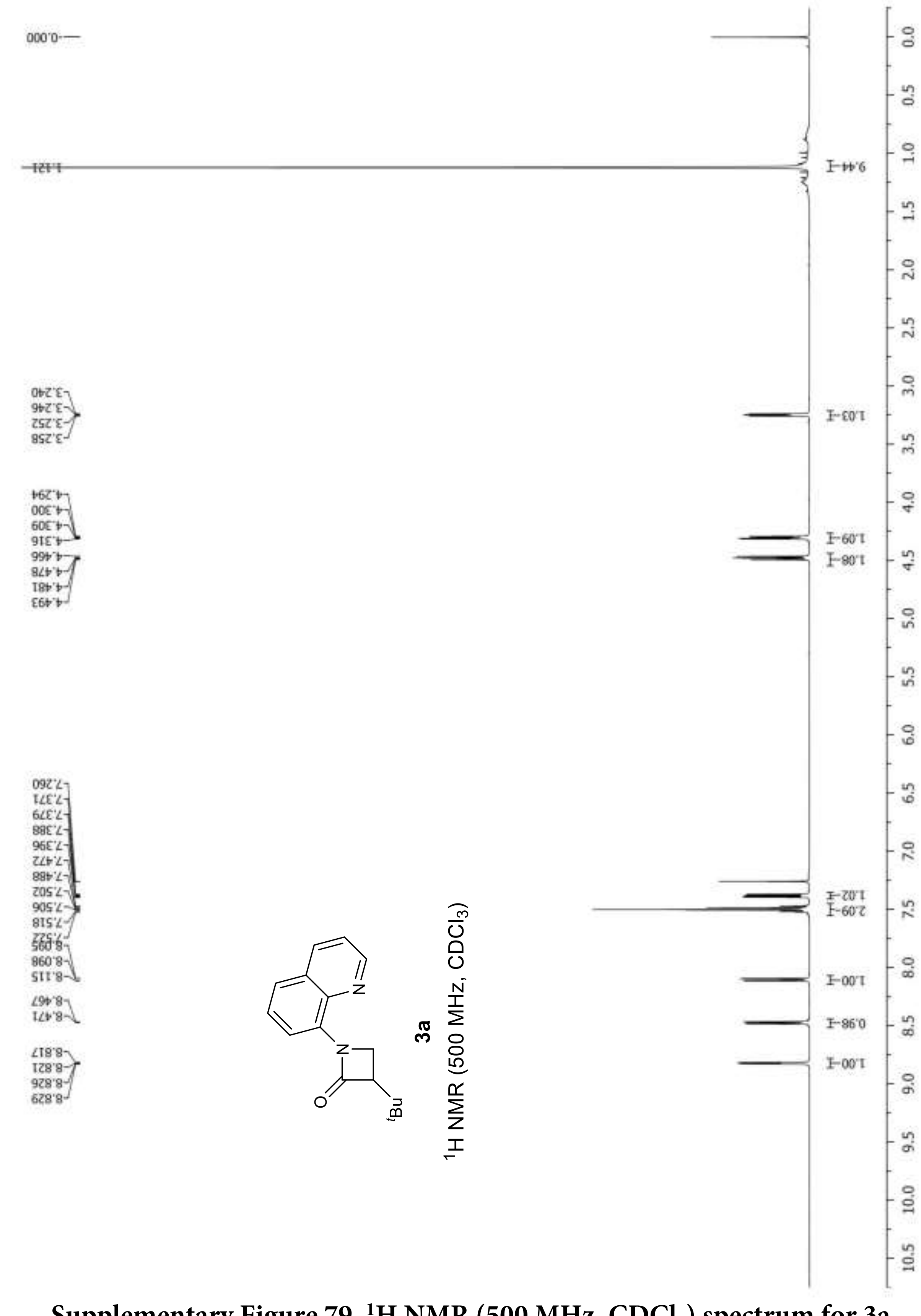

Supplementary Figure 79. ^{1}H NMR (500 MHz, $CDCl_3$) spectrum for 3a.

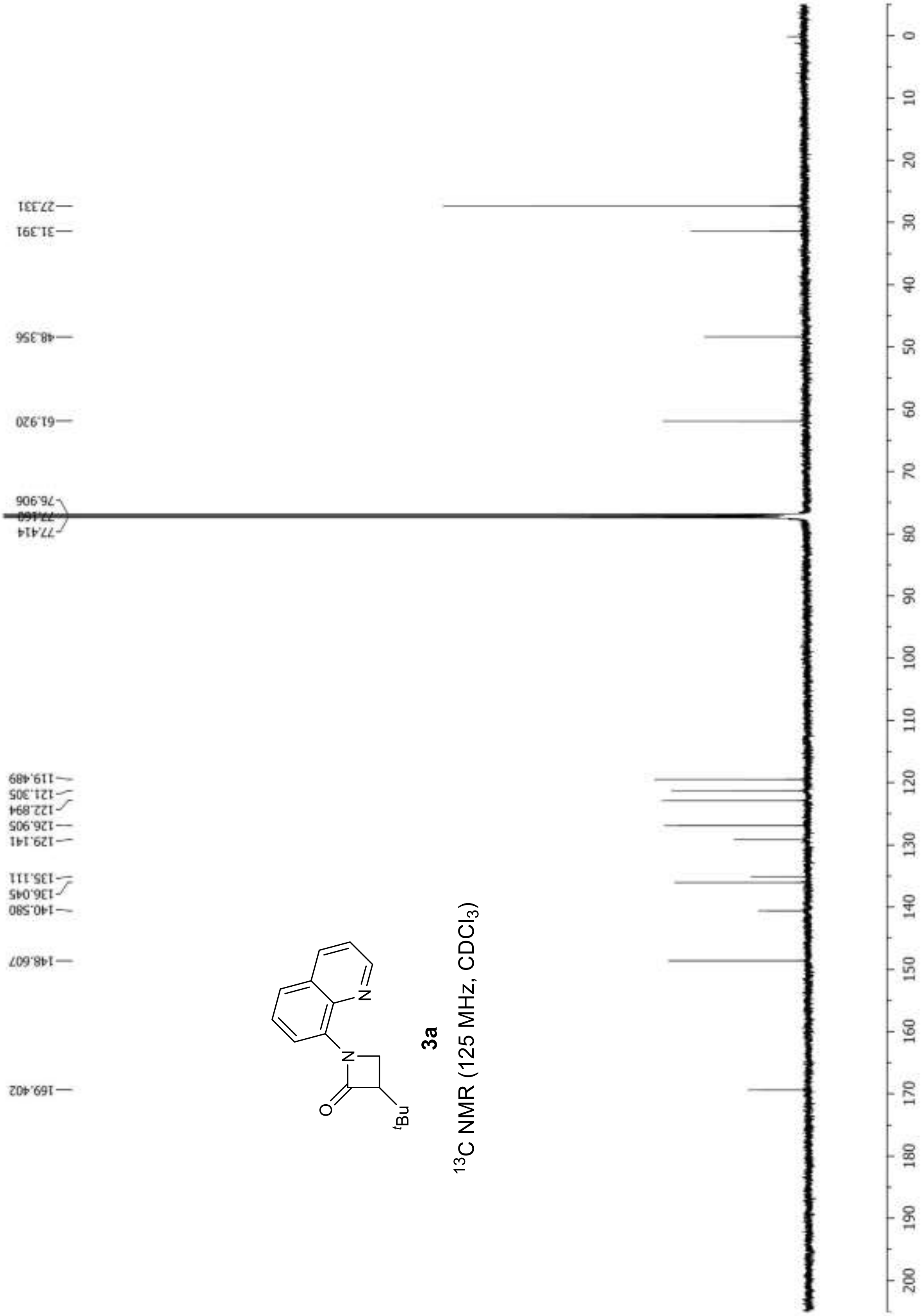

Supplementary Figure 80. ^{13}C NMR (125 MHz, $CDCl_3$) spectrum for 3a.

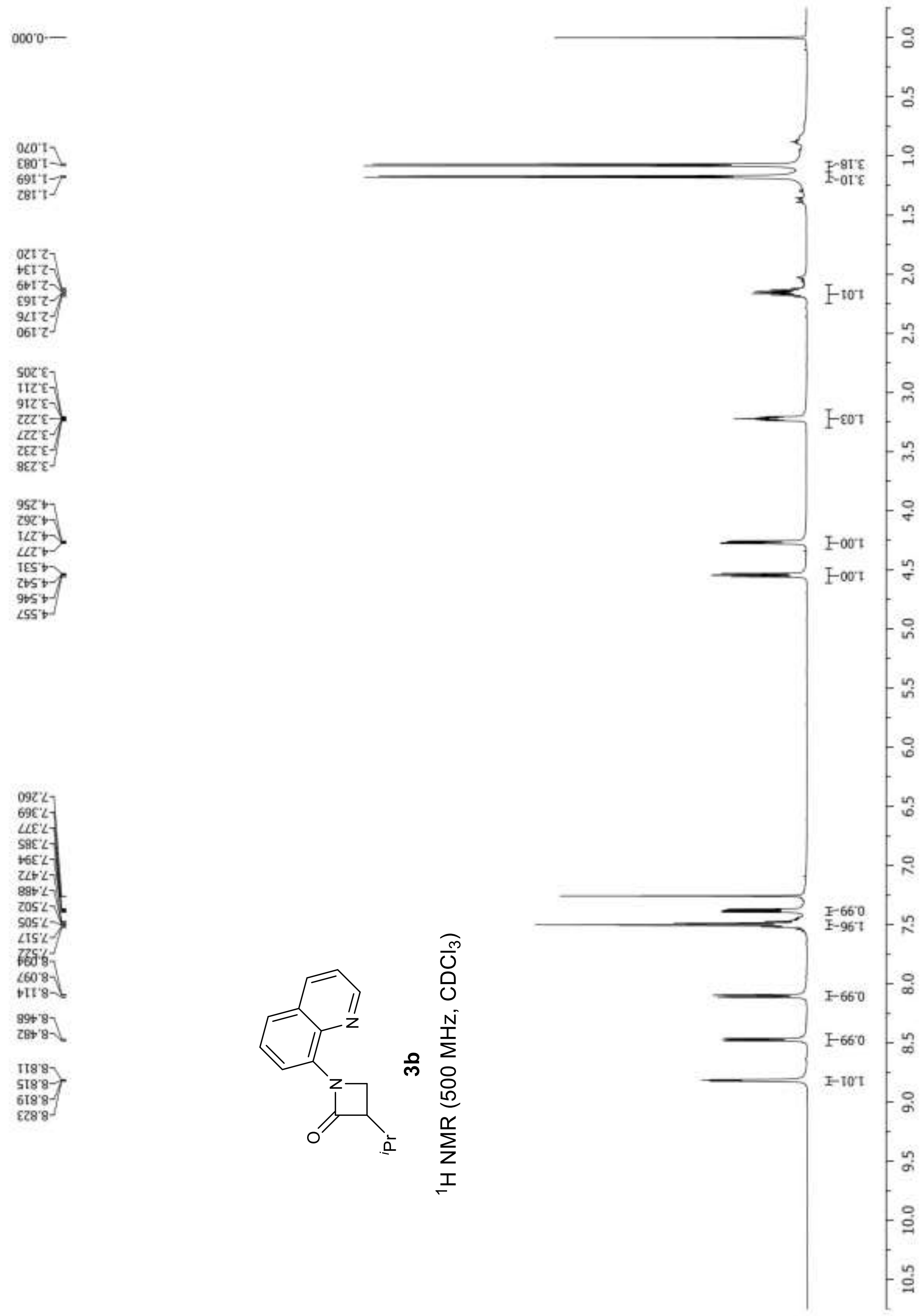

Supplementary Figure 81. ^{1}H NMR (500 MHz, $CDCl_3$) spectrum for 3b.

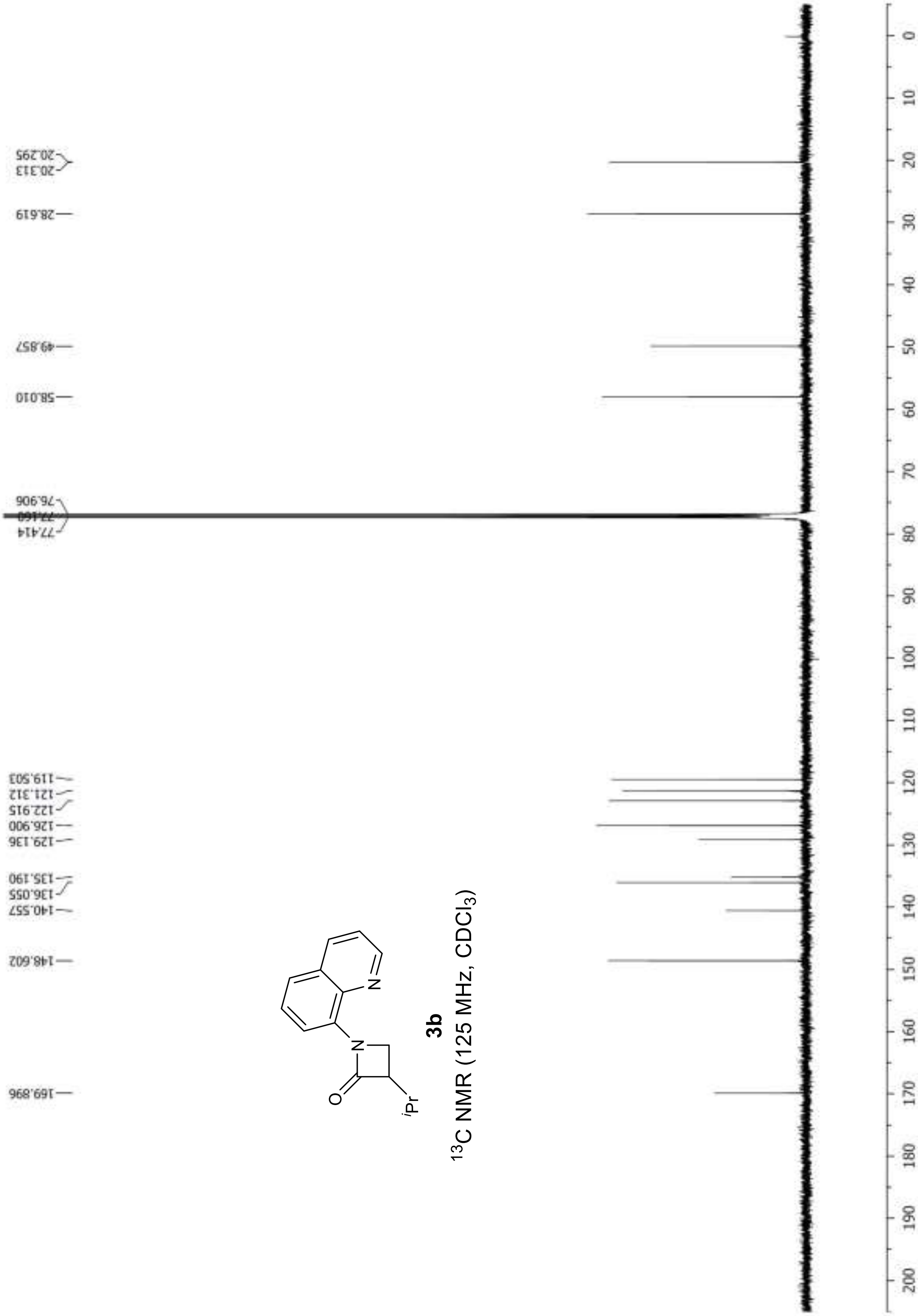

Supplementary Figure 82. ^{13}C NMR (125 MHz, $CDCl_3$) spectrum for 3b.

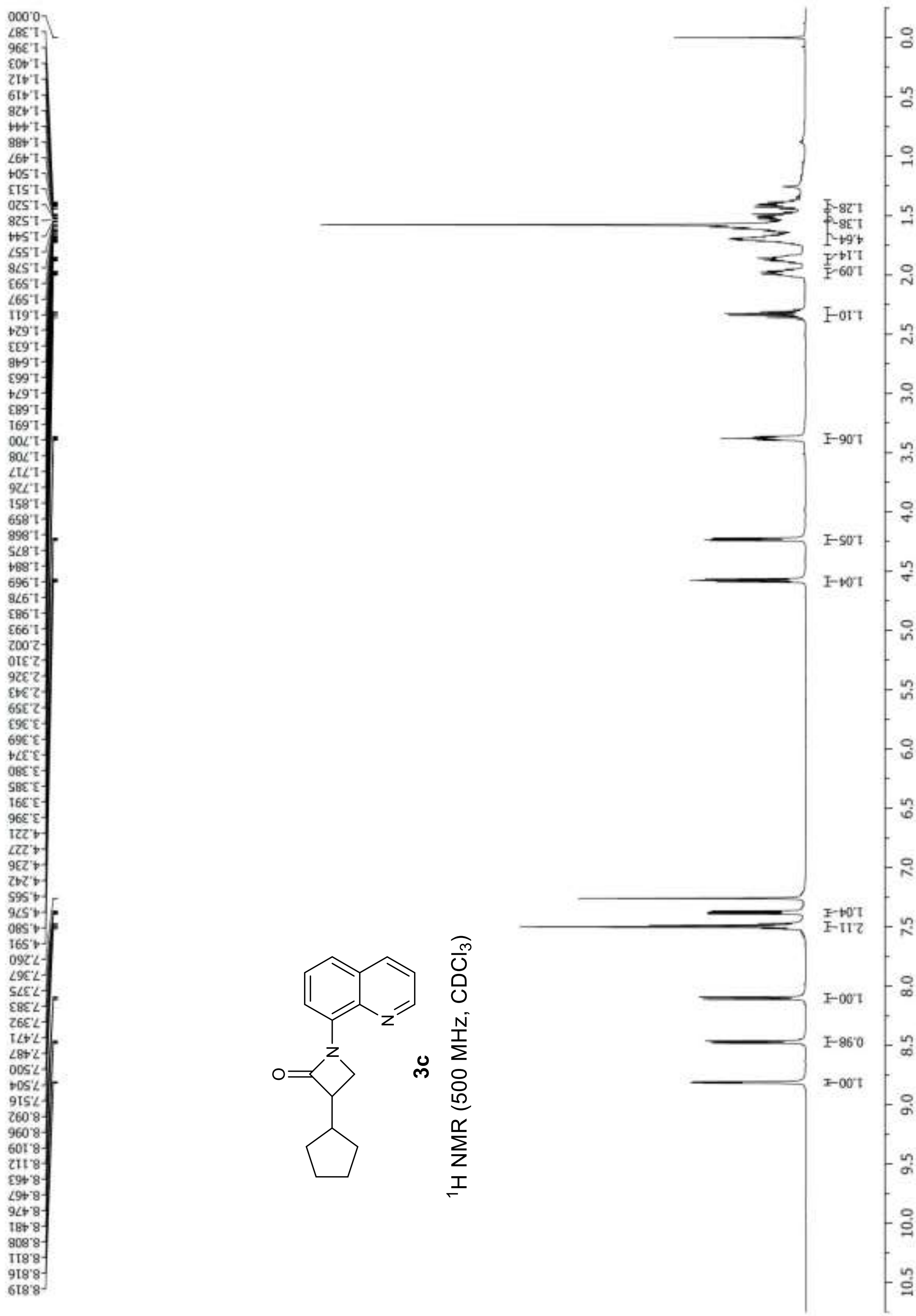

Supplementary Figure 83. ^{1}H NMR (500 MHz, $CDCl_3$) spectrum for 3c.

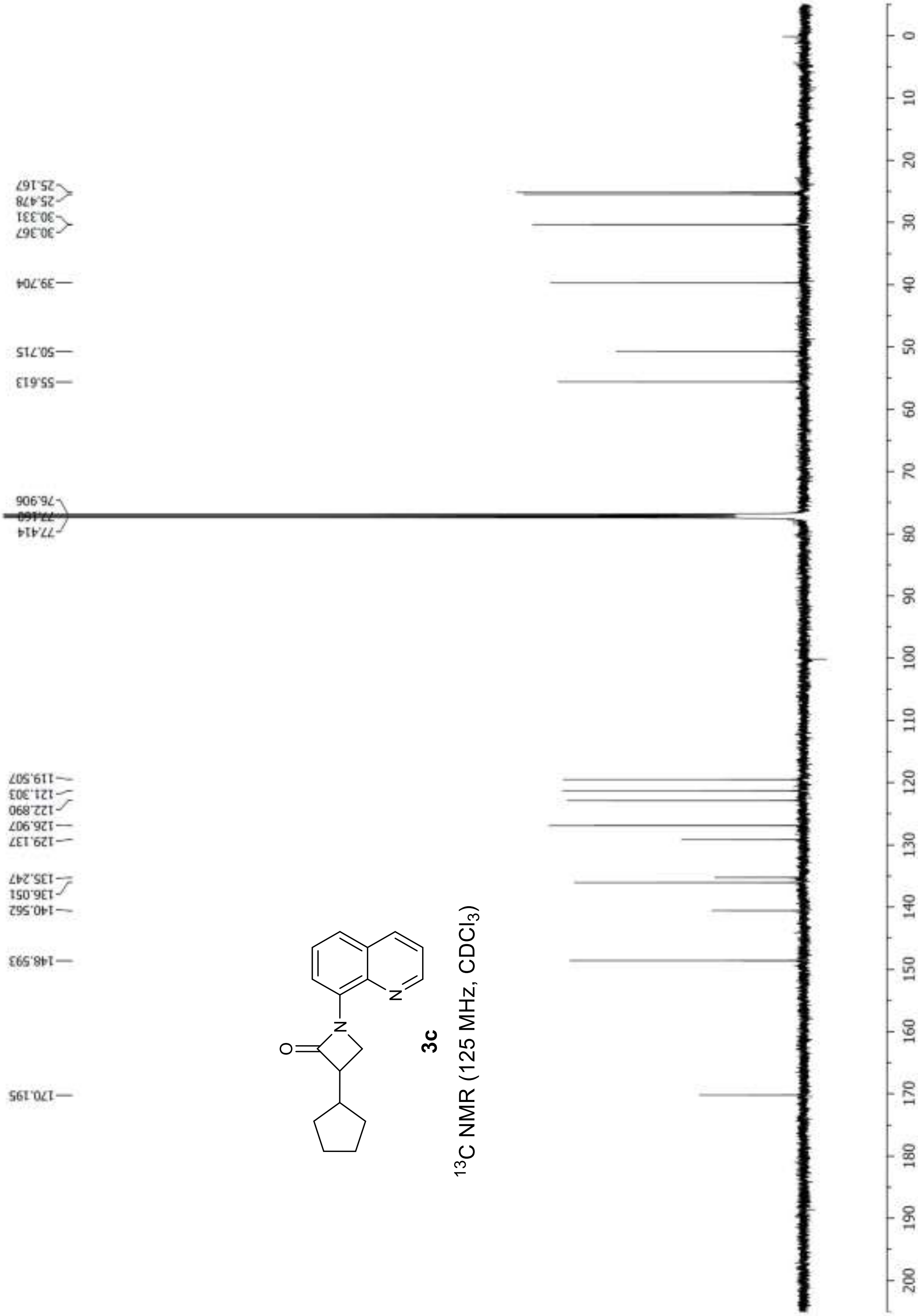

Supplementary Figure 84. ^{13}C NMR (125 MHz, $CDCl_3$) spectrum for 3c.

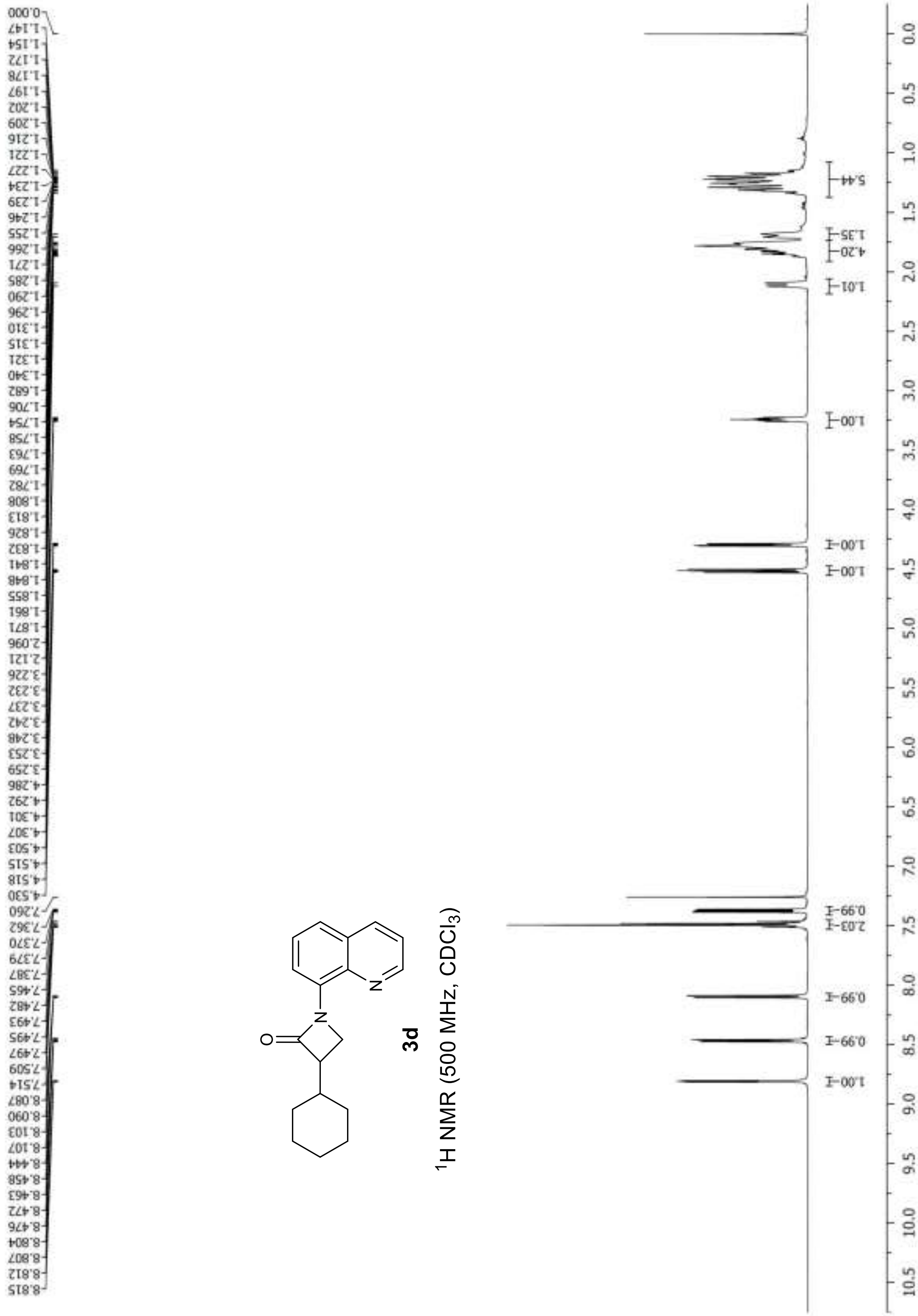

Supplementary Figure 85. ^{1}H NMR (500 MHz, CDCl$_3$) spectrum for 3d.

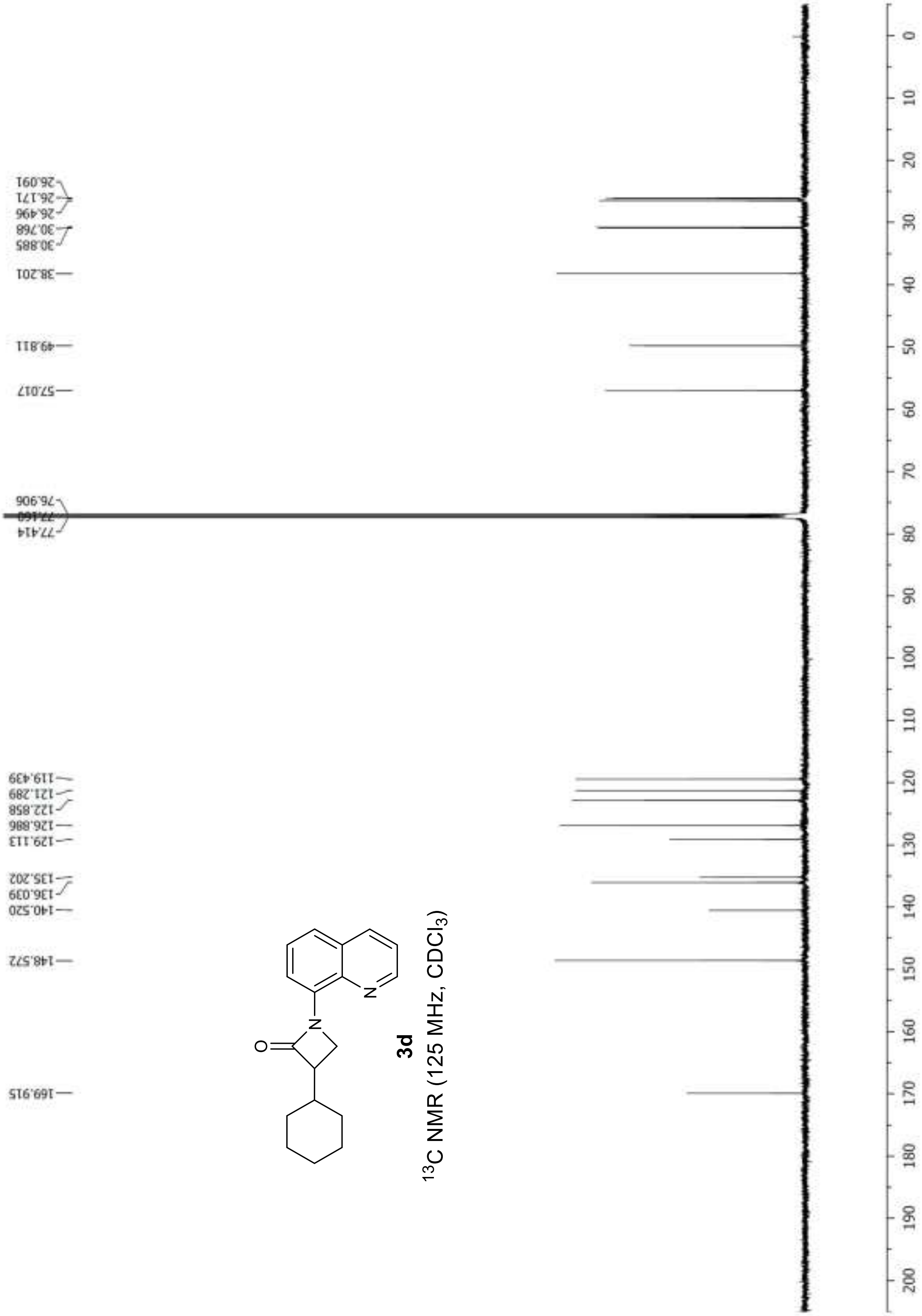

Supplementary Figure 86. ^{13}C NMR (125 MHz, $CDCl_3$) spectrum for 3d.

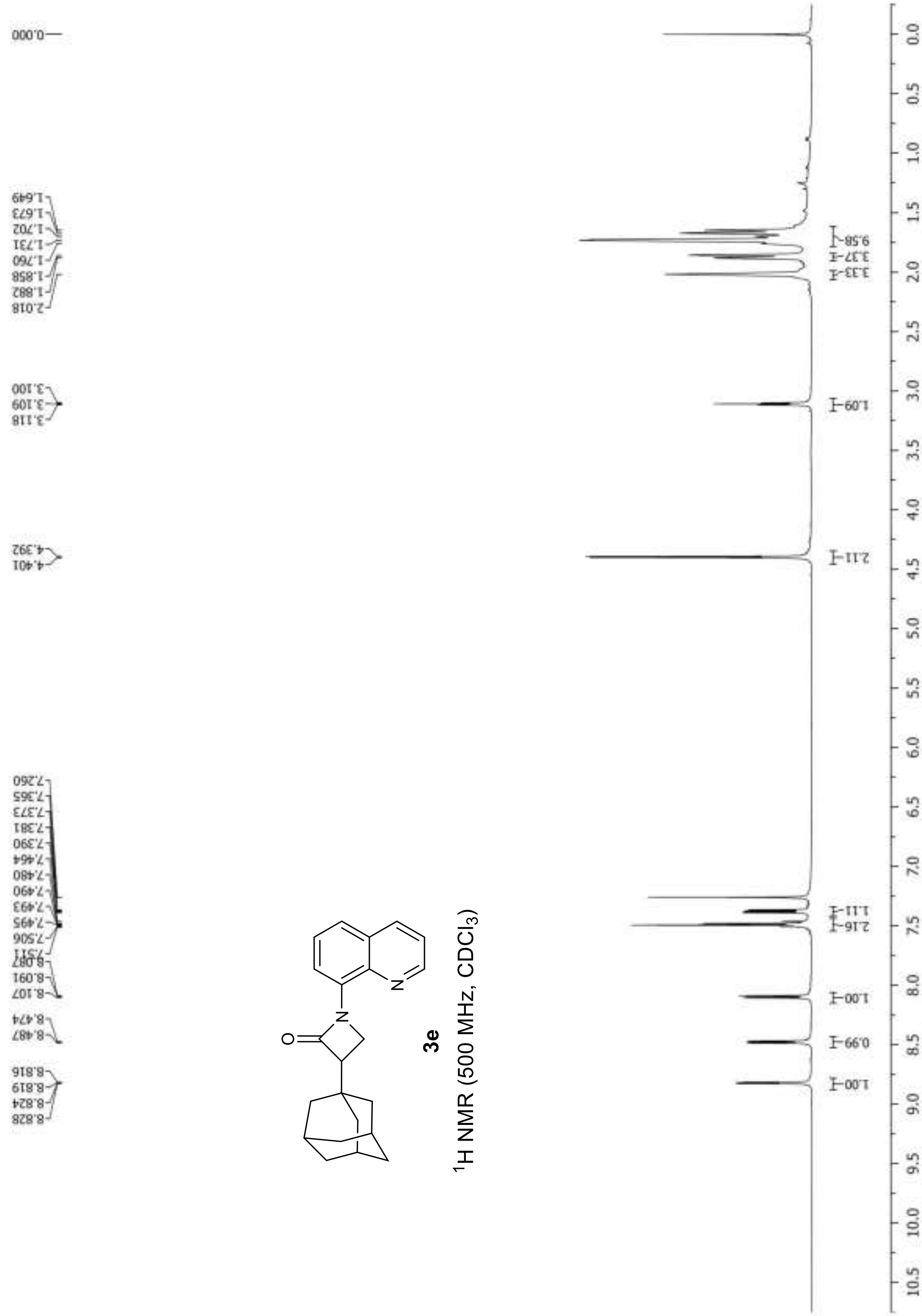

Supplementary Figure 87. ^{1}H NMR (500 MHz, $CDCl_3$) spectrum for 3e.

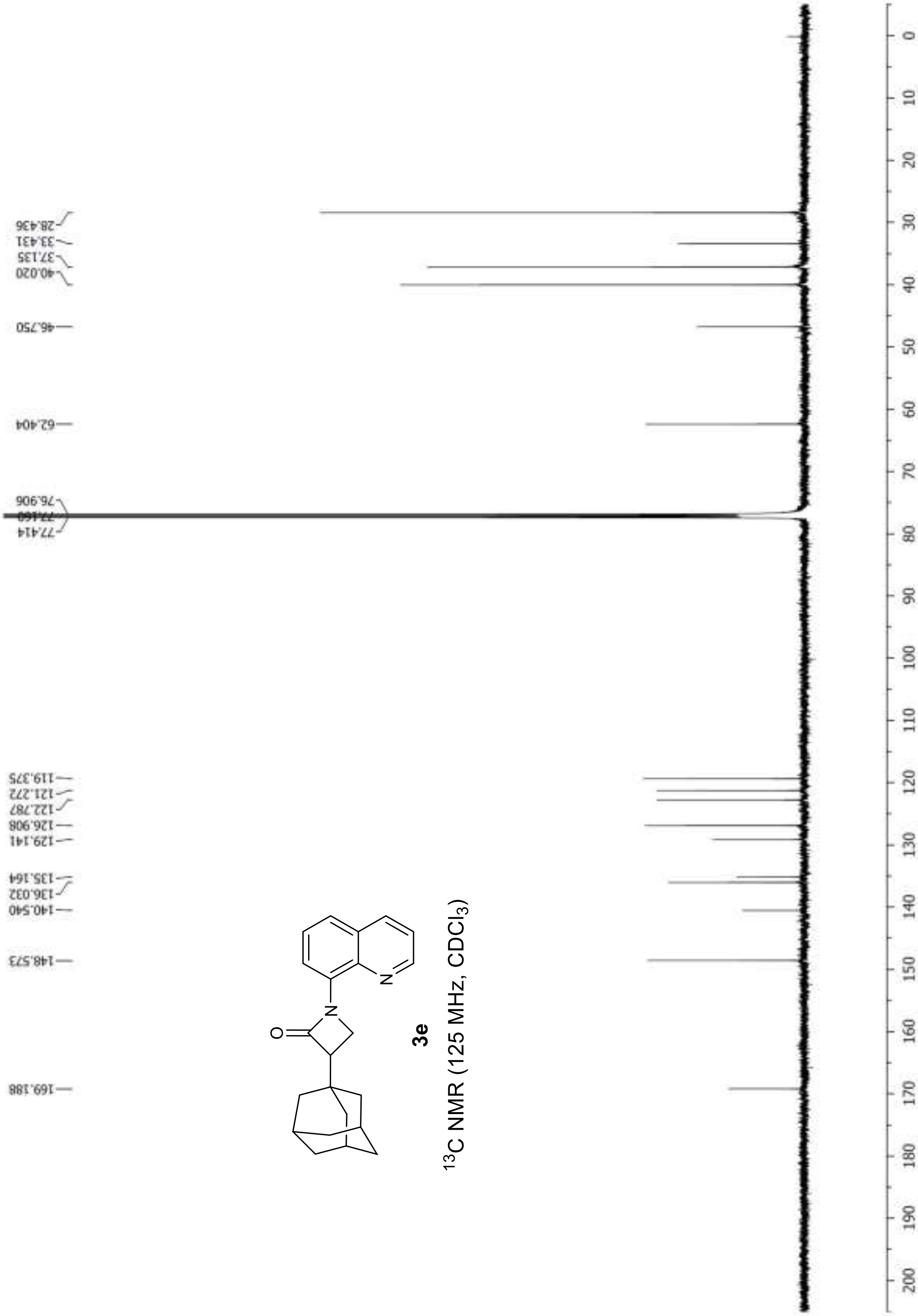

Supplementary Figure 88. ^{13}C NMR (125 MHz, $CDCl_3$) spectrum for 3e.

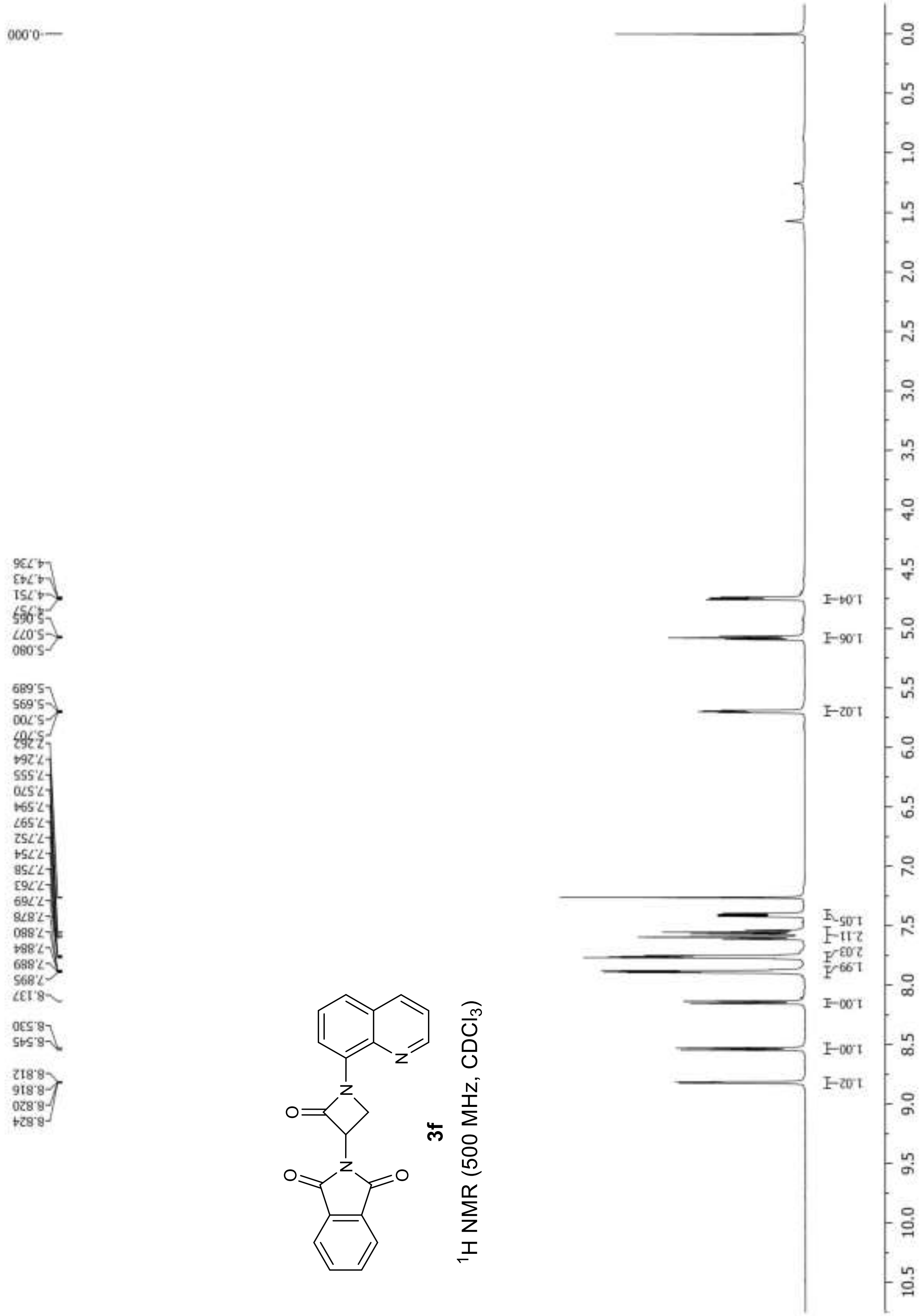

Supplementary Figure 89. ^{1}H NMR (500 MHz, $CDCl_3$) spectrum for 3f.

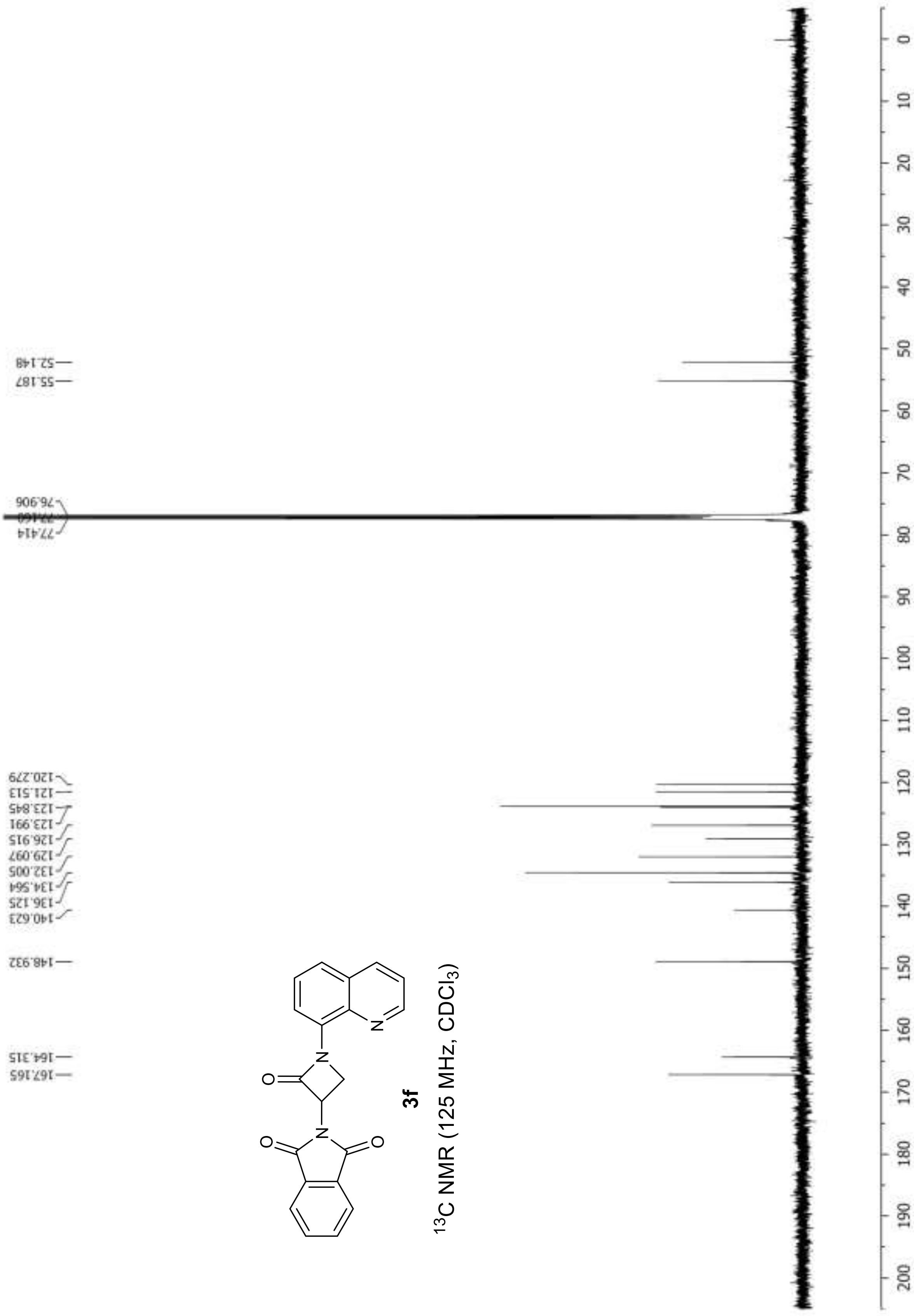

Supplementary Figure 90. ^{13}**C NMR (125 MHz, CDCl**$_3$**) spectrum for 3f.**

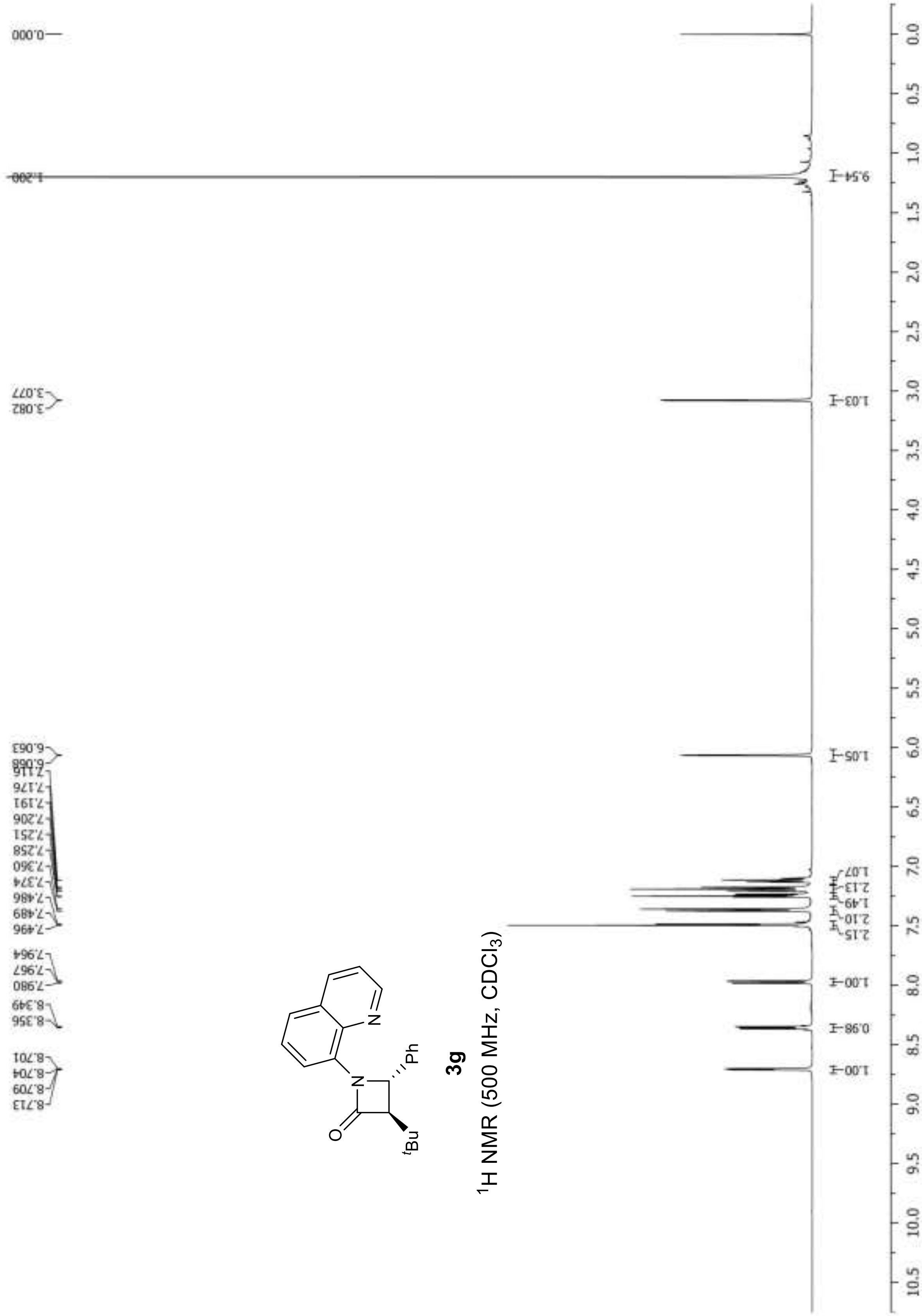

Supplementary Figure 91. ^{1}H NMR (500 MHz, $CDCl_3$) spectrum for 3g.

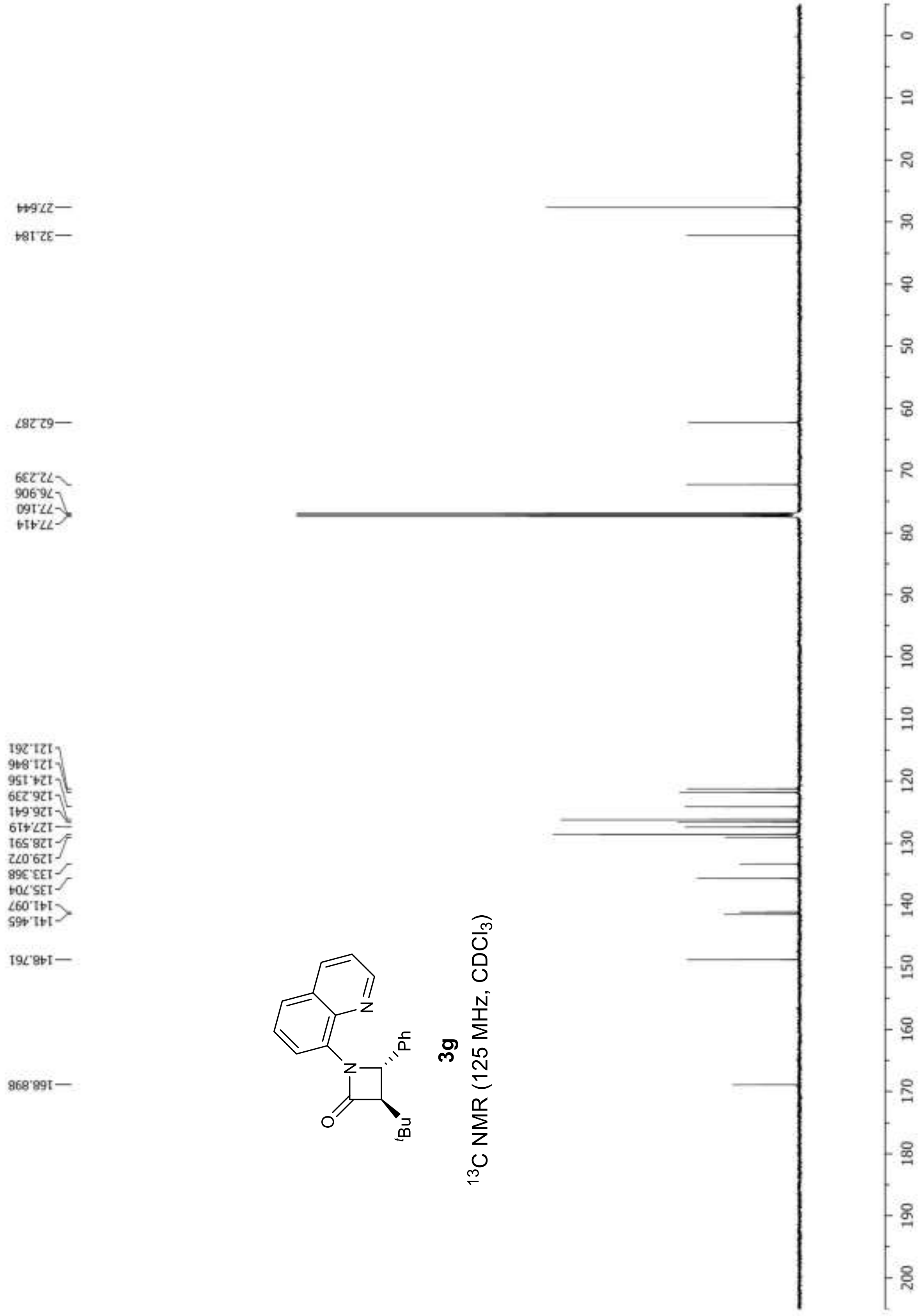

Supplementary Figure 92. ^{13}C NMR (125 MHz, $CDCl_3$) spectrum for 3g.

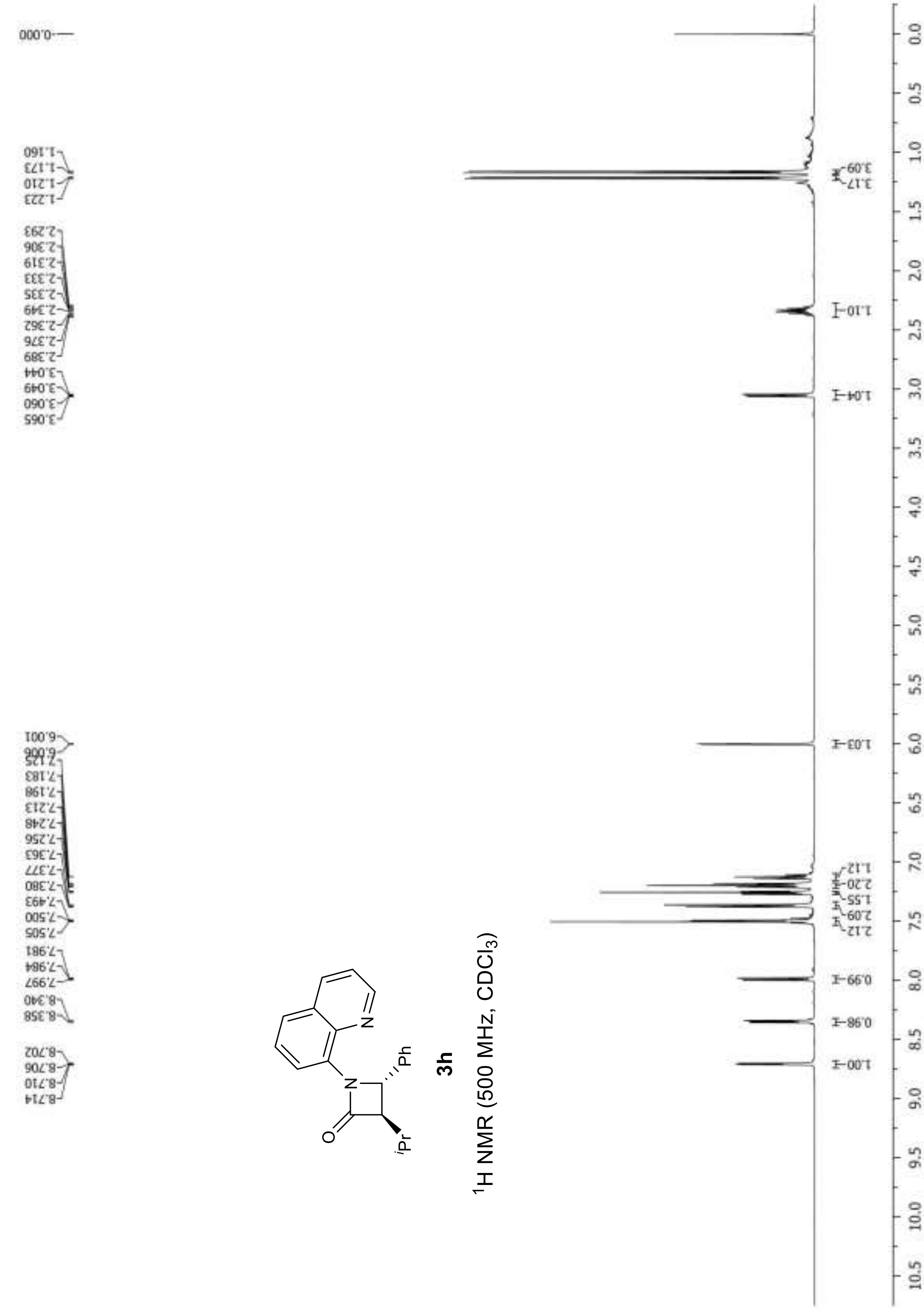

Supplementary Figure 93. ^{1}H NMR (500 MHz, CDCl$_3$) spectrum for 3h.

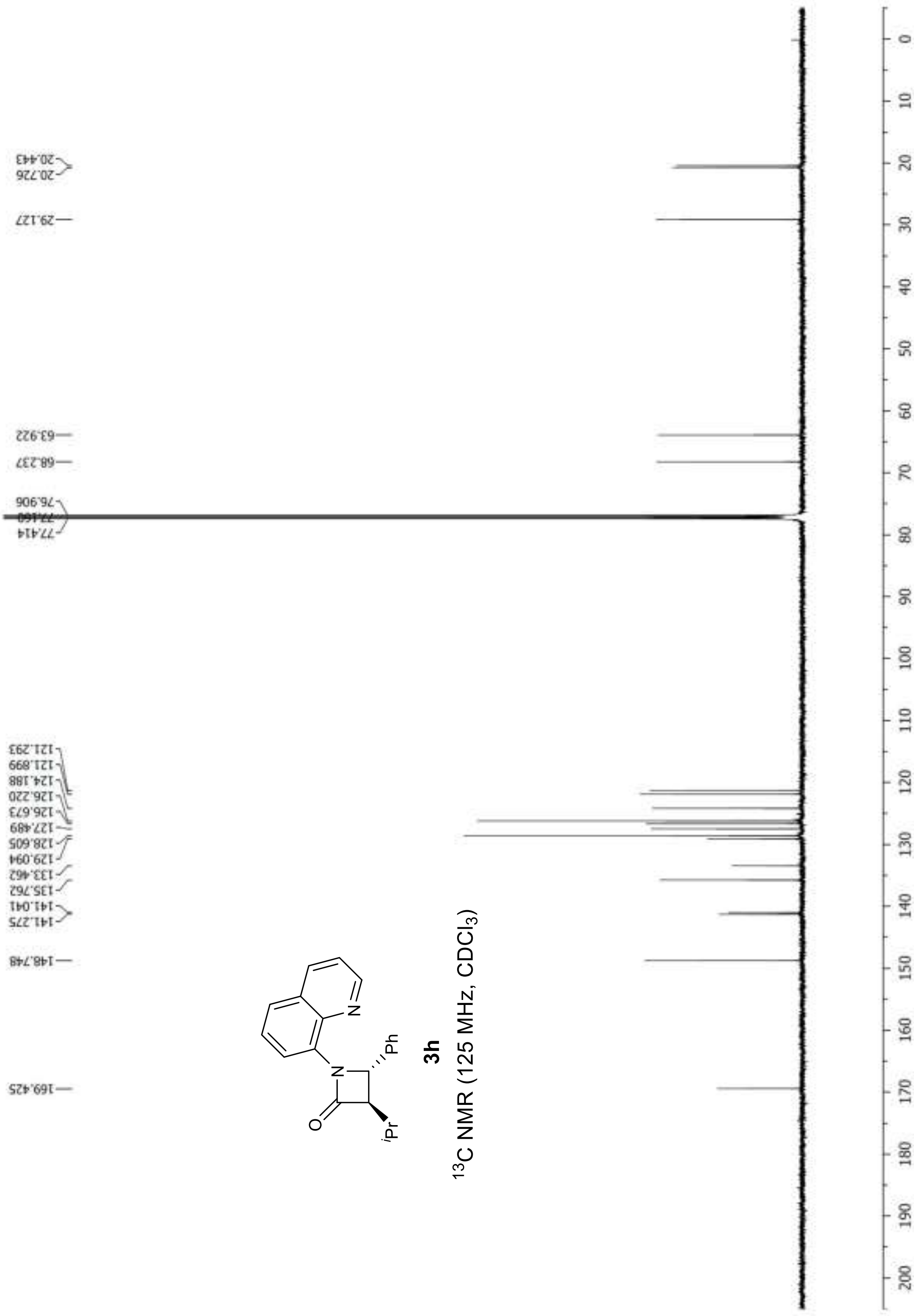

Supplementary Figure 94. ^{13}C NMR (125 MHz, $CDCl_3$) spectrum for 3h.

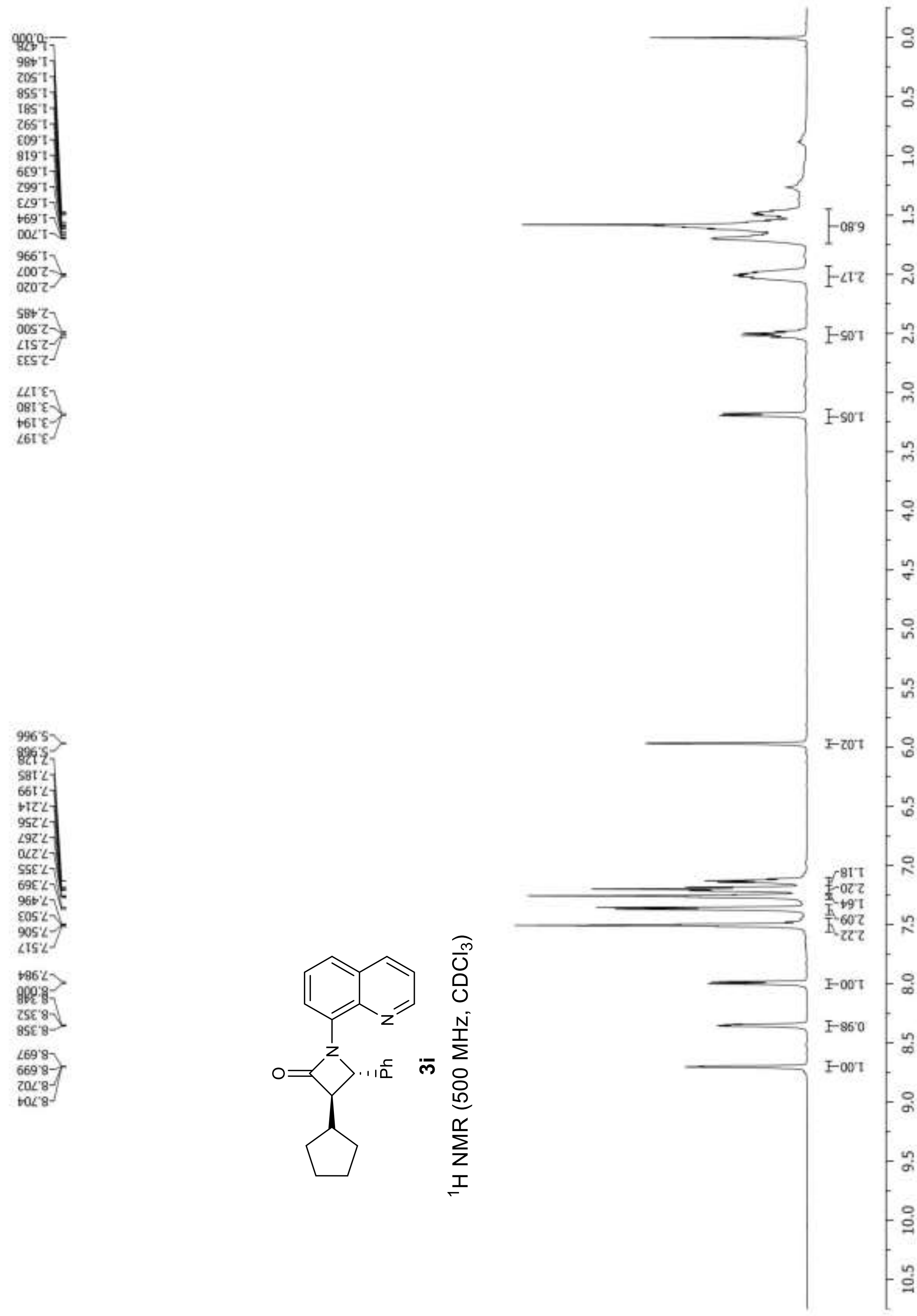

Supplementary Figure 95. ^{1}H NMR (500 MHz, $CDCl_3$) spectrum for 3i.

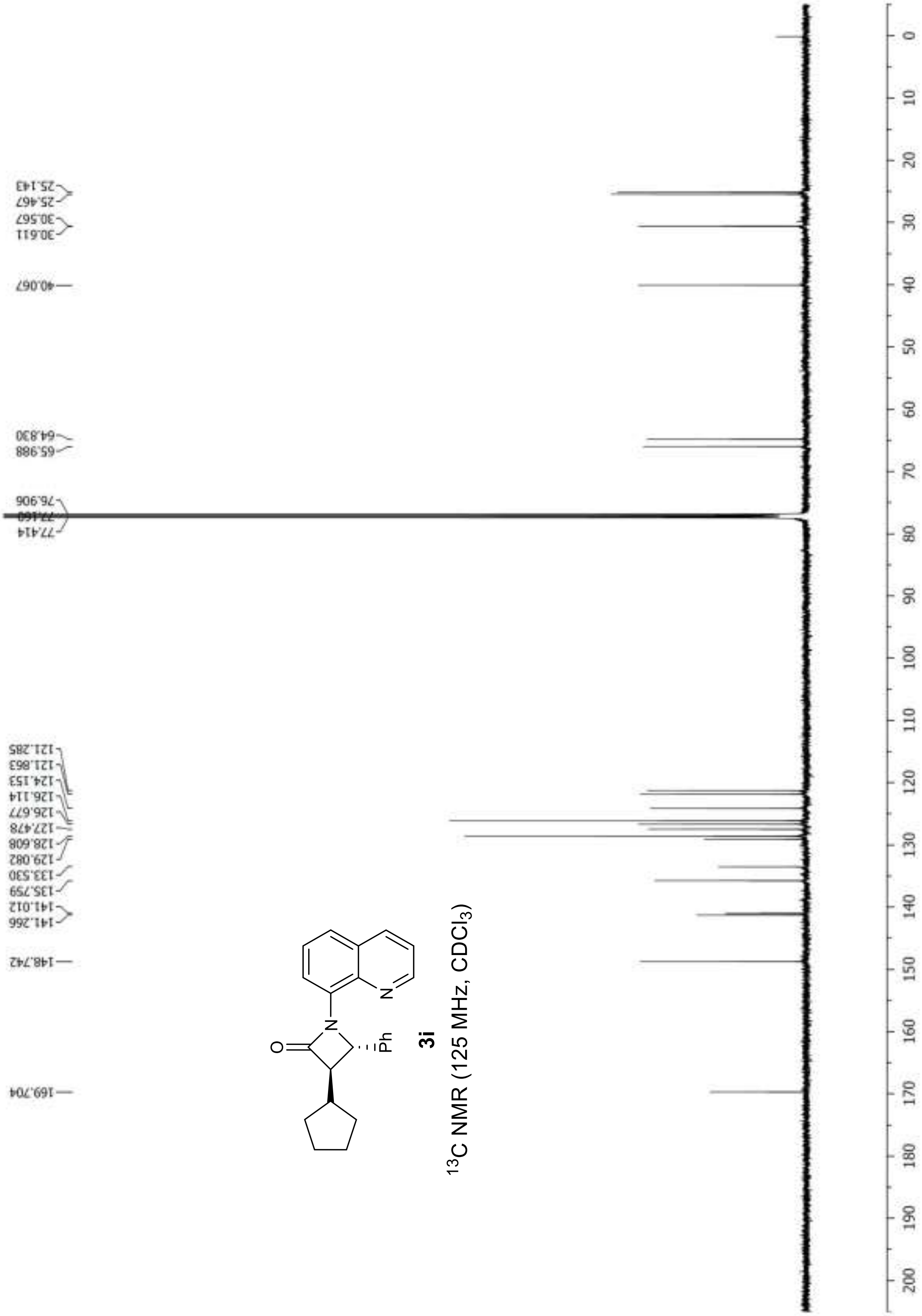

Supplementary Figure 96. ^{13}**C NMR (125 MHz, CDCl$_3$) spectrum for 3i.**

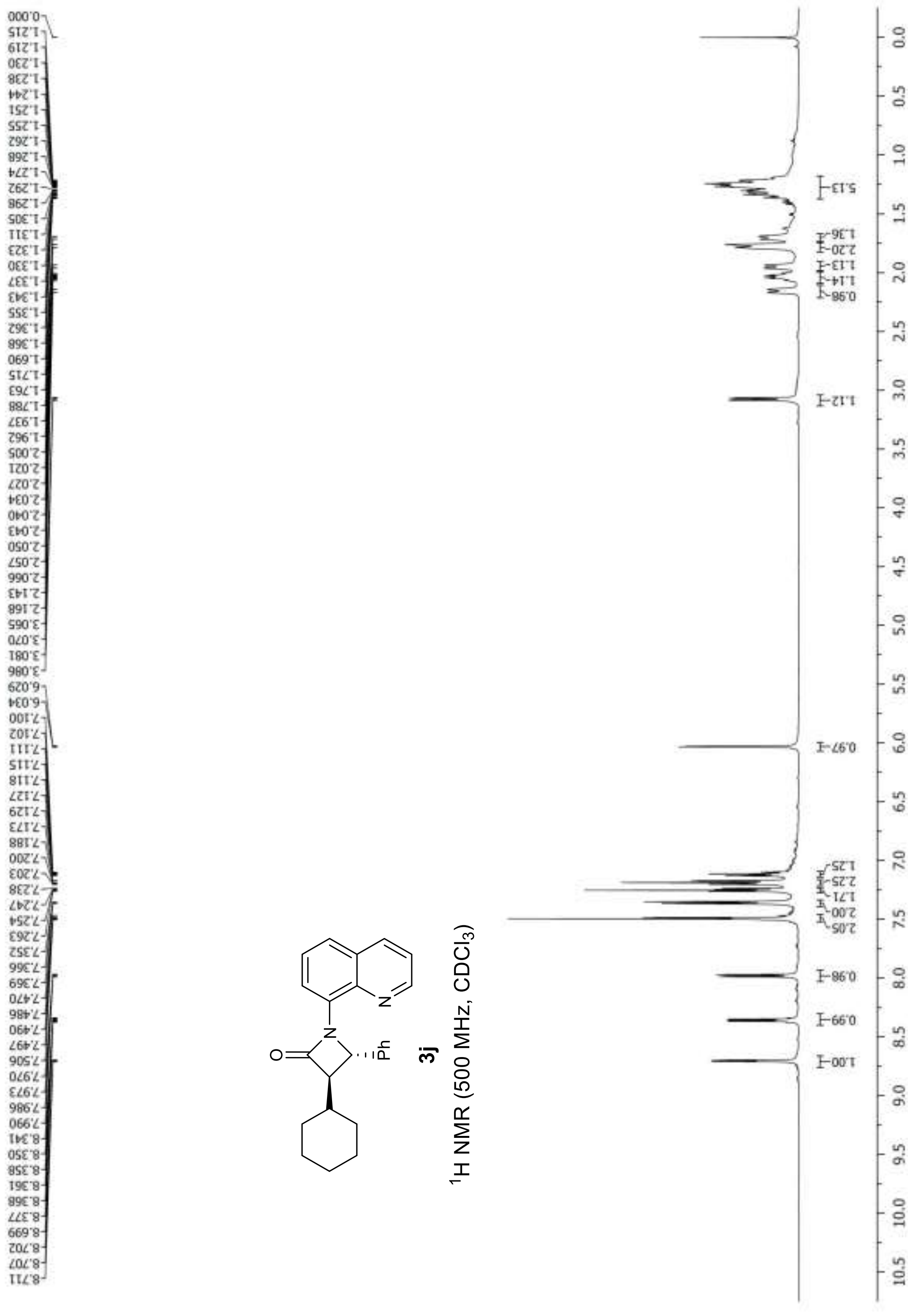

Supplementary Figure 97. ^{1}H NMR (500 MHz, $CDCl_3$) spectrum for 3j.

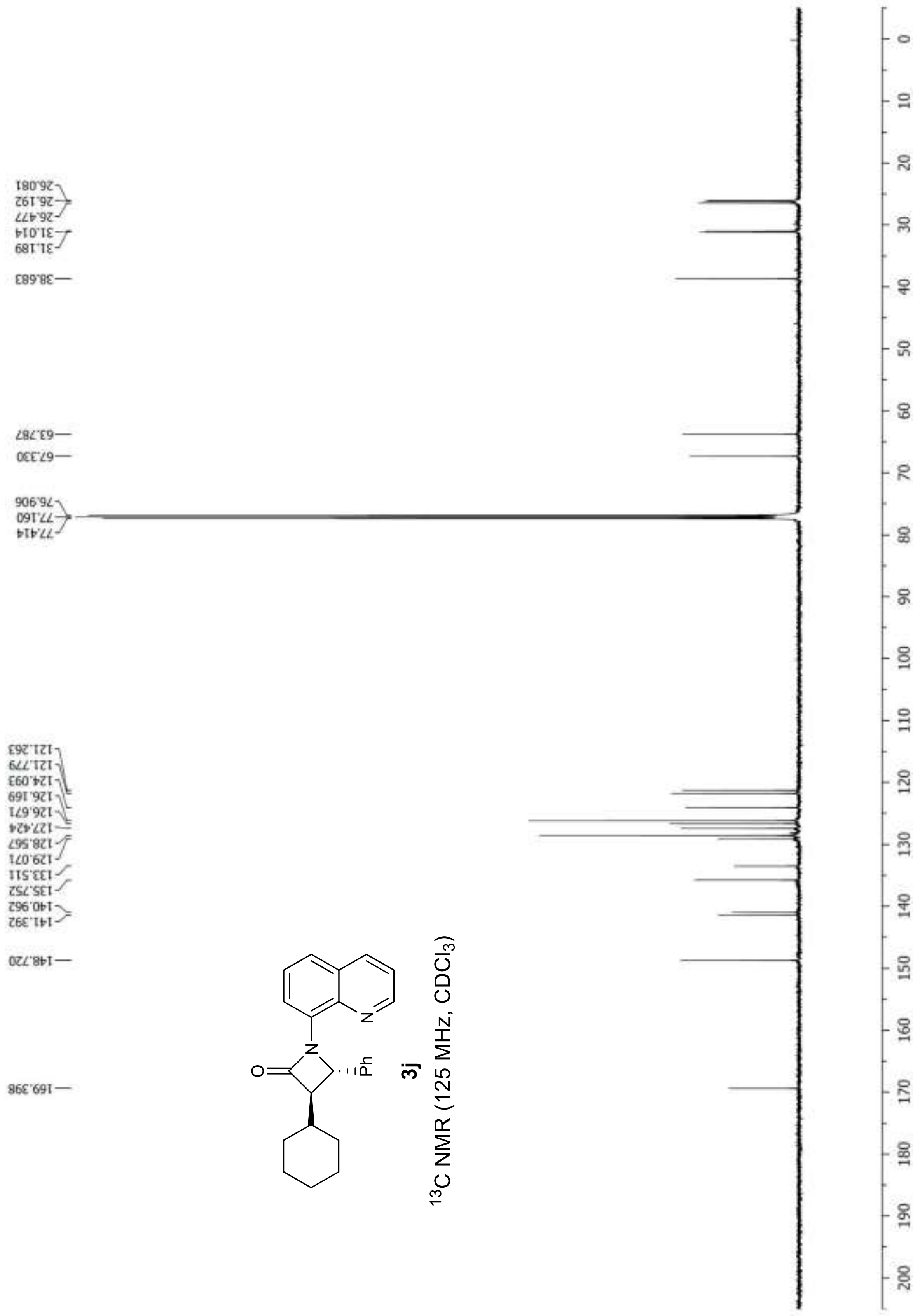

Supplementary Figure 98. ^{13}C NMR (125 MHz, $CDCl_3$) spectrum for 3j.

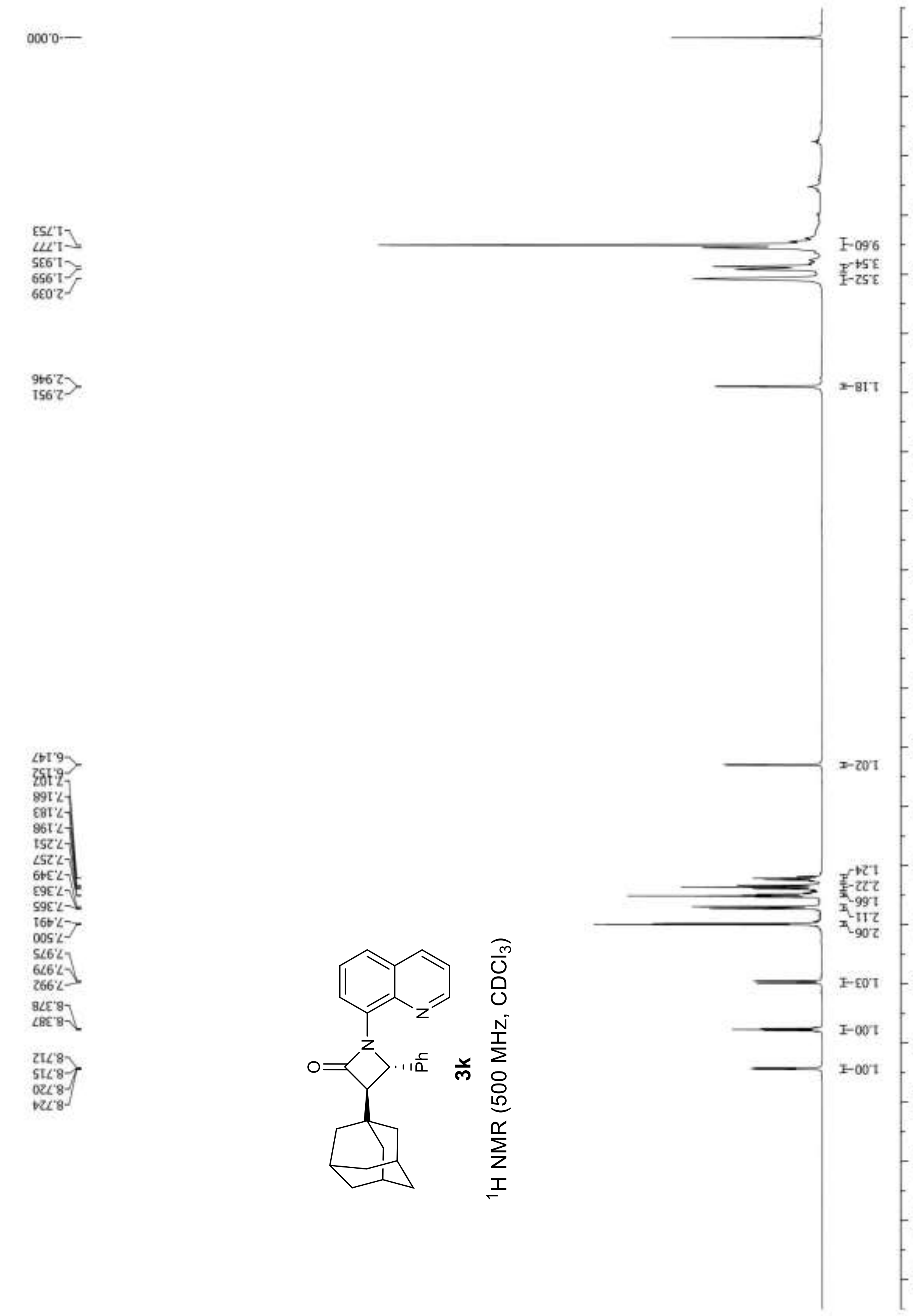

Supplementary Figure 99. ^{1}H NMR (500 MHz, $CDCl_3$) spectrum for 3k.

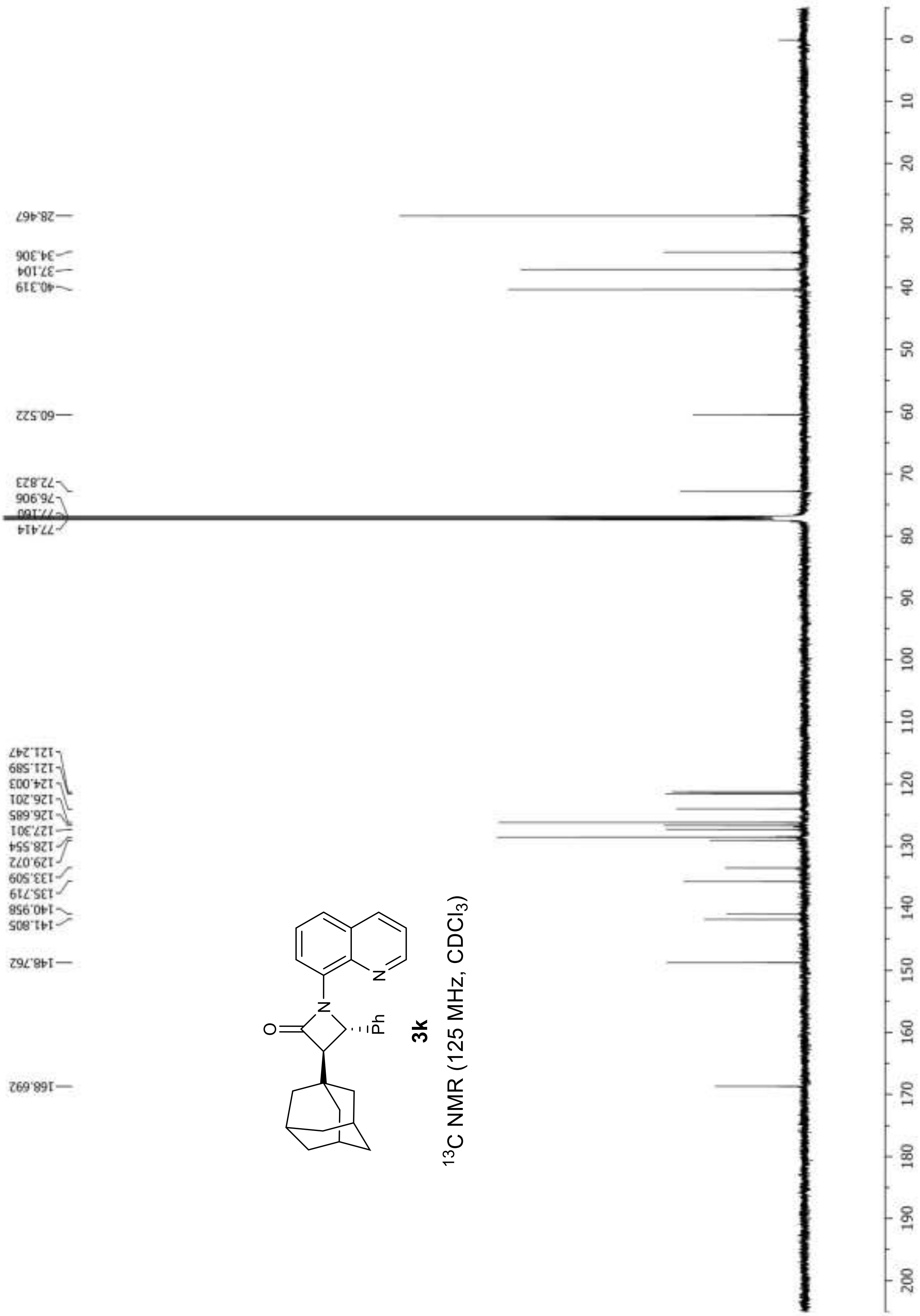

Supplementary Figure 100. ^{13}C NMR (125 MHz, $CDCl_3$) spectrum for 3k.

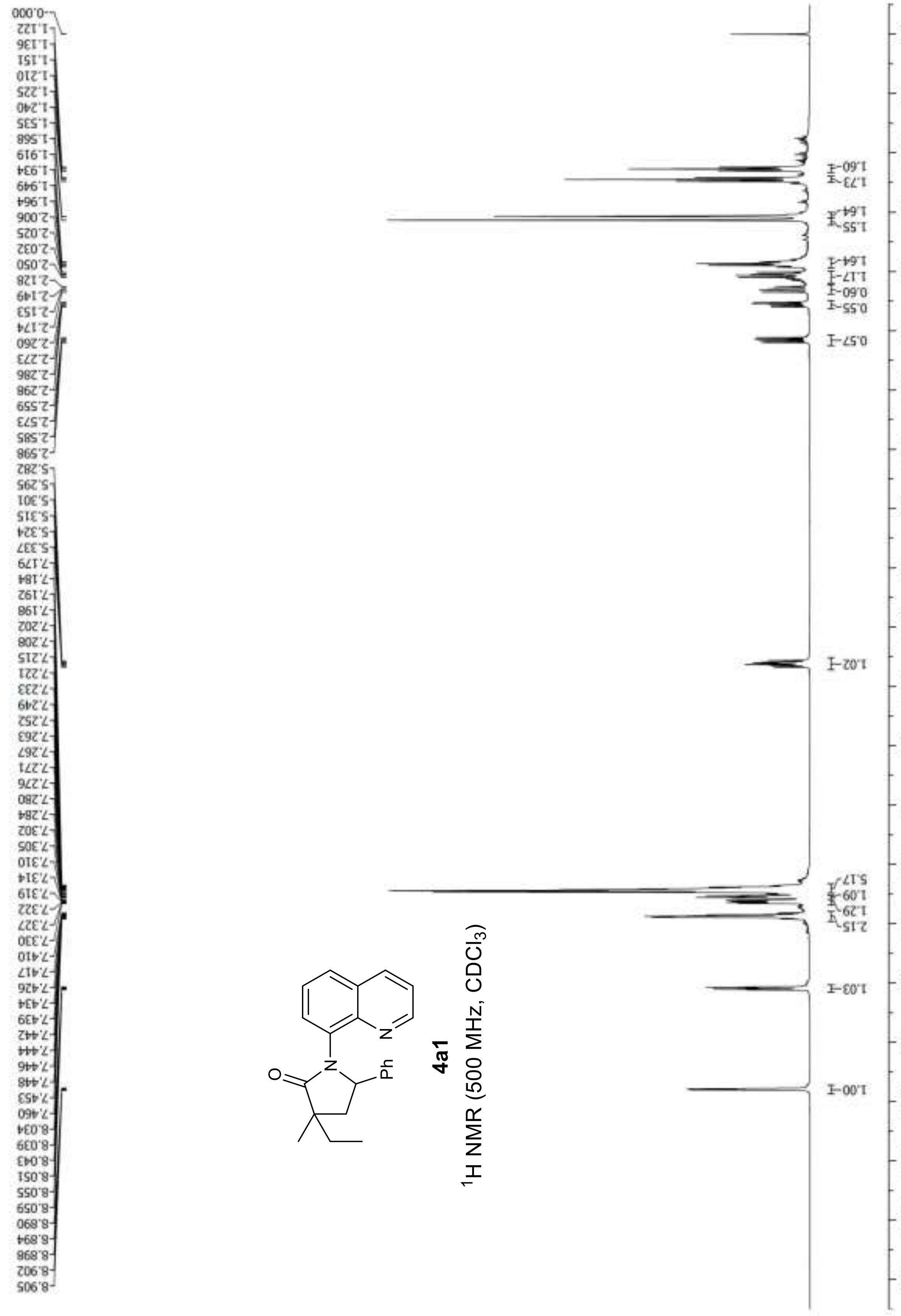

Supplementary Figure 101. ^{1}H NMR (500 MHz, $CDCl_3$) spectrum for 4a1.

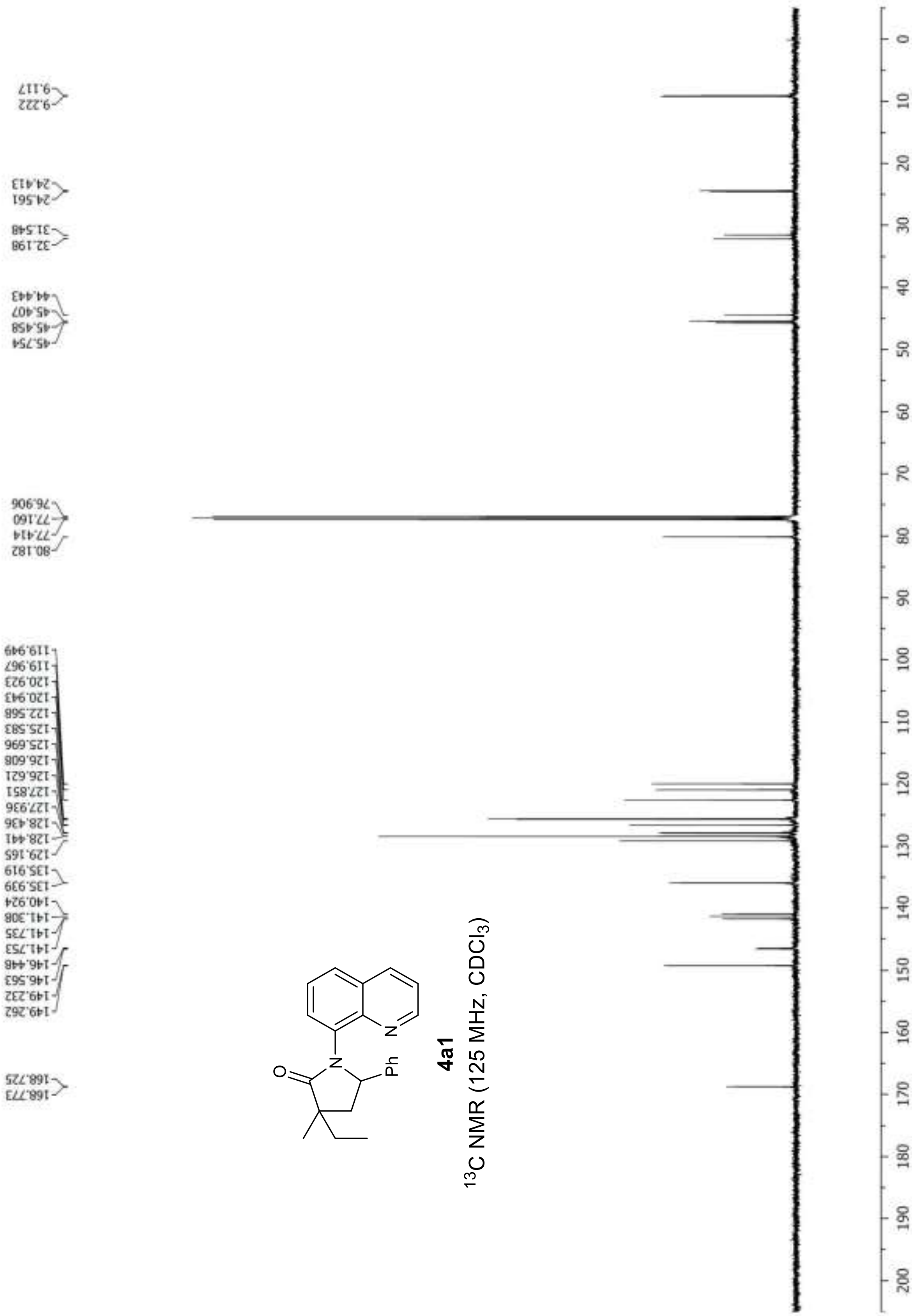

Supplementary Figure 102. ^{13}C NMR (125 MHz, $CDCl_3$) spectrum for 4a1.

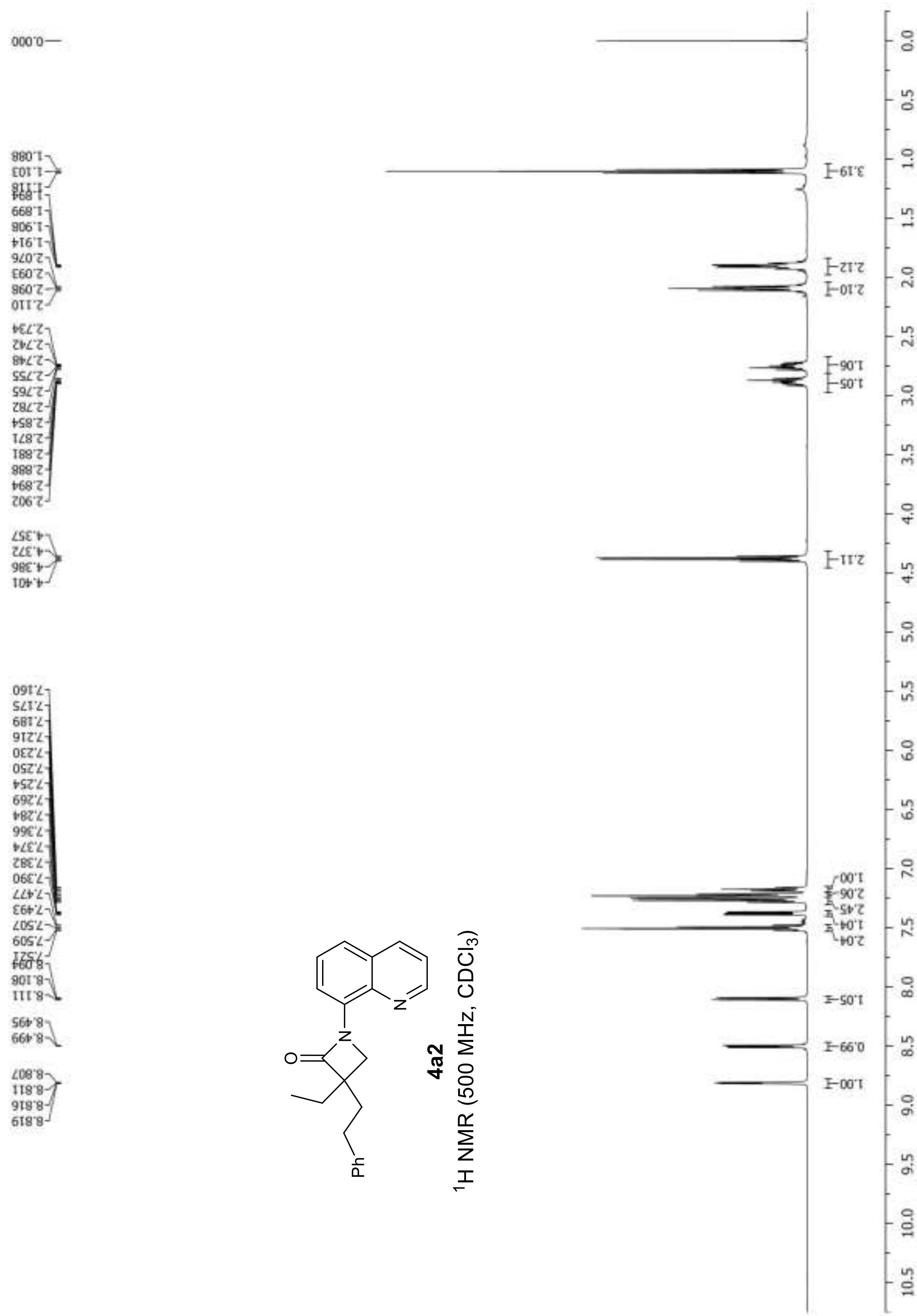

Supplementary Figure 103. ^{1}H NMR (500 MHz, $CDCl_3$) spectrum for 4a2.

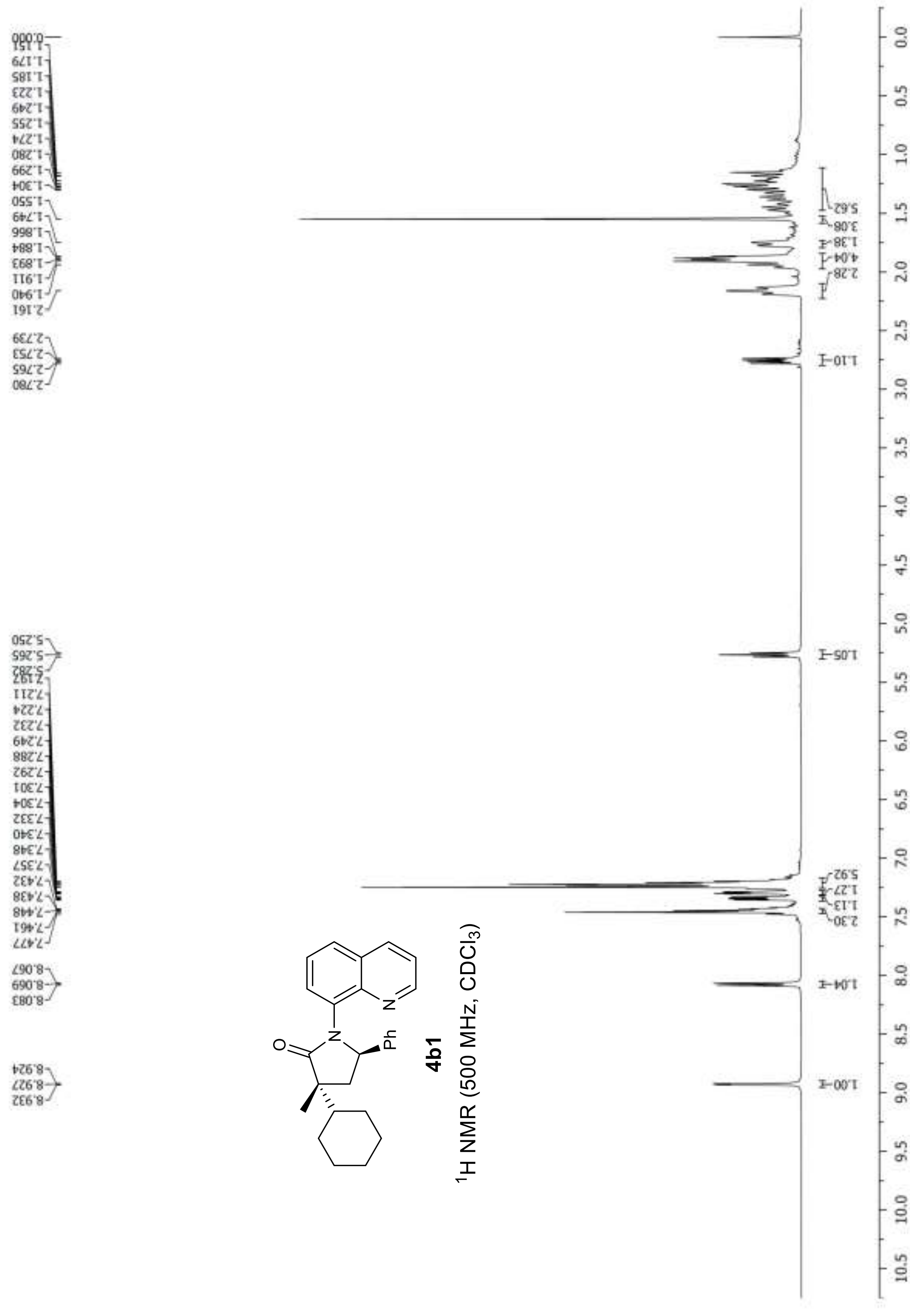

Supplementary Figure 104. ^{1}H NMR (500 MHz, $CDCl_3$) spectrum for 4b1.

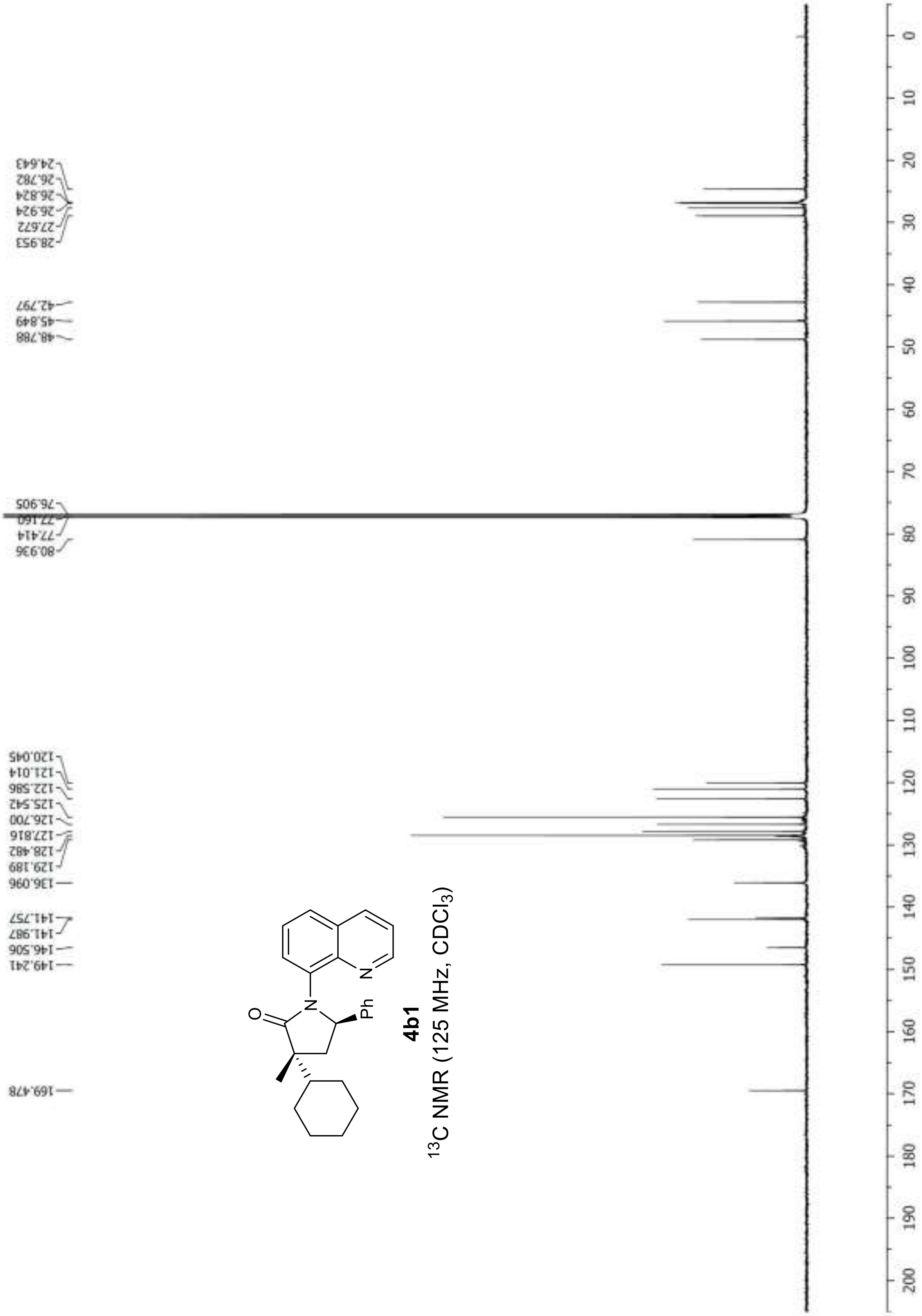

Supplementary Figure 105. ^{13}**C NMR (125 MHz, CDCl$_3$) spectrum for 4b1.**

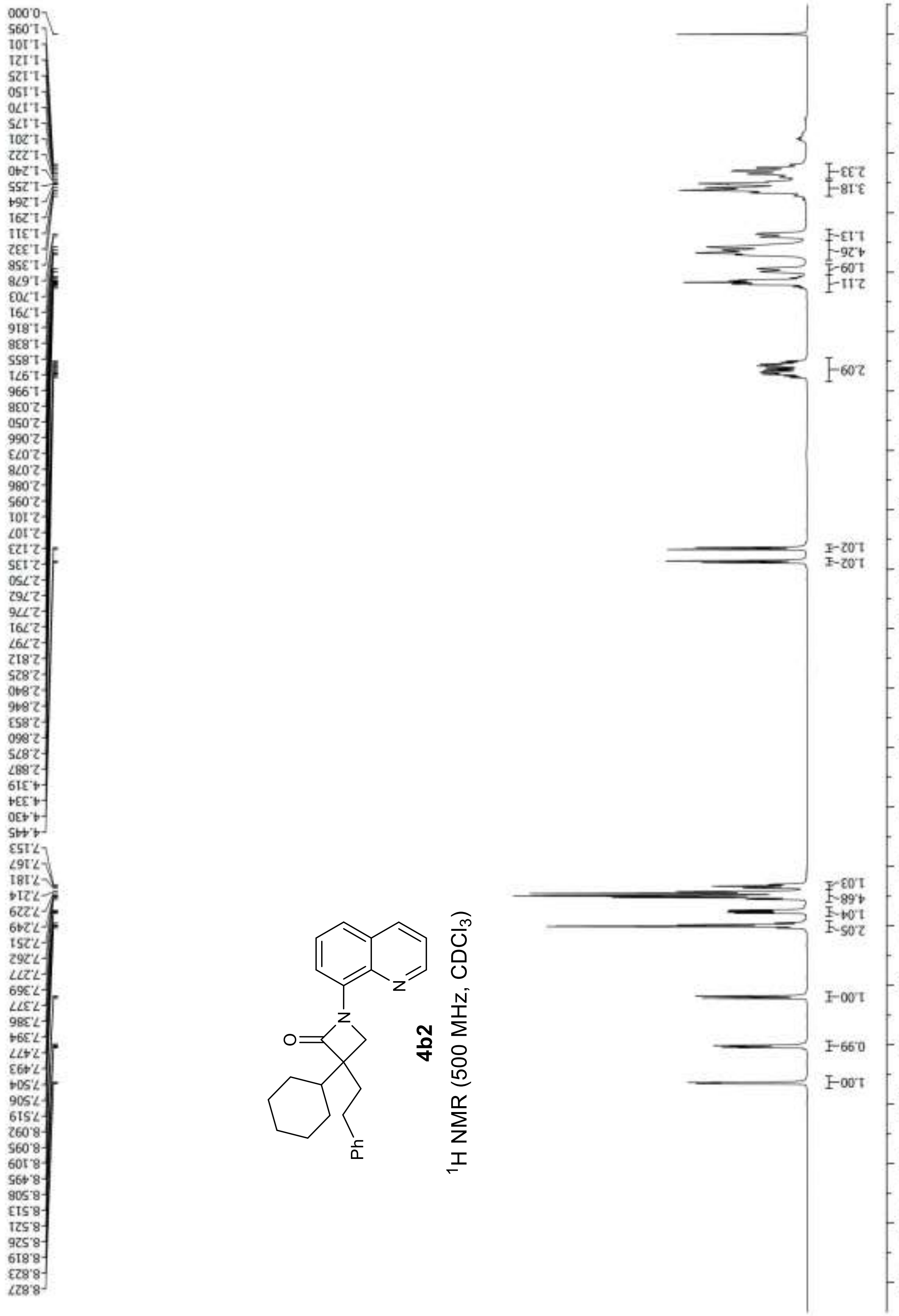

Supplementary Figure 106. ^{1}H NMR (500 MHz, $CDCl_3$) spectrum for 4b2.

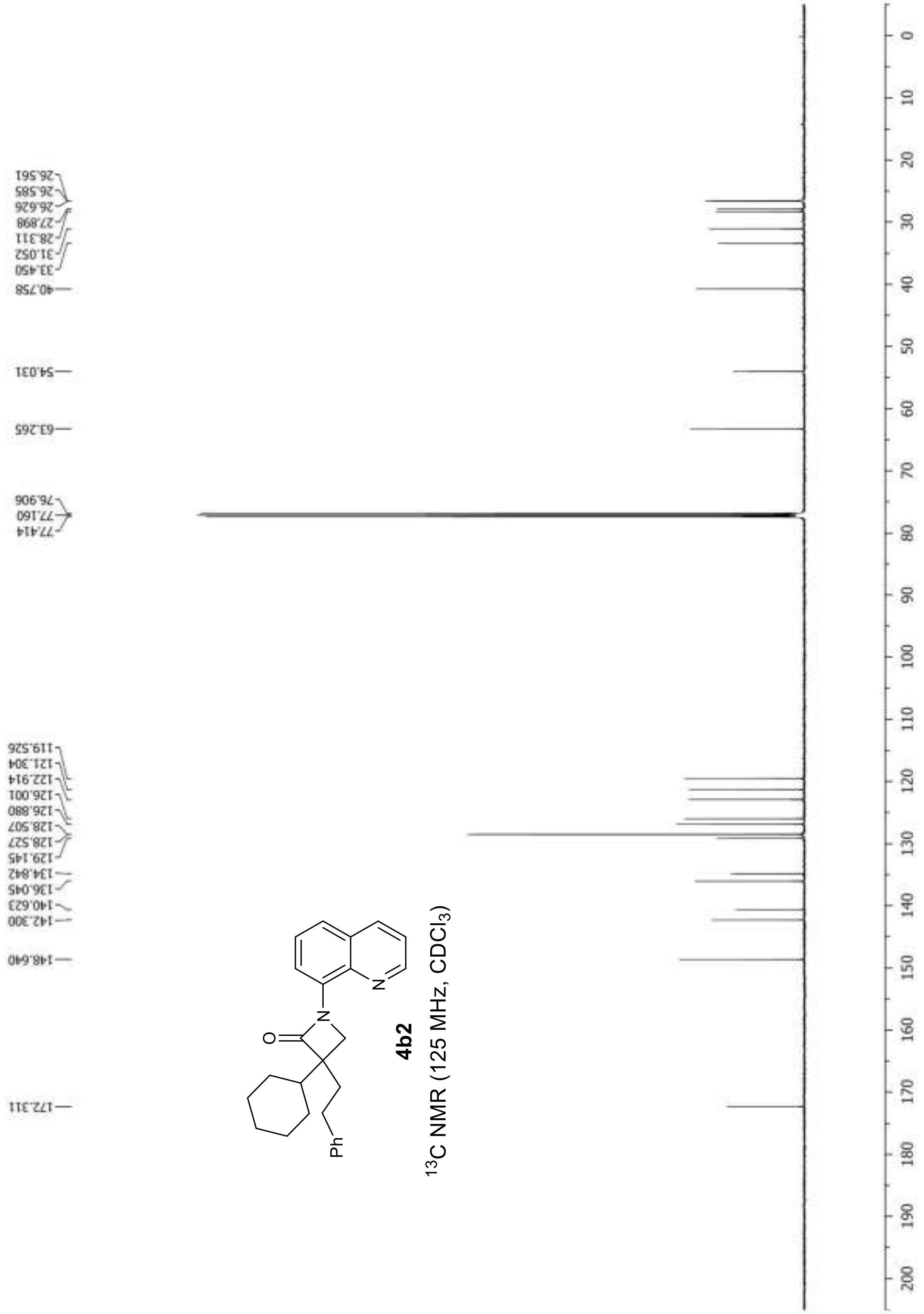

Supplementary Figure 107. ^{13}C NMR (125 MHz, CDCl$_3$) spectrum for 4b2.

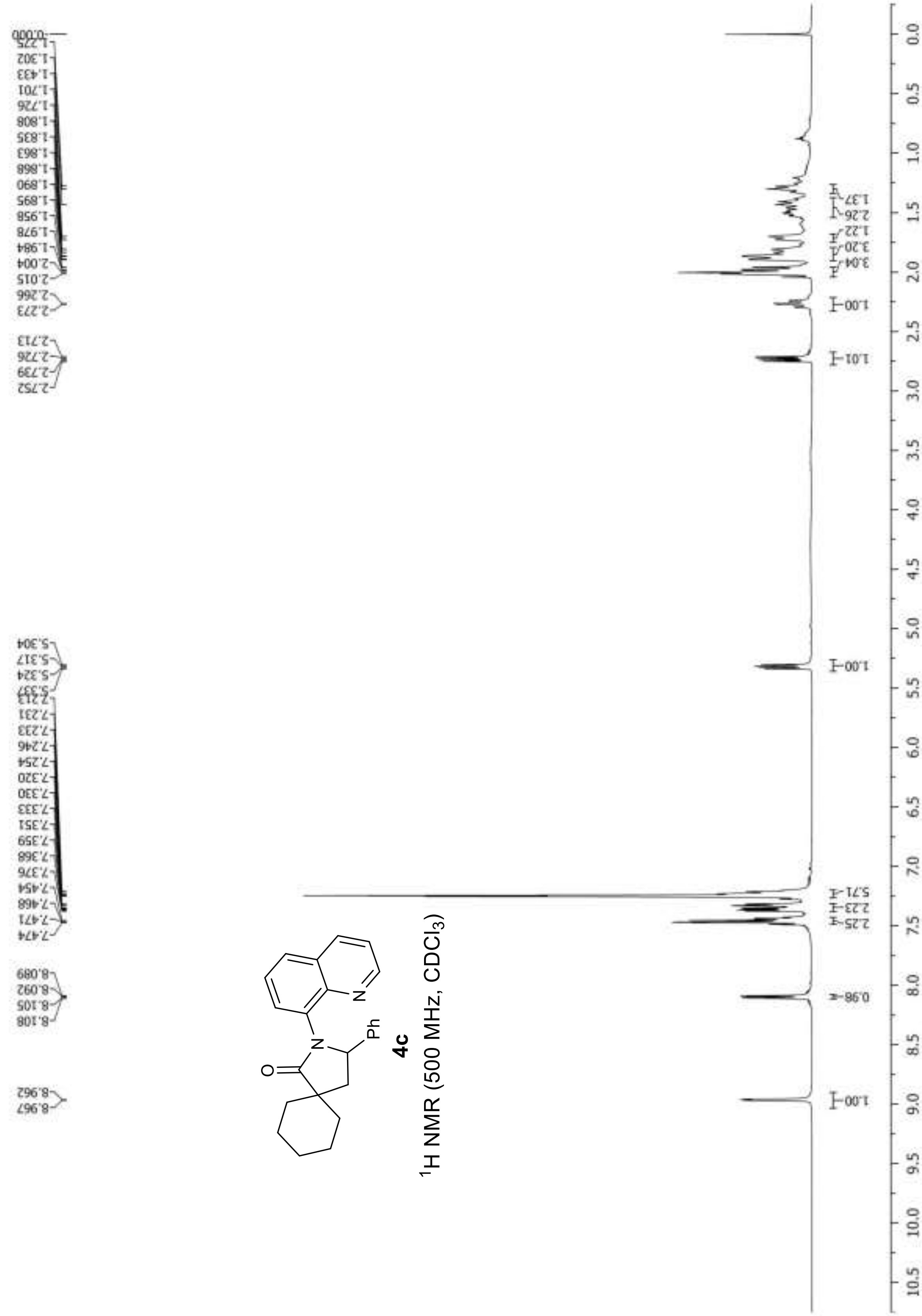

Supplementary Figure 108. ^{1}H NMR (500 MHz, $CDCl_3$) spectrum for 4c.

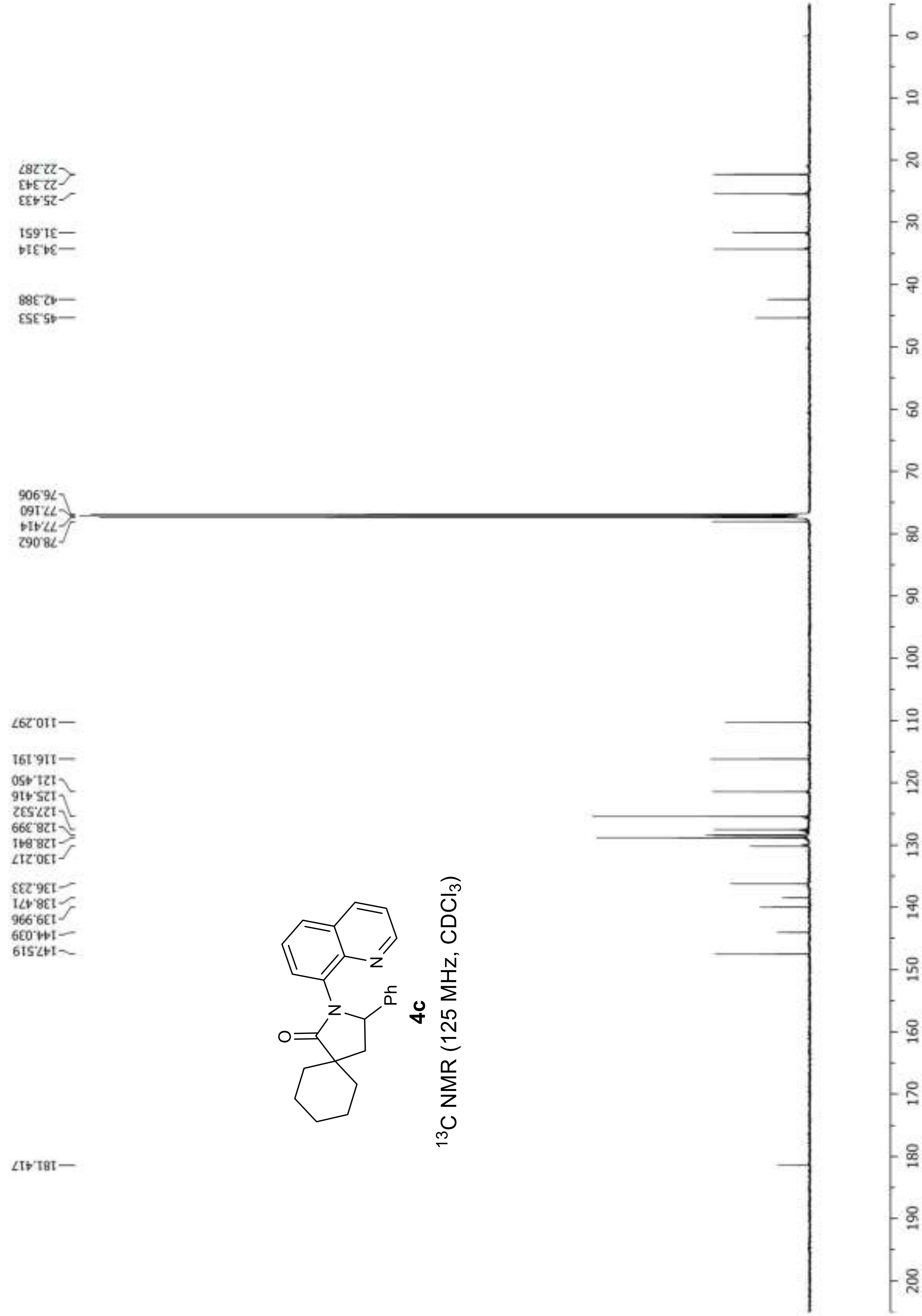

Supplementary Figure 109. ^{13}**C NMR (125 MHz, CDCl$_3$) spectrum for 4c.**

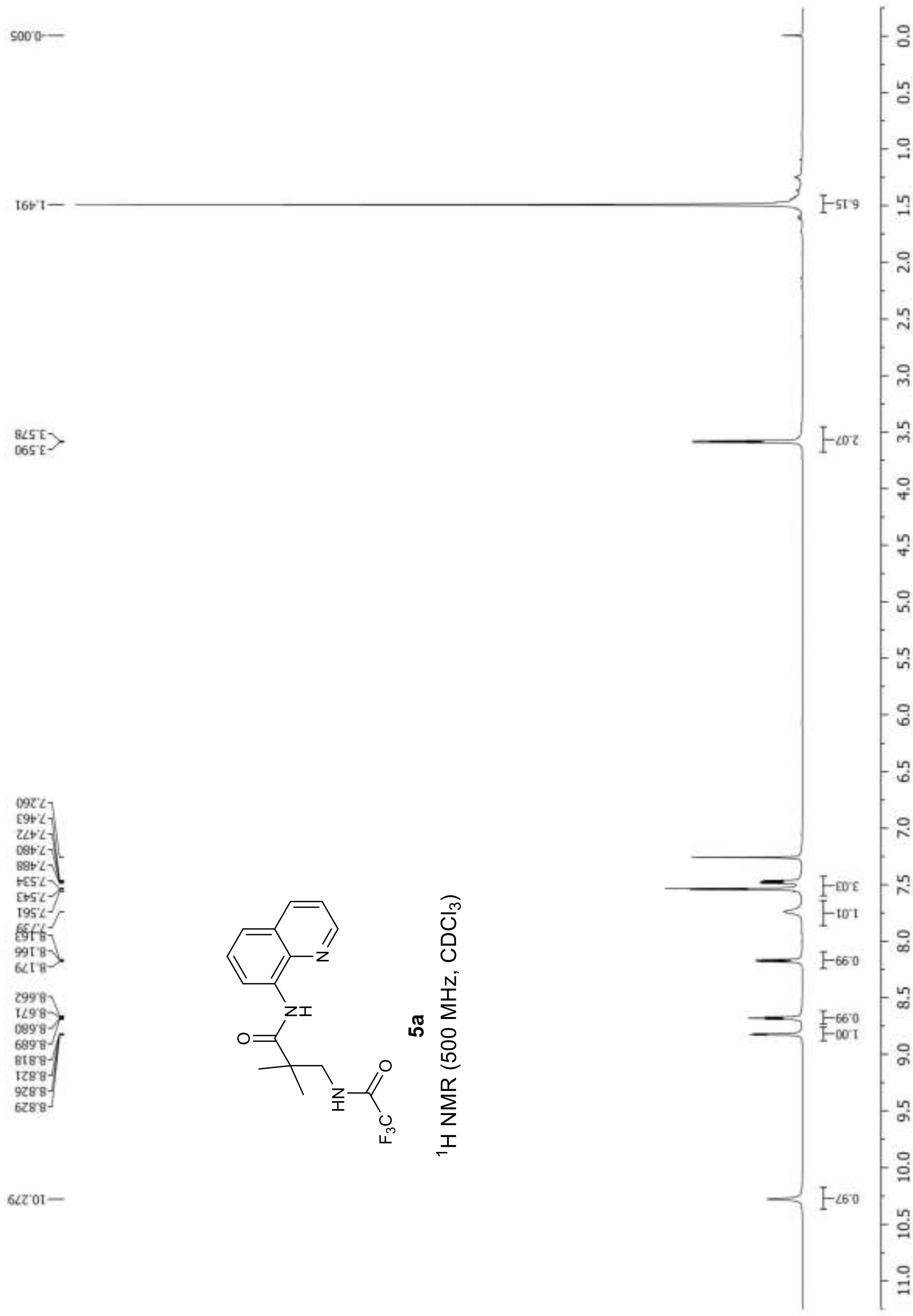

Supplementary Figure 110. ^{1}H NMR (500 MHz, $CDCl_3$) spectrum for 5a.

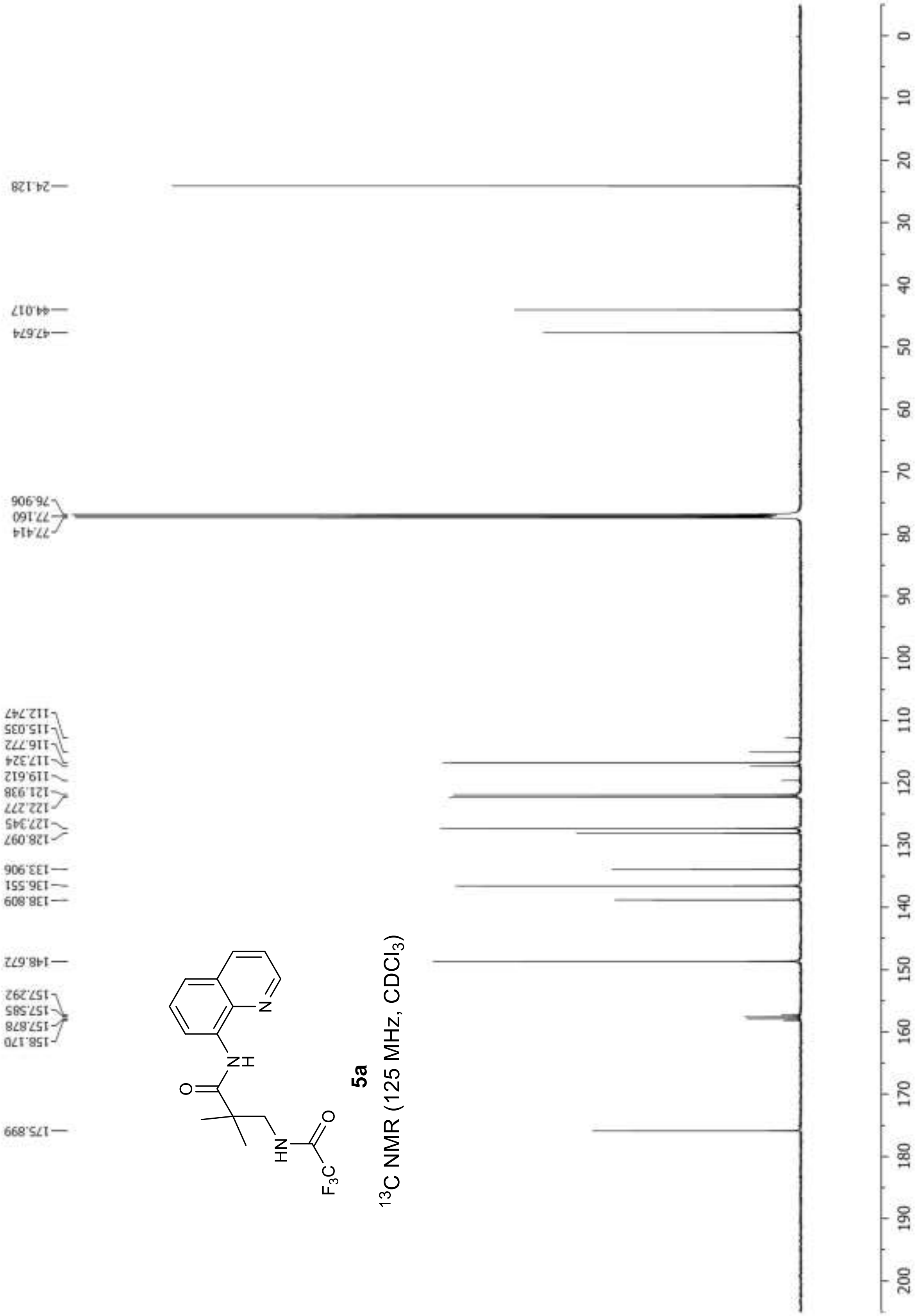

Supplementary Figure 111. ^{13}**C NMR (125 MHz, CDCl$_3$) spectrum for 5a.**

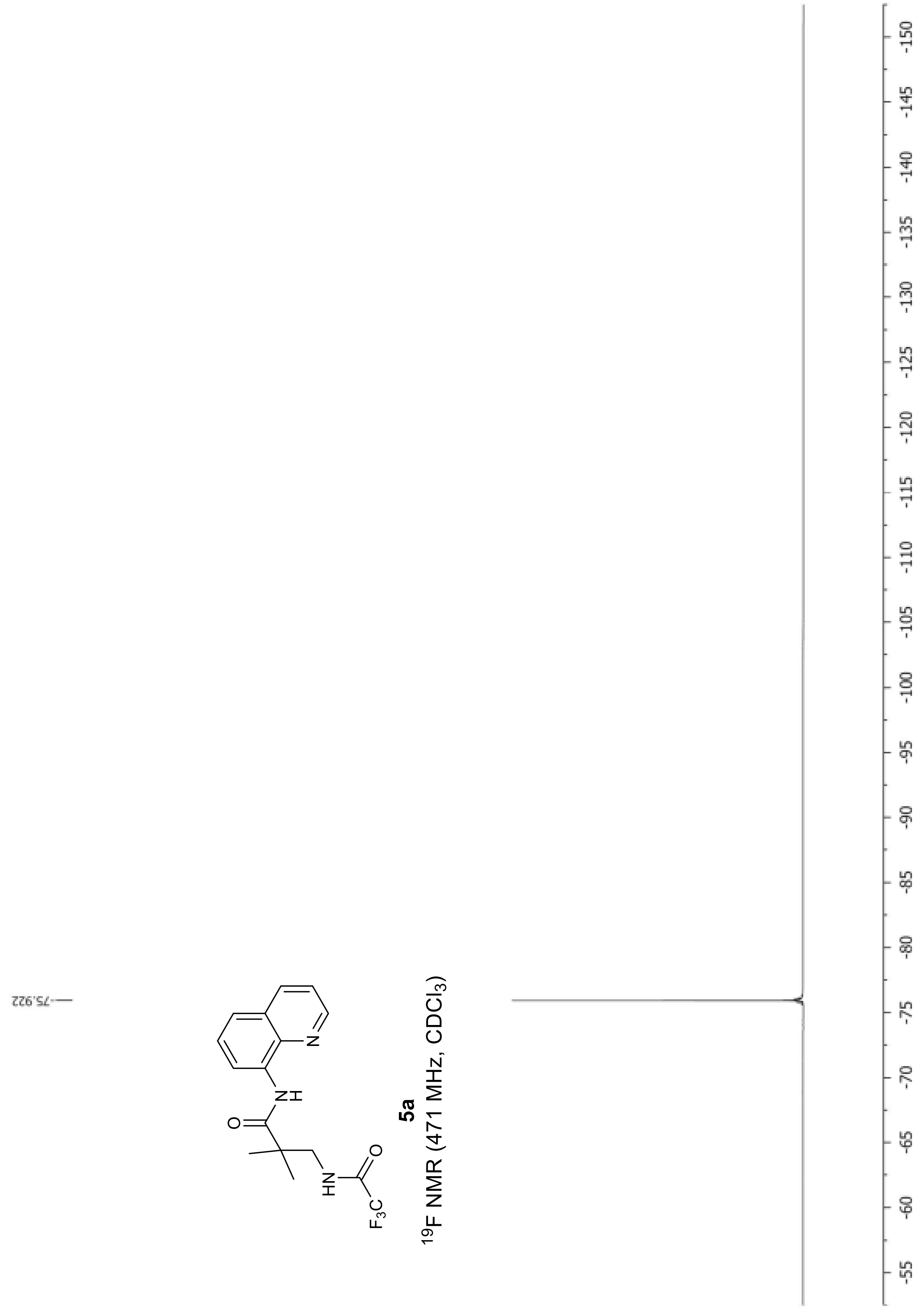

Supplementary Figure 112. ^{19}F NMR (471 MHz, $CDCl_3$) spectrum for 5a.

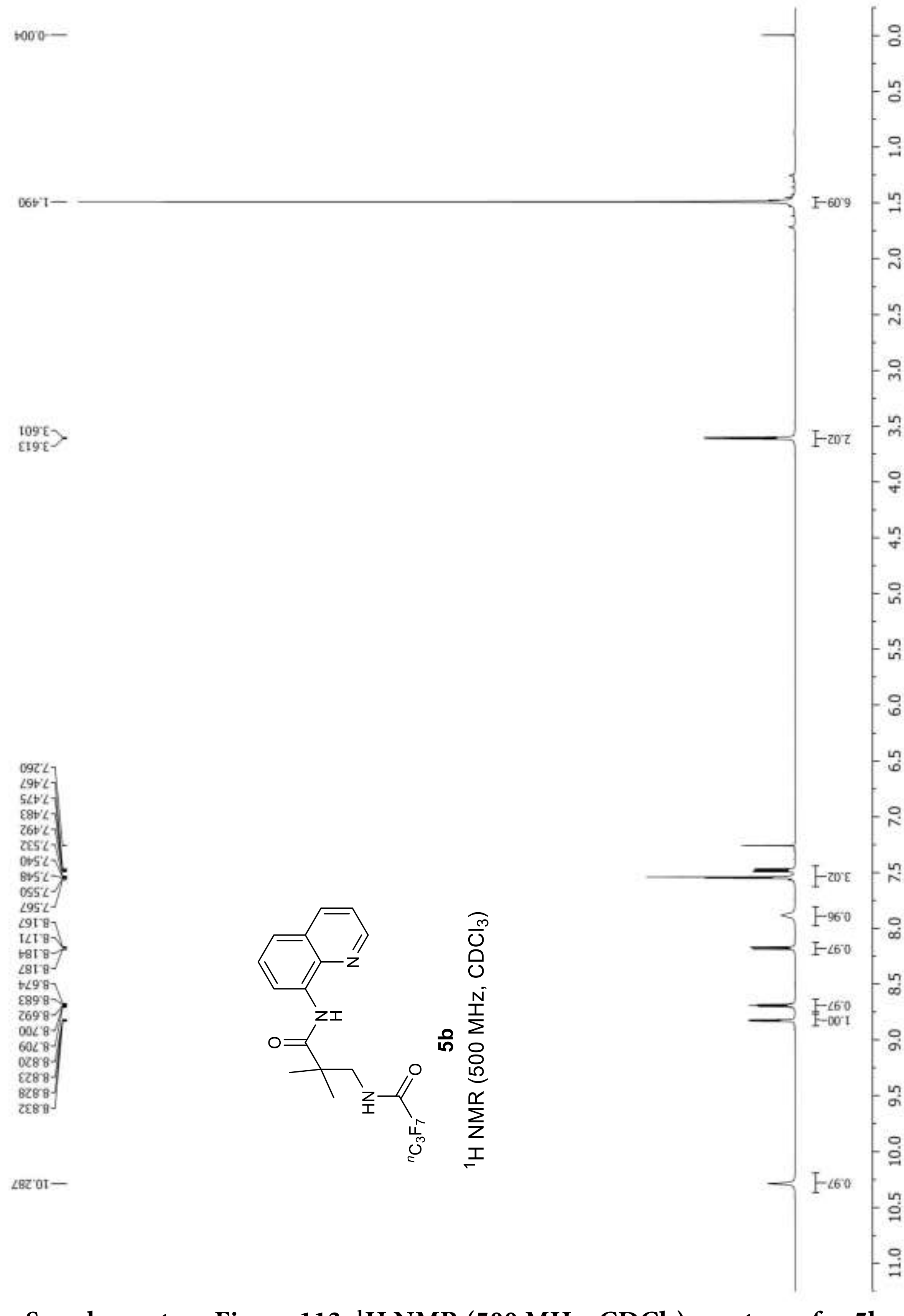

Supplementary Figure 113. ^{1}H NMR (500 MHz, $CDCl_3$) spectrum for 5b.

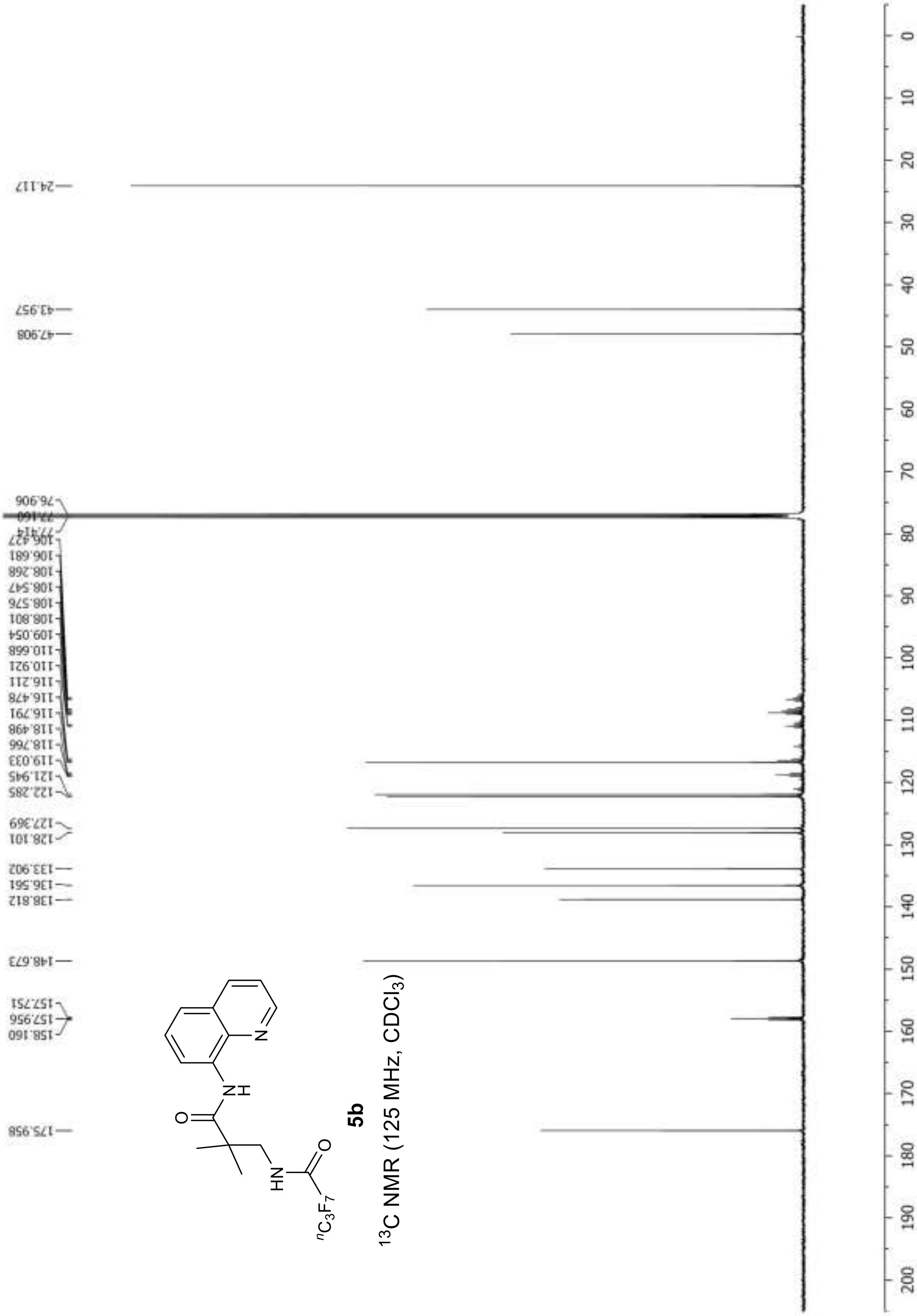

Supplementary Figure 114. ^{13}C NMR (125 MHz, $CDCl_3$) spectrum for 5b.

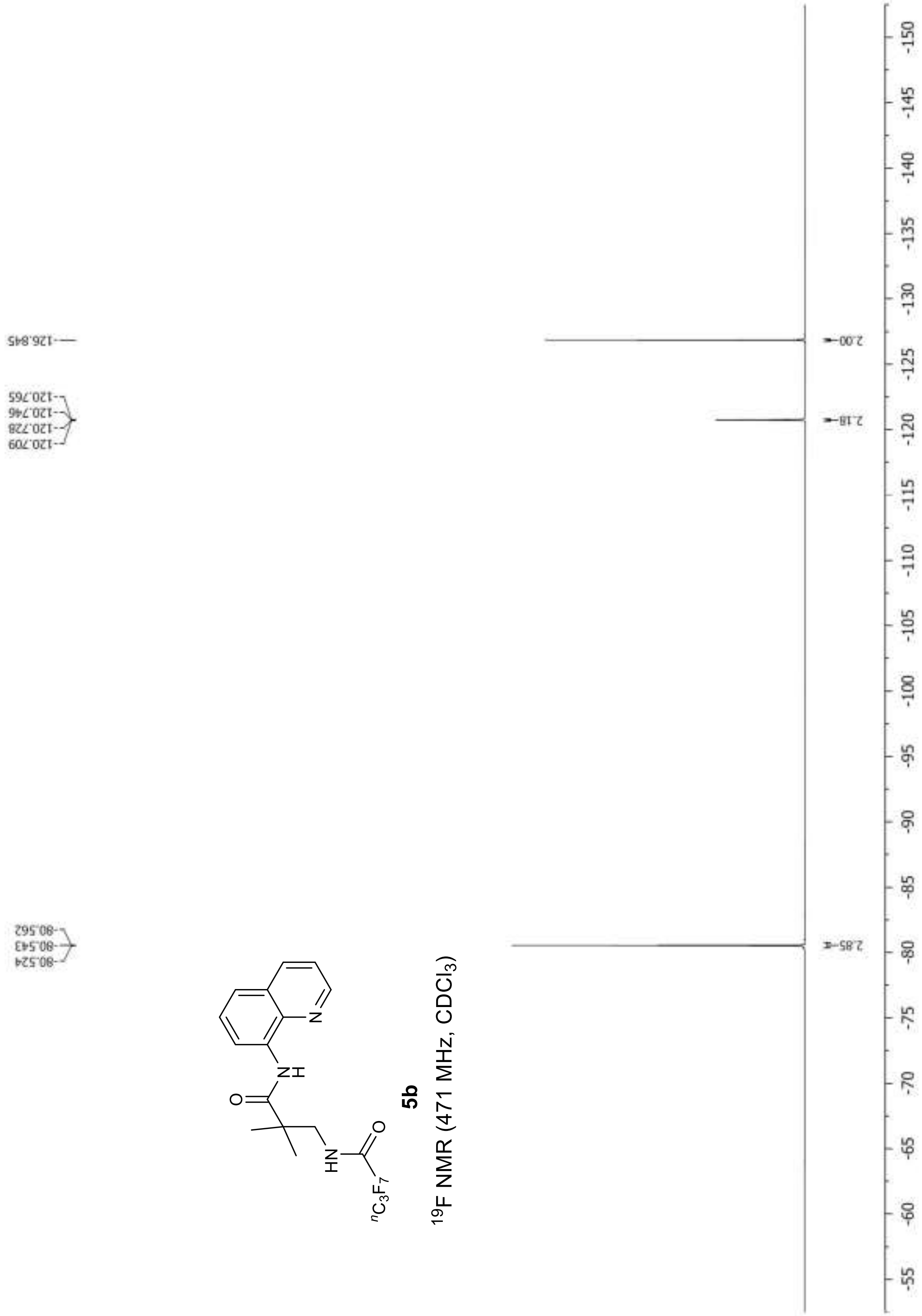

Supplementary Figure 115. ^{19}F NMR (471 MHz, CDCl$_3$) spectrum for 5b.

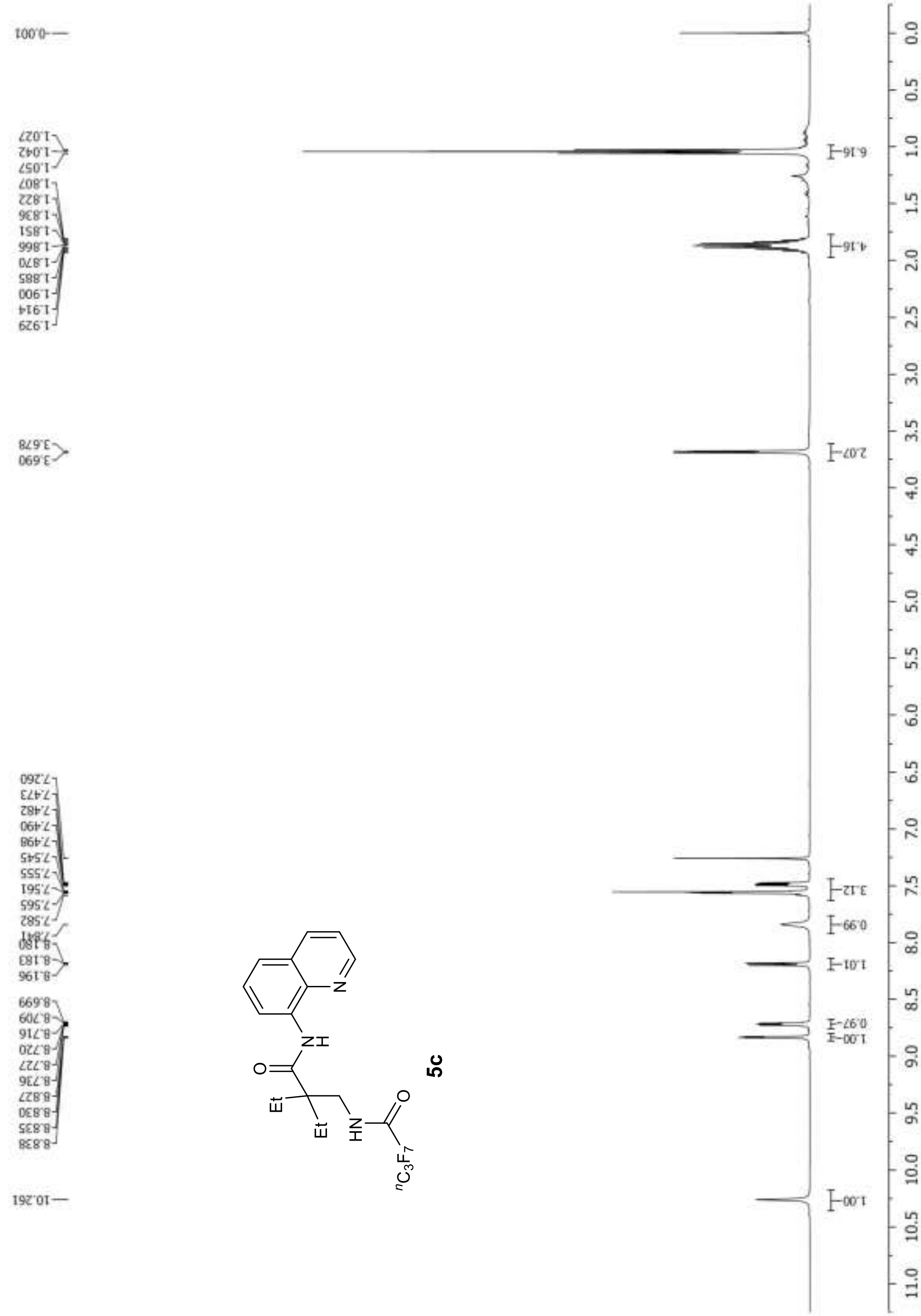

Supplementary Figure 116. ^{1}H NMR (500 MHz, $CDCl_3$) spectrum for 5c.

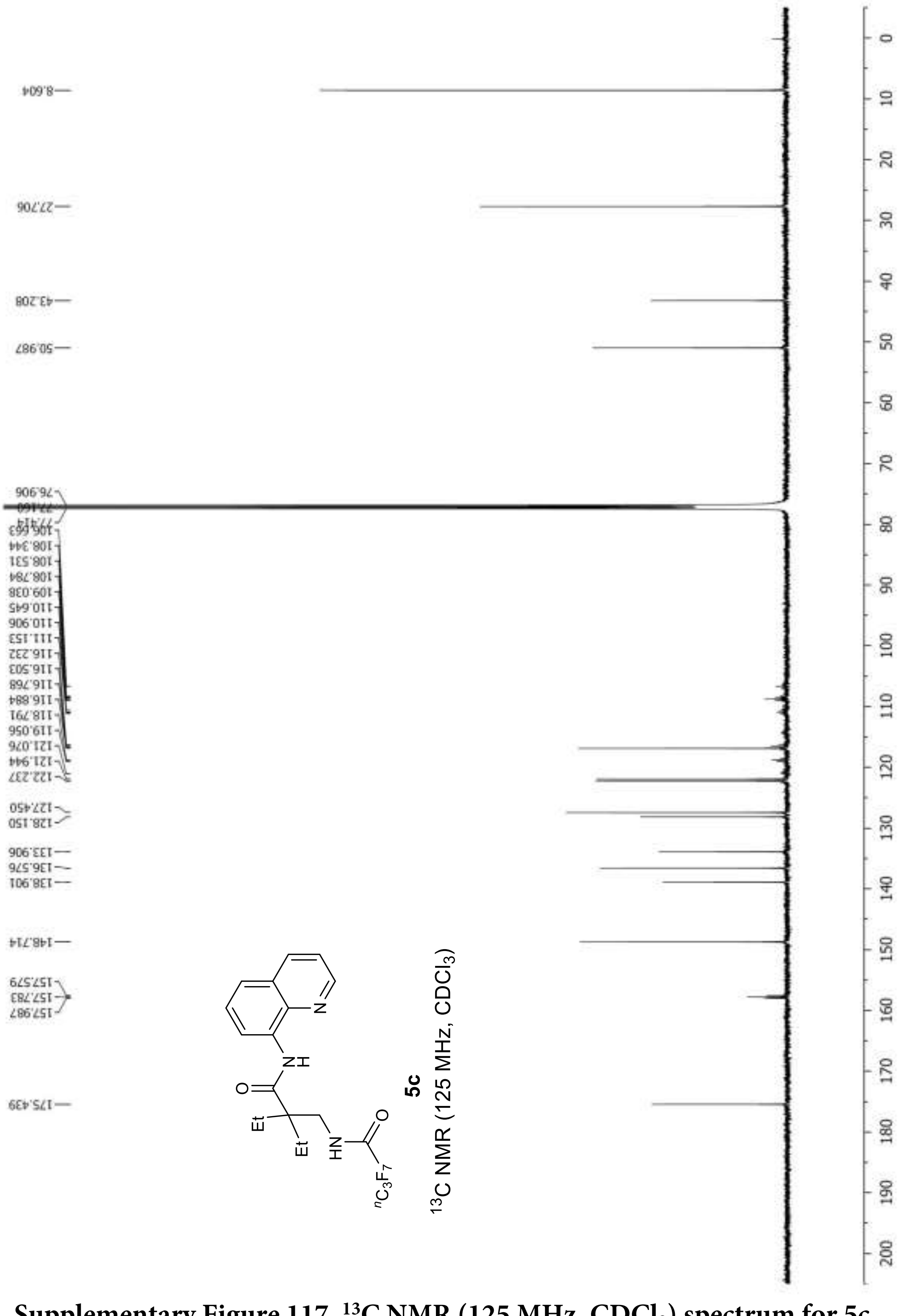

Supplementary Figure 117. ^{13}**C NMR (125 MHz, CDCl$_3$) spectrum for 5c.**

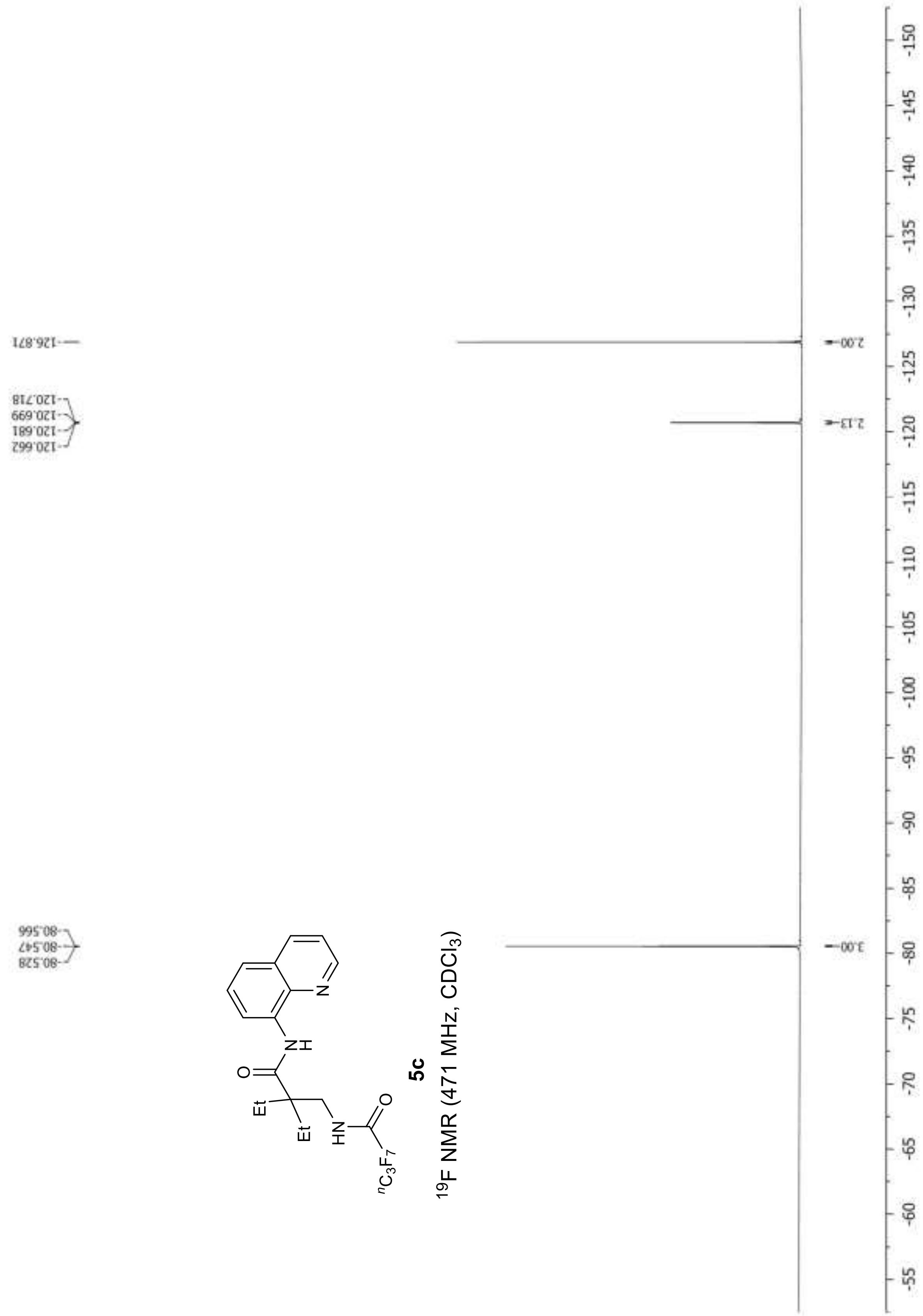

Supplementary Figure 118. ^{19}F NMR (471 MHz, $CDCl_3$) spectrum for 5c.

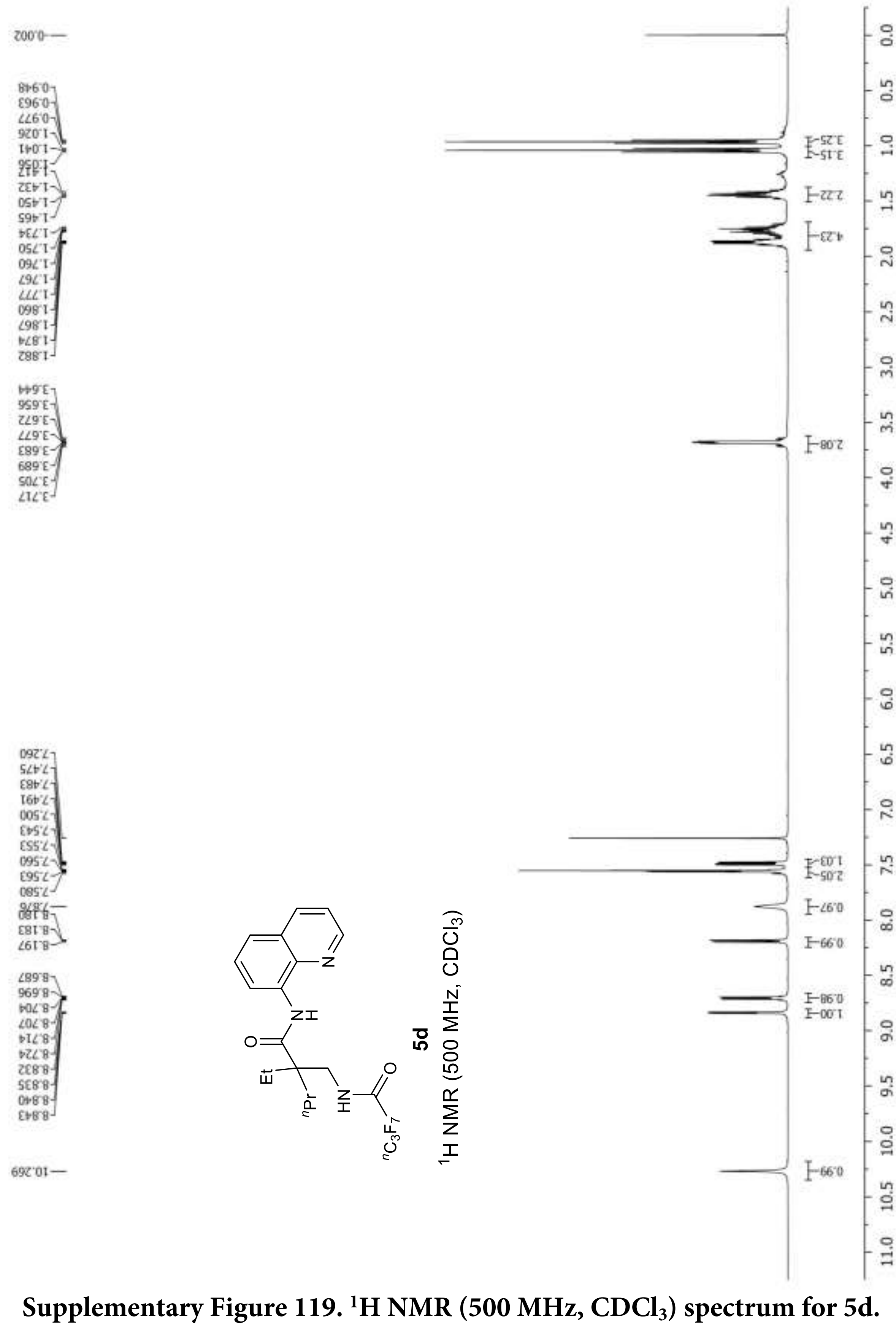

Supplementary Figure 119. ^{1}H NMR (500 MHz, $CDCl_3$) spectrum for 5d.

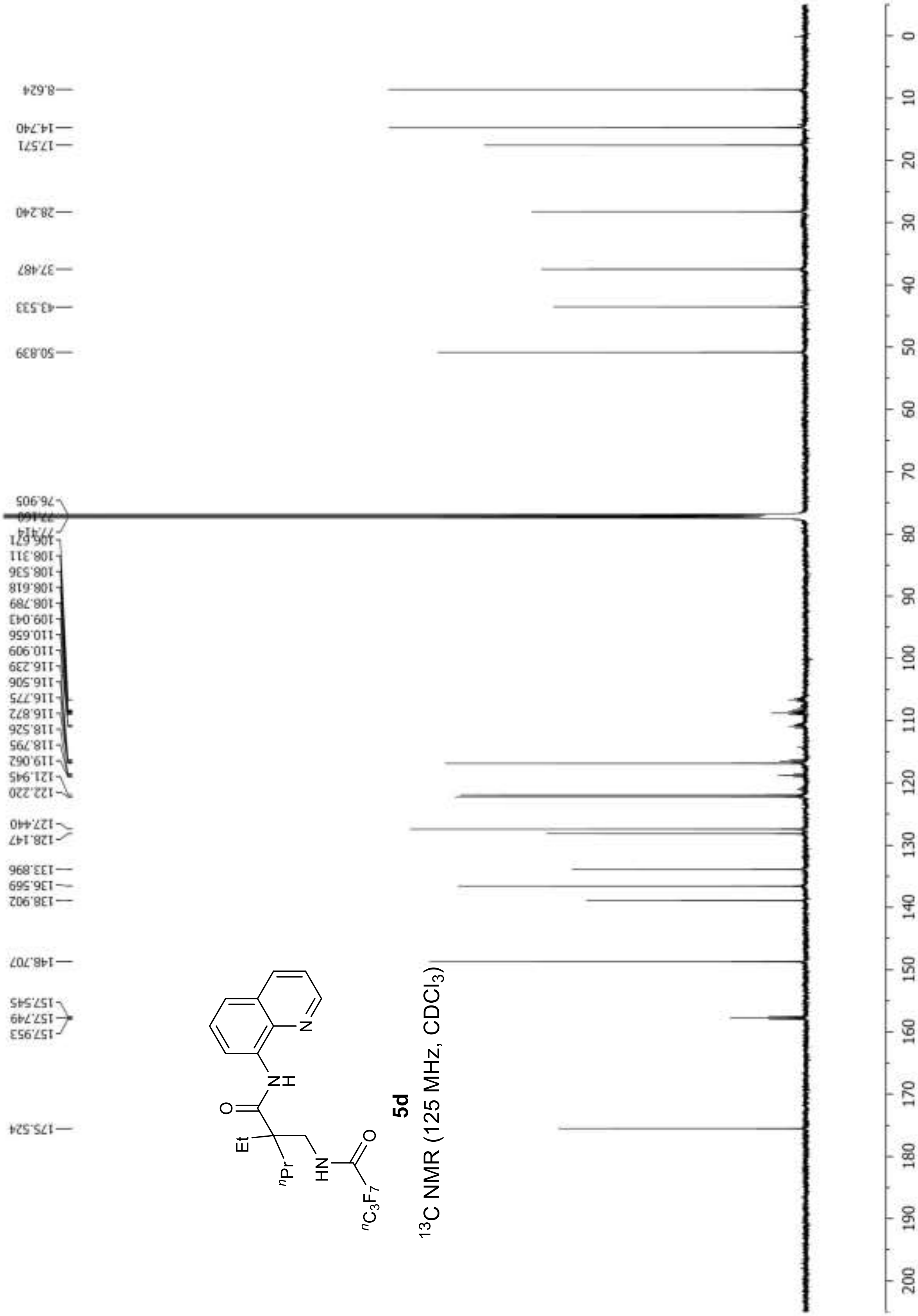

Supplementary Figure 120. ^{13}**C NMR (125 MHz, CDCl**$_3$**) spectrum for 5d.**

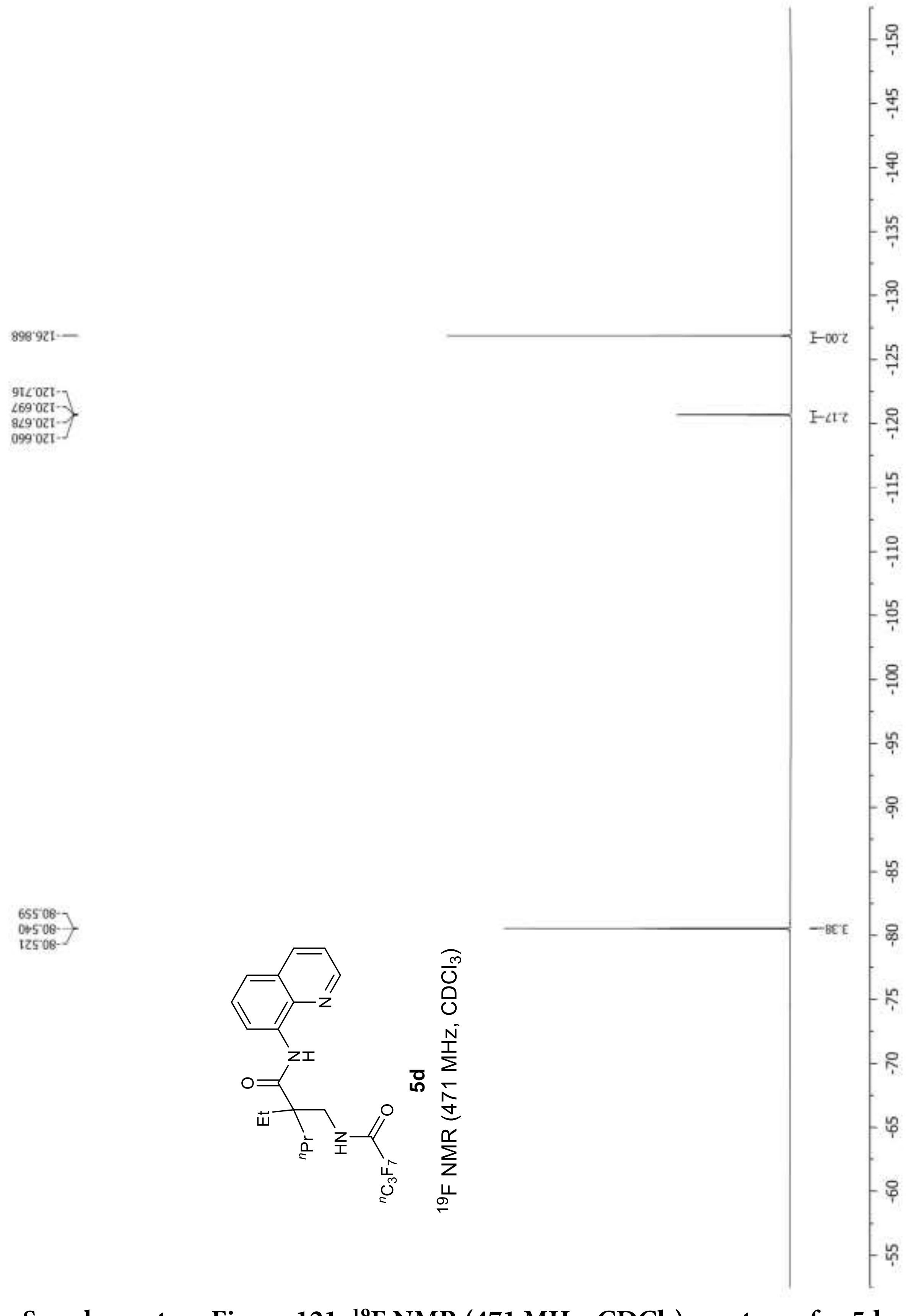

Supplementary Figure 121. ^{19}F NMR (471 MHz, $CDCl_3$) spectrum for 5d.

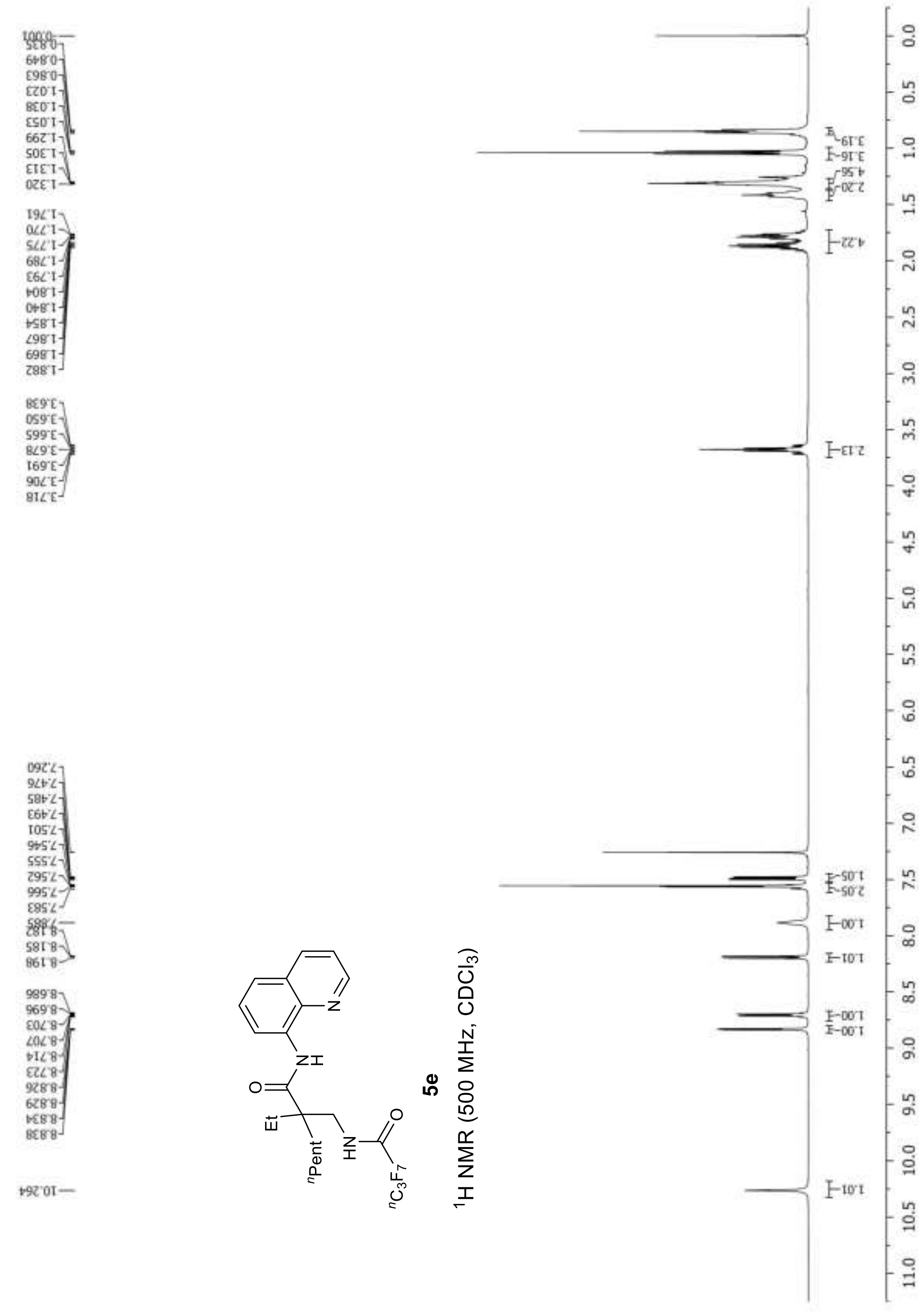

Supplementary Figure 122. ^{1}**H NMR (500 MHz,** $CDCl_3$**) spectrum for 5e.**

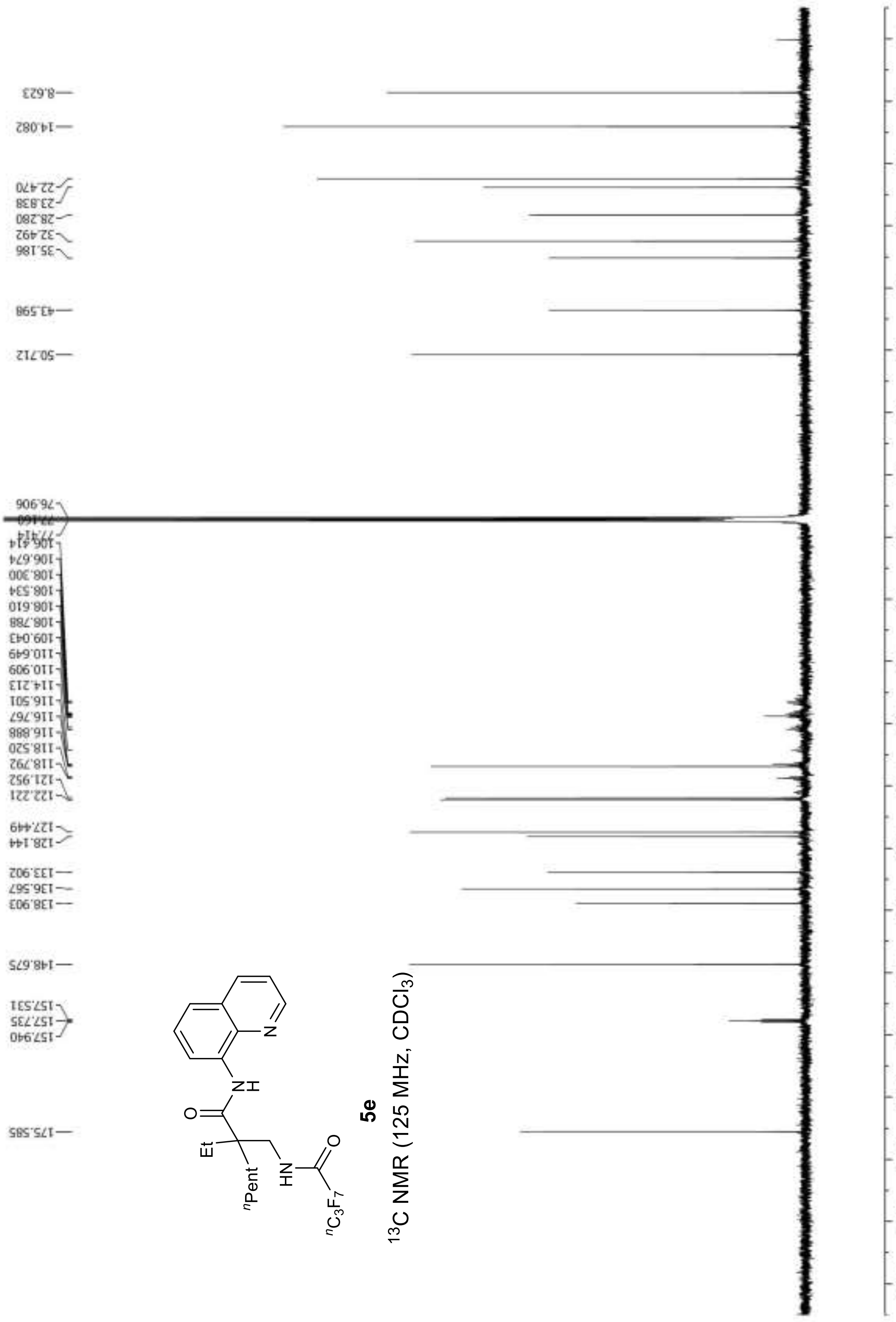

Supplementary Figure 123. ^{13}C NMR (125 MHz, $CDCl_3$) spectrum for 5e.

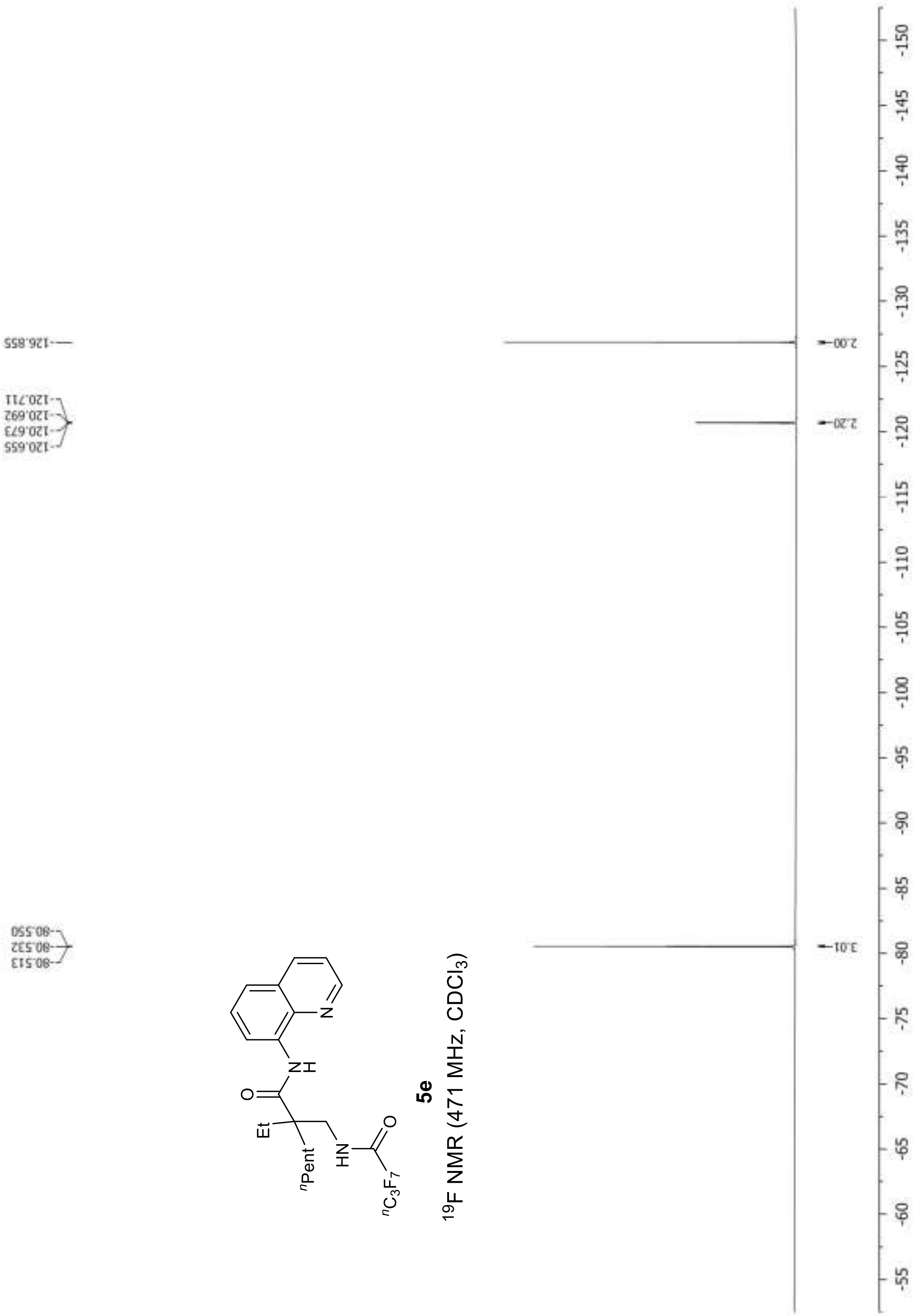

Supplementary Figure 124. ^{19}**F NMR (471 MHz,** $CDCl_3$**) spectrum for 5e.**

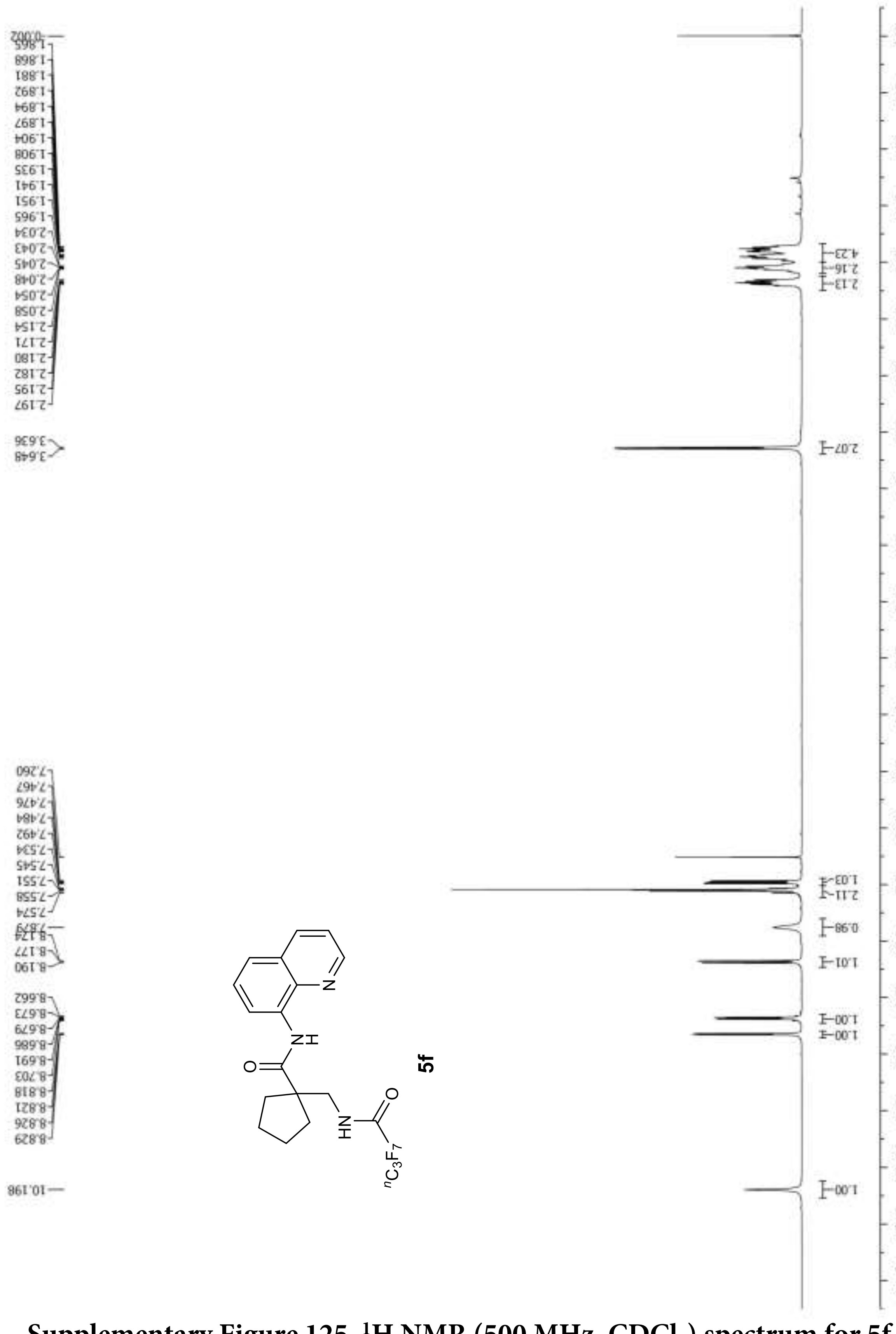

Supplementary Figure 125. ^{1}H NMR (500 MHz, $CDCl_3$) spectrum for 5f.

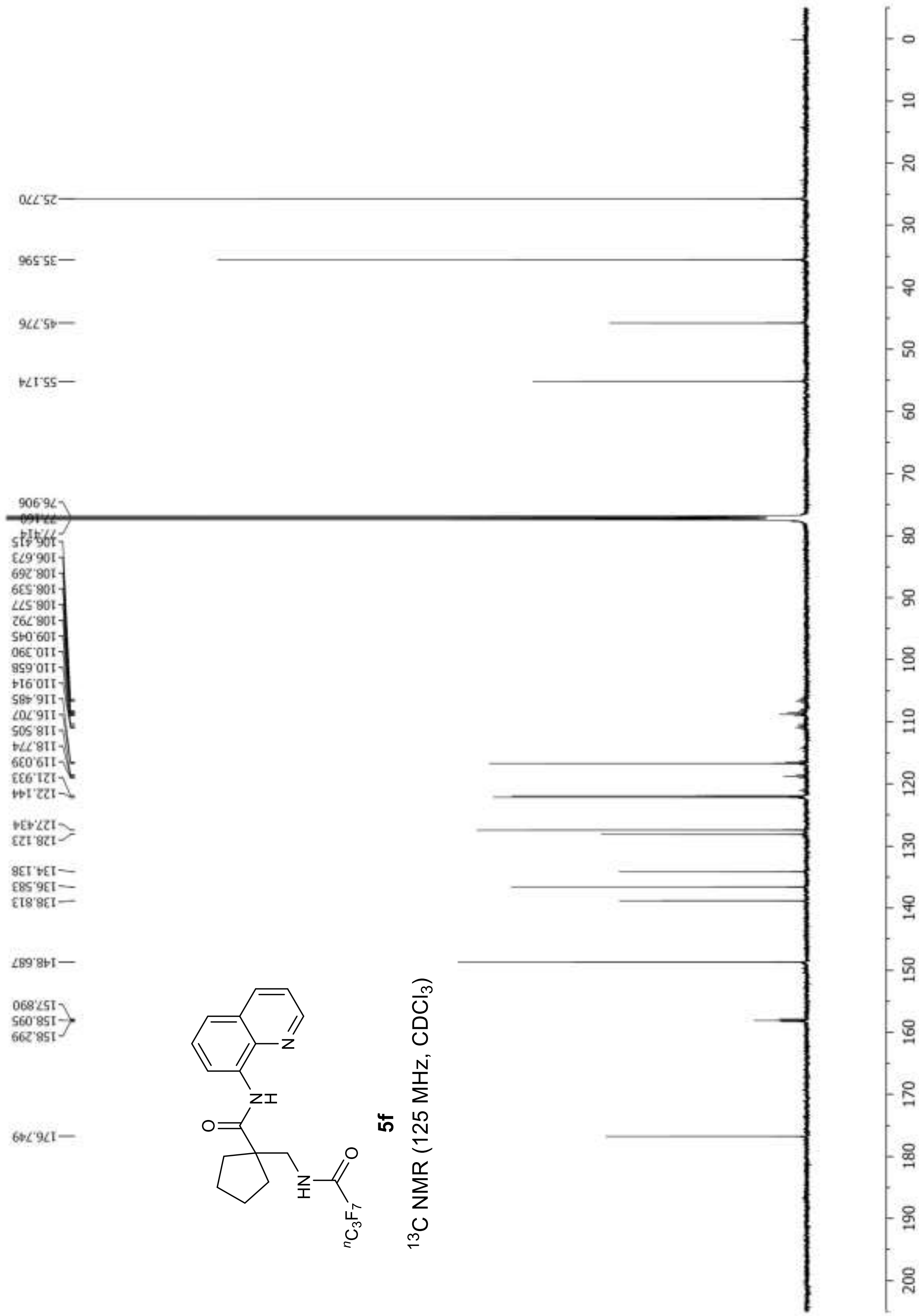

Supplementary Figure 126. ^{13}C NMR (125 MHz, $CDCl_3$) spectrum for 5f.

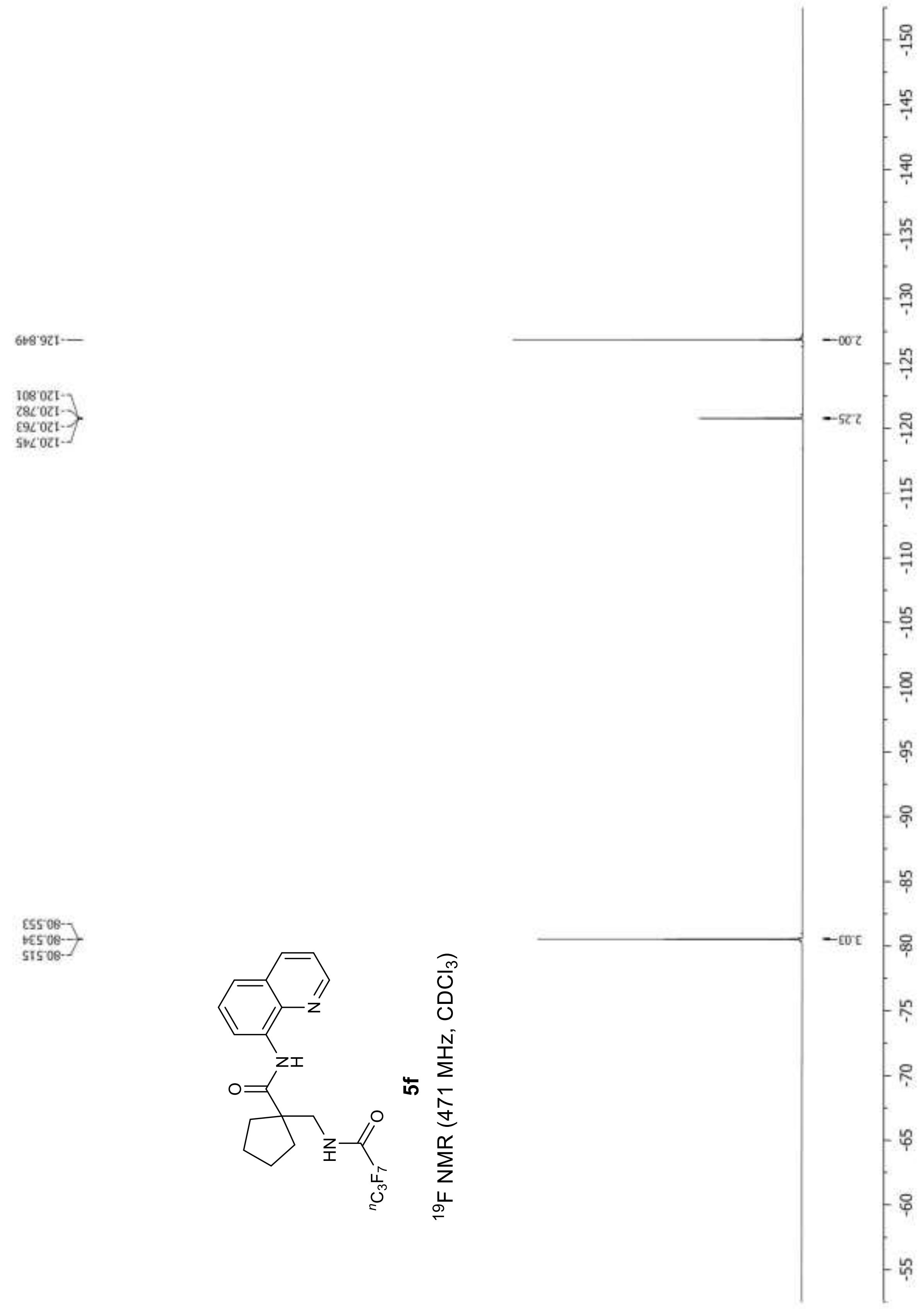

Supplementary Figure 127. ^{19}F NMR (471 MHz, $CDCl_3$) spectrum for 5f.

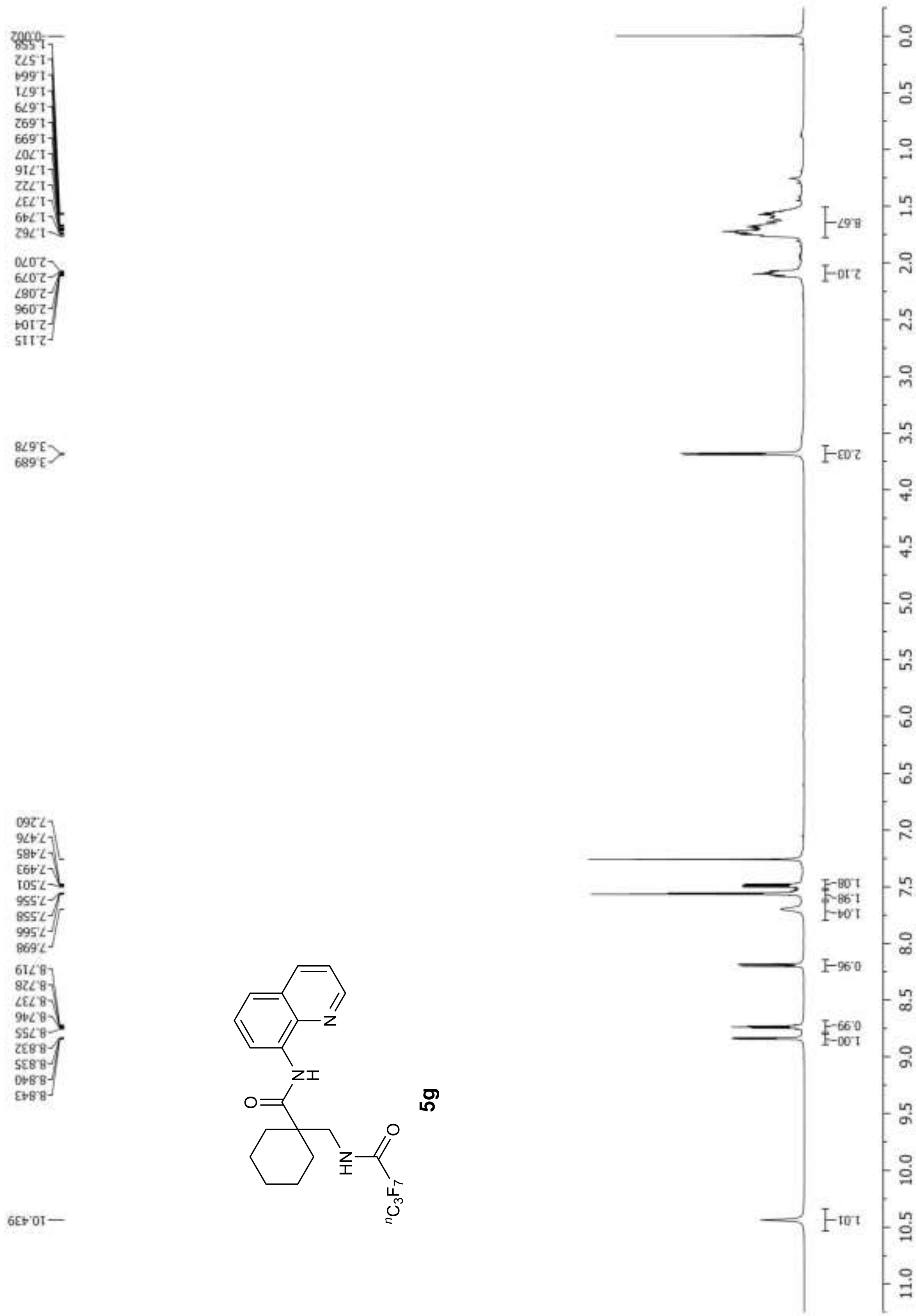

Supplementary Figure 128. ^{1}H NMR (500 MHz, $CDCl_3$) spectrum for 5g.

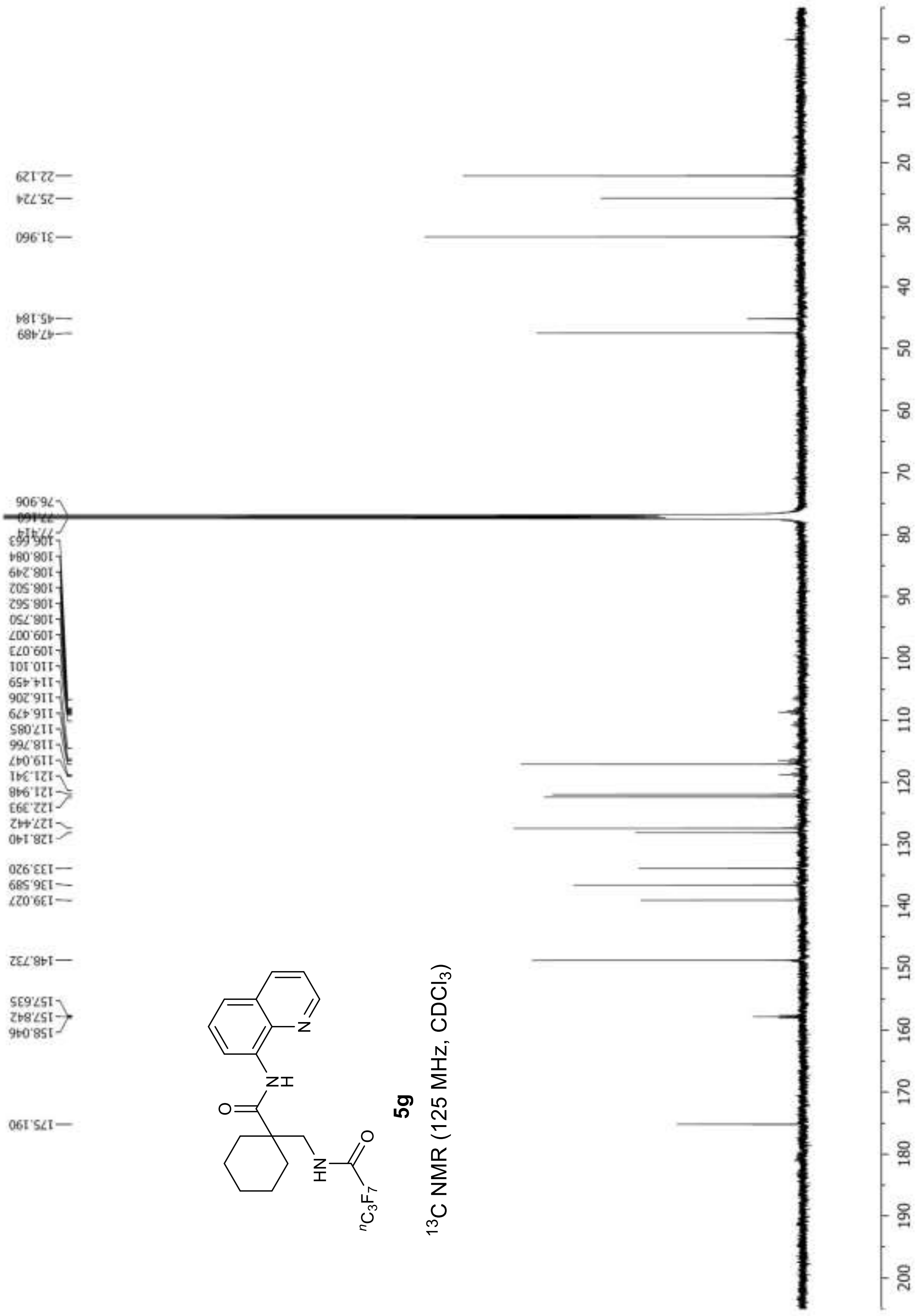

Supplementary Figure 129. ^{13}**C NMR (125 MHz, CDCl**$_3$**) spectrum for 5g.**

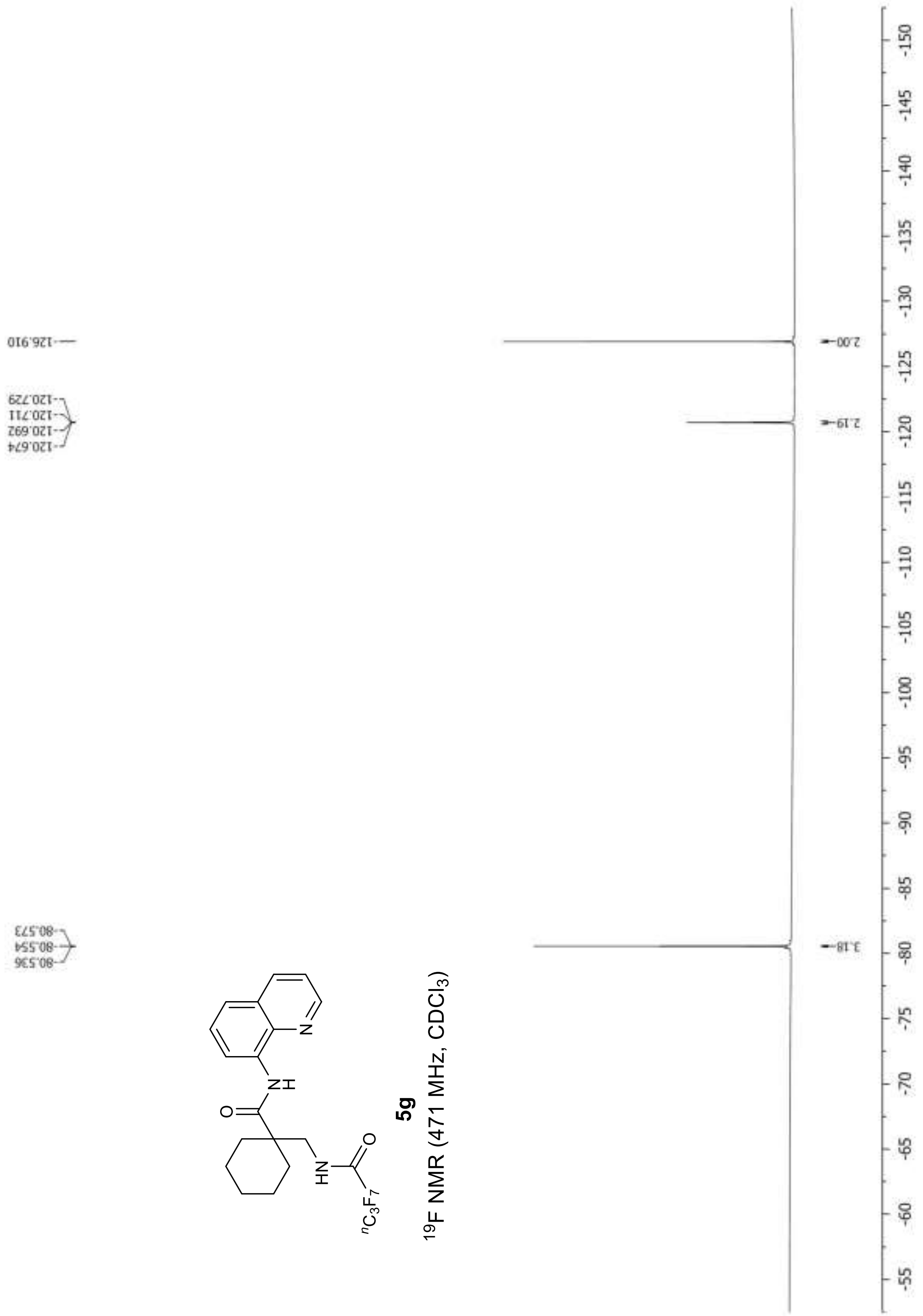

Supplementary Figure 130. ^{19}F NMR (471 MHz, $CDCl_3$) spectrum for 5g.

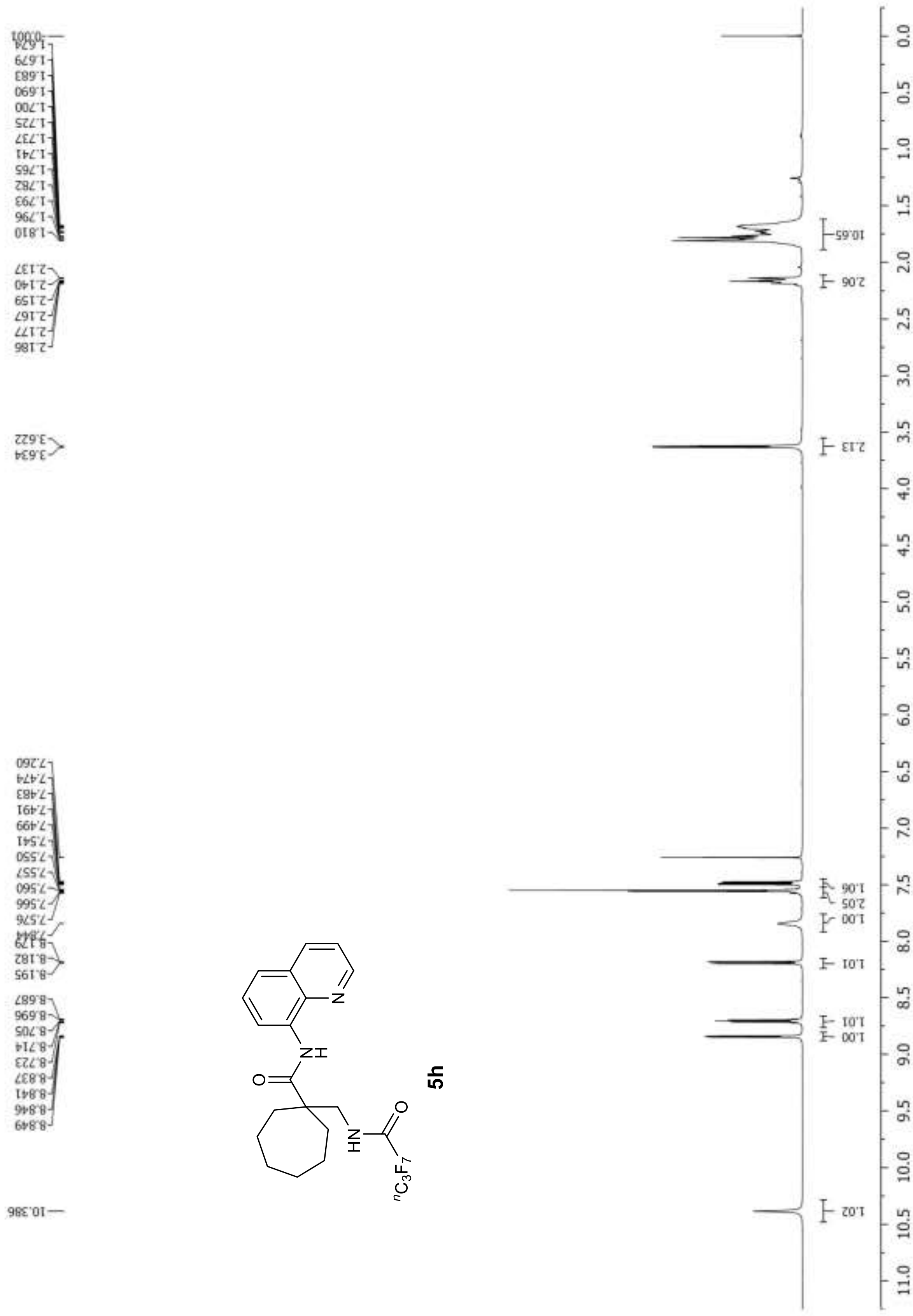

Supplementary Figure 131. ^{1}H NMR (500 MHz, $CDCl_3$) spectrum for 5h.

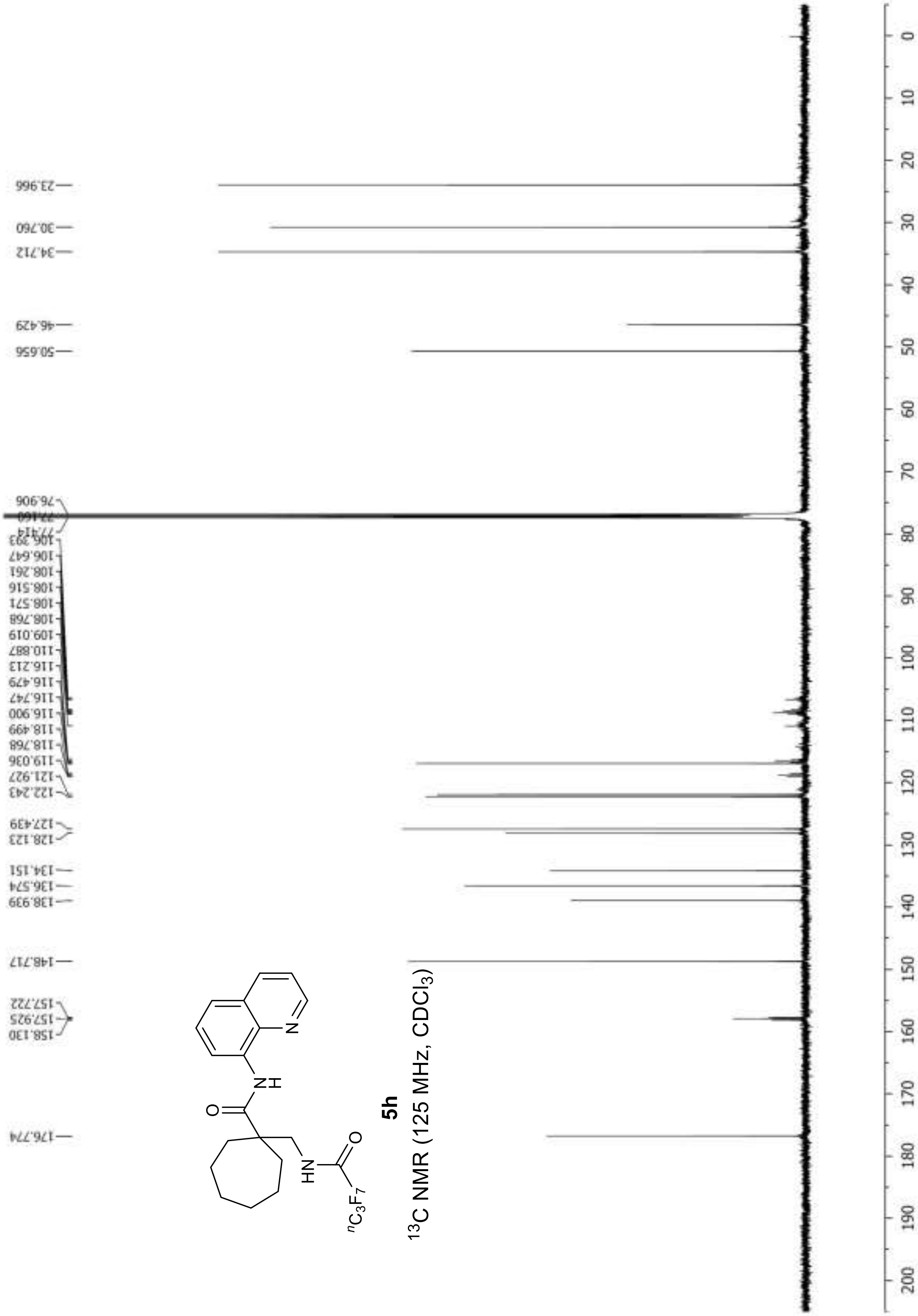

Supplementary Figure 132. ^{13}C NMR (125 MHz, $CDCl_3$) spectrum for 5h.

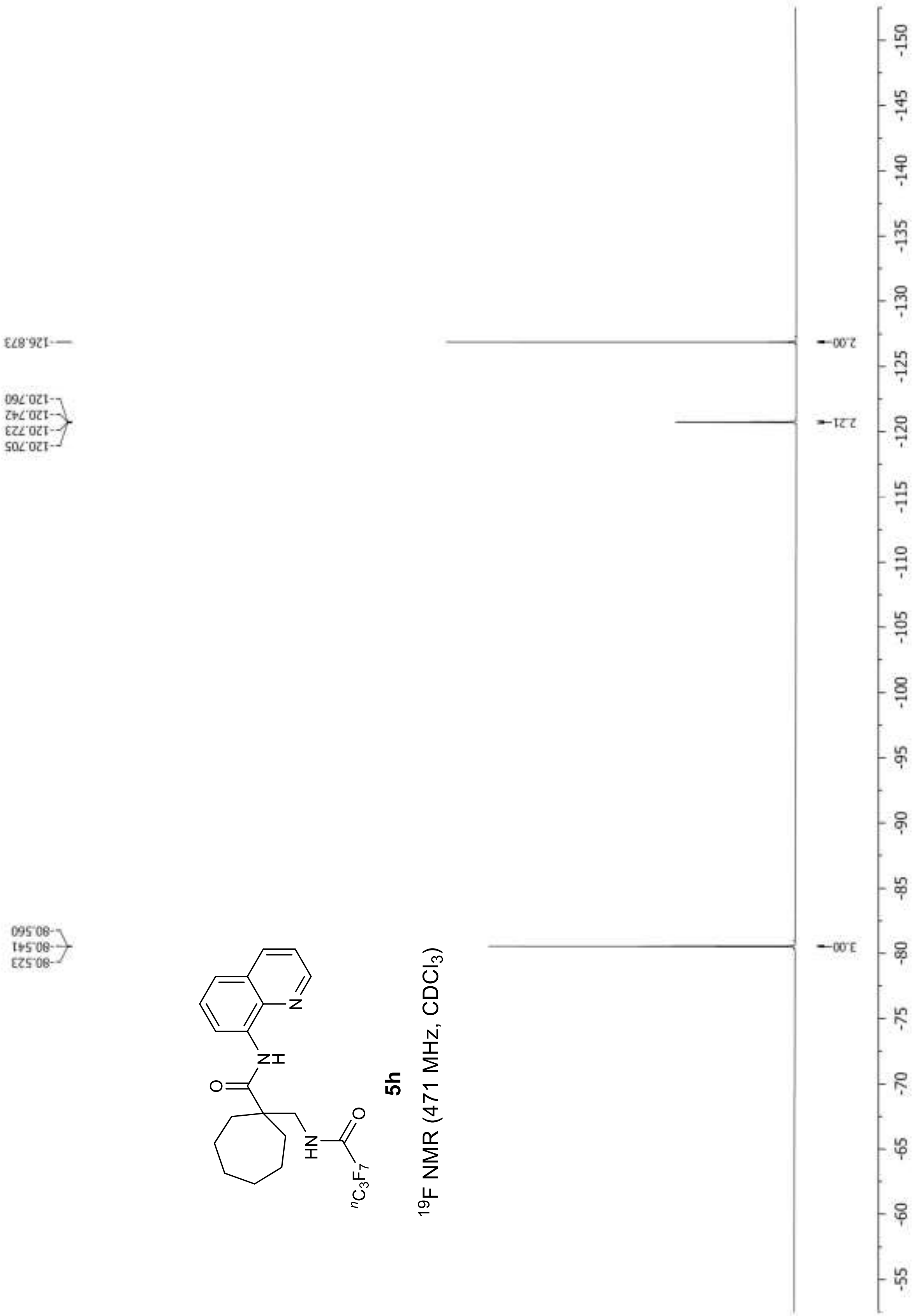

Supplementary Figure 133. ^{19}F NMR (471 MHz, $CDCl_3$) spectrum for 5h.

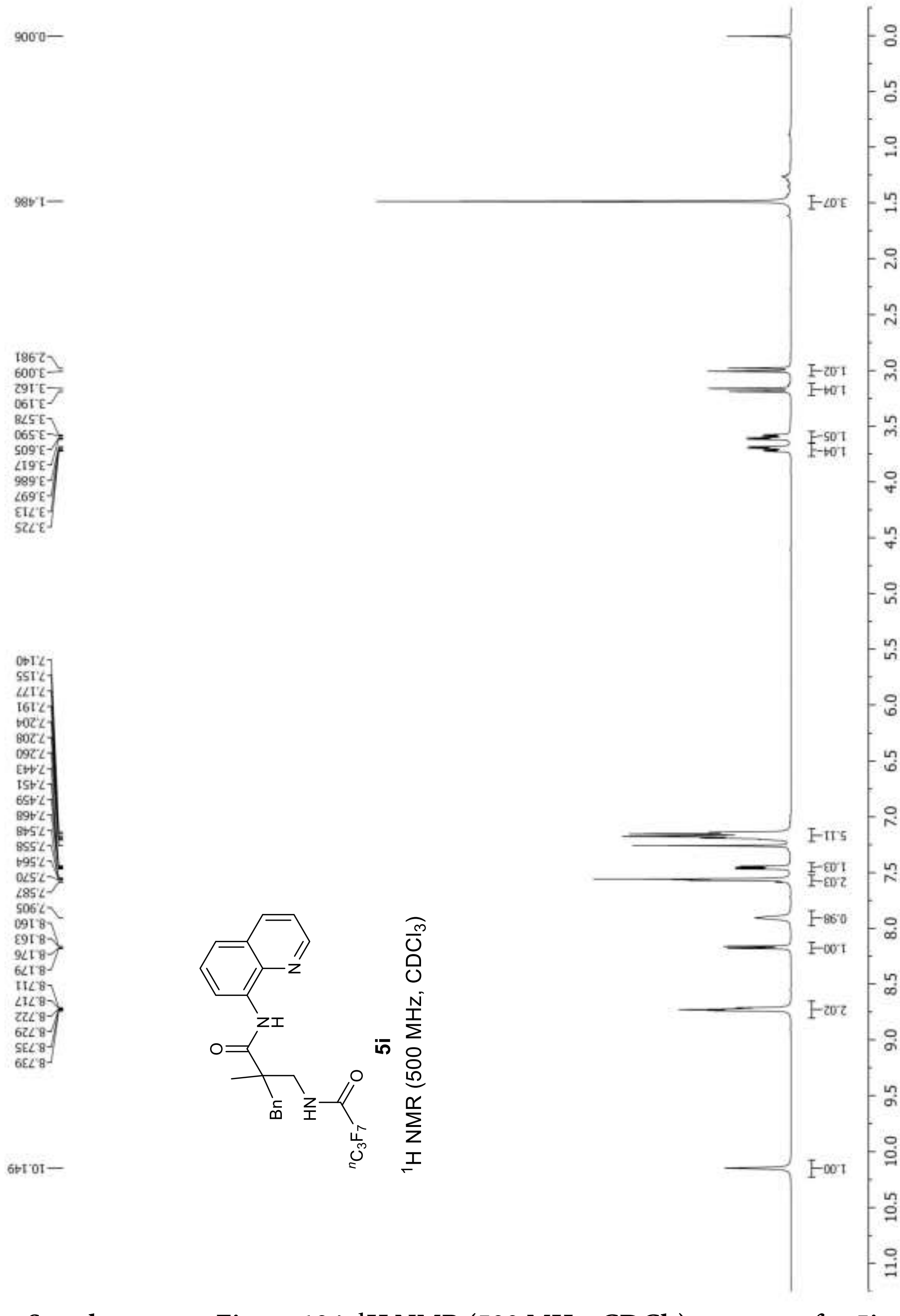

Supplementary Figure 134. ^{1}H NMR (500 MHz, $CDCl_3$) spectrum for 5i.

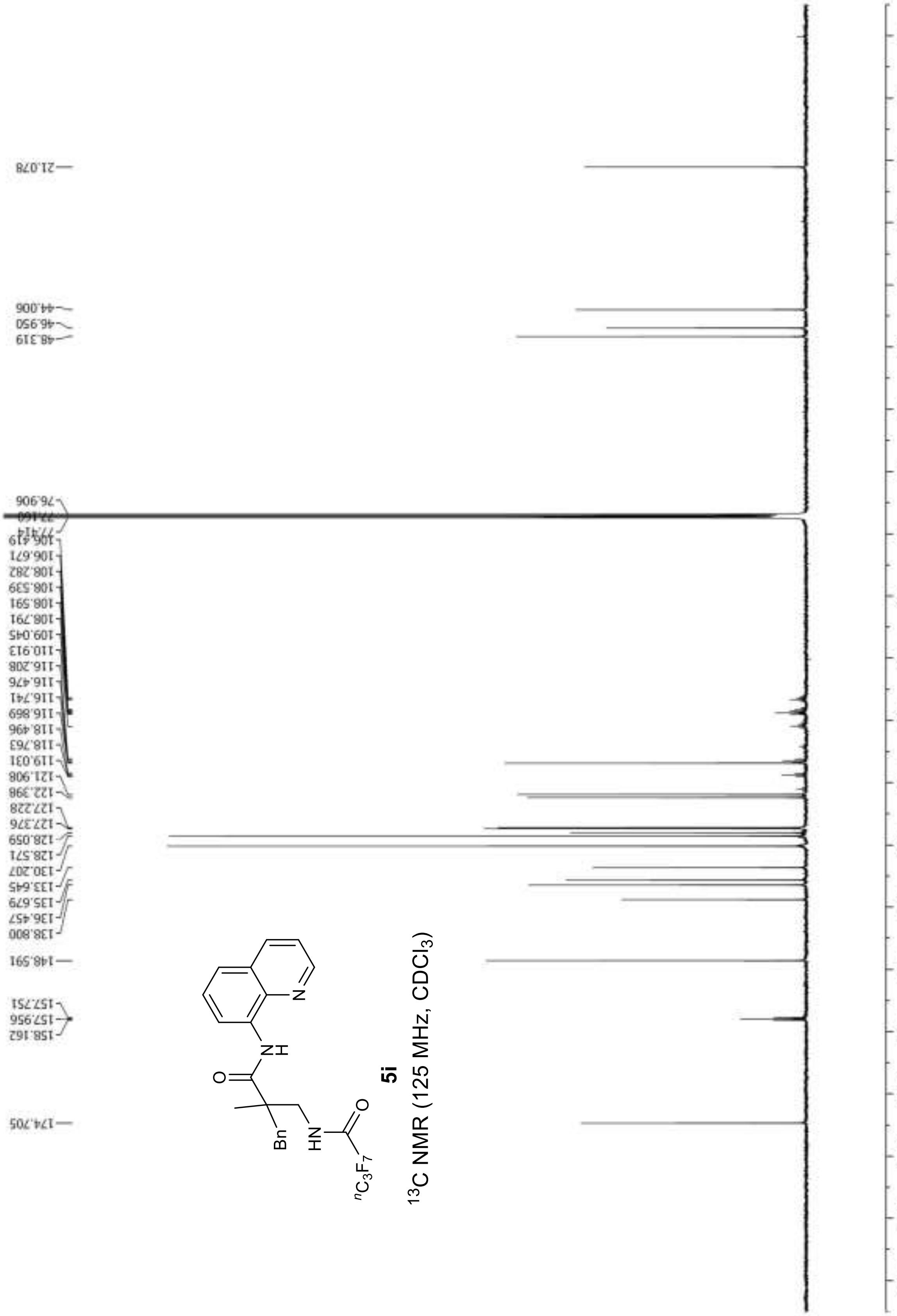

Supplementary Figure 135. ^{13}C NMR (125 MHz, $CDCl_3$) spectrum for 5i.

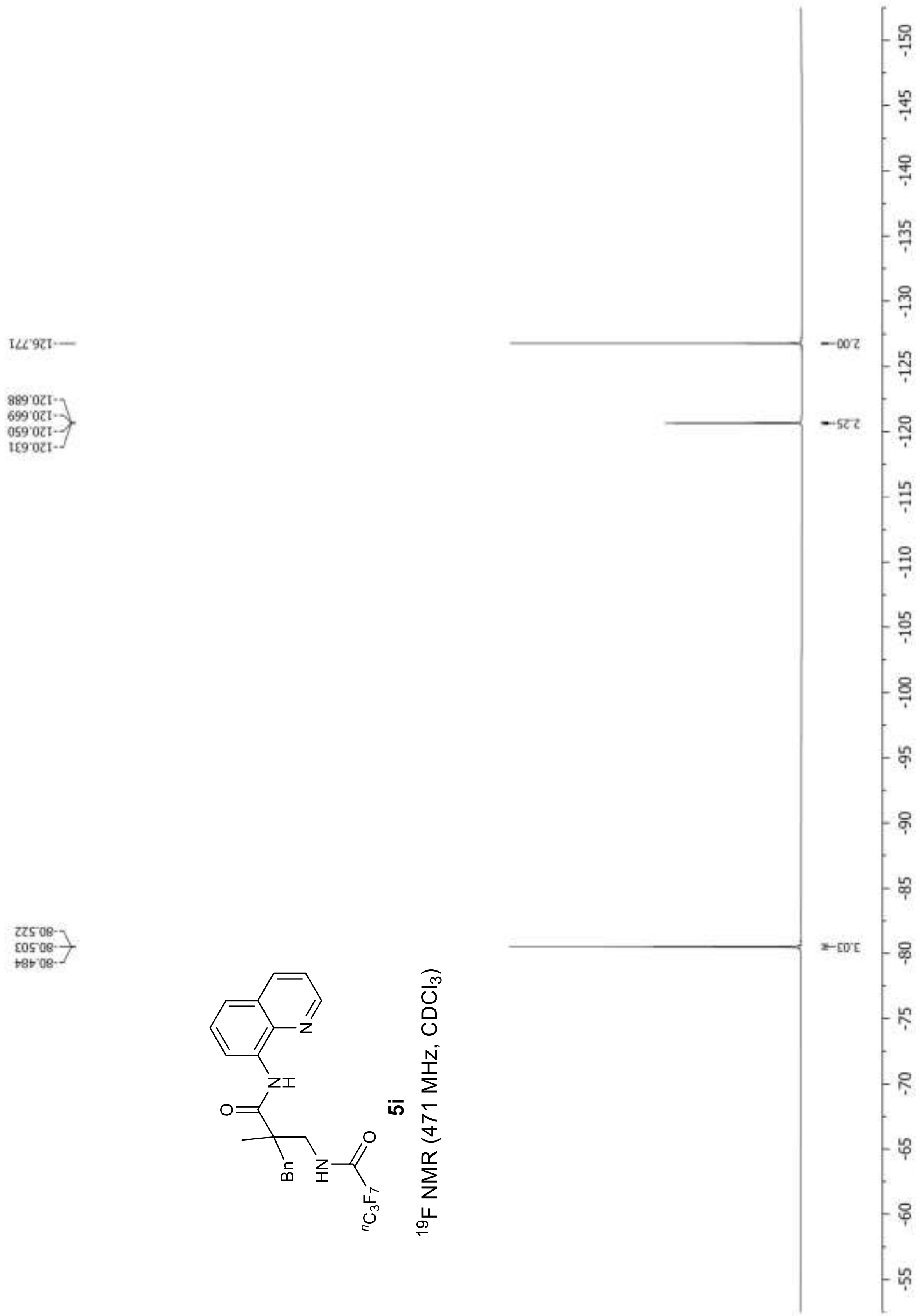

Supplementary Figure 136. ^{19}F NMR (471 MHz, $CDCl_3$) spectrum for 5i.

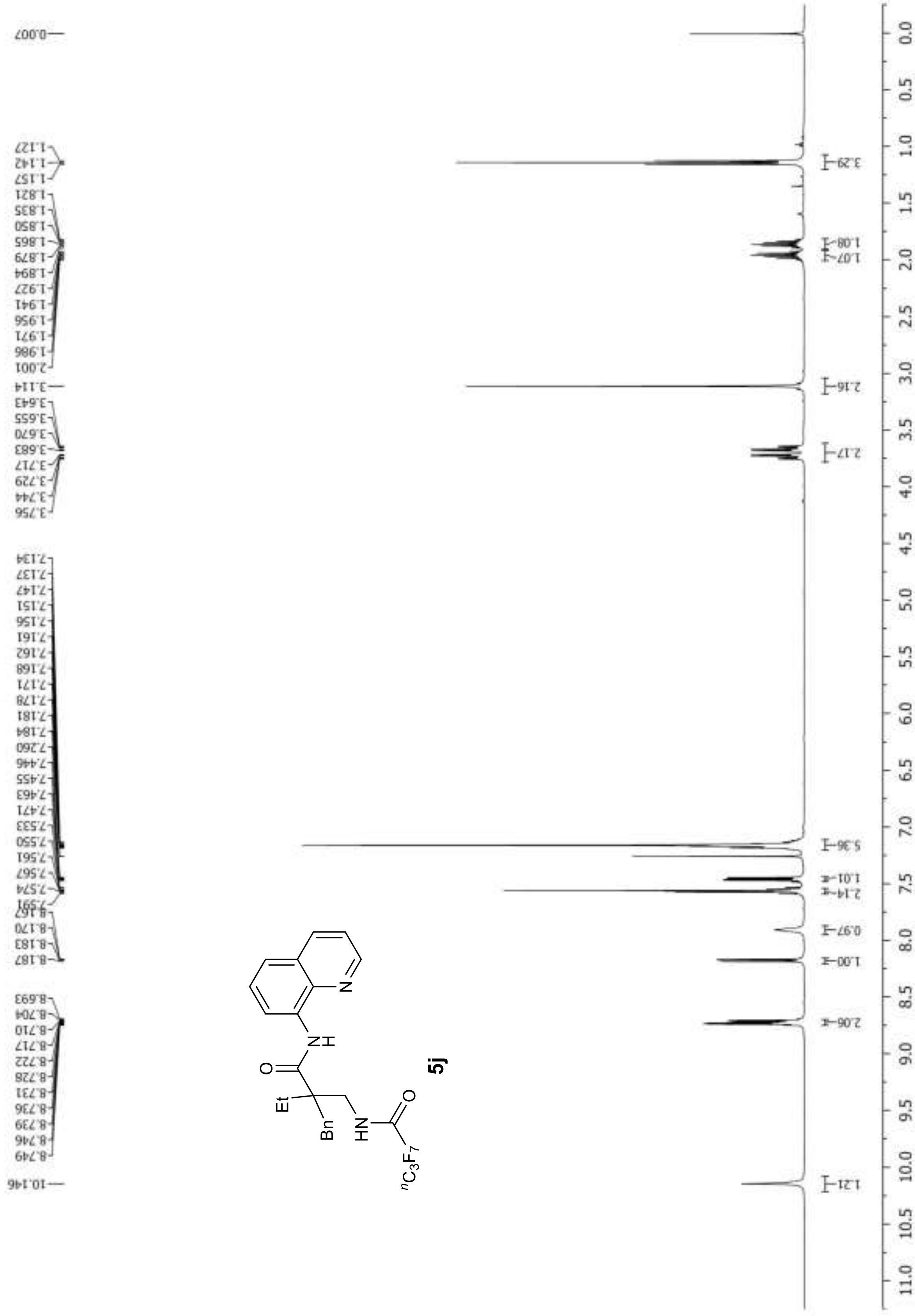

Supplementary Figure 137. ^{1}H NMR (500 MHz, $CDCl_3$) spectrum for 5j.

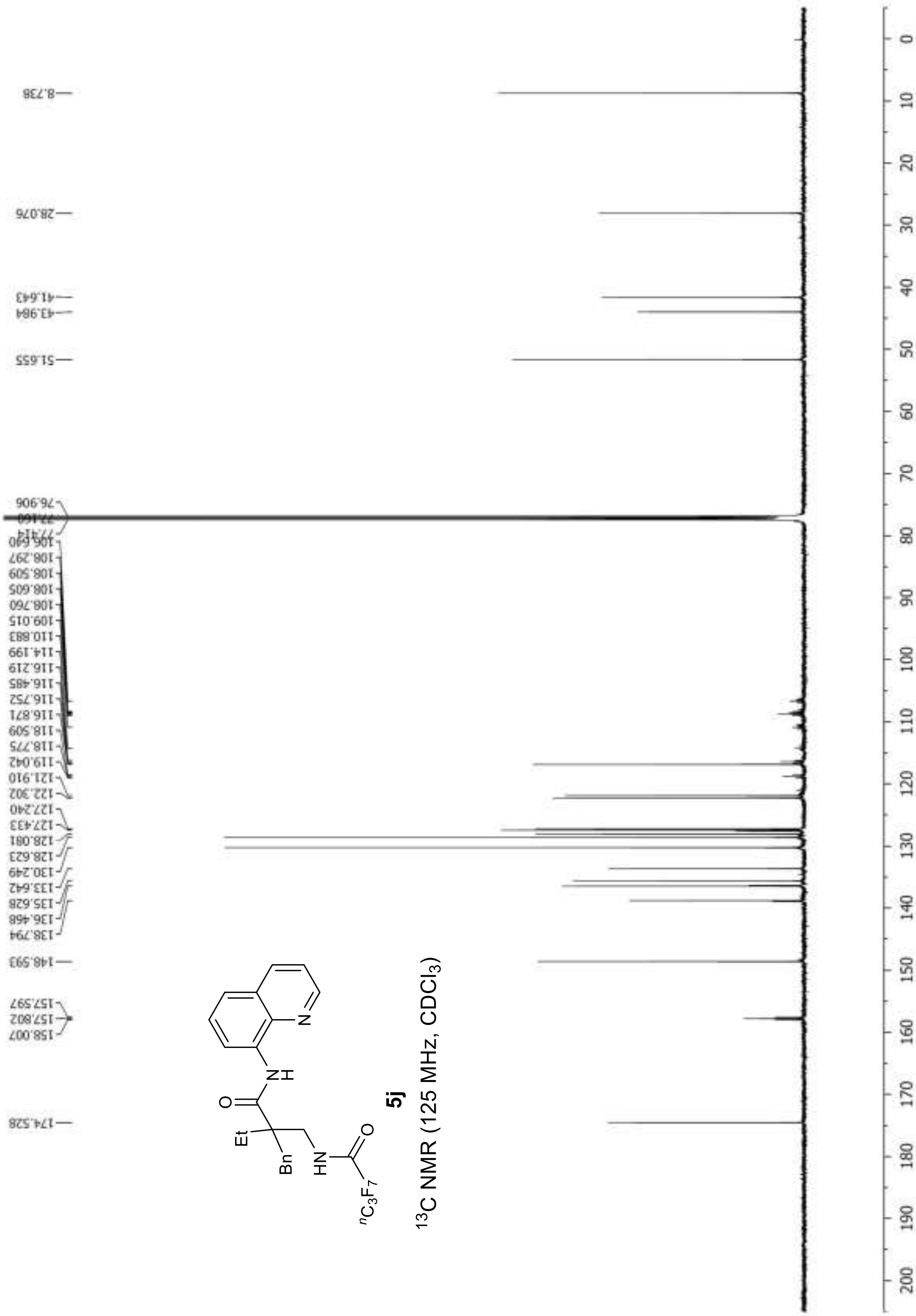

Supplementary Figure 138. ^{13}C NMR (125 MHz, $CDCl_3$) spectrum for 5j.

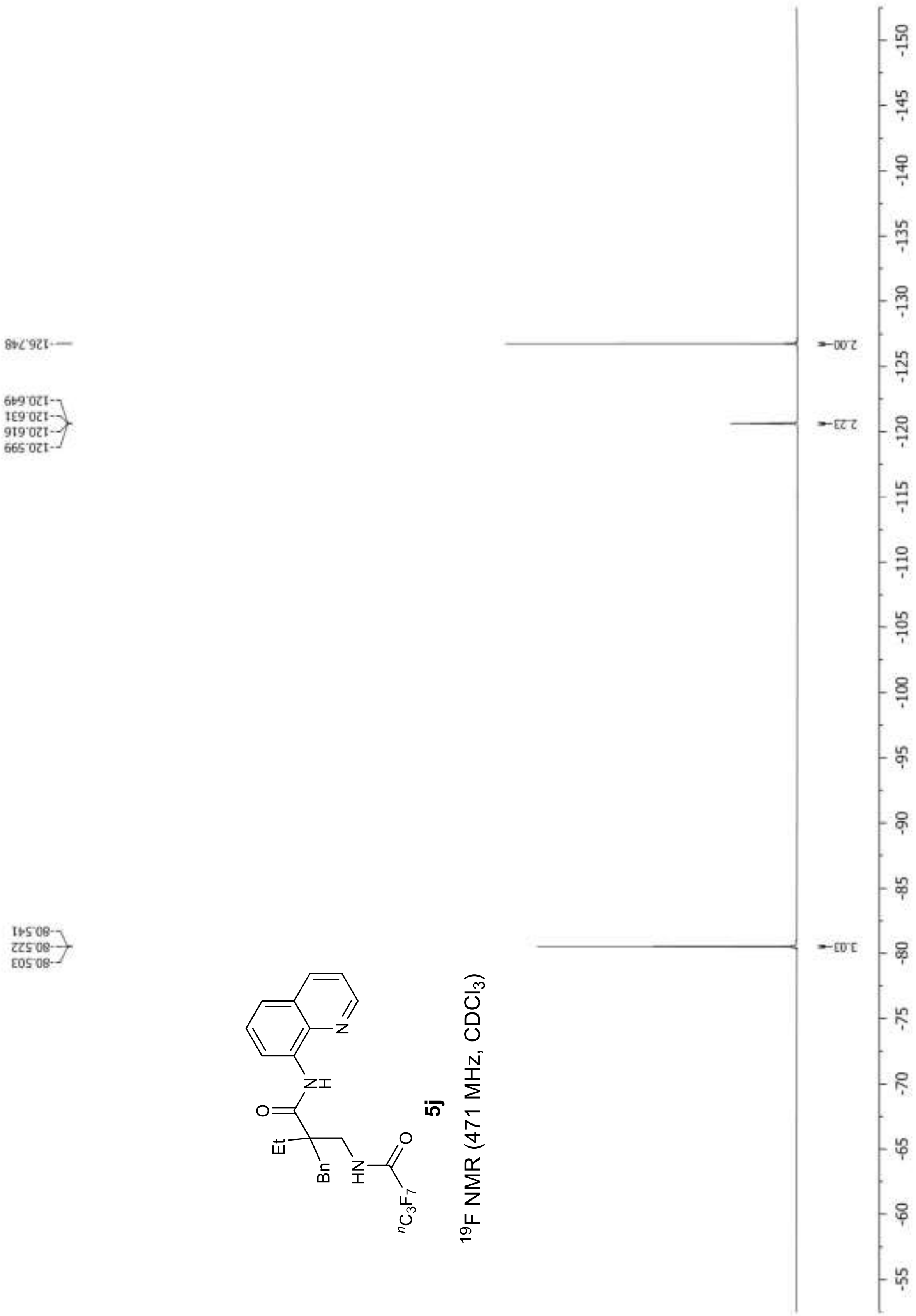

Supplementary Figure 139. ^{19}F NMR (471 MHz, $CDCl_3$) spectrum for 5j.

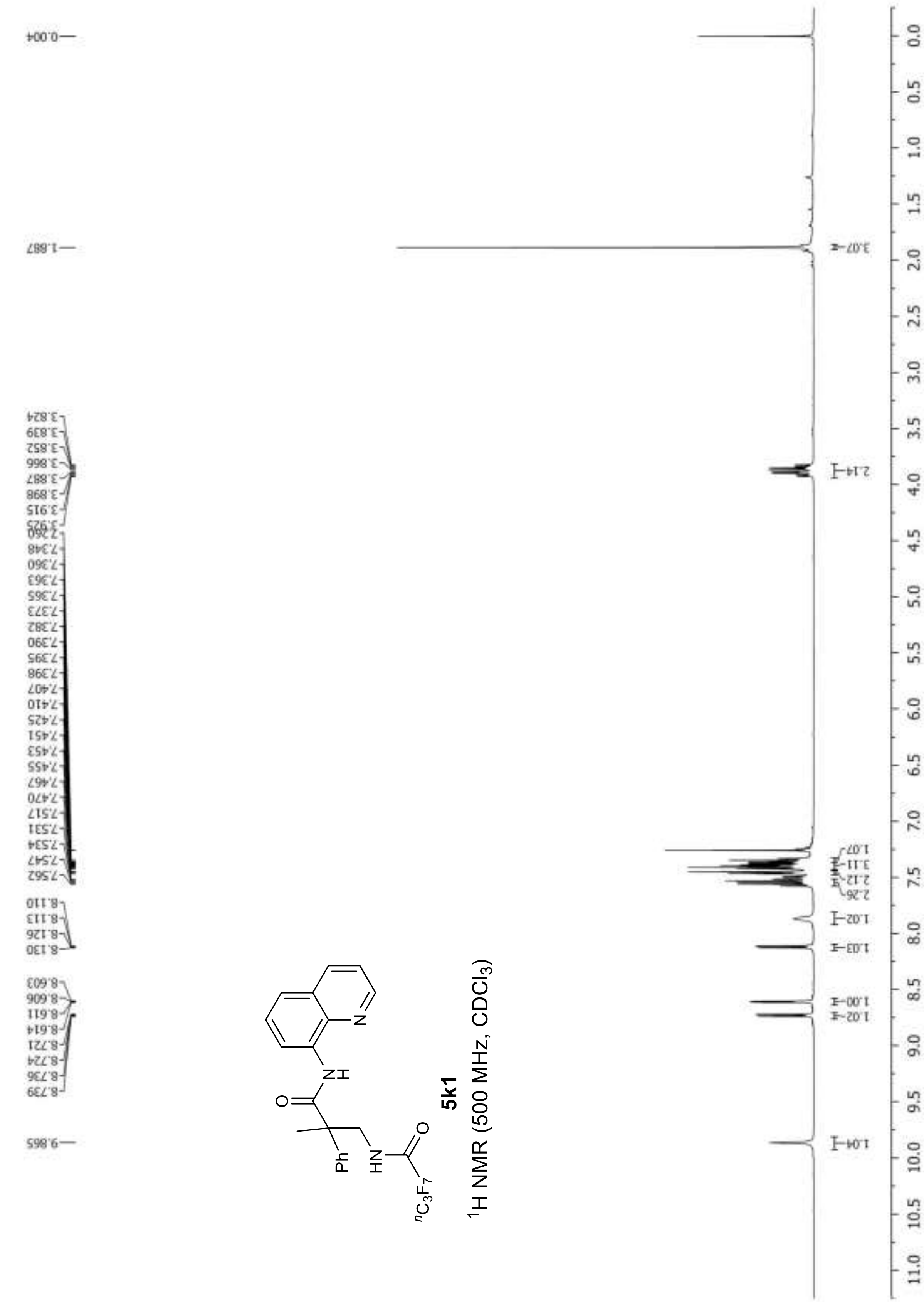

Supplementary Figure 140. ^{1}H NMR (500 MHz, $CDCl_3$) spectrum for 5k1.

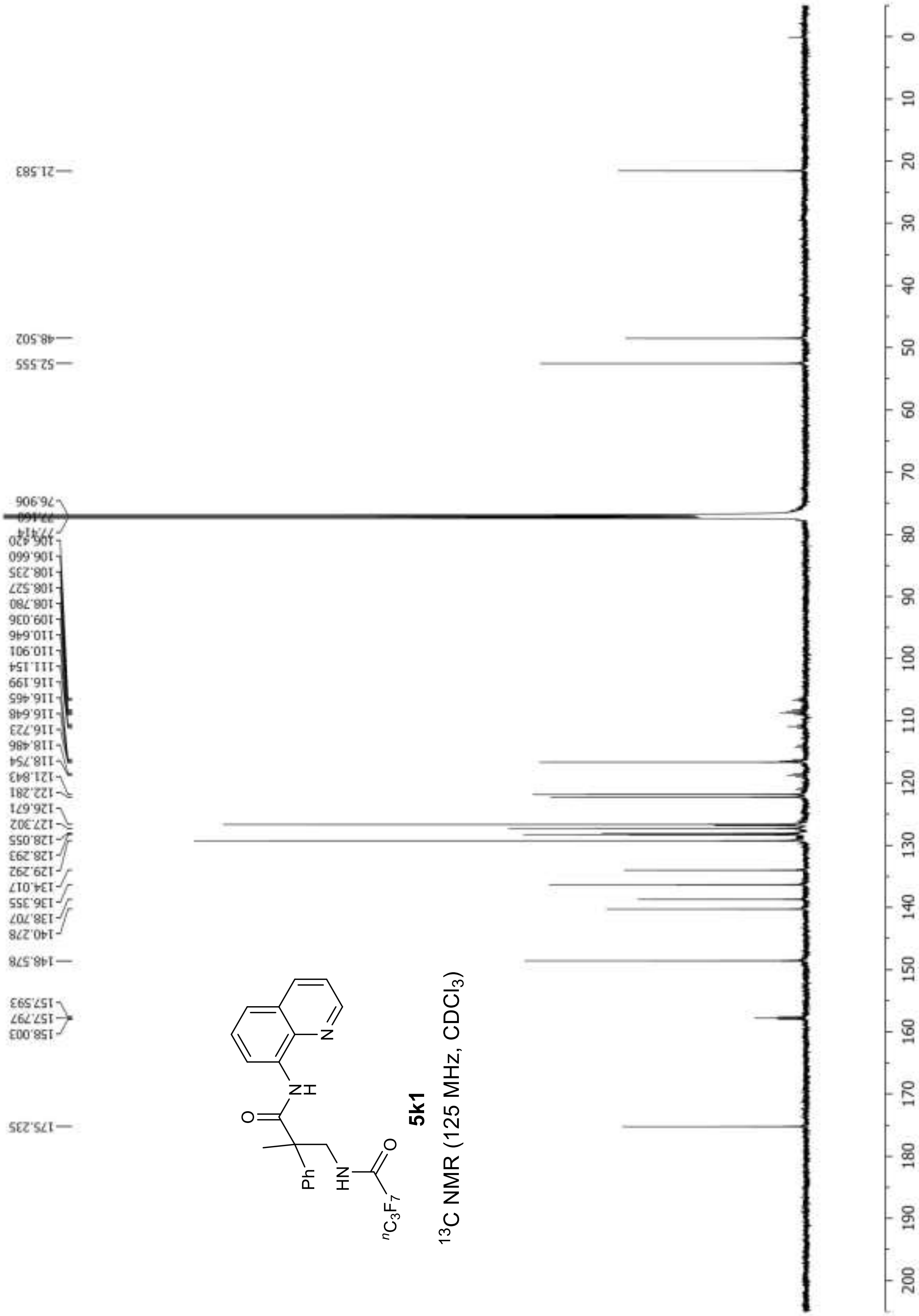

Supplementary Figure 141. ^{13}C NMR (125 MHz, $CDCl_3$) spectrum for 5k1.

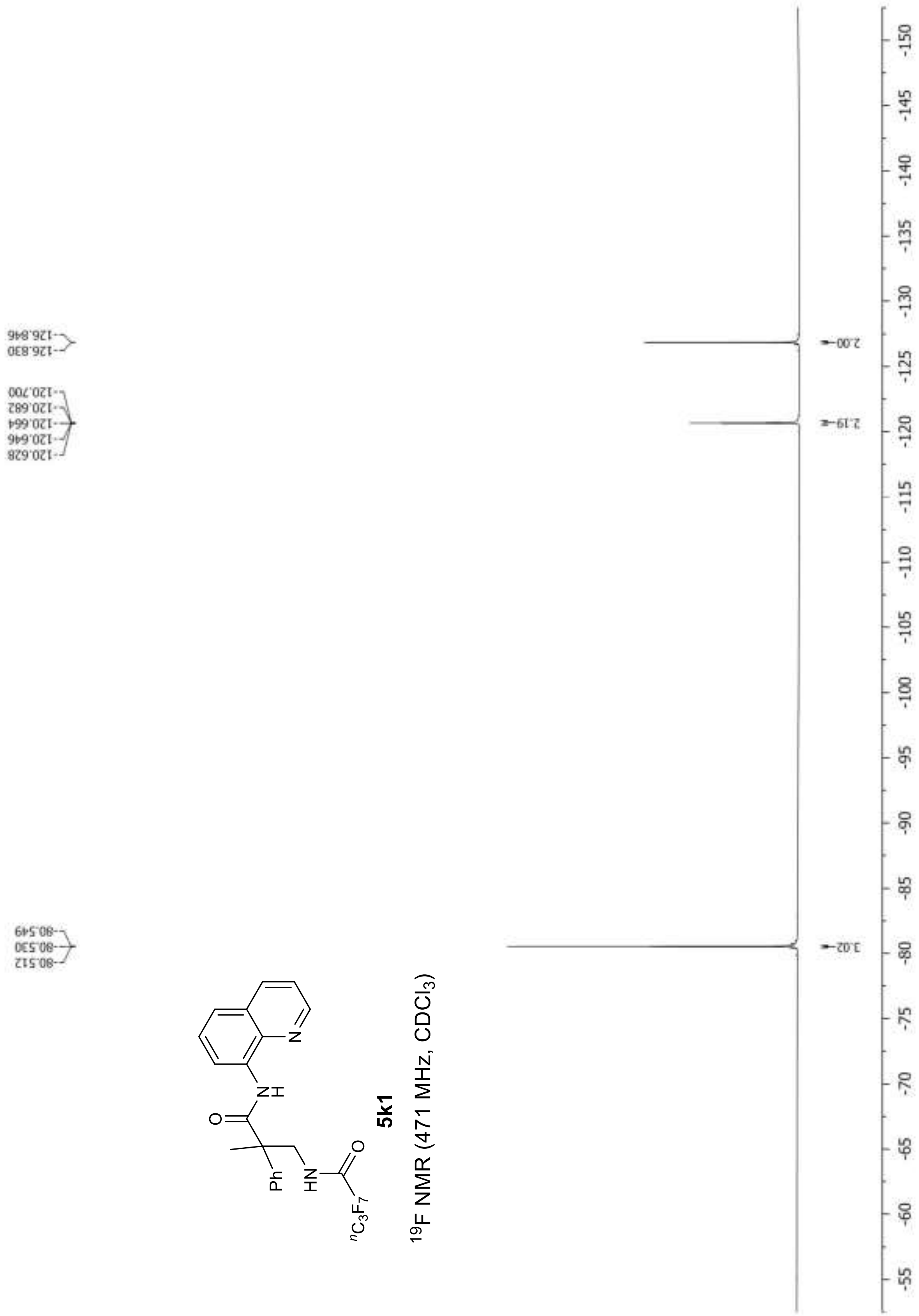

Supplementary Figure 142. ^{19}F NMR (471 MHz, CDCl$_3$) spectrum for 5k1.

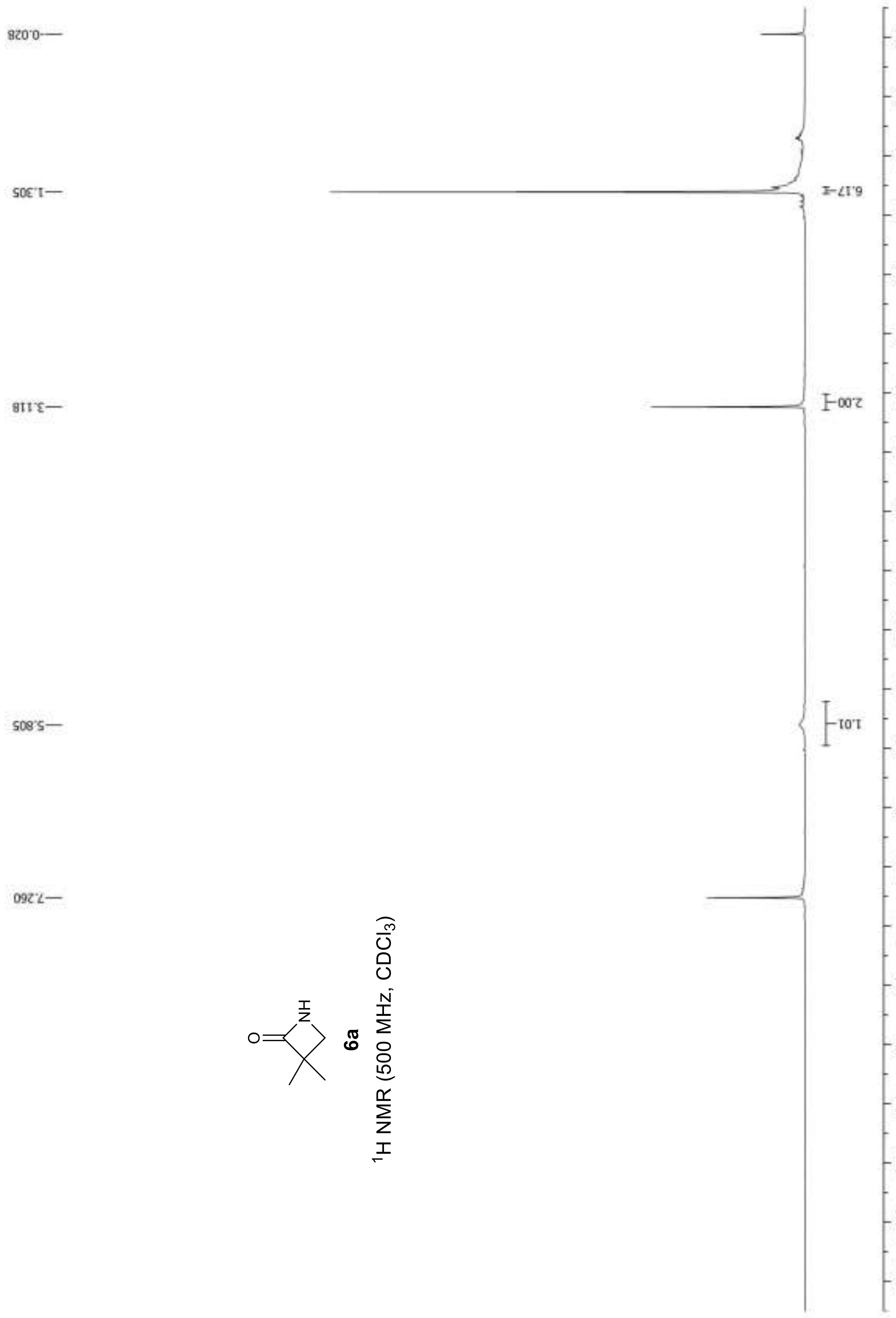

Supplementary Figure 143. ^{1}H NMR (500 MHz, $CDCl_3$) spectrum for 6a.

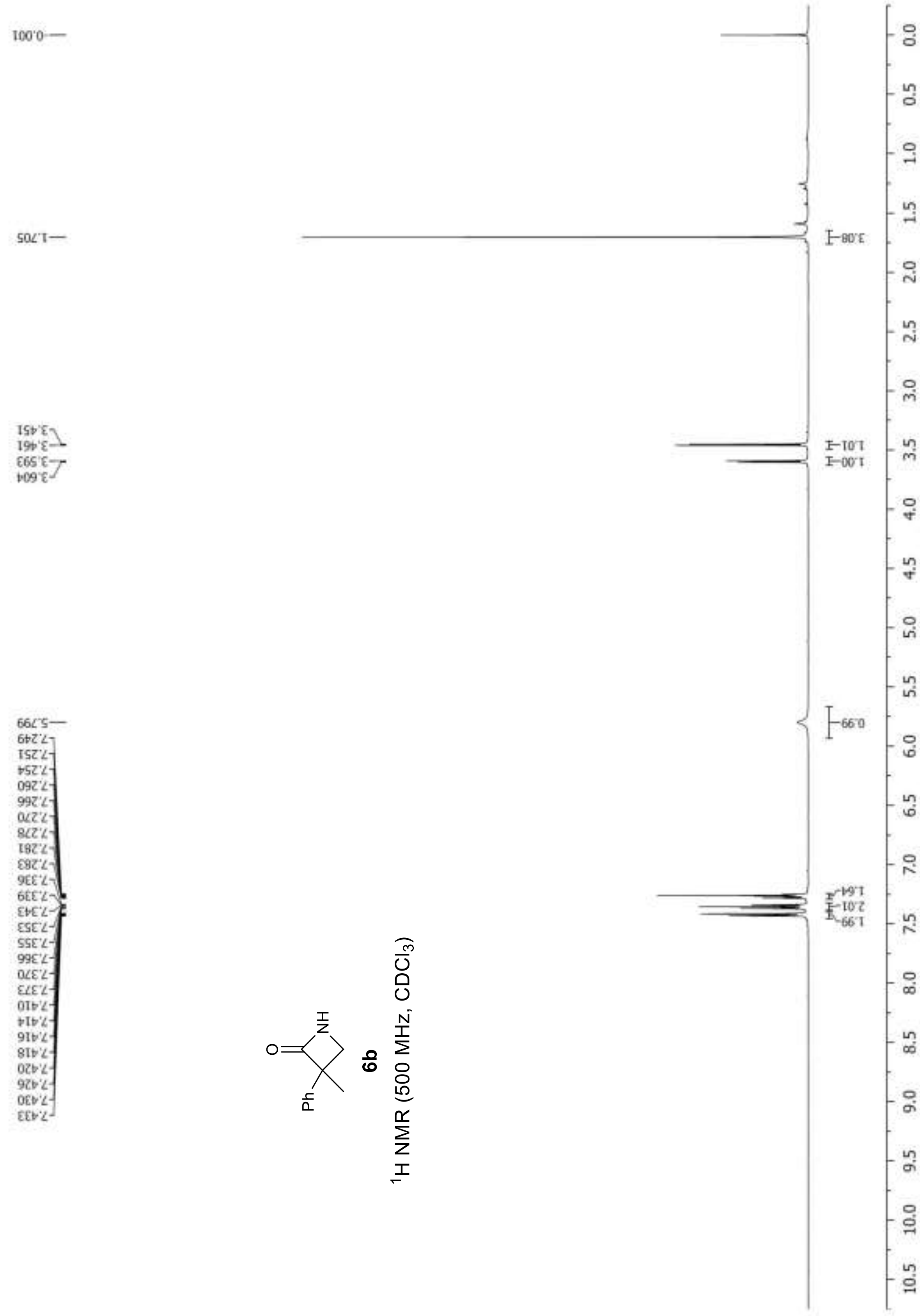

Supplementary Figure 144. ^{1}H NMR (500 MHz, $CDCl_3$) spectrum for 6b.

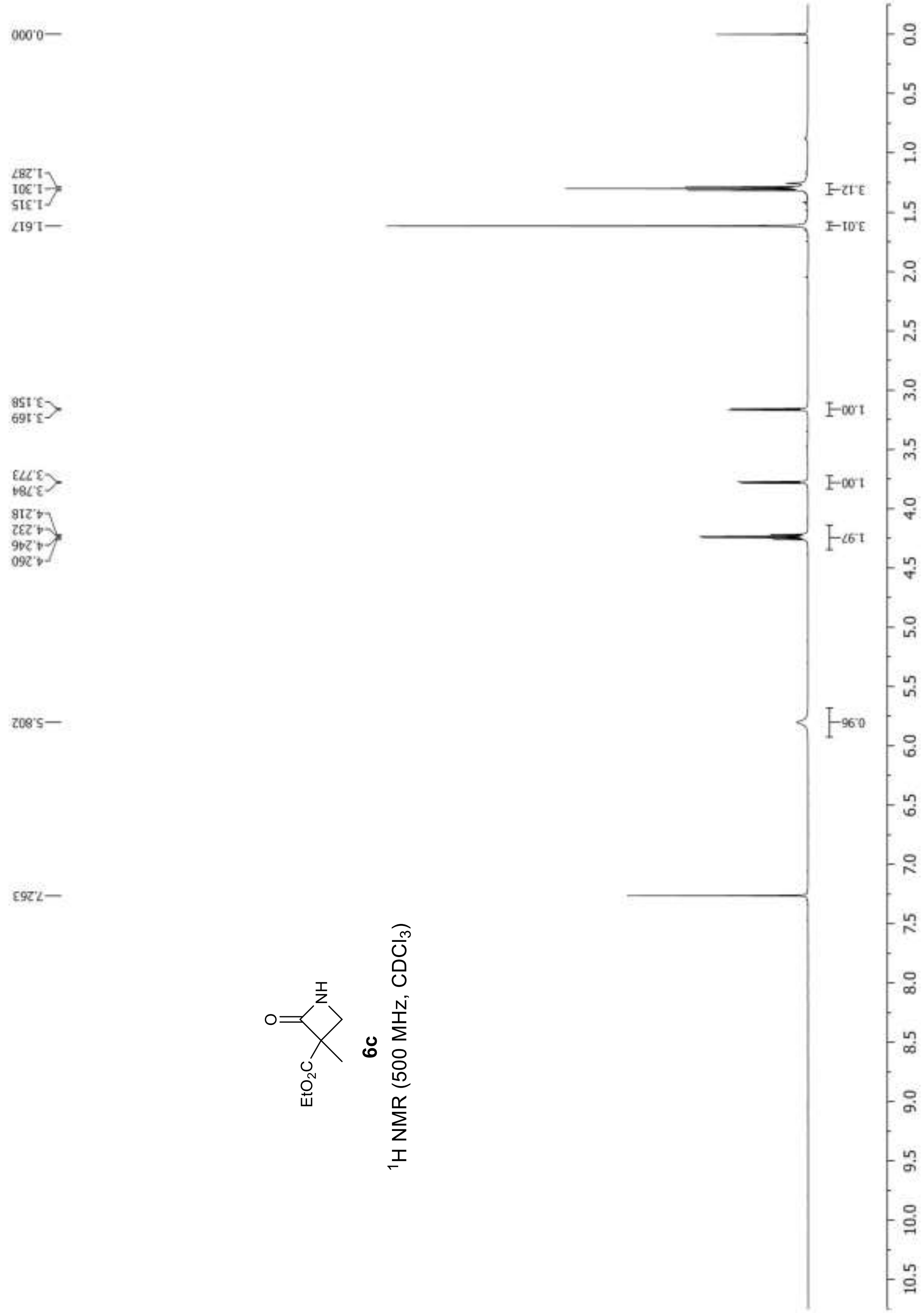

Supplementary Figure 145. ^{1}H NMR (500 MHz, $CDCl_3$) spectrum for 6c.

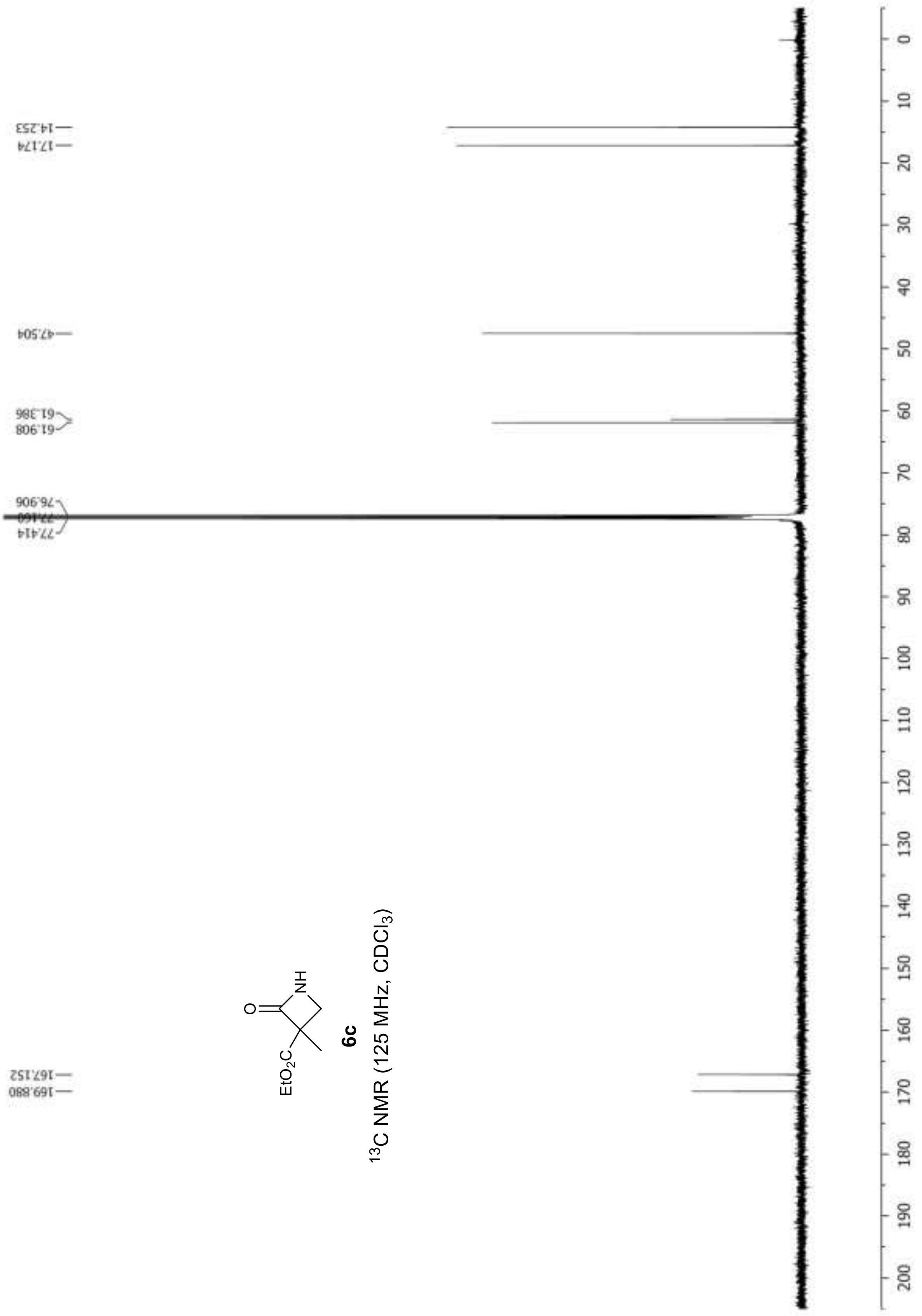

Supplementary Figure 146. ^{13}C NMR (125 MHz, $CDCl_3$) spectrum for 6c.

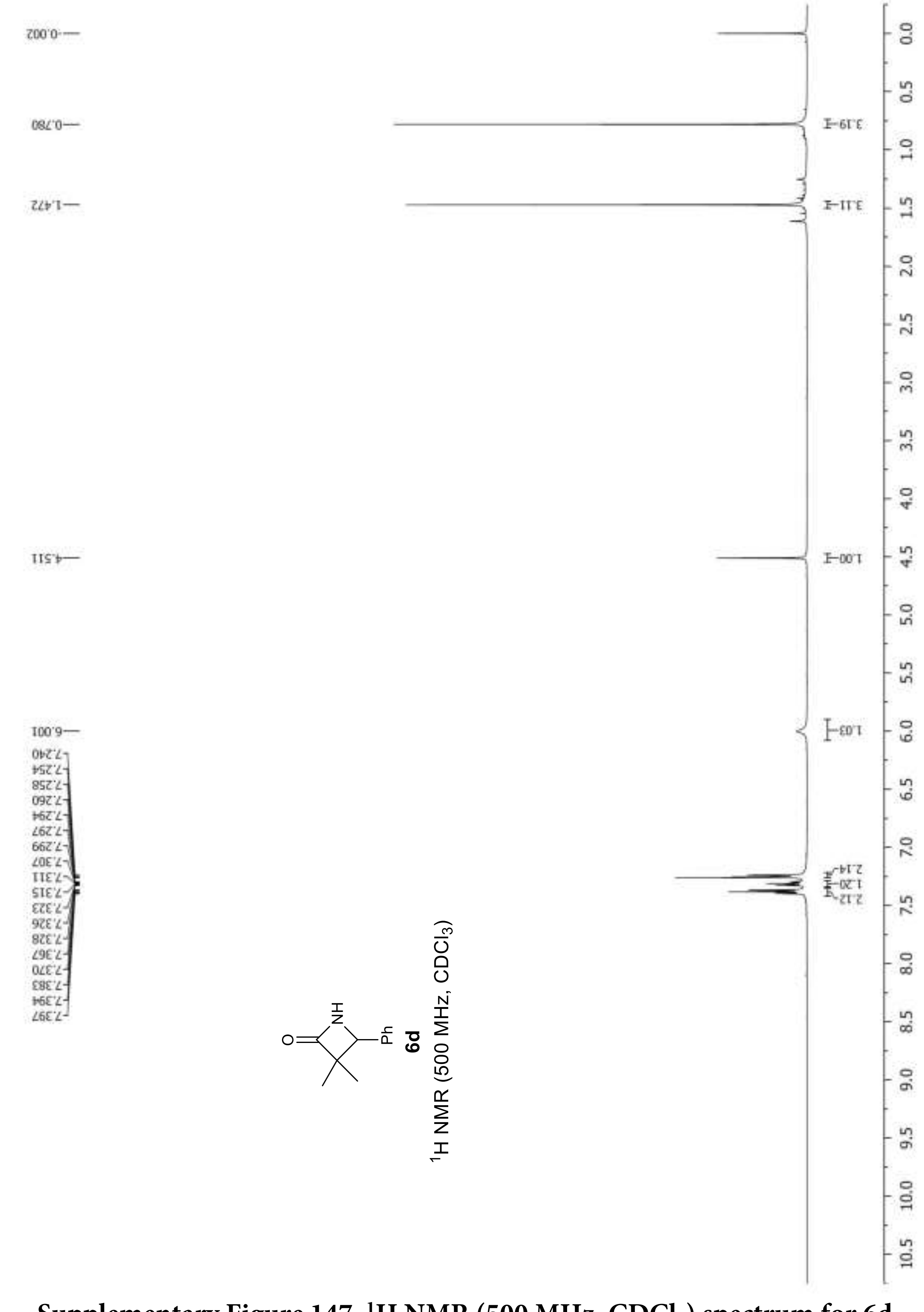

Supplementary Figure 147. ^{1}H NMR (500 MHz, $CDCl_3$) spectrum for 6d.

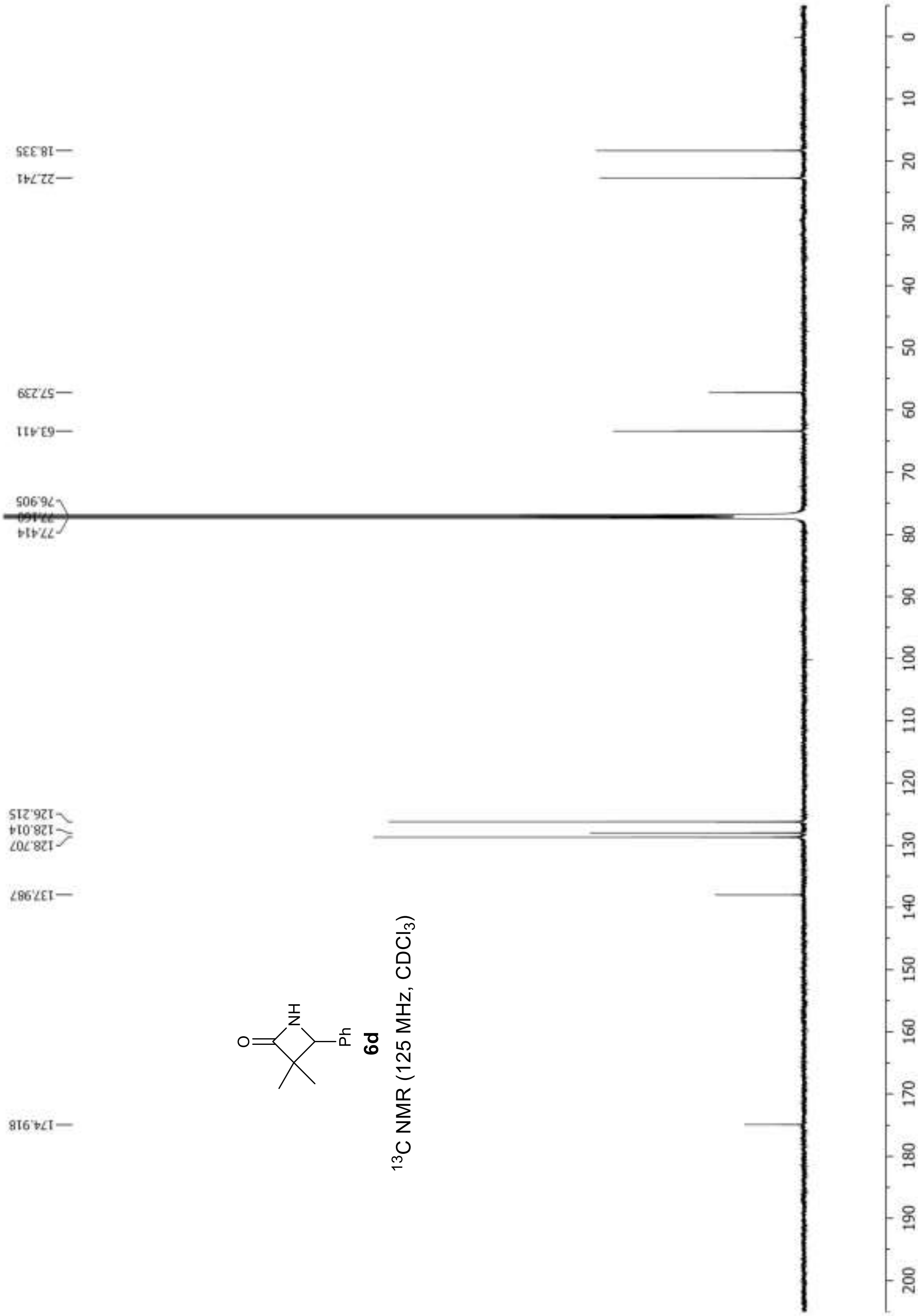

Supplementary Figure 148. ^{13}**C NMR (125 MHz, CDCl**$_3$**) spectrum for 6d.**

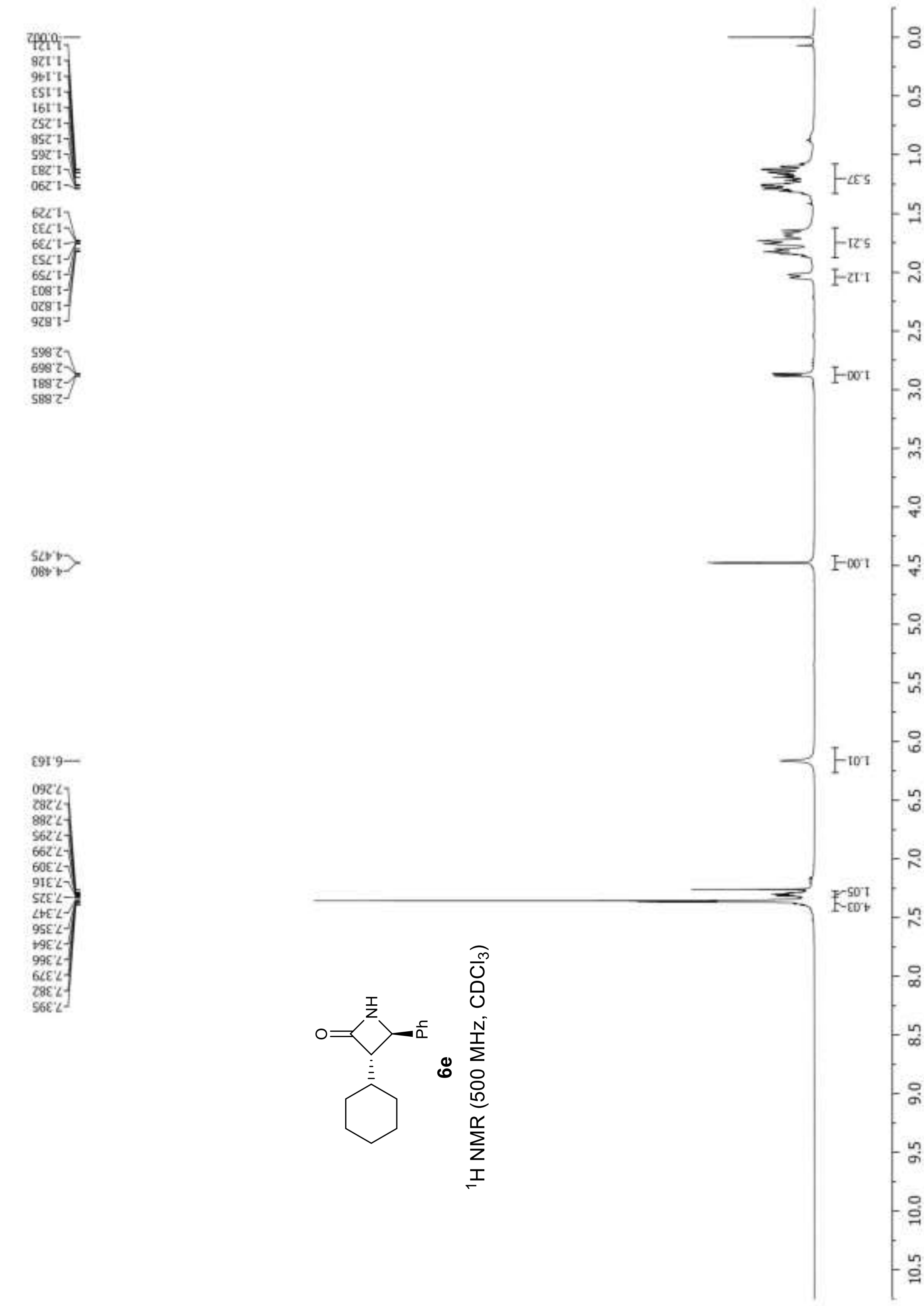

Supplementary Figure 149. ^{1}H NMR (500 MHz, $CDCl_3$) spectrum for 6e.

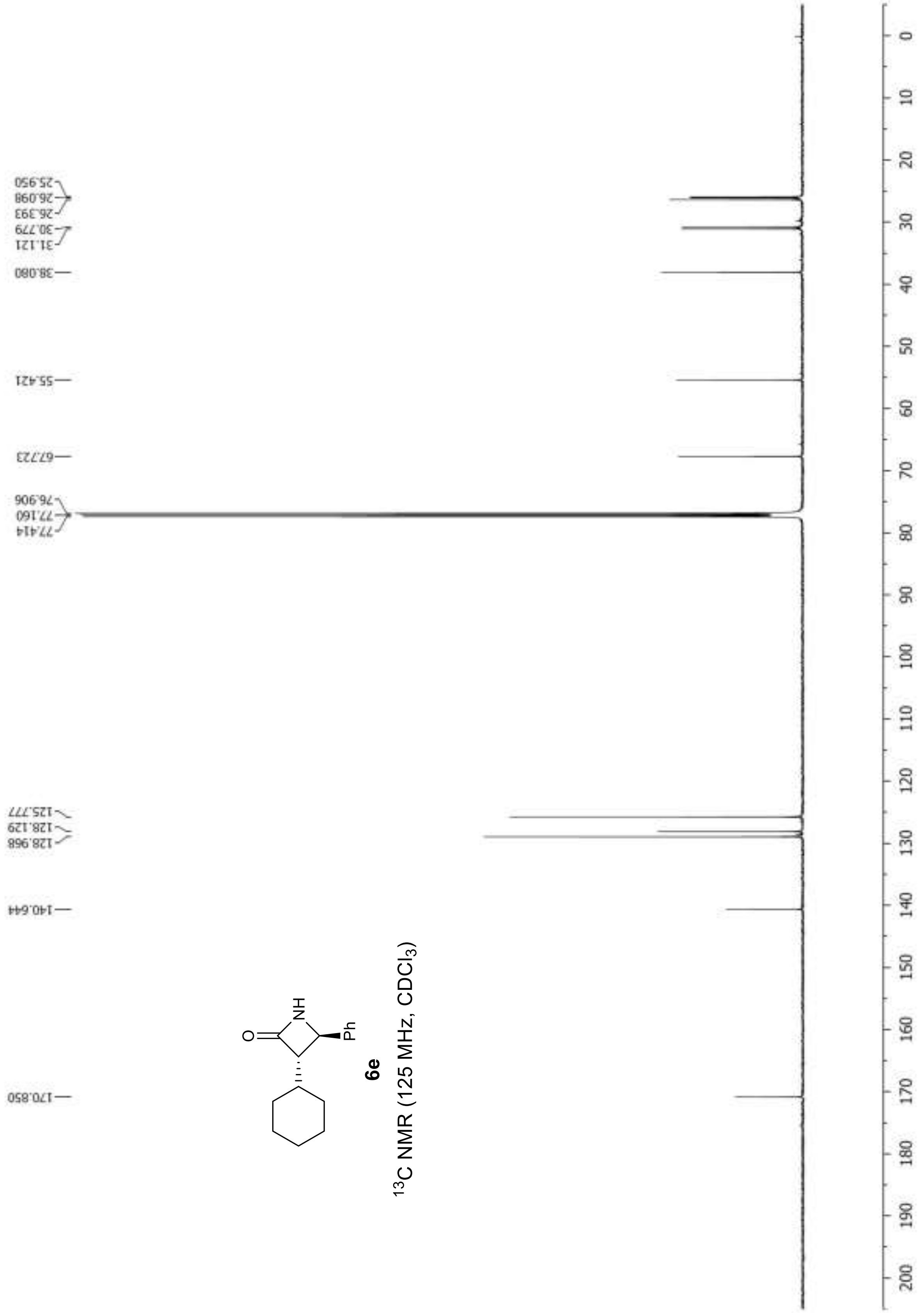

Supplementary Figure 150. ^{13}C NMR (125 MHz, CDCl$_3$) spectrum for 6e.

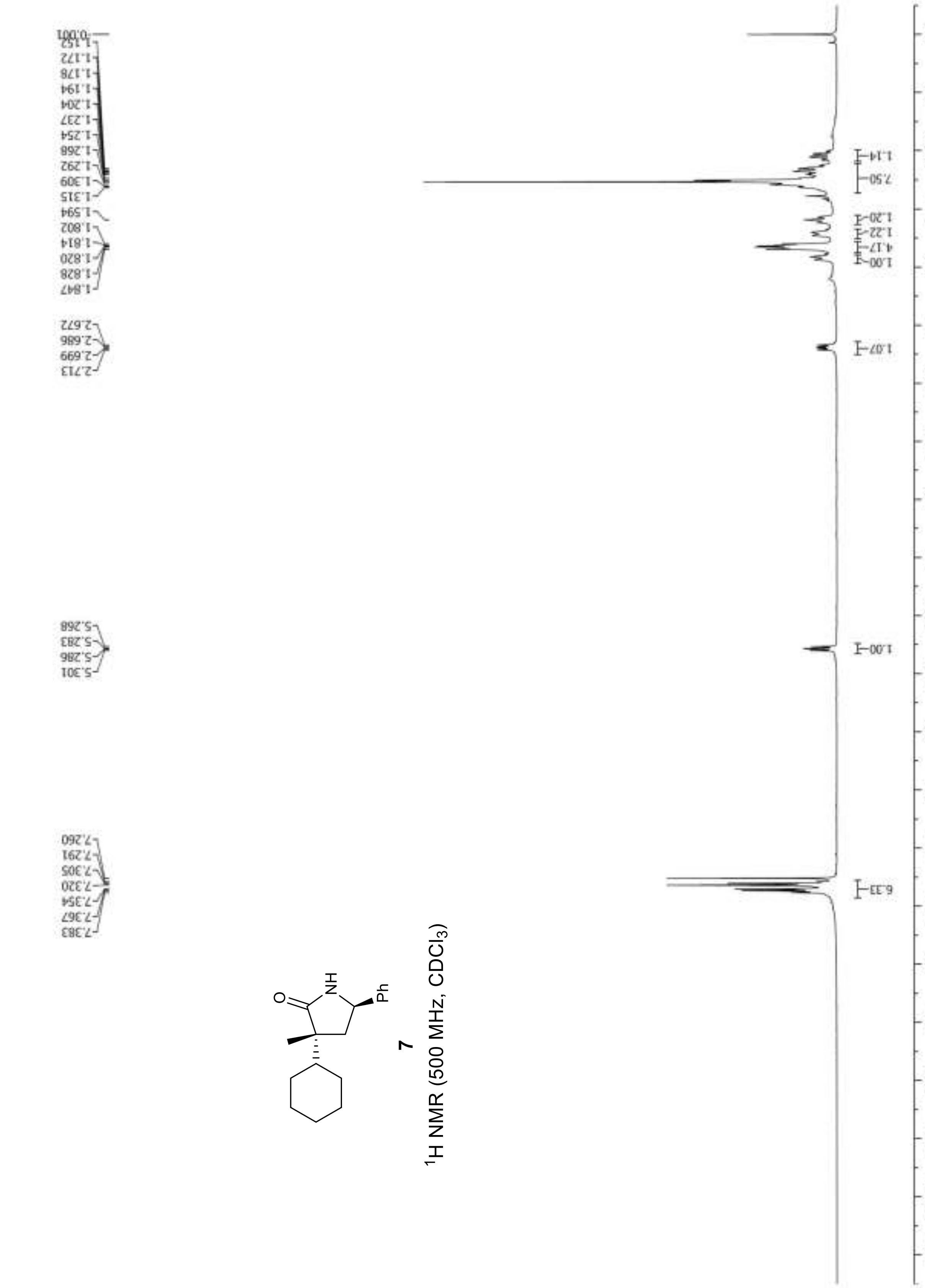

Supplementary Figure 151. ^{1}H NMR (500 MHz, $CDCl_3$) spectrum for 7.

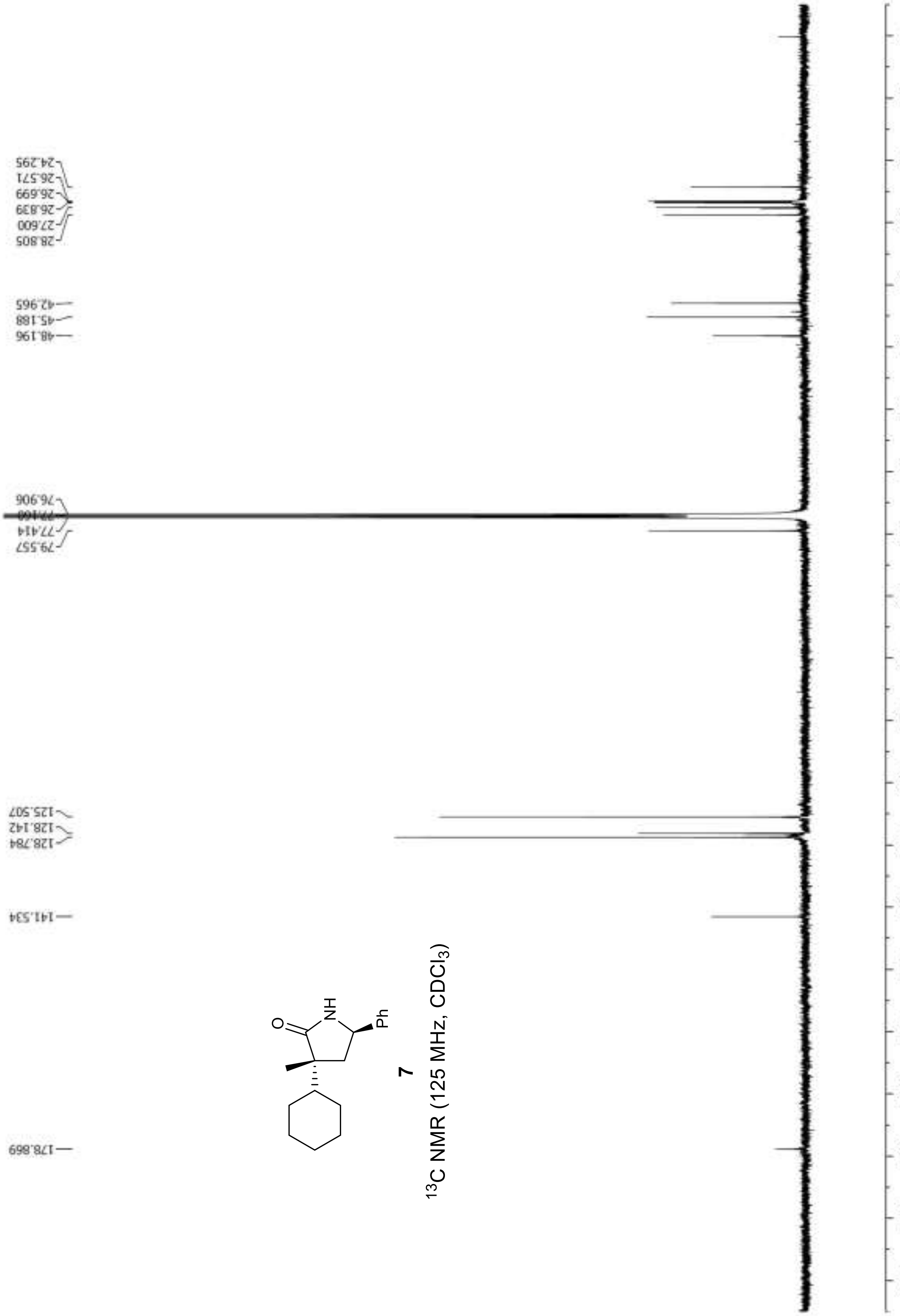

Supplementary Figure 152. ^{13}C NMR (125 MHz, $CDCl_3$) spectrum for 7.

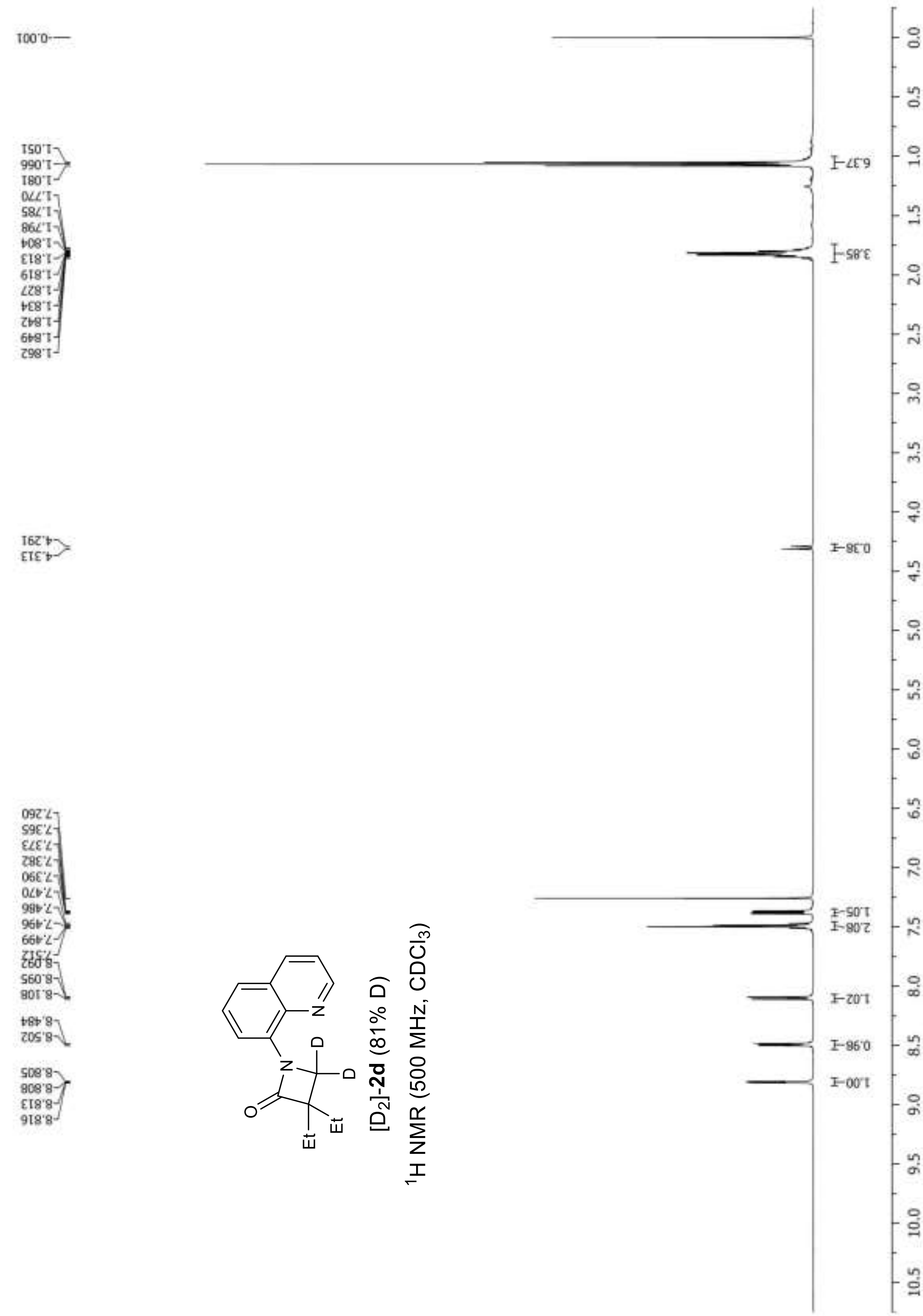

Supplementary Figure 153. ^{1}H NMR (500 MHz, $CDCl_3$) spectrum for [D_2]-2d.

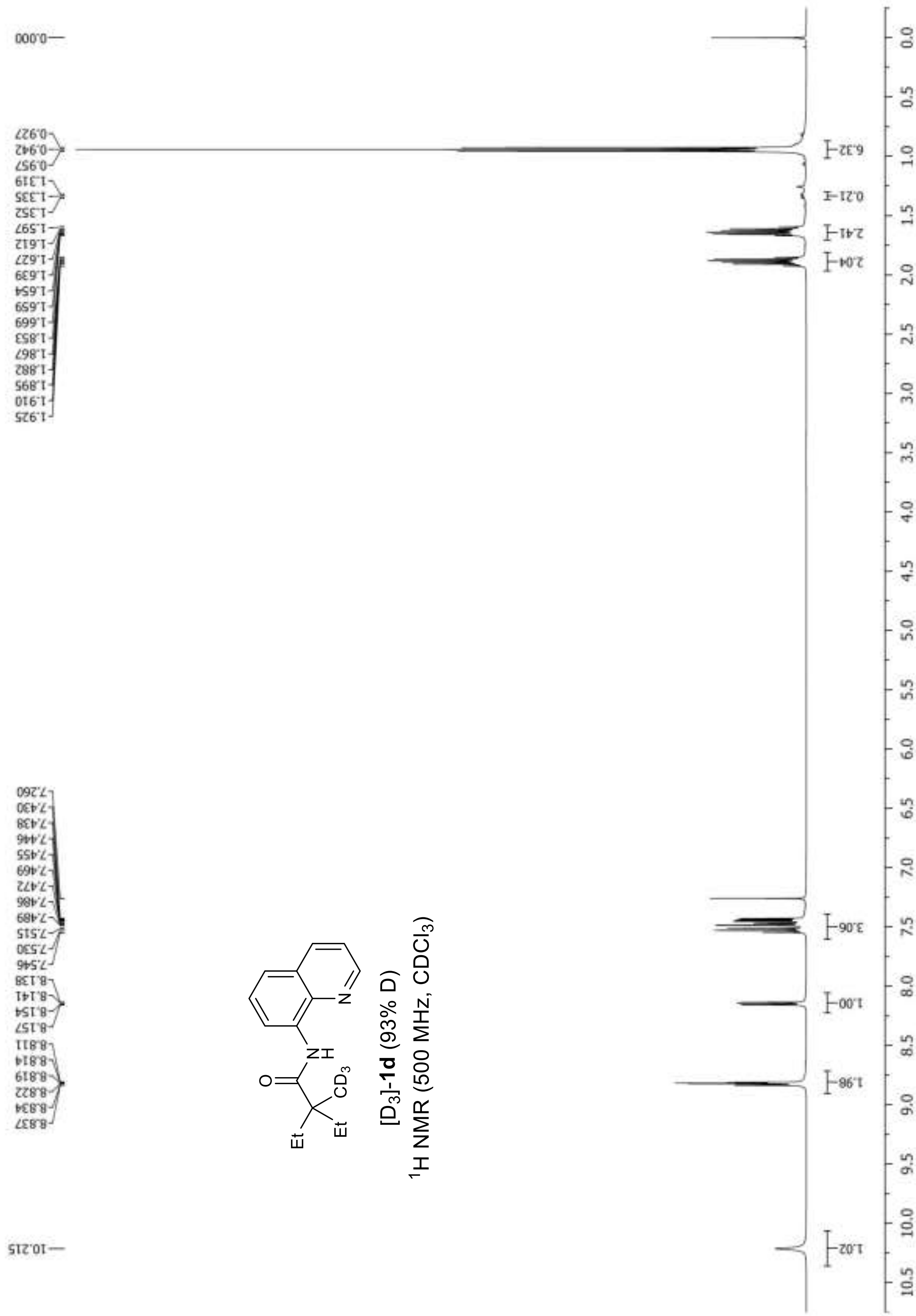

Supplementary Figure 154. ^{1}**H NMR (500 MHz,** $CDCl_3$**) spectrum for [D**$_3$**]-1d.**

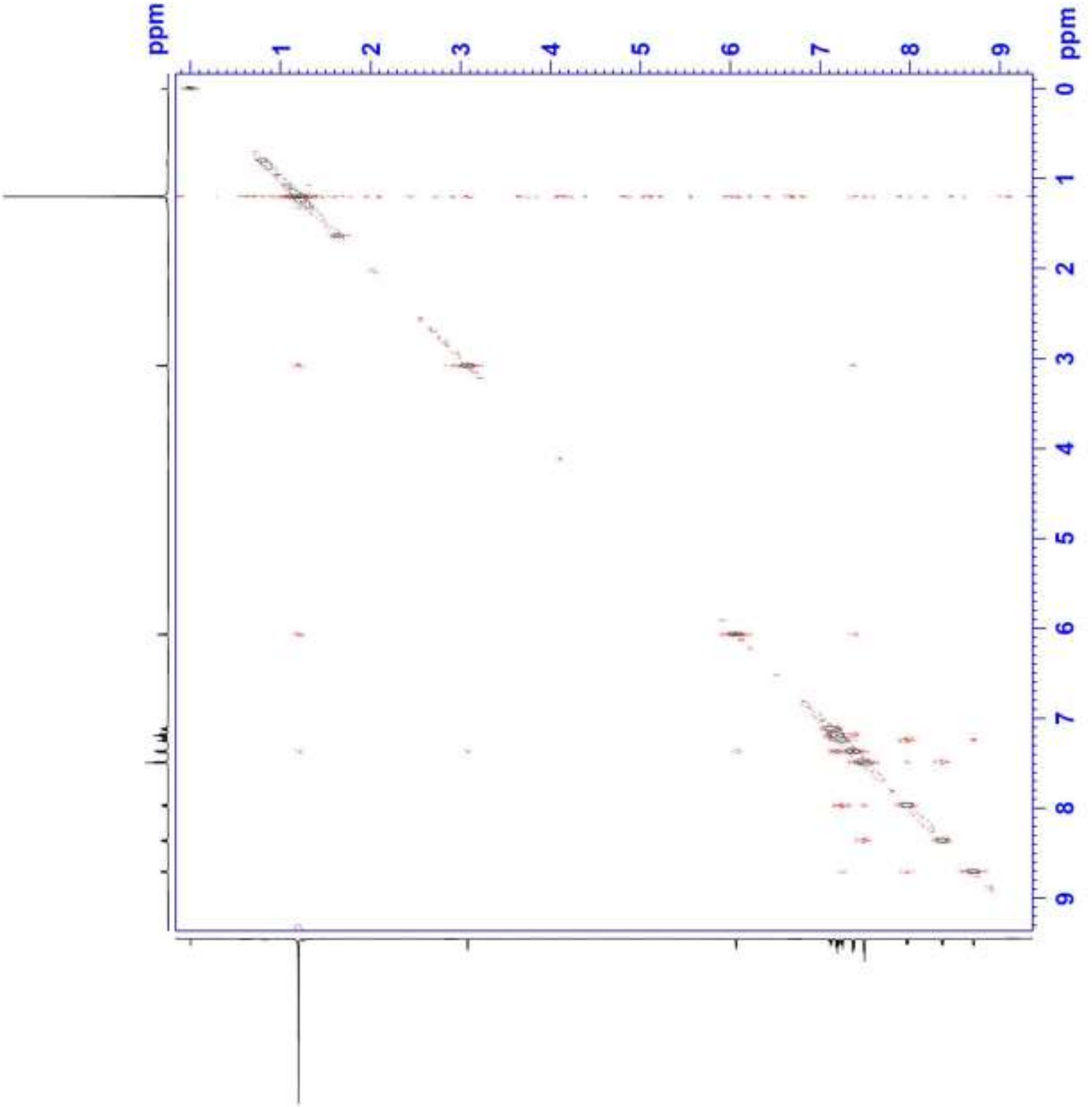

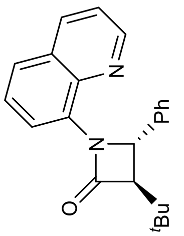

3g
NOESY

Supplementary Figure 155. NOESY spectrum for 3g.

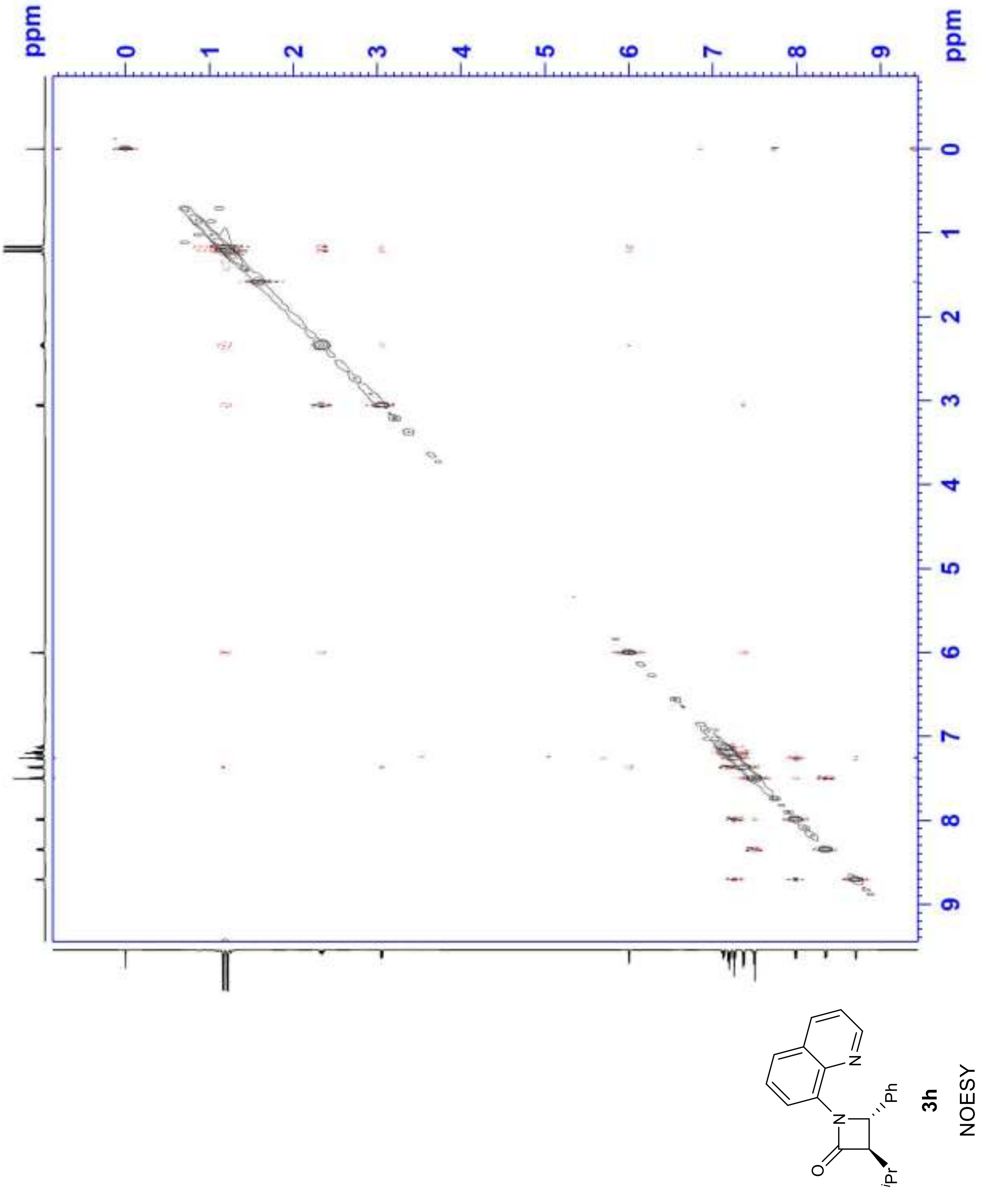

Supplementary Figure 156. NOESY spectrum for 3h.

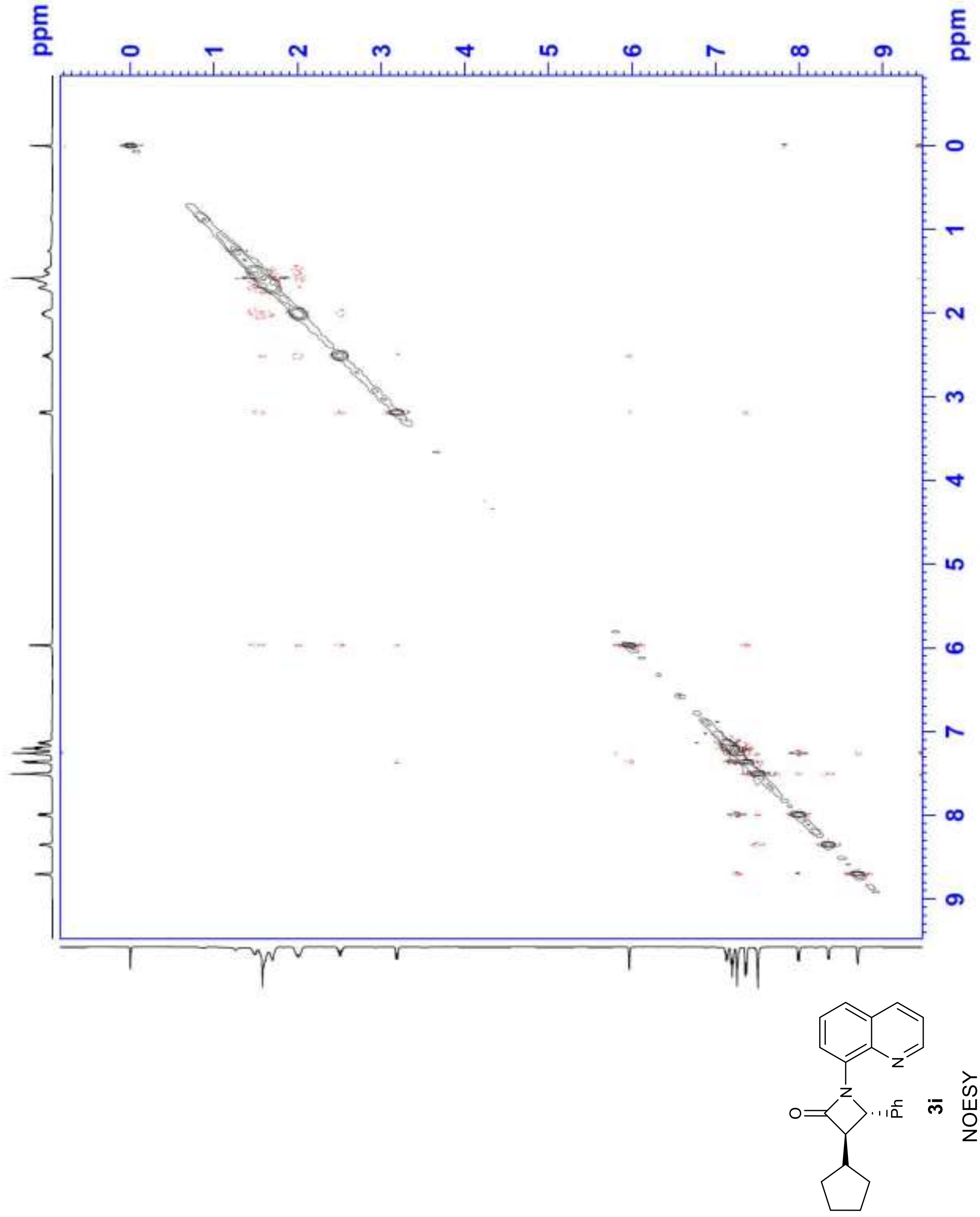

Supplementary Figure 157. NOESY spectrum for 3i.

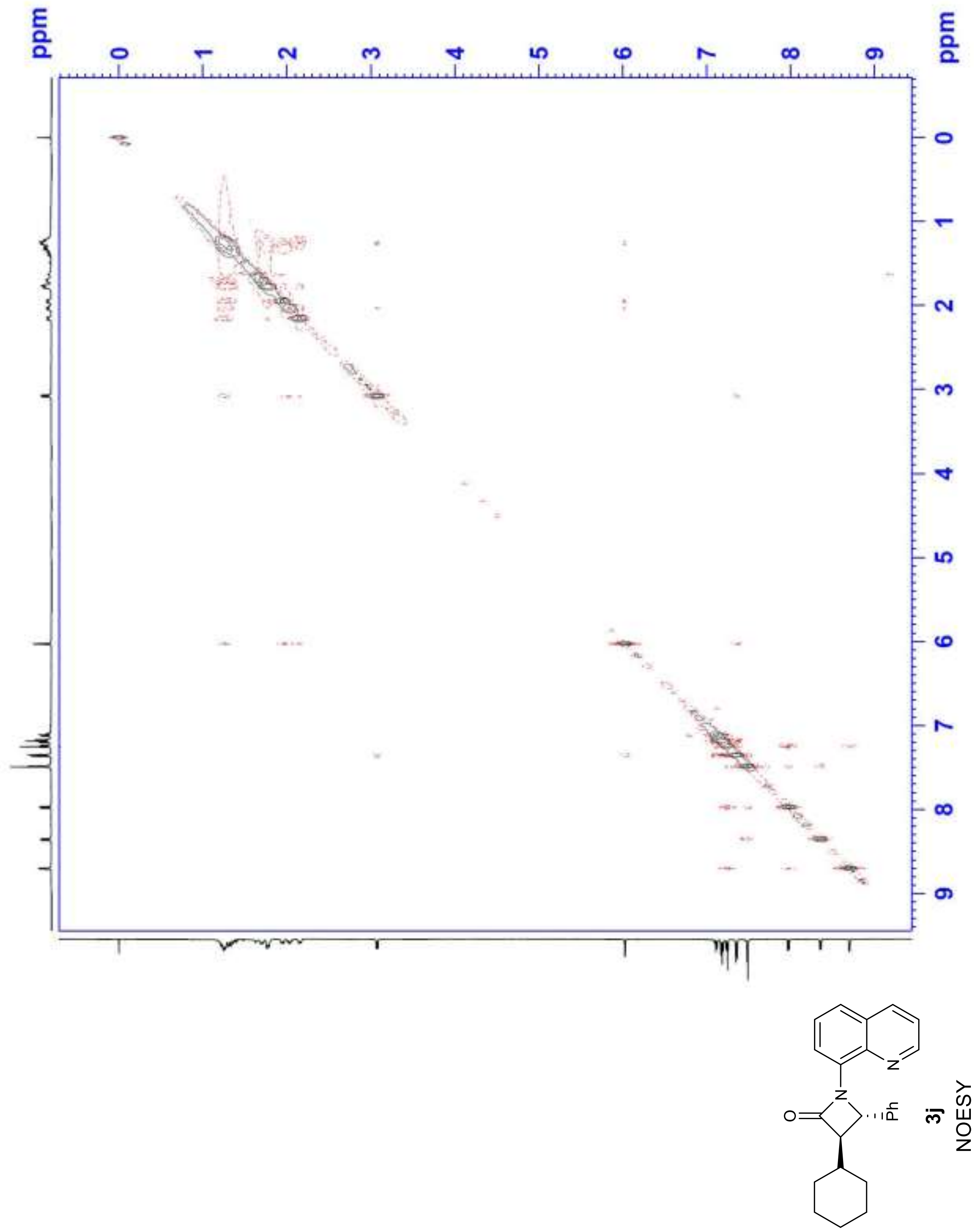

Supplementary Figure 158. NOESY spectrum for 3j.

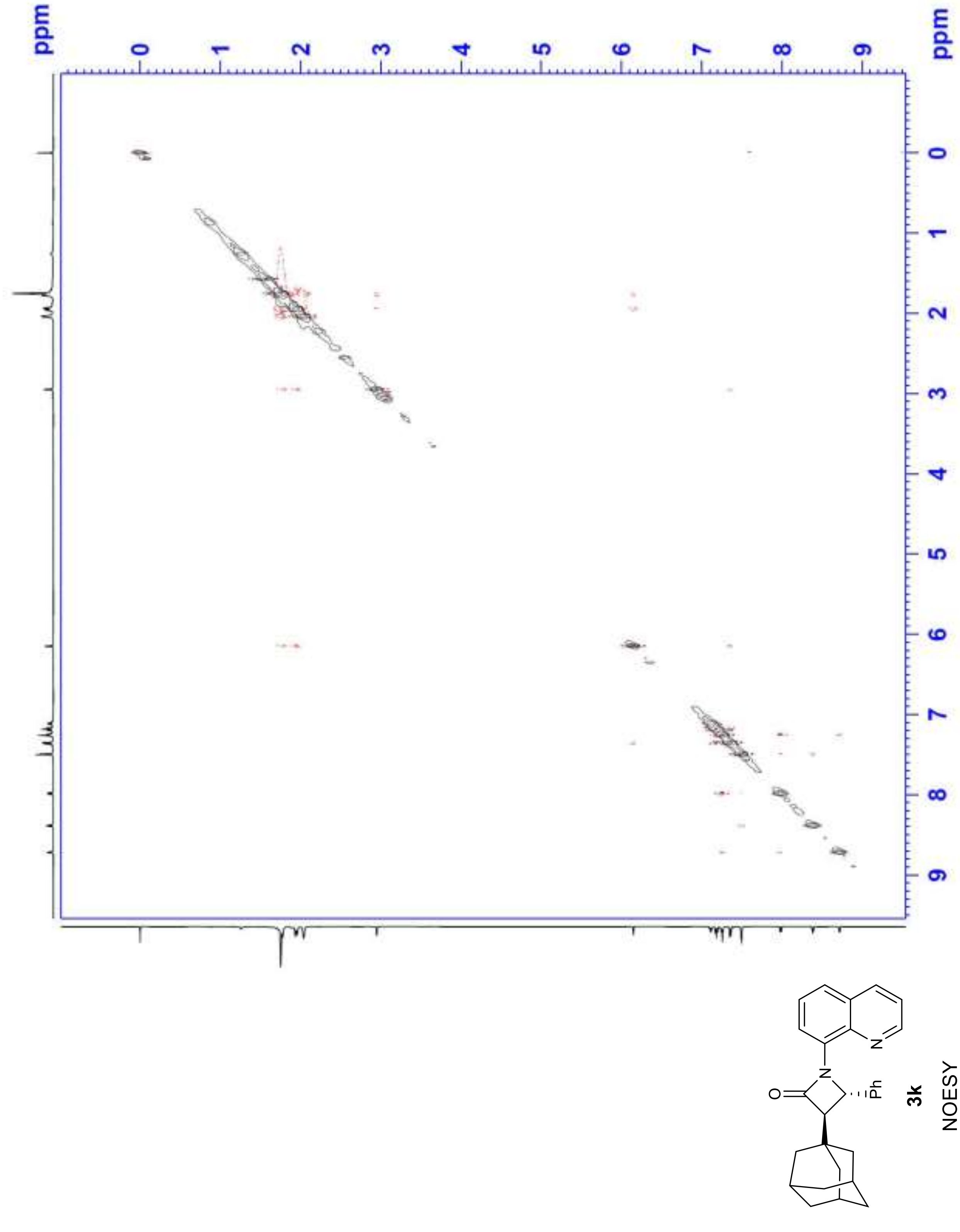

Supplementary Figure 159. NOESY spectrum for 3k.

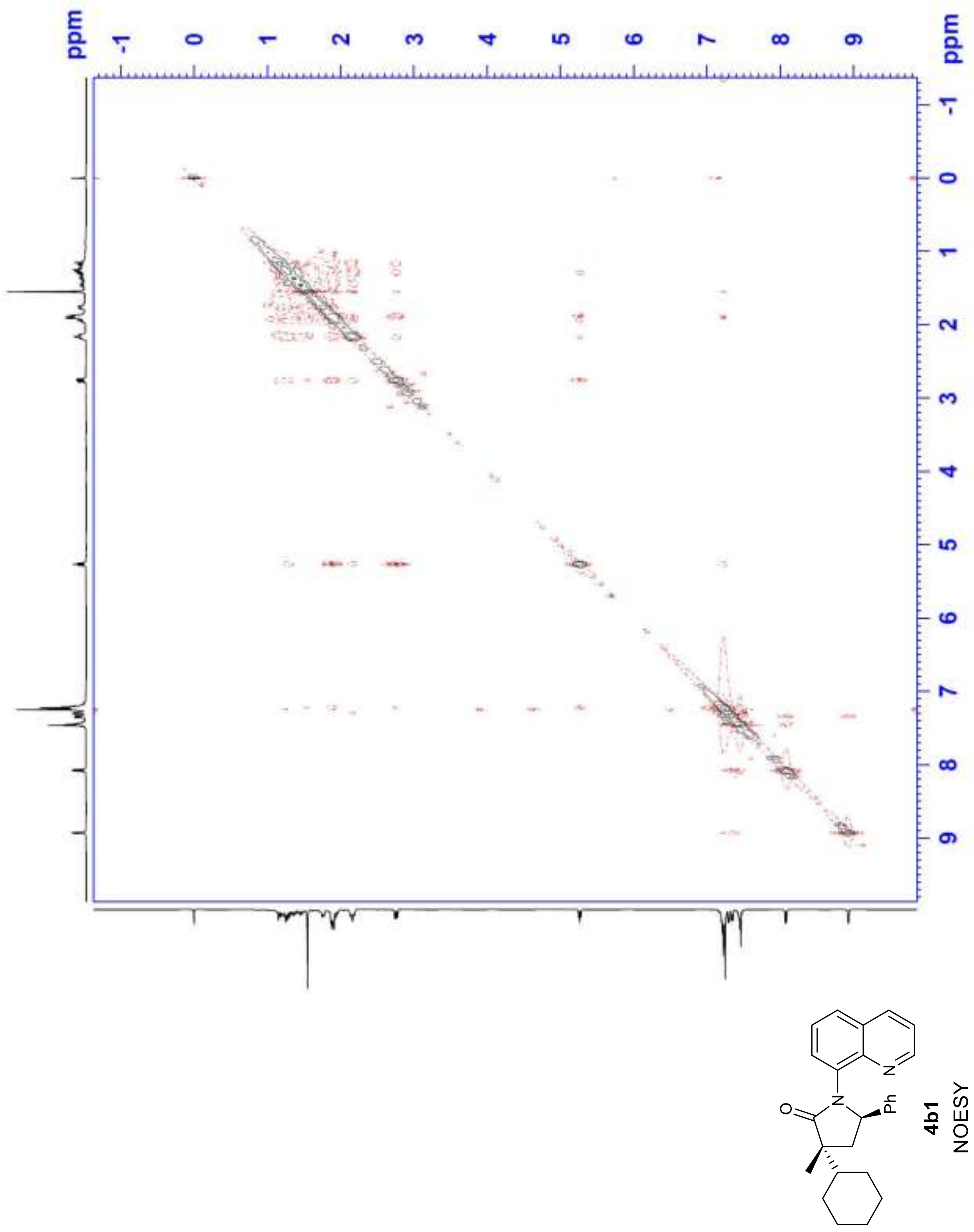

Supplementary Figure 160. NOESY spectrum for 4b1.

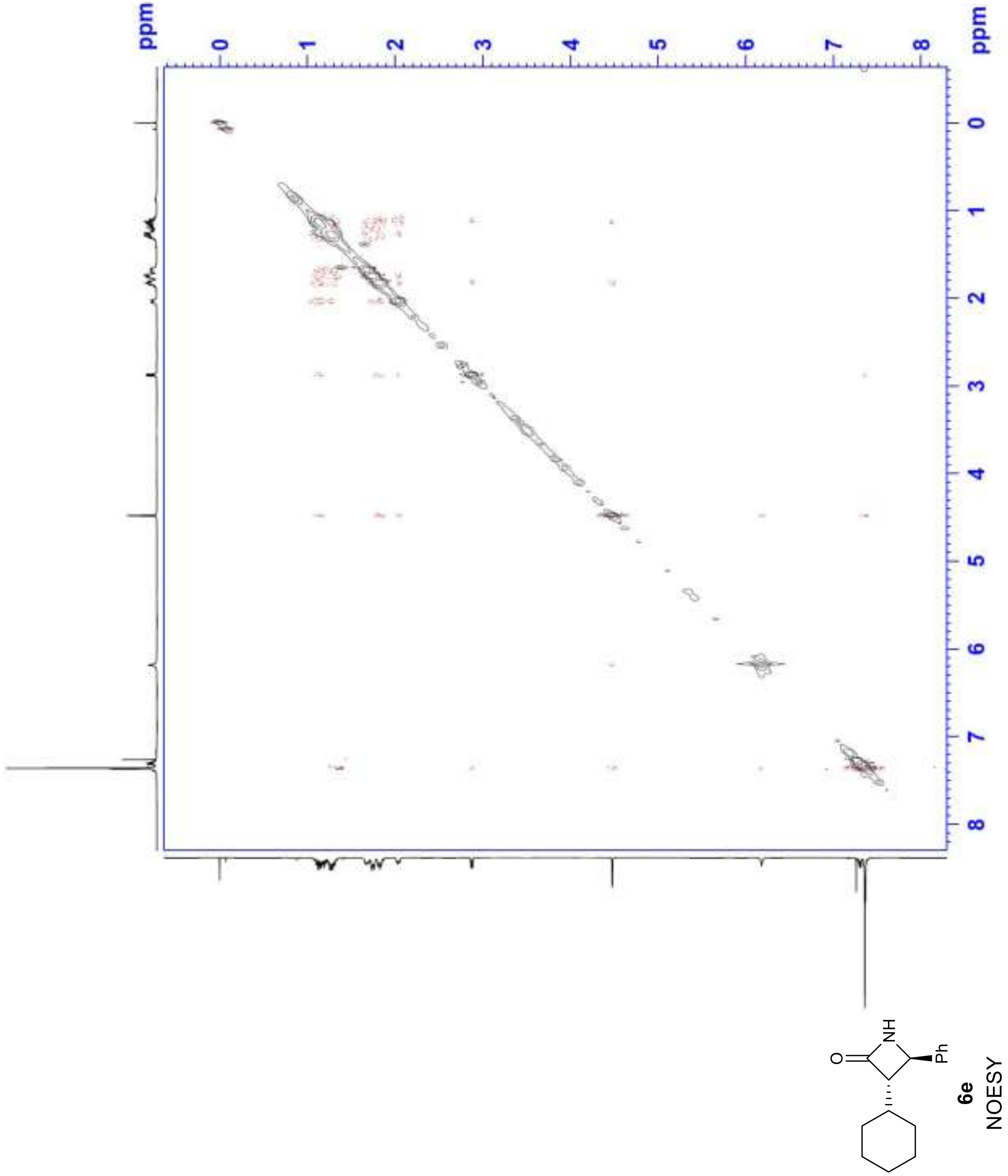

Supplementary Figure 161. NOESY spectrum for 6e.

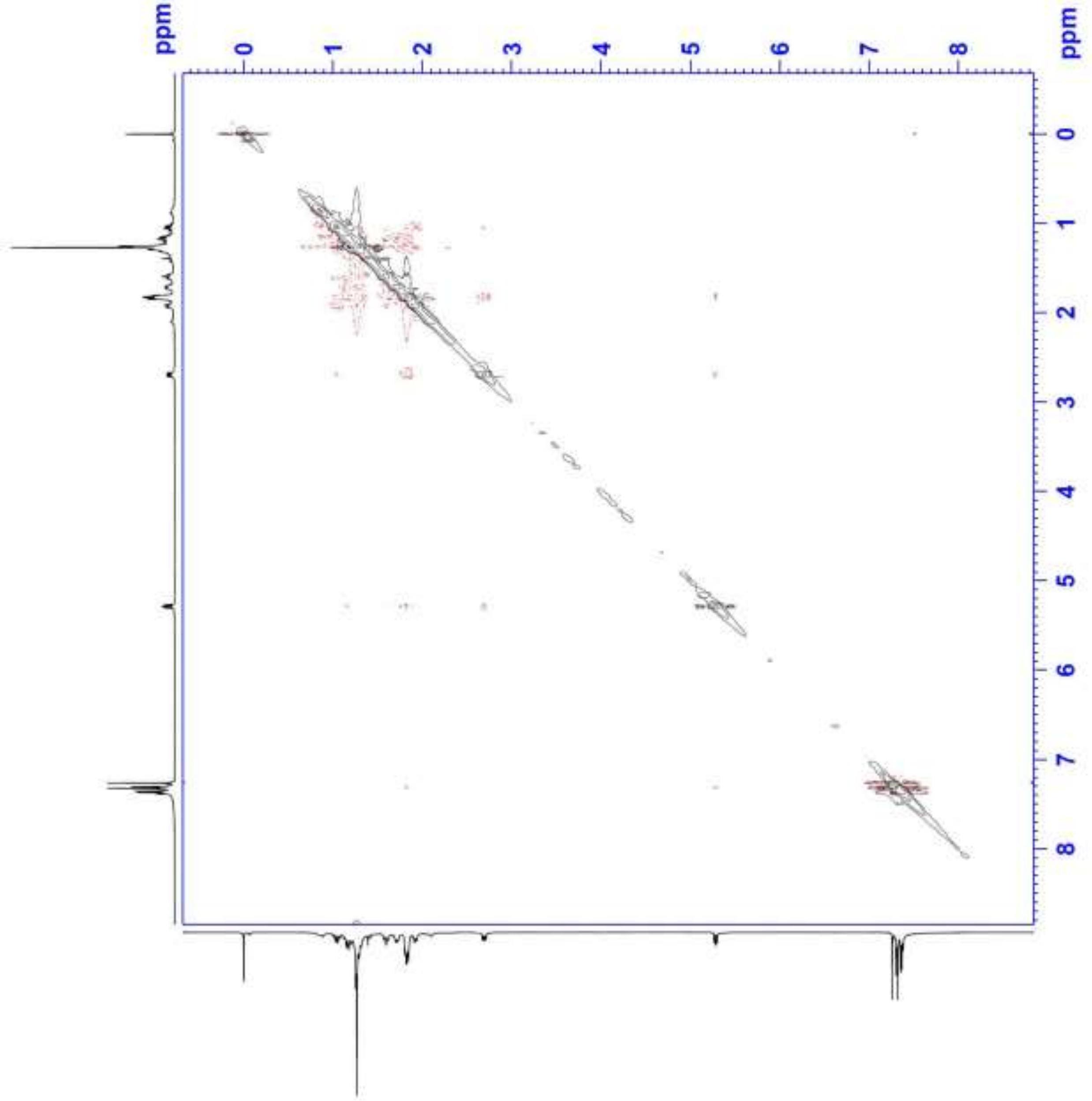

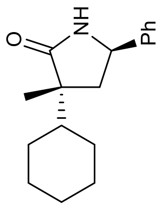

7
NOESY

Supplementary Figure 162. NOESY spectrum for 7.

Supplementary Tables

Supplementary Table 1. Control Experiments of γ–Lactam Formation

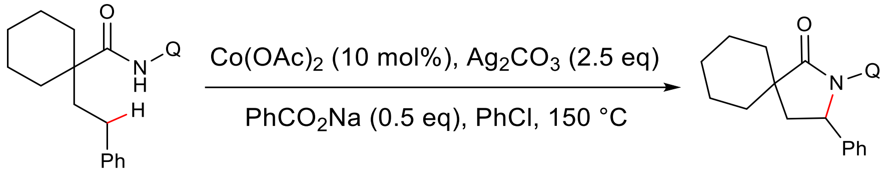

Entry	Change from the "standard conditions"[a]	Yield of **4c** (%)[b]
1	none	87
2	no $Co(OAc)_2$	8
3	Shi's conditions[c] instead of the standard conditions	0
4	$Co(OAc)_2$ (1 eq), no Ag_2CO_3	0
5	$Ce(SO_4)_2$ (6 eq) instead of Ag_2CO_3	5
6	addition of TEMPO (1 eq)	27
7	addition of TEMPO (2 eq)	7

[a]Reaction conditions: **1an** (0.3 mmol), $Co(OAc)_2$ (0.03 mmol), Ag_2CO_3 (0.75 mmol), $PhCO_2Na$ (0.15 mmol), 0.6 mL PhCl, 150 °C, 24 h.

[b]Yields are based on **1an**, determined by ^{1}H-NMR using dibromomethane as the internal standard.

[c]Shi's conditions: see Table 2.

Supplementary Table 2. Optimization of Reaction Conditions for Cobalt-Catalyzed Intermolecular Amination of α,α–Disubstituted Propanamides

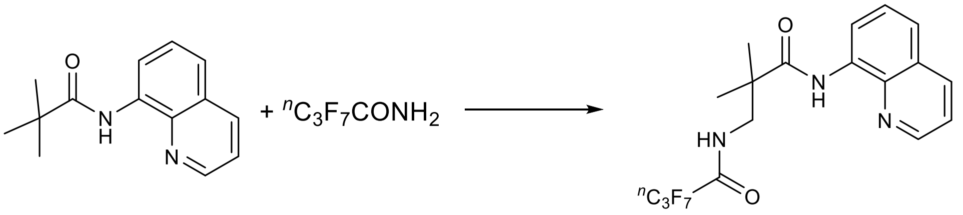

Entry	Co source (mol%)	Oxidant (equiv)	Base (equiv)	Additive	Solvent	Yield (%)[b]
1	$Co(OAc)_2$ (20)	Ag_2CO_3	K_2HPO_4	4A MS	DMF	0
2	$Co(OAc)_2$ (20)	Ag_2CO_3	K_2HPO_4	4A MS	dioxane	0
3	$Co(OAc)_2$ (20)	Ag_2CO_3	K_2HPO_4	4A MS	t-BuOH	0
4	$Co(OAc)_2$ (20)	Ag_2CO_3	K_2HPO_4	4A MS	toluene	<5
5	$Co(OAc)_2$ (20)	Ag_2CO_3	K_2HPO_4	4A MS	o-xylene	<5
6	$Co(OAc)_2$ (20)	Ag_2CO_3	K_2HPO_4	4A MS	PhCl	<5
7	$Co(OAc)_2$ (20)	Ag_2CO_3	K_2HPO_4	4A MS	$PhCF_3$	9
8	$Co(OAc)_2$ (20)	Ag_2CO_3	K_2HPO_4	4A MS	DCE	6
9	$Co(OAc)_2$ (20)	$PhI(OAc)_2$	K_2HPO_4	4A MS	$PhCF_3$	0
10	$Co(OAc)_2$ (20)	$K_2S_2O_8$	K_2HPO_4	4A MS	$PhCF_3$	0
11	$Co(OAc)_2$ (20)	AgOAc	K_2HPO_4	4A MS	$PhCF_3$	0
12	$Co(OAc)_2$ (20)	Ag_2O	K_2HPO_4	4A MS	$PhCF_3$	<5
13	$Co(OAc)_2$ (20)	Ag_2CO_3	Na_2HPO_4	4A MS	$PhCF_3$	8
14	$Co(OAc)_2$ (20)	Ag_2CO_3	Li_2CO_3	4A MS	$PhCF_3$	<5
15	$Co(OAc)_2$ (20)	Ag_2CO_3	KOAc	4A MS	$PhCF_3$	5
16	$Co(OAc)_2$ (20)	Ag_2CO_3	$PhCO_2K$	4A MS	$PhCF_3$	<5
17	$CoCl_2$ (20)	Ag_2CO_3	K_2HPO_4	4A MS	$PhCF_3$	<5
18	$CoBr_2$ (20)	Ag_2CO_3	K_2HPO_4	4A MS	$PhCF_3$	<5
19	CoF_2 (20)	Ag_2CO_3	K_2HPO_4	4A MS	$PhCF_3$	<5
20	$Co(acac)_2$ (20)	Ag_2CO_3	K_2HPO_4	4A MS	$PhCF_3$	17
21	CoF_3 (20)	Ag_2CO_3	K_2HPO_4	4A MS	$PhCF_3$	7
22	$Co(acac)_3$ (20)	Ag_2CO_3	K_2HPO_4	4A MS	$PhCF_3$	38
23	$Co(acac)_3$ (20)	Ag_2CO_3	K_2HPO_4	5A MS	$PhCF_3$	32
24	$Co(acac)_3$ (20)	Ag_2CO_3	K_2HPO_4	3A MS	$PhCF_3$	51
25	$Co(acac)_3$ (20)	Ag_2CO_3	K_2HPO_4	3A MS, $ZnCl_2$	$PhCF_3$	<5
26	$Co(acac)_3$ (20)	Ag_2CO_3	K_2HPO_4	3A MS, $MgCl_2$	$PhCF_3$	15
27	$Co(acac)_3$ (20)	Ag_2CO_3	K_2HPO_4	3A MS, MgO	$PhCF_3$	57
28	$Co(acac)_3$ (20)	Ag_2CO_3	K_2HPO_4	3A MS, $B(OMe)_3$	$PhCF_3$	20
29	$Co(acac)_3$ (20)	Ag_2CO_3	K_2HPO_4	3A MS, $B(OH)_3$	$PhCF_3$	67(64)

Supplementary Table 3. Control Experiments of Intermolecular Amination

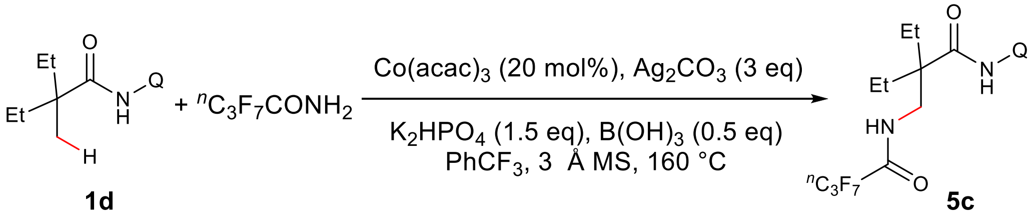

1d **5c**

Entry	Change from the "standard conditions"[a]	Yield of **5c** (%)[b]
1	none	55
2	no $Co(OAc)_2$	0
3	Shi's conditions[c] instead of the standard conditions	0
4	Co(acac)3 (1eq), no Ag2CO3	0
5	addition of TEMPO (1 eq)	48
6	addition of TEMPO (2 eq)	41

[a]Reaction conditions: **1d** (0.15 mmol), heptafluorobutanamide (0.45 mmol), $Co(acac)_3$ (0.03 mmol), Ag_2CO_3 (0.45 mmol), K_2HPO_4 (0.23 mmol), $B(OH)_3$ (0.075 mmol), 2.0 mL $PhCF_3$, 160 °C, 24 h.

[b]Yields are based on **1d**, determined by ^{1}H-NMR using dibromomethane as the internal standard.

[c]Shi’s conditions: see Table 2.

Supplementary Methods

General Information

^{1}H, ^{13}C, ^{19}F NMR and NOESY spectra were recorded on a Bruker 500 MHz NMR Fourier transform spectrometer (500 MHz and 125 MHz, respectively) using tetramethylsilane as an internal reference, and chemical shifts (δ) and coupling constants (J) were expressed in ppm and Hz, respectively. Infrared spectra were obtained using a Thermo Nicolet IR 330 spectrometer. Mass (MS) analysis were abtained using Agilent 1100 series LC/MSD system with Electrospray Ionization (ESI). All the solvents and commercially available reagents were purchased from commercial sources and used directly. Starting materials **1a-k**, **1m**, **1s-1ab**, **1an**, **1as** and [D_3]-**1d** were prepared according to literature procedures.[1,2,3] **1q** and **1ah** were prepared based on reported reaction protocol.[4] The relative configuration of **3g-k**, **4b1**, **6e** and **7** was assigned by NOESY analysis.

General Procedure A for the Preparation of Starting Materials (1l, 1r1, 1r2, 1ac-ag, 1ai-am and 1ao-ar)

A solution of LDA (10 mmol) in THF was prepared from diisopropylamine (1.5 mL, 10.7 mmol) and 2.5 M *n*-BuLi in hexane (4.0 mL, 10 mmol) at -78 ^oC. To this LDA solution, carboxylate ester (10 mmol) was added dropwise at -78 ^oC and the mixture was stirred at this temperature for 1 h. Alkyl halide (15 mmol) was then added dropwise to the solution at -78 ^oC. After the addition, the mixture was warmed to room temperature and stirred overnight. Then the mixture was quenched with water at 0 ^oC, extracted with Et_2O (15 mL x 3). The combined organic layers were washed with brine, dried over $MgSO_4$, and then evaporated in vacuo to give the crude ester.[1]

To the ester was added a solution of NaOH (2 M, 8.0 mL) and methanol (10 mL). The mixture was stirred overnight at 60 ^oC. After removal of methanol in vacuo, the pH of the mixture was adjusted to 2 with 3.0 M HCl. The mixture was then saturated with NaCl and extracted with Et_2O (15 mL x 3). The combined organic layers were washed with brine, dried over $MgSO_4$, and then evaporated in vacuo to give the crude carboxylic acid, which was used directly for the next step without further purification.[1]

Oxalyl chloride (1.75 mL, 20 mmol) was added slowly to a stirred solution of the carboxylic acid in CH_2Cl_2 (20 mL) and DMF (0.1 mL) at 0 ^oC. The mixture was stirred for 1 h at 0 ^oC and another 16 h at room temperature, and evaporated in vacuo. The residue was then dissolved in toluene (5 mL), evaporated in vacuo twice, to give the crude acid chloride, which was used directly for the next step without further purification.[5]

The acid chloride was added dropwise to a solution of 8-aminoquinoline (1.01 g, 7.0 mmol) and Et_3N (1.7 mL, 12 mmol) in CH_2Cl_2 (12 mL). The mixture was stirred overnight at room temperature. Then the mixture was diluted with CH_2Cl_2 (10 mL), washed successively with water, saturated aqueous $NaHCO_3$, and brine. The organic layer was dried over $MgSO_4$ and concentrated under reduced pressure. The residue was purified by flash column chromatography on silica gel, eluting with EtOAc/hexane (1:100, v/v), to afford corresponding 8-aminoquinolinyl amides **1**.[1]

General Procedure B for the Preparation of Starting Materials (1n-p)

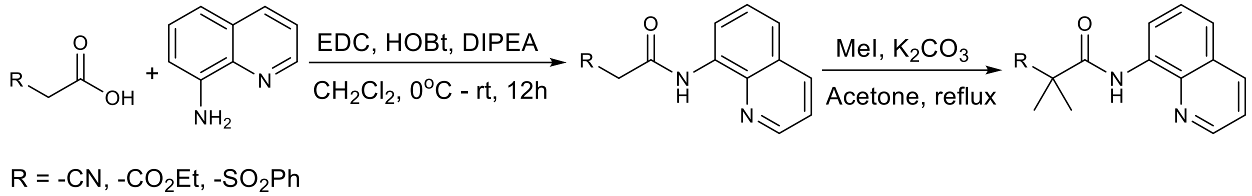

A mixture of 2-cyanoacetic acid (0.43 g, 5.0 mmol), 8-aminoquinoline (0.72 g, 5.0 mmol), HOBt·H_2O (0.84 g, 5.5 mmol) and DIPEA (0.71 g, 5.5 mmol) in anhydrous CH_2Cl_2 (20 mL) was stirred at 0 °C for 15 min. After EDC (1.05 g, 5.5 mmol) was added, the mixture was stirred for another 15 min at 0 °C and 12 h at room temperature. Then water was added and the mixture was extracted with CH_2Cl_2 (50 mL). The organic layer was washed with water and brine, dried over Na_2SO_4, and concentrated under reduced pressure. The residue was purified by flash column chromatography on silica gel, eluting with EtOAc/hexane (1:20, v/v), to afford 2-cyano-N-(quinolin-8-yl)acetamide.

K_2CO_3 (1.66 g, 12 mmol) was added dropwise to a solution of 2-cyano-N-(quinolin-8-yl)acetamide (0.74g, 0.35 mmol) in acetone (10 mL). The mixture was set to reflux. Once the reaction had reached refluxe temperature, iodomethane (1.42 g, 10 mmol) was added, and the reaction was allowed to reflux overnight. Then the solvent was reduced to 1/5 of the original volume by evaporation, poured into a beaker containing 20 mL of ice-water, extracted with CH_2Cl_2 (5×5 mL). The combined organic layers were dried over Na_2SO_4, and concentrated under reduced pressure. The residue was purified by flash column chromatography on silica gel, eluting with EtOAc/hexane (1:20, v/v), to afford 2-cyano-2-methyl-N-(quinolin-8-yl)propanamide (**1n**) in 65% yield over two steps.

1m and **1p** were prepared by following the same procedure.

Procedure for the Preparation of Starting Materials (1r2)

Methyl 2-hydroxyisobutyrate (0.59 g, 5.0 mmol) was dissolved under N_2 in THF (10 mL) and cooled to 0oC. NaH (60 wt%, 0.32 g, 8.0 mmol) was added portionwise followed by iodomethane (1.42 g, 10 mmol), then the mixture was stirred at room temperature overnight. The mixture was quenched with saturated aq. NH_4Cl and extracted with EtOAc. The combined organic layers were dried over Na_2SO_4, and

concentrated under reduced pressure to give the crude methyl 2-methoxyisobutyrate, which was used directly for the next step without further purification. 2-methoxy-2-methyl-N-(quinolin-8-yl)propanamide **1r2** was prepared from methyl 2-methoxyisobutyrate by following the general procedure A.

Analytical Data of Starting Materials

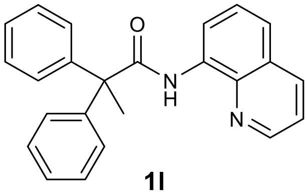

1l

Compound **1l**,[6] white solid, was prepared from methyl 3,3-dimethylbutanoate and iodomethane. ^{1}H NMR (500 MHz, $CDCl_3$) *δ* 2.17 (s, 3H), 7.28-7.42 (m, 11H), 7.44-7.47 (m, 1H), 7.50-7.54 (m, 1H), 8.06 (d, *J* = 8.2 Hz, 1H), 8.49 (d, *J* = 4.0 Hz, 1H), 8.86 (d, *J* = 7.6 Hz, 1H), 10.12 (brs, 1H).

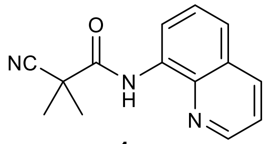

1n

Compound **1n**, white solid, was prepared from 2-cyanoacetic acid and iodomethane. ^{1}H NMR (500 MHz, $CDCl_3$) δ 1.78 (s, 6H), 7.47-7.60 (m, 3H), 8.18 (dd, *J* = 8.3, 1.4 Hz, 1H), 8.70 (dd, *J* = 7.2, 1.5 Hz, 1H), 8.88 (dd, *J* = 4.2, 1.5 Hz, 1H), 10.83 (brs, 1H); ^{13}C NMR (125 MHz, $CDCl_3$) *δ* 25.5, 40.2, 116.9, 122.1, 122.2, 122.8, 127.3, 128.0, 133.8, 136.4, 138.9, 149.0, 167.0; IR (neat) *v* 3312, 3050, 2986, 2940, 2235, 1694, 1533, 1487, 1425, 1327, 1159, 918, 826, 791, 756, 668; MS (ESI) (*m*/*z*): $[M+H]^+$ calcd for $C_{14}H_{14}N_3O$ 240.1, found 240.1.

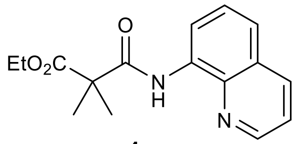

1o

Compound **1o**, colorless oil, was prepared from mono-ethyl malonate and iodomethane. ^{1}H NMR (500 MHz, $CDCl_3$) δ 1.32 (t, J = 7.1 Hz, 3H), 1.67 (s, 6H), 4.29 (q, J = 7.1 Hz, 2H), 7.41-7.53 (m, 3H), 8.12 (dd, J = 8.3, 1.6 Hz, 1H), 8.75-8.83 (m, 2H), 10.63 (brs, 1H); ^{13}C NMR (125 MHz, $CDCl_3$) δ 14.0, 23.6, 51.6, 61.8, 116.5, 121.6, 121.8, 127.3, 127.9, 134.5, 136.3, 138.8, 148.4, 170.4, 173.9; IR (neat) ν 3325, 3049, 2983, 2939, 1733, 1683, 1538, 1487, 1386, 1269, 1141, 1024, 916, 826, 792, 757; MS (ESI) (m/z): $[M+H]^+$ calcd for $C_{16}H_{19}N_2O_3$ 287.1, found 287.1.

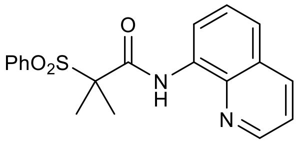

1p

Compound **1p**, white solid, was prepared from 2-(phenylsulfonyl)acetic acid and iodomethane. ^{1}H NMR (500 MHz, $CDCl_3$) δ 1.81 (s, 6H), 7.42-7.62 (m, 6H), 7.86 (d, J = 7.9 Hz, 2H), 8.16-8.20 (m, 1H), 8.61-8.64 (m, 1H), 8.91-8.94 (m, 1H), 11.17 (brs, 1H); ^{13}C NMR (125 MHz, $CDCl_3$) δ 20.7, 69.6, 116.9, 122.0, 122.5, 127.2, 128.1, 129.0, 130.3, 134.3 (2C), 135.3, 136.3, 139.1, 149.0, 165.9; IR (neat) ν 3338, 3065, 2990, 2942, 1684, 1539, 1488, 1305, 1156, 1077, 912, 827, 792, 730, 690; MS (ESI) (m/z): $[M+H]^+$ calcd for $C_{19}H_{19}N_2O_3S$ 355.1, found 355.0.

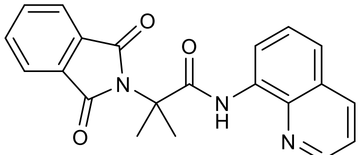

1q

Compound **1q**, white solid, was prepared from 2-aminoisobutyric acid based on reported reaction protocol. ^{1}H NMR (500 MHz, $CDCl_3$) δ 2.08 (s, 6H), 7.38-7.42 (m, 1H), 7.49-7.56 (m, 2H), 7.69-7.73 (m, 2H), 7.79-7.83 (m, 2H), 8.12-8.15 (m, 1H), 8.64-8.66 (m, 1H), 8.74-8.77 (m, 1H), 10.33 (brs, 1H); ^{13}C NMR (125 MHz, $CDCl_3$) δ 25.1, 62.7, 116.7, 121.7, 121.8, 123.3, 127.8, 128.1, 132.1, 134.2, 134.4, 136.4, 138.9, 148.4, 168.9, 171.3; IR (neat) ν 3356, 3049, 2987, 2939, 1778, 1713, 1531, 1487, 1371, 1325, 1145, 1070, 917, 826, 791, 720; MS (ESI) (m/z): $[M+H]^+$ calcd for $C_{21}H_{18}N_3O_3$ 360.1, found 360.1.

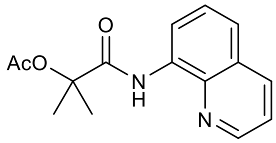

1r1

Compound **1r1**, white solid, was prepared from 2- acetoxyisobutyric acid which was prepared from 2-hydroxyisobutyric acid according to the reported literature procedure.[7] ^{1}H NMR (500 MHz, $CDCl_3$) δ 1.81 (s, 6H), 2.22 (s, 3H), 7.44-7.56 (m, 3H), 8.16 (dd, J = 8.3, 1.6 Hz, 1H), 8.73-8.82 (m, 2H), 10.53 (brs, 1H); ^{13}C NMR (125 MHz, $CDCl_3$) δ 22.0, 24.7, 81.9, 116.6, 121.8, 121.9, 127.6, 128.1, 134.3, 136.5, 139.0, 148.5, 169.7, 171.6; IR (neat) v 3348, 2939, 1745, 1695, 1532, 1487, 1368, 1240, 1142, 1018, 969,826, 792, 758; MS (ESI) (m/z): $[M+H]^+$ calcd for $C_{15}H_{17}N_2O_3$ 273.1, found 273.1.

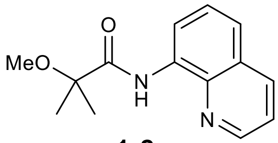

1r2

Compound **1r2**, white solid, was prepared from methyl 2-methoxyisobutyrate which was prepared from methyl 2-hydroxyisobutyrate. ^{1}H NMR (500 MHz, $CDCl_3$) δ 1.55 (s, 6H), 3.46 (s, 3H), 7.43-7.56 (m, 3H), 8.15 (dd, J = 8.3, 1.6 Hz, 1H), 8.78-8.81 (m, 1H), 8.84-8.87 (m, 1H), 10.95 (brs, 1H); ^{13}C NMR (125 MHz, $CDCl_3$) δ 23.7, 51.8, 79.6, 116.5, 121.7, 121.8, 127.4, 128.2, 134.6, 136.3, 139.2, 148.7, 174.4; IR (neat) v 3341, 3047, 2980, 2936, 2832, 1685, 1521, 1485, 1385, 1154, 1068, 917, 826, 791, 757; MS (ESI) (m/z): $[M+H]^+$ calcd for $C_{14}H_{17}N_2O_2$ 245.1, found 245.1.

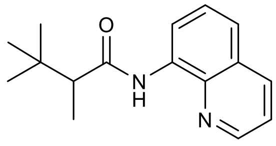

1ac

Compound **1ac**, colorless oil, was prepared from methyl 3,3-dimethylbutanoate and iodomethane. ^{1}H NMR (500 MHz, $CDCl_3$) δ 1.09 (s, 9H), 1.30 (d, J = 7.0 Hz, 3H), 2.40 (q, J = 7.0 Hz, 1H), 7.42-7.55 (m, 3H), 8.15 (dd, J = 8.3, 1.6 Hz, 1H), 8.80-8.83 (m, 2H), 9.79 (brs, 1H); ^{13}C NMR (125 MHz, $CDCl_3$) δ 13.3, 27.9, 33.4, 53.0, 116.6, 121.4, 121.7, 127.6, 128.1, 134.7, 136.5, 138.7, 148.3, 174.6; IR (neat) v 3360, 2969,

1682, 1524, 1485, 1424, 1386, 1323, 1156, 826, 791, 668; MS (ESI) (*m*/*z*): $[M+H]^+$ calcd for $C_{16}H_{21}N_2O$ 257.2, found 257.2.

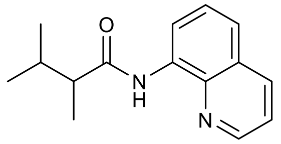

1ad

Compound **1ad**, colorless oil, was prepared from methyl 3-methylbutanoate and iodomethane. ^1H NMR (500 MHz, $CDCl_3$) δ 1.03 (t, J = 6.2 Hz, 6H), 1.31 (d, J = 6.9 Hz, 3H), 2.00-2.10 (m, 1H), 2.30-2.37 (m, 1H), 7.42-7.55 (m, 3H), 8.15 (dd, J = 8.3, 1.5 Hz, 1H), 8.80-8.83 (m, 2H), 9.84 (brs, 1H); ^{13}C NMR (125 MHz, $CDCl_3$) δ 15.1, 19.7, 21.3, 31.9, 50.1, 116.6, 121.4, 121.7, 127.6, 128.1, 134.8, 136.5, 138.7, 148.3, 175.3; IR (neat) ν 3357, 2963, 1683, 1525, 1485, 1324, 826, 791, 756, 675; MS (ESI) (*m*/*z*): $[M+H]^+$ calcd for $C_{15}H_{19}N_2O$ 243.2, found 243.2.

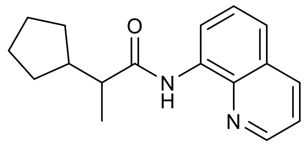

1ae

Compound **1ae**, colorless oil, was prepared from methyl 2-cyclopentylacetate and iodomethane. ^1H NMR (500 MHz, $CDCl_3$) δ 1.21-1.32 (m, 2H), 1.34 (d, J = 6.8 Hz, 3H), 1.53-1.69 (m, 4H), 1.81-1.88 (m, 1H), 1.89-1.96 (m, 1H), 2.11-2.20 (m, 1H), 2.34-2.41 (m, 1H), 7.43-7.55 (m, 3H), 8.16 (dd, J = 8.2, 1.5 Hz, 1H), 8.80-8.83 (m, 2H), 9.83 (brs, 1H); ^{13}C NMR (125 MHz, $CDCl_3$) δ 17.2, 25.2, 25.4, 30.8, 31.5, 44.3, 49.1, 116.6, 121.4, 121.7, 127.6, 128.1, 134.8, 136.5, 138.7, 148.3, 175.6; IR (neat) ν 3356, 2952, 1685, 1523, 1485, 1323, 1155, 825, 791, 756, 669; MS (ESI) (*m*/*z*): $[M+H]^+$ calcd for $C_{17}H_{21}N_2O$ 269.2, found 269.2.

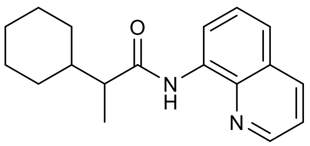

1af

Compound **1af**,[6] pale yellow oil, was prepared from methyl 2-cyclohexylacetate and iodomethane. ^{1}H NMR (500 MHz, $CDCl_3$) δ 0.98-1.07 (m, 1H), 1.08-1.19 (m, 2H), 1.23-1.28 (m, 2H), 1.30 (d, J = 6.9 Hz, 3H), 1.62-1.83 (m, 5H), 1.88-1.93 (m, 1H), 2.32-2.38 (m, 1H), 7.42-7.54 (m, 3H), 8.14 (dd, J = 8.3, 1.6 Hz, 1H), 8.79-8.84 (m, 2H), 9.82 (brs, 1H).

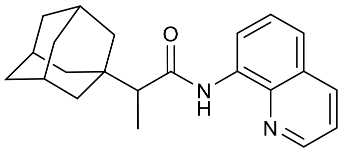

1ag

Compound **1ag**, white solid, was prepared from methyl 2-(adamantan-1-yl)acetate and iodomethane. ^{1}H NMR (500 MHz, $CDCl_3$) δ 1.26 (d, J = 7.0 Hz, 3H), 1.62-1.72 (m, 9H), 1.81-1.86 (m, 3H), 1.97-2.00 (m, 3H), 2.23 (q, J = 7.0 Hz, 1H), 7.43-7.55 (m, 3H), 8.15 (dd, J = 8.3, 1.6 Hz, 1H), 8.80-8.85 (m, 2H), 9.77 (brs, 1H); ^{13}C NMR (125 MHz, $CDCl_3$) δ 11.5, 28.8, 35.1, 37.2, 40.1, 54.2, 116.5, 121.3, 121.7, 127.6, 128.1, 134.7, 136.5, 138.7, 148.3, 174.2; IR (neat) v 3359, 2903, 2847, 1679, 1523, 1484, 1322, 1160, 924, 825, 791, 732; MS (ESI) (m/z): $[M+H]^+$ calcd for $C_{22}H_{27}N_2O$ 335.2, found 335.2.

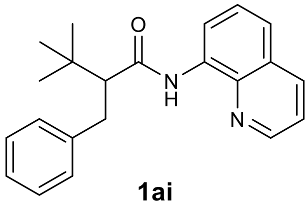

1ai

Compound **1ai**, colorless oil, was prepared from methyl 3,3-dimethylbutanoate and benzyl bromide. ^{1}H NMR (500 MHz, $CDCl_3$) δ 1.18 (s, 9H), 2.48 (dd, J = 11.5, 2.6 Hz, 1H), 2.92 (dd, J = 13.4, 2.4 Hz, 1H), 3.20 (dd, J = 13.4, 11.5 Hz, 1H), 7.03-7.07 (m, 1H), 7.14-7.18 (m, 2H), 7.24 (d, J = 7.1 Hz, 2H), 7.36-7.51 (m, 3H), 8.09 (dd, J = 8.3, 1.6 Hz, 1H), 8.69-8.77 (m, 2H), 9.50 (brs, 1H); ^{13}C NMR (125 MHz, $CDCl_3$) δ 28.3, 33.9, 34.6, 62.3, 116.6, 121.3, 121.5, 126.1, 127.5, 128.0, 128.5, 128.9, 134.5, 136.3, 138.6, 141.0, 148.1, 172.9; IR (neat) v 3360, 2960, 1682, 1524, 1485, 1322, 825, 791, 746, 699; MS (ESI) (m/z): $[M+H]^+$ calcd for $C_{22}H_{25}N_2O$ 333.2, found 333.2.

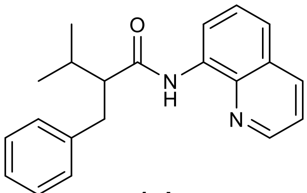

1aj

Compound **1aj**, yellow oil, was prepared from methyl 3-methylbutanoate and benzyl bromide. ^{1}H NMR (500 MHz, $CDCl_3$) δ 1.09 (d, J = 6.7 Hz, 3H), 1.14 (d, J = 6.8 Hz, 3H), 2.06-2.14 (m, 1H), 2.50-2.55 (m, 1H), 2.97 (dd, J = 13.6, 4.3 Hz, 1H), 3.11 (dd, J = 13.6, 10.4 Hz, 1H), 7.03-7.06 (m, 1H), 7.14-7.18 (m, 2H), 7.25 (d, J = 7.0 Hz, 2H), 7.32-7.35 (m, 1H), 7.40 (dd, J = 8.3, 1.2 Hz, 1H), 7.44-7.48 (m, 1H), 8.04 (dd, J = 8.3, 1.6 Hz, 1H), 8.68 (dd, J = 4.2, 1.7 Hz, 1H), 8.76 (dd, J = 7.6, 1.2 Hz, 1H), 9.55 (brs, 1H); ^{13}C NMR (125 MHz, $CDCl_3$) δ 20.6, 20.8, 31.3, 36.3, 58.4, 116.5, 121.3, 121.5, 126.1, 127.4, 127.9, 128.4, 129.0, 134.4, 136.2, 138.4, 140.4, 148.1, 173.2; IR (neat) ν 3357, 2960, 1682, 1525, 1485, 1424, 1386, 1323, 1159, 913, 825, 791, 744, 699; MS (ESI) (m/z): $[M+H]^+$ calcd for $C_{21}H_{23}N_2O$ 319.2, found 319.2.

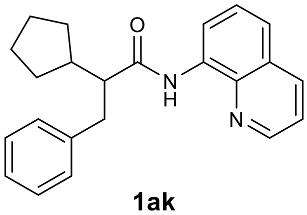

1ak

Compound **1ak**, pale yellow solid, was prepared from methyl 2-cyclopentylacetate and benzyl bromide. ^{1}H NMR (500 MHz, $CDCl_3$) δ 1.32-1.42 (m, 2H), 1.51-1.72 (m, 4H), 1.80-1.87 (m, 1H), 1.99-2.06 (m, 1H), 2.21-2.30 (m, 1H), 2.52-2.58 (m, 1H), 2.99 (dd, J = 13.7, 3.9 Hz, 1H), 3.12 (dd, J = 13.6, 10.6 Hz, 1H), 7.02-7.06 (m, 1H), 7.13-7.18 (m, 2H), 7.23 (d, J = 7.0 Hz, 2H), 7.35-7.39 (m, 1H), 7.42-7.50 (m, 2H), 8.08 (dd, J = 8.3, 1.5 Hz, 1H), 8.68-8.76 (m, 2H), 9.52 (brs, 1H); ^{13}C NMR (125 MHz, $CDCl_3$) δ 25.1, 25.2, 31.2, 31.3, 38.4, 43.6, 57.6, 116.6, 121.4, 121.5, 126.2, 127.5, 128.0, 128.5, 128.9, 134.5, 136.3, 138.5, 140.2, 148.1, 173.7; IR (neat) ν 3356, 2951, 1633, 1525, 1485, 1323, 1159, 915, 825, 791, 740, 699; MS (ESI) (m/z): $[M+H]^+$ calcd for $C_{23}H_{25}N_2O$ 345.2, found 345.2.

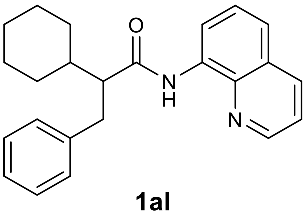

1al

Compound **1al**, pale yellow solid, was prepared from methyl 2-cyclohexylacetate and benzyl bromide. ^{1}H NMR (500 MHz, $CDCl_3$) δ 1.10-1.35 (m, 5H), 1.64-1.90 (m, 5H), 2.02-2.08 (m, 1H), 2.51-2.56 (m, 1H), 2.98-3.12 (m, 2H), 7.03-7.07 (m, 1H), 7.14-7.19 (m, 2H), 7.22-7.28 (m, 2H), 7.38-7.51 (m, 3H), 8.10 (d, J = 8.2 Hz, 1H), 8.71-8.77 (m, 2H), 9.53 (brs, 1H); ^{13}C NMR (125 MHz, $CDCl_3$) δ 26.5, 26.6 (2C), 31.1, 31.3, 36.2, 41.0, 57.9, 116.5, 121.4, 121.6, 126.1, 127.5, 128.0, 128.5, 129.0, 134.5, 136.3, 138.5, 140.5, 148.1, 173.4; IR (neat) *v* 3365, 2927, 1678, 1525, 1494, 1270, 1090, 788, 735, 700; MS (ESI) (*m*/*z*): $[M+H]^+$ calcd for $C_{24}H_{27}N_2O$ 359.2, found 359.2.

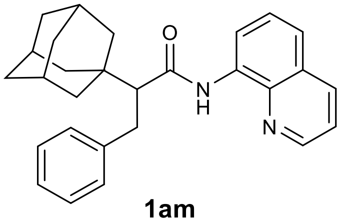

1am

Compound **1am**, white solid, was prepared from methyl 2-(adamantan-1-yl)acetate and benzyl bromide. ^{1}H NMR (500 MHz, $CDCl_3$) δ 1.66-1.72 (m, 9H), 1.96-2.04 (m, 6H), 2.30 (dd, *J* = 11.5, 2.4 Hz, 1H), 2.95 (dd, *J* = 13.4, 2.3 Hz, 1H), 3.15 (dd, *J* = 13.2, 11.7 Hz, 1H), 7.02-7.06 (m, 1H), 7.13-7.17 (m, 2H), 7.23 (d, *J* = 7.2 Hz, 2H), 7.37-7.41 (m, 1H), 7.45 (dd, *J* = 8.2, 1.3 Hz, 1H), 7.48-7.52 (m, 1H), 8.10 (dd, *J* = 8.3, 1.6 Hz, 1H), 8.72 (dd, *J* = 4.2, 1.6 Hz, 1H), 8.78 (dd, *J* =7.6, 1.2 Hz, 1H), 9.49 (brs, 1H); ^{13}C NMR (125 MHz, $CDCl_3$) δ 28.9, 32.7, 35.8, 37.1, 40.4, 63.6, 116.5, 121.3, 121.6, 126.0, 127.5, 128.0, 128.5, 129.0, 134.5, 136.3, 138.5, 141.2, 148.2, 172.4; IR (neat) *v* 3360, 2903, 2847, 1681, 1523, 1484, 1322, 909, 825, 790, 733, 699; MS (ESI) (*m*/*z*): $[M+H]^+$ calcd for $C_{28}H_{31}N_2O$ 411.2, found 411.2.

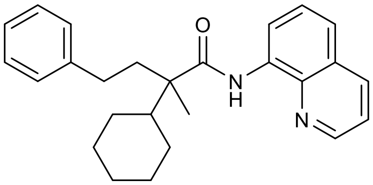

1ao

Compound **1ao**, white solid, was prepared from methyl 2-cyclohexylacetate with iodomethane and (2-bromoethyl)benzene. ^{1}H NMR (500 MHz, $CDCl_3$) δ 1.06-1.30 (m, 5H), 1.41 (s, 3H), 1.62-1.85 (m, 6H), 1.86-1.93 (m, 1H), 2.12-2.19 (m, 1H), 2.56-2.69 (m, 2H), 7.12-7.26 (m, 5H), 7.44-7.57 (m, 3H), 8.16 (dd, J = 8.2, 1.3 Hz, 1H), 8.82-8.87 (m, 2H), 10.31 (brs, 1H); ^{13}C NMR (125 MHz, $CDCl_3$) δ 16.9, 26.8, 27.0, 27.1, 27.4, 28.7, 31.5, 40.9, 47.1, 51.1, 116.5, 121.4, 121.7, 125.8, 127.6, 128.1, 128.4, 128.6, 134.7, 136.4, 139.0, 142.8, 148.4, 175.9; IR (neat) ν 3363, 2928, 2852, 1674, 1526, 1486, 1383, 1325, 909, 825, 791, 732, 699; MS (ESI) (*m*/*z*): $[M+H]^+$ calcd for $C_{26}H_{31}N_2O$ 387.2, found 387.2.

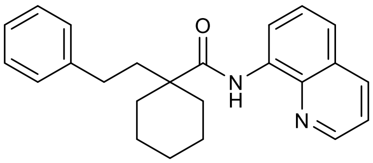

1ap

Compound **1ap**, yellow oil, was prepared from methyl cyclohexanecarboxylate and (2-bromoethyl)benzene. ^{1}H NMR (500 MHz, $CDCl_3$) δ 1.36-1.45 (m, 1H), 1.56-1.74 (m, 7H), 1.97-2.01 (m, 2H), 2.29-2.34 (m, 2H), 2.62-2.67 (m, 2H), 7.11-7.24 (m, 5H), 7.44-7.58 (m, 3H), 8.17 (dd, J = 8.3, 1.5 Hz, 1H), 8.82-8.87 (m, 2H), 10.36 (brs, 1H); ^{13}C NMR (125 MHz, $CDCl_3$) δ 23.1, 26.3, 30.8, 34.6, 42.9, 48.2, 116.5, 121.3, 121.7, 125.8, 127.6, 128.1, 128.4, 128.5, 134.8, 136.4, 139.0, 142.5, 148.4, 175.3; IR (neat) ν 3362, 2929, 2857, 1678, 1526, 1486, 1325, 912, 825, 791, 742, 698; MS (ESI) (*m*/*z*): $[M+H]^+$ calcd for $C_{24}H_{27}N_2O$ 359.2, found 359.2.

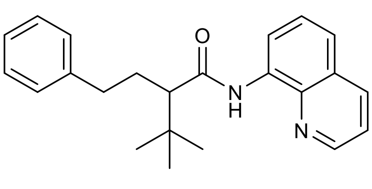

1aq

Compound **1aq**, white solid, was prepared from methyl 3,3-dimethylbutanoate and (2-bromoethyl)benzene. ^{1}H NMR (500 MHz, $CDCl_3$) δ 1.06 (s, 9H), 1.85-1.92 (m, 1H), 2.18-2.28 (m, 2H), 2.48-2.56 (m, 1H), 2.76-2.83 (m, 1H), 7.14-7.28 (m, 5H), 7.44-7.58 (m, 3H), 8.15-8.19 (m, 1H), 8.81-8.88 (m, 2H), 9.81 (brs, 1H); ^{13}C NMR (125 MHz, $CDCl_3$) δ 28.2, 30.0, 33.6, 34.7, 59.0, 116.7, 121.5, 121.7, 126.0, 127.6, 128.1, 128.5, 128.7, 134.6, 136.5, 138.7, 142.1, 148.3, 173.7; IR (neat) ν 3353, 2966, 2930, 1686, 1524, 1485, 1424, 1323, 826, 791, 752, 699, 607; MS (ESI) (*m*/*z*): $[M+H]^+$ calcd for $C_{23}H_{27}N_2O$ 347.2, found 347.2.

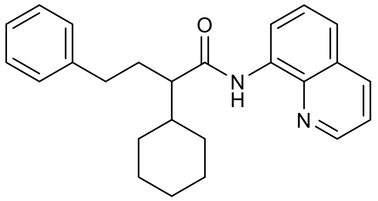

1ar

Compound **1ar**, colorless oil, was prepared from methyl 2-cyclohexylacetate and (2-bromoethyl)benzene. ^{1}H NMR (500 MHz, $CDCl_3$) δ 0.96-1.05 (m, 1H), 1.07-1.16 (m, 2H), 1.18-1.30 (m, 2H), 1.60-1.82 (m, 5H), 1.90-2.01 (m, 2H), 2.09-2.18 (m, 1H), 2.22-2.27 (m, 1H), 2.53-2.60 (m, 1H), 2.75-2.82 (m, 1H), 7.15-7.27 (m, 5H), 7.44-7.58 (m, 3H), 8.15-8.18 (m, 1H), 8.81-8.89 (m, 2H), 9.83 (brs, 1H); ^{13}C NMR (125 MHz, $CDCl_3$) δ 26.4, 26.5 (2C), 31.0, 31.4, 31.8, 34.1, 40.9, 55.1, 116.6, 121.5, 121.7, 125.9, 127.6, 128.1, 128.5, 128.7, 134.5, 136.5, 138.6, 142.2, 148.3, 174.2; IR (neat) ν 3355, 2925, 2851, 1682, 1519, 1485, 1424, 1385, 1323, 826, 791, 737, 699; MS (ESI) (*m*/*z*): $[M+H]^+$ calcd for $C_{25}H_{29}N_2O$ 373.2, found 373.2.

General Procedure for Cobalt-Catalyzed Intramolecular Amidation of Substituted *N*-(quinolin-8-yl)acetamides

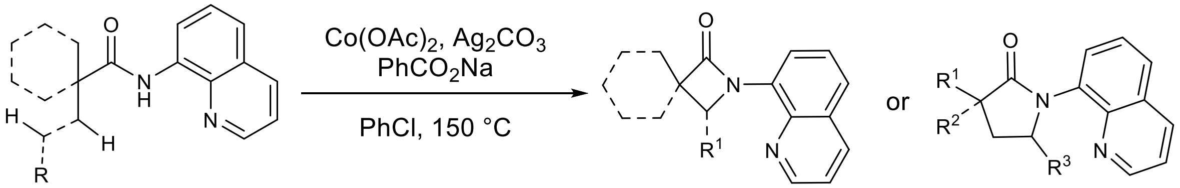

A 20 mL tube was charged with α,α,α-trisubstituted *N*-(quinolin-8-yl)acetamides (**1**, 0.30 mmol), $Co(OAc)_2$ (5.3 mg, 0.030 mmol), Ag_2CO_3 (207 mg, 0.75 mmol), $PhCO_2Na$ (21.6 mg, 0.15 mmol) and 0.60 mL of PhCl. The reaction mixture was stirred rigorously open to the air at 150 ˚C for 24 h. Then the mixture was cooled to

room temperature, diluted with EtOAc (2 mL), filtered through a celite pad, and concentrated in vacuo. The residue was purified by flash chromatography on silica gel (gradient eluent of 2% EtOAc in hexanes, v/v) to give the desired product.

General Procedure for Cobalt-Catalyzed Intermolecular Amination of α,α–Disubstituted Propanamides

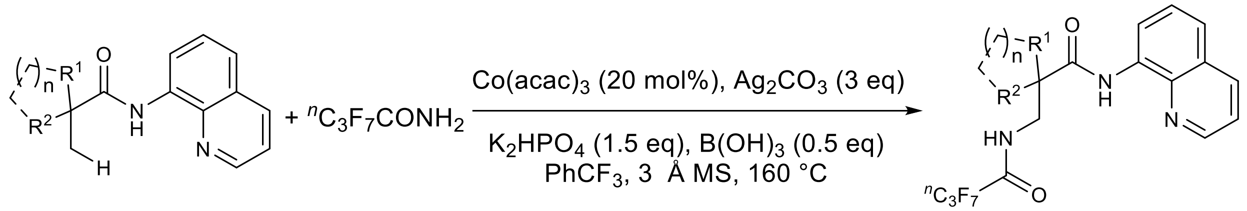

A 20 mL tube was charged with α,α,α-trisubstituted *N*-(quinolin-8-yl)acetamides (**1**, 0.15 mmol), Heptafluorobutyramide (95.9 mg, 0.45 mmol), $Co(acac)_3$ (10.7 mg, 0.030 mmol), Ag_2CO_3 (75.1 mg, 0.45 mmol), K_2HPO_4 (39.2 mg, 0.23 mmol), $B(OH)_3$ (4.6 mg, 0.075 mmol), 3Å MS (200 mg) and 2.0 mL of $PhCF_3$. Then the vial was sealed, and stirred rigorously at 160 ˚C for 24 h. The mixture was cooled to room temperature, diluted with CH_2Cl_2 (5 mL), filtered through a celite pad, and concentrated in vacuo. The residue was purified by flash chromatography on silica gel (gradient eluent of 2~5% EtOAc in hexanes, v/v) to give the desired product **5**.

Analytical Data of Products

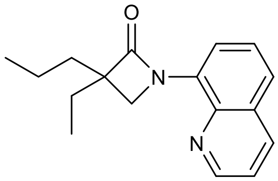

2a

Compound **2a**,[2] colorless oil, yield: 87%. ^{1}H NMR (500 MHz, $CDCl_3$) δ 0.97 (t, J = 7.3 Hz, 3H), 1.06 (t, J = 7.5 Hz, 3H), 1.40-1.49 (m, 1H), 1.53-1.62 (m, 1H), 1.72-1.85 (m, 4H), 4.29-4.34 (m, 2H), 7.34-7.37 (m, 1H), 7.45-7.49 (m, 2H), 8.08 (dd, J = 8.3, 1.7 Hz, 1H), 8.47-8.52 (m, 1H), 8.80 (dd, J = 4.1, 1.8 Hz, 1H).

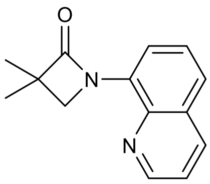

2b

Compound **2b**,[2] colorless oil, yield: 83%. ^{1}H NMR (500 MHz, $CDCl_3$) δ 1.45 (s, 6H), 4.33 (s, 2H), 7.34-7.38 (m, 1H), 7.46-7.50 (m, 2H), 8.09 (dd, *J* = 8.3, 1.6 Hz, 1H), 8.47-8.51 (m, 1H), 8.79 (dd, *J* = 4.1, 1.7 Hz, 1H).

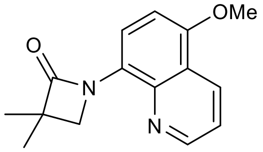

2c

Compound **2c**,[2] yellow solid, yield: 86%. ^{1}H NMR (500 MHz, $CDCl_3$) δ 1.44 (s, 6H), 3.97 (s, 3H), 4.22 (s, 2H), 6.81 (d, J = 8.5 Hz, 1H), 7.34-7.37 (m, 1H), 8.83 (dd, J = 8.5, 1.8 Hz, 1H), 8.52 (d, J = 8.5 Hz, 1H), 8.79-8.81 (m, 1H).

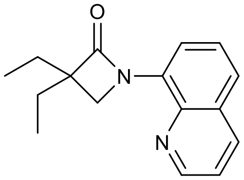

2d

Compound **2d**,[2] colorless oil, yield: 85%. ^{1}H NMR (500 MHz, $CDCl_3$) δ 1.07 (t, *J* = 7.5 Hz, 6H), 1.79-1.86 (m, 4H), 4.31 (s, 2H), 7.35-7.38 (m, 1H), 7.47-7.50 (m, 2H), 8.09 (dd, *J* = 8.3, 1.8 Hz, 1H), 8.48-8.51 (m, 1H), 8.81 (dd, *J* = 4.1, 1.8 Hz, 1H).

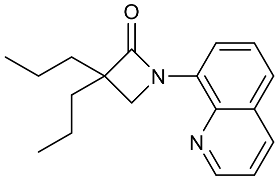

2e

Compound **2e**,[2] pale yellow oil, yield: 88%. ^{1}H NMR (500 MHz, $CDCl_3$) δ 0.96 (t, *J* = 7.3 Hz, 6H), 1.40-1.49 (m, 2H), 1.53-1.62 (m, 2H), 1.71-1.76 (m, 4H), 4.32 (s,

2H), 7.34-7.37 (m, 1H), 7.46-748 (m, 2H), 8.07 (dd, J = 8.3, 1.8 Hz, 1H), 8.48-8.51 (m, 1H), 8.80 (dd, J = 4.1, 1.8 Hz, 1H).

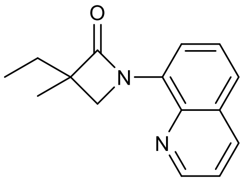

2f

Compound **2f**,[2] colorless oil, yield: 80%. ^{1}H NMR (500 MHz, $CDCl_3$) δ 1.06 (t, J = 7.5 Hz, 3H), 1.43 (s, 3H), 1.73-1.85 (m, 2H), 4.24 (d, J = 7.3 Hz, 1H), 4.39 (d, J = 7.3 Hz, 1H), 7.34-7.37 (m, 1H), 7.46-7.49 (m, 2H), 8.08 (dd, J = 8.3, 1.6 Hz, 1H), 8.48-8.51 (m, 1H), 8.79 (dd, J = 4.0, 1.6 Hz, 1H).

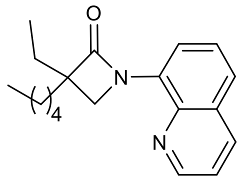

2g

Compound **2g**,[3] pale yellow oil, yield: 85%. ^{1}H NMR (500 MHz, $CDCl_3$) δ 0.89 (t, J = 7.0 Hz, 3H), 1.06 (t, J = 7.5 Hz, 3H), 1.30-1.35 (m, 4H), 1.39-1.46 (m, 1H), 1.50-1.58 (m, 1H), 1.73-1.86 (m, 4H), 4.32 (dd, J = 8.5, 7.5 Hz, 2H), 7.36-7.39 (m, 1H), 7.47-7.50 (m, 2H), 8.10 (dd, J = 8.3, 1.7 Hz, 1H), 8.48-8.51 (m, 1H), 8.81 (dd, J = 4.1, 1.8 Hz, 1H).

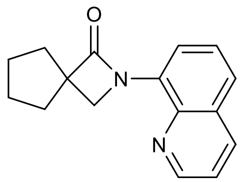

2h

Compound **2h**,[2] pale yellow oil, yield: 69%. ^{1}H NMR (500 MHz, $CDCl_3$) δ 1.70-1.77 (m, 2H), 1.81-1.88 (m, 2H), 1.96-2.02 (m, 2H), 2.16-2.22 (m, 2H), 4.45 (s, 2H), 7.35-7.39 (m, 1H), 7.48-7.51 (m, 2H), 8.10 (dd, J = 8.3, 1.8 Hz, 1H), 8.49-8.52 (m, 1H), 8.79 (dd, J = 4.1, 1.8 Hz, 1H).

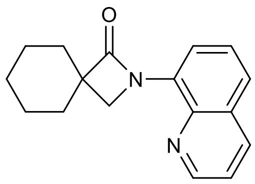

2i

Compound **2i**,[2] pale yellow oil, yield: 80%. ^{1}H NMR (500 MHz, $CDCl_3$) δ 1.38-1.51 (m, 3H), 1.58-1.64 (m, 1H), 1.82-1.92 (m, 6H), 4.33 (s, 2H), 7.34-7.38 (m, 1H), 7.46-7.50 (m, 2H), 8.09 (dd, J = 8.3, 1.7 Hz, 1H), 8.48-8.51 (m, 1H), 8.80 (dd, J = 4.1, 1.8 Hz, 1H).

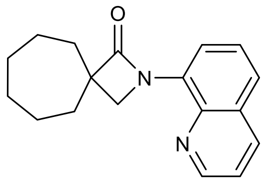

2j

Compound **2j**,[2] pale yellow oil, yield: 83%. ^{1}H NMR (500 MHz, $CDCl_3$) δ 1.58-1.66 (m, 6H), 1.80-1.86 (m, 2H), 2.02-2.06 (m, 4H), 4.35 (s, 2H), 7.35-7.38 (m, 1H), 7.47-7.49 (m, 2H), 8.08 (dd, J = 8.3, 1.8 Hz, 1H), 8.47-8.50 (m, 1H), 8.80 (dd, J = 4.1, 1.8 Hz, 1H).

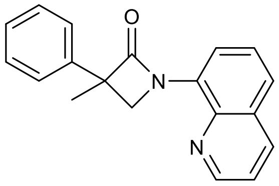

2k1

Compound **2k1**,[2] yellow oil, yield: 35%. ^{1}H NMR (500 MHz, $CDCl_3$) δ 1.81 (s, 3H), 4.66 (d, J = 7.2 Hz, 1H), 4.81 (d, J = 7.2 Hz, 1H), 7.23-7.28 (m, 1H), 7.32-7.39 (m, 3H), 7.46-7.50 (m, 2H), 7.54-7.57 (m, 2H), 8.06 (dd, J = 8.3, 1.5 Hz, 1H), 8.53-8.56 (m, 1H), 8.78 (dd, J = 4.0, 1.5 Hz, 1H).

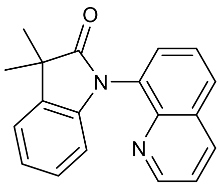

2k2

Compound **2k2**,[2] yellow oil, yield: 55%. ^{1}H NMR (500 MHz, $CDCl_3$) δ 1.57 (s, 3H), 1.65 (s, 3H), 6.36-6.38 (m, 1H), 7.06-7.09 (m, 2H), 7.30-7.33 (m, 1H), 7.40-7.44 (m, 1H), 7.65-7.69 (m, 1H), 7.78 (dd, J = 7.3, 1.4 Hz, 1H), 7.94 (dd, J = 8.2, 1.4 Hz, 1H), 8.22 (dd, J = 8.3, 1.7 Hz, 1H), 8.82 (dd, J = 4.1, 1.7 Hz, 1H).

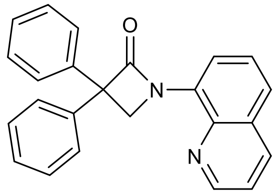

2l1

Compound **2l1**, yellow oil, yield: 18%. ^{1}H NMR (500 MHz, $CDCl_3$) δ 5.19 (s, 2H), 7.24-7.28 (m, 2H), 7.34-7.38 (m, 5H), 7.48-7.53 (m, 2H), 7.54-7.57 (m, 4H), 8.08 (dd, *J* = 8.3, 1.7 Hz, 1H), 8.58-8.61 (m, 1H), 8.80 (dd, *J* = 4.1, 1.8 Hz, 1H); ^{13}C NMR (125 MHz, $CDCl_3$) δ 61.0, 67.5, 119.9, 121.4, 123.3, 126.9, 127.3, 127.4, 128.8, 129.1, 134.8, 136.1, 140.6, 140.8, 148.7, 169.0; IR (neat) v 2960, 2930, 2871, 1728, 1505, 1473, 1402, 1347, 1155, 824, 789, 701; MS (ESI) (*m*/*z*): $[M+H]^+$ calcd for $C_{24}H_{19}N_2O$ 351.2, found 351.1.

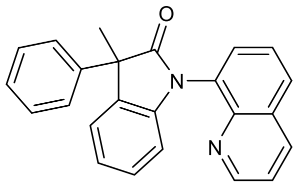

2l2

Compound **2l2**, yellow solid, yield: 70%. ^{1}H NMR (500 MHz, $CDCl_3$, a mixture of diastereomers in ratio 1:1, the minor one is marked with an *) δ 1.99 (s, 3H), 6.39-6.42 (m, 1H), 7.01-7.04 (m, 1H), 7.06-7.13 (m, 1H), 7.18-7.20 (m, 1H), 7.24-7.29 (m, 1H), 7.33-7.37 (m, 2H), 7.39-7.42 (m, 1H), 7.50 (d, *J* = 7.0 Hz, 1H), 7.61-7.65 (m, 1H), 7.72 (d, *J* = 7.0 Hz, 1H), 7.75 (dd, *J* = 7.3, 1.3 Hz, 1H), 7.90-7.94 (m, 1H), 8.20 (dd, *J* = 8.3, 1.6 Hz, 1H), 8.81-8.84 (m, 1H); 2.07 (s, 3H)*, 6.39-6.42 (m, 1H)*,

7.06-7.13 (m, 2H)*, 7.24-7.29 (m, 2H)*, 7.33-7.37 (m, 2H)*, 7.39-7.42 (m, 1H)*, 7.50 (d, *J* = 7.0 Hz, 1H)*, 7.65-7.69 (m, 1H)*, 7.72 (d, *J* = 7.0 Hz, 1H)*, 7.83 (dd, *J* = 7.3, 1.3 Hz, 1H)*, 7.90-7.94 (m, 1H)*, 8.20 (dd, *J* = 8.3, 1.6 Hz, 1H)*, 8.81-8.84 (m, 1H)*; ^{13}C NMR (125 MHz, $CDCl_3$) δ 23.3, 52.8, 110.0, 122.0, 122.9, 123.9, 126.5, 127.0, 127.2, 127.7, 128.6, 129.3, 129.7, 130.0, 132.7, 134.8, 136.2, 141.4, 144.5, 144.6, 151.0, 180.0; 24.5*, 52.9*, 110.1*, 122.0*, 122.9*, 124.4*, 126.5*, 127.2*, 127.3*, 127.9*, 128.7*, 129.3*, 129.8*, 130.2*, 132.8*, 135.8*, 136.2*, 141.6*, 144.5*, 145.0*, 151.1*, 180.3*; IR (neat) ν 2958, 2928, 2871, 1721, 1610, 1501, 1485, 1461, 1192, 824, 791, 749, 630; MS (ESI) (*m*/*z*): $[M+H]^+$ calcd for $C_{24}H_{19}N_2O$ 351.2, found 351.2.

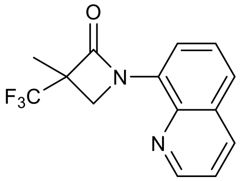

2m

Compound **2m**,[3] colorless oil, yield: 90%. ^{1}H NMR (500 MHz, $CDCl_3$) δ 1.71 (s, 3H), 4.48 (d, *J* = 8.1 Hz, 1H), 4.78 (d, *J* = 8.1 Hz, 1H), 7.38-7.42 (m, 1H), 7.48-7.52 (m, 1H), 7.55-7.58 (m, 1H), 8.12 (dd, *J* = 8.3, 1.6 Hz, 1H), 8.45 (dd, *J* = 7.6, 1.3 Hz, 1H), 8.81 (dd, *J* = 4.1, 1.7 Hz, 1H).

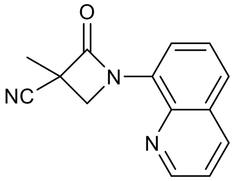

2n

Compound **2n**, yellow oil, yield: 41%. ^{1}H NMR (500 MHz, $CDCl_3$) δ 1.86 (s, 3H), 4.60 (d, *J* = 7.8 Hz, 1H), 4.94 (d, *J* = 7.8 Hz, 1H), 7.41-7.45 (m, 1H), 7.51-7.55 (m, 1H), 7.61 (dd, *J* = 8.2, 1.2 Hz, 1H), 8.15 (dd, *J* = 8.3, 1.7 Hz, 1H), 8.40 (dd, *J* = 7.6, 1.2 Hz, 1H), 8.81 (dd, *J* = 4.1, 1.7 Hz, 1H); ^{13}C NMR (125 MHz, $CDCl_3$) δ 19.3, 46.9, 57.5, 118.1, 120.2, 121.8, 124.5, 126.8, 129.0, 133.7, 136.2, 140.5, 149.2, 161.1; IR (neat) *ν* 2978, 2924, 2852, 2240, 1759, 1505, 1473, 1404, 1354, 1156, 825, 788, 760; MS (ESI) (*m*/*z*): $[M+H]^+$ calcd for $C_{14}H_{12}N_3O$ 238.1, found 238.1.

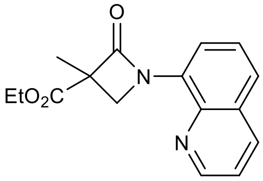

2o

Compound **2o**, yellow oil, yield: 83%. ^{1}H NMR (500 MHz, CDCl$_3$) δ 1.30 (t, J = 7.1 Hz, 3H), 1.73 (s, 3H), 4.26 (q, J = 7.1 Hz, 2H), 4.44 (d, J = 7.4 Hz, 1H), 4.87 (d, J = 7.4 Hz, 1H), 7.37-7.41 (m, 1H), 7.48-7.55 (m, 2H), 8.11 (dd, J = 8.3, 1.7 Hz, 1H), 8.47 (dd, J = 7.4, 1.4 Hz, 1H), 8.80 (dd, J = 4.1, 1.7 Hz, 1H); ^{13}C NMR (125 MHz, CDCl$_3$) δ 14.3, 17.3, 57.3, 60.5, 61.8, 119.7, 121.5, 123.5, 126.8, 129.0, 134.6, 136.1, 140.5, 148.8, 165.8, 170.3; IR (neat) v 2980, 2935, 1751, 1684, 1505, 1475, 1404, 1352, 1273, 1153, 1031, 905, 826, 789, 758; MS (ESI) (m/z): [M+H]$^+$ calcd for $C_{16}H_{17}N_2O_3$ 285.1, found 285.1.

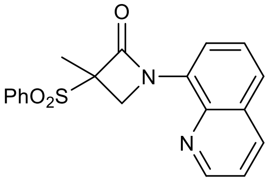

2p

Compound **2p**, pale yellow solid, yield: 73%. ^{1}H NMR (500 MHz, CDCl$_3$) δ 1.76 (s, 3H), 4.65 (d, J = 8.4 Hz, 1H), 5.17 (d, J = 8.4 Hz, 1H), 7.39-7.43 (m, 1H), 7.47-7.51 (m, 1H), 7.56-7.60 (m, 3H), 7.66-7.70 (m, 1H), 8.08 (d, J = 7.7 Hz, 2H), 8.12 (dd, J = 8.3, 1.5 Hz, 1H), 8.30-8.36 (m, 1H), 8.81 (dd, J = 4.1, 1.6 Hz, 1H); ^{13}C NMR (125 MHz, CDCl$_3$) δ 15.5, 54.2, 75.9, 120.1, 121.7, 124.3, 126.7, 129.0, 129.2, 130.2, 133.8, 134.5, 135.8, 136.1, 140.5, 149.1, 162.2; IR (neat) v 3065, 2972, 2926, 2853, 1752, 1505, 1475, 1404, 1308, 1146, 1080, 906, 826, 789, 758, 722, 690, 619; MS (ESI) (m/z): [M+H]$^+$ calcd for $C_{19}H_{17}N_2O_3S$ 353.1, found 353.1.

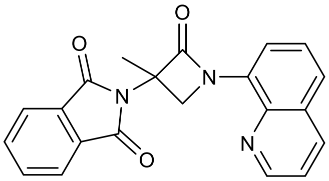

2q

Compound **2q**, white solid, yield: 81%. ^{1}H NMR (500 MHz, CDCl$_3$) δ 2.15 (s, 3H), 4.82 (s, 2H), 7.35-7.38 (m, 1H), 7.50-7.56 (m, 2H), 7.69-7.72 (m, 2H), 7.80-7.84 (m,

2H), 8.10 (dd, J = 8.3, 1.7 Hz, 1H), 8.58 (dd, J = 7.2, 1.8 Hz, 1H), 8.77 (dd, J = 4.1, 1.8 Hz, 1H); ^{13}C NMR (125 MHz, $CDCl_3$) δ 19.5, 60.2, 67.3, 120.1, 121.4, 123.4, 123.7, 126.9, 129.0, 131.9, 134.3, 134.7, 136.0, 140.5, 148.7, 166.1, 167.8; IR (neat) ν 3062, 2977, 2934, 1751, 1717, 1505, 1474, 1373, 1154, 1077, 909, 826, 790, 717; MS (ESI) (m/z): $[M+H]^+$ calcd for $C_{21}H_{16}N_3O_3$ 358.1, found 358.0.

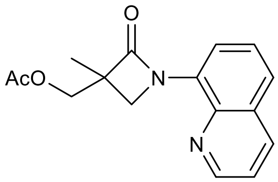

2s

Compound **2s**,[3] pale yellow oil, yield: 78%. ^{1}H NMR (500 MHz, $CDCl_3$) δ 1.50 (s, 3H), 2.06 (s, 3H), 4.33 (s, 2H), 4.36 (d, J = 7.5 Hz, 1H), 4.53 (d, J = 7.4 Hz, 1H), 7.37-7.41 (m, 1H), 7.48-7.55 (m, 2H), 8.11 (dd, J = 8.3, 1.7 Hz, 1H), 8.47 (dd, J = 7.4, 1.6 Hz, 1H), 8.80 (dd, J = 4.1, 1.7 Hz, 1H).

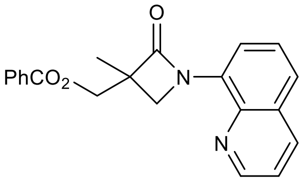

2t

Compound **2t**,[3] yellow oil, yield: 74%. ^{1}H NMR (500 MHz, $CDCl_3$) δ 1.59 (s, 3H), 4.42 (d, J = 7.4 Hz, 1H), 4.58 (dd, J = 18.5, 11.4 Hz, 2H), 4.65 (d, J = 7.4 Hz, 1H), 7.30-7.34 (m, 2H), 7.37-7.40 (m, 1H), 7.47-7.57 (m, 3H), 7.98-8.01 (m, 2H), 8.12 (dd, J = 8.3, 1.7 Hz, 1H), 8.48 (dd, J = 7.4, 1.5 Hz, 1H), 8.79 (dd, J = 4.1, 1.7 Hz, 1H).

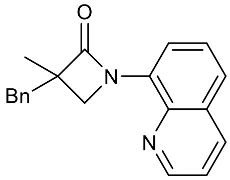

2u1

Compound **2u1**,[2] yellow oil, yield: 75%. ^{1}H NMR (500 MHz, $CDCl_3$) δ 1.46 (s, 3H), 2.96 (d, J = 13.9 Hz, 1H), 3.20 (d, J = 13.9 Hz, 1H), 4.30 (d, J = 7.3 Hz, 1H), 4.44

(d, J = 7.3 Hz, 1H), 7.18-7.22 (m, 1H), 7.27-7.32 (m, 4H), 7.34-7.37 (m, 1H), 7.45-7.50 (m, 2H), 8.07 (dd, J = 8.3, 1.7 Hz, 1H), 8.40-8.43 (m, 1H), 8.77 (dd, J = 4.1, 1.7 Hz, 1H).

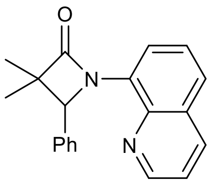

2u2

Compound **2u2**,[2] yellow oil, yield: 11%. ^{1}H NMR (500 MHz, $CDCl_3$) δ 0.92 (s, 3H), 1.65 (s, 3H), 6.08 (s, 1H), 7.12-7.17 (m, 1H), 7.19-7.21 (m, 4H), 7.23-7.26 (m, 1H), 7.54-7.56 (m, 2H), 8.02 (dd, J = 8.3, 1.7 Hz, 1H), 8.38-8.41 (m, 1H), 8.62 (dd , J = 4.1, 1.7 Hz, 1H).

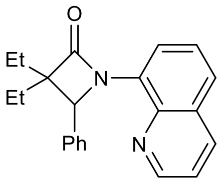

2v

Compound **2v**,[2] pale yellow oil, yield: 65%. ^{1}H NMR (500 MHz, $CDCl_3$) δ 0.81 (t, J = 7.5 Hz, 3H), 1.19 (t, J = 7.4 Hz, 3H), 1.22-1.30 (m, 1H), 1.49-1.57 (m, 1H), 2.01-2.11 (m, 2H), 6.14 (s, 1H), 7.09-7.13 (m, 1H), 7.15-7.20 (m, 3H), 7.21-7.25 (m, 2H), 7.50-7.52 (m, 2H), 7.95 (dd, J = 8.3, 1.7 Hz, 1H), 8.36-8.39 (m, 1H), 8.60 (dd, J = 4.1, 1.8 Hz, 1H).

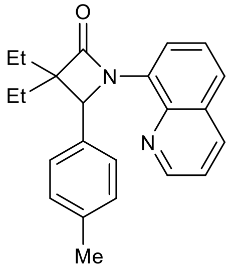

2w

Compound **2w**,[3] pale yellow oil, yield: 59%. ^{1}H NMR (500 MHz, $CDCl_3$) δ 0.83 (t, J = 7.5 Hz, 3H), 1.19 (t, J = 7.4 Hz, 3H), 1.22-1.30 (m, 1H), 1.51-1.58 (m, 1H), 2.01-

2.10 (m, 2H), 2.21 (s, 3H), 6.11 (s, 1H), 6.98 (d, *J* = 7.9 Hz, 2H), 7.12 (d, *J* = 8.0 Hz, 2H), 7.21-7.25 (m, 1H), 7.52-7.54 (m, 2H), 7.99 (dd, *J* = 8.3, 1.8 Hz, 1H), 8.33-8.36 (m, 1H), 8.64 (dd, *J* = 4.1, 1.8 Hz, 1H).

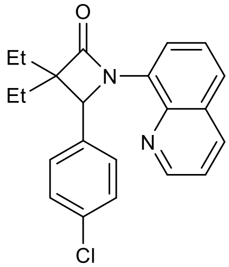

2x

Compound **2x**,[3] pale yellow solid, yield: 67%. ^{1}H NMR (500 MHz, $CDCl_3$) δ 0.83 (t, *J* = 7.5 Hz, 3H), 1.18 (t, *J* = 7.4 Hz, 3H), 1.22-1.29 (m, 1H), 1.49-1.56 (m, 1H), 1.99-2.10 (m, 2H), 6.12 (s, 1H), 7.13-7.18 (m, 4H), 7.21-7.24 (m, 1H), 7.52-7.54 (m, 2H), 7.99 (dd, *J* = 8.3, 1.8 Hz, 1H), 8.39-8.42 (m, 1H), 8.59 (dd, *J* = 4.1, 1.8 Hz, 1H).

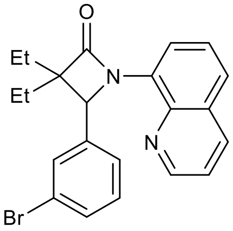

2y

Compound **2y**,[3] yellow solid, yield: 64%. ^{1}H NMR (500 MHz, $CDCl_3$) δ 0.84 (t, *J* = 7.5 Hz, 3H), 1.18 (t, *J* = 7.4 Hz, 3H), 1.22-1.30 (m, 1H), 1.49-1.56 (m, 1H), 2.00-2.10 (m, 2H), 6.10 (s, 1H), 7.02-7.05 (m, 1H), 7.13-7.16 (m, 1H), 7.23-7.27 (m, 2H), 7.38-7.41 (m, 1H), 7.53-7.56 (m, 2H), 8.01 (dd, *J* = 8.3, 1.4 Hz, 1H), 8.37-8.40 (m, 1H), 8.60-8.62 (m, 1H).

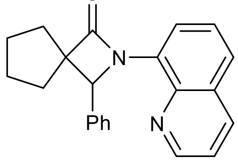

2z

Compound **2z**,[3] pale yellow oil, yield: 51%. ^{1}H NMR (500 MHz, $CDCl_3$) δ 1.21-1.27 (m, 1H), 1.37-1.44 (m, 1H), 1.62-1.75 (m, 2H), 1.80-1.89 (m, 2H), 2.25-2.39 (m, 2H), 6.13 (s, 1H), 7.12-7.16 (m, 1H), 7.18-7.25 (m, 5H), 7.51-7.54 (m, 2H), 7.98 (dd, J = 8.3, 1.7 Hz, 1H), 8.40-8.43 (m, 1H), 8.63 (dd, J = 4.1, 1.7 Hz, 1H).

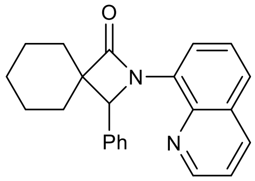

2aa

Compound **2aa**,[3] yellow oil, yield: 61%. ^{1}H NMR (500 MHz, $CDCl_3$) δ 1.00-1.09 (m, 1H), 1.29-1.35 (m, 1H), 1.40-1.52 (m, 3H), 1.59-1.72 (m, 2H), 1.88-1.96 (m, 1H), 2.04-2.11 (m, 1H), 2.15-2.22 (m, 1H), 6.04 (s, 1H), 7.10-7.14 (m, 1H), 7.16-7.21 (m, 3H), 7.26-7.29 (m, 2H), 7.48-7.53 (m, 2H), 7.96 (dd, J = 8.3, 1.7 Hz, 1H), 8.37-8.41 (m, 1H), 8.61 (dd, J = 4.1, 1.7 Hz, 1H).

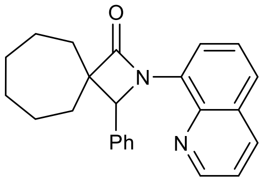

2ab

Compound **2ab**,[2] yellow solid, yield: 68%. ^{1}H NMR (500 MHz, $CDCl_3$) δ 1.19-1.27 (m, 1H), 1.33-1.39 (m, 2H), 1.44-1.52 (m, 1H), 1.56-1.79 (m, 5H), 1.93-2.00 (m, 1H), 2.26-2.35 (m, 2H), 6.09 (s, 1H), 7.10-7.14 (m, 1H), 7.16-7.25 (m, 5H), 7.50-7.52 (m, 2H), 7.97 (dd, J = 8.3, 1.7 Hz, 1H), 8.37-8.40 (m, 1H), 8.60 (dd, J = 4.1, 1.7 Hz, 1H).

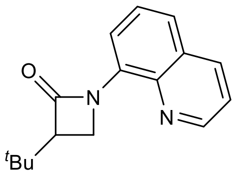

3a

Compound **3a**, colorless oil, yield: 75%. ^{1}H NMR (500 MHz, $CDCl_3$) δ 1.12 (s, 9H), 3.25 (dd, J = 5.8, 3.1 Hz, 1H), 4.30 (dd, J = 7.7, 3.1 Hz, 1H), 4.48 (dd, J = 7.7, 5.8

Hz, 1H), 7.37-7.40 (m, 1H), 7.47-7.53 (m, 2H), 8.10 (dd, J = 8.3, 1.7 Hz, 1H), 8.46-8.49 (m, 1H), 8.82 (dd, J = 4.1, 1.8 Hz, 1H); ^{13}C NMR (125 MHz, $CDCl_3$) δ 27.3, 31.4, 48.4, 61.9, 119.5, 121.3, 122.9, 126.9, 129.1, 135.1, 136.1, 140.6, 148.6, 169.4; IR (neat) ν 2956, 1738, 1504, 1474, 1401, 1348, 1151, 825, 788, 756; MS (ESI) (m/z): $[M+H]^+$ calcd for $C_{16}H_{19}N_2O$ 255.2, found 255.2.

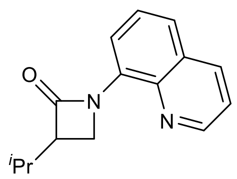

3b

Compound **3b**, colorless oil, yield: 52%. ^{1}H NMR (500 MHz, $CDCl_3$) δ 1.08 (d, J = 6.7 Hz, 3H), 1.18 (d, J = 6.7 Hz, 3H), 2.12-2.19 (m, 1H), 3.20-3.24 (m, 1H), 4.27 (dd, J = 7.5, 3.0 Hz, 1H), 4.54 (dd, J = 7.5, 5.7 Hz, 1H), 7.36-7.40 (m, 1H), 7.47-7.52 (m, 2H), 8.10 (dd, J = 8.3, 1.7 Hz, 1H), 8.45-8.49 (m, 1H), 8.82 (dd, J = 4.1, 1.7 Hz, 1H); ^{13}C NMR (125 MHz, $CDCl_3$) δ 20.2, 20.3, 28.6, 49.9, 58.0, 119.5, 121.3, 122.9, 126.9, 129.1, 135.2, 136.1, 140.6, 148.6, 169.9; IR (neat) ν 2958, 2926, 1738, 1500, 1474, 1401, 1340, 1151, 825, 789, 757; MS (ESI) (m/z): $[M+H]^+$ calcd for $C_{15}H_{17}N_2O$ 241.1, found 241.1.

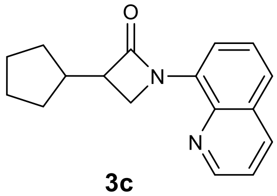

3c

Compound **3c**, pale yellow oil, yield: 71%. ^{1}H NMR (500 MHz, $CDCl_3$) δ 1.39-1.44 (m, 1H), 1.47-1.54 (m, 1H), 1.58-1.74 (m, 4H), 1.83-1.90 (m, 1H), 1.95-2.02 (m, 1H), 2.29-2.38 (m, 1H), 3.36-3.40 (m, 1H), 4.23 (dd, J = 7.5, 2.9 Hz, 1H), 4.58 (dd, J = 7.4, 5.7 Hz, 1H), 7.36-7.39 (m, 1H), 7.47-7.52 (m, 2H), 8.10 (dd, J = 8.3, 1.7 Hz, 1H), 8.45-8.49 (m, 1H), 8.81 (dd, J = 4.1, 1.7 Hz, 1H); ^{13}C NMR (125 MHz, $CDCl_3$) δ 25.2, 25.5, 30.3, 30.4, 39.7, 50.7, 55.6, 119.5, 121.3, 122.9, 126.9, 129.1, 135.3, 136.1, 140.6, 148.6, 170.2; IR (neat) ν 2952, 1738, 1504, 1473, 1401, 1342, 1150, 824, 788; MS (ESI) (m/z): $[M+H]^+$ calcd for $C_{17}H_{19}N_2O$ 267.2, found 267.2.

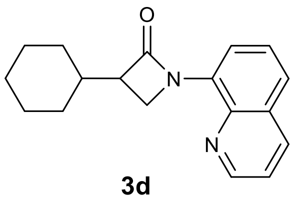

3d

Compound **3d**, pale yellow oil, yield: 69%. ^{1}H NMR (500 MHz, $CDCl_3$) δ 1.15-1.35 (m, 5H), 1.66-1.72 (m, 1H), 1.74-1.83 (m, 4H), 2.08-2.13 (m, 1H), 3.22-3.26 (m, 1H), 4.30 (dd, *J* = 7.5, 3.0 Hz, 1H), 4.52 (dd, *J* = 7.5, 5.7 Hz, 1H), 7.36-7.39 (m, 1H), 7.46-7.51 (m, 2H), 8.10 (dd, *J* = 8.3, 1.7 Hz, 1H), 8.45-8.48 (m, 1H), 8.81 (dd, *J* = 4.1, 1.8 Hz, 1H); ^{13}C NMR (125 MHz, $CDCl_3$) δ 26.1, 26.2, 26.5, 30.8, 30.9, 38.2, 49.8, 57.0, 119.4, 121.3, 122.9, 126.9, 129.1, 135.2, 136.0, 140.5, 148.6, 169.9; IR (neat) *v* 2923, 2851, 1738, 1504, 1474, 1401, 1346, 1150, 825, 788, 756; MS (ESI) (*m*/*z*): $[M+H]^+$ calcd for $C_{18}H_{21}N_2O$ 281.2, found 281.2.

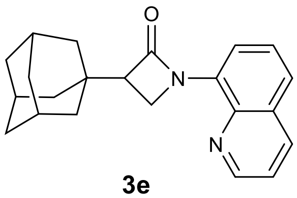

3e

Compound **3e**, yellow oil, yield: 74%. ^{1}H NMR (500 MHz, $CDCl_3$) δ 1.64-1.76 (m, 9H), 1.84-1.89 (m, 3H), 2.00-2.04 (m, 3H), 3.09-3.13 (m, 1H), 4.40 (d, *J* = 4.5 Hz, 2H), 7.36-7.39 (m, 1H), 7.46-7.51 (m, 2H), 8.10 (dd, *J* = 8.3, 1.8 Hz, 1H), 8.46-8.49 (m, 1H), 8.82 (dd, *J* = 4.1, 1.8 Hz, 1H); ^{13}C NMR (125 MHz, $CDCl_3$) δ 28.4, 33.4, 37.1, 40.0, 46.8, 62.4, 119.4, 121.3, 122.8, 126.9, 129.1, 135.2, 136.0, 140.5, 148.6, 169.2; IR (neat) *v* 2902, 2848, 1738, 1504, 1474, 1401, 1351, 1150, 825, 788; MS (ESI) (*m*/*z*): $[M+H]^+$ calcd for $C_{22}H_{25}N_2O$ 333.2, found 333.2.

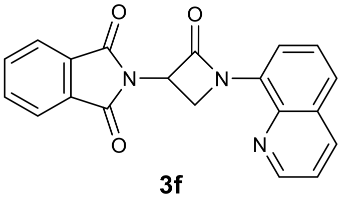

3f

Compound **3f**, white solid, yield: 43%. ^{1}H NMR (500 MHz, $CDCl_3$) δ 4.75 (dd, *J* = 7.4, 3.2 Hz, 1H), 5.08 (dd, *J* = 7.4, 6.0 Hz, 1H), 5.70 (dd, *J* = 6.0, 3.2 Hz, 1H), 7.39-7.43 (m, 1H), 7.53-7.62 (m, 2H), 7.75-7.78 (m, 2H), 7.87-7.90 (m, 2H), 8.14 (dd, *J*

= 8.3, 1.6 Hz, 1H), 8.54 (dd, *J* = 7.5, 1.4 Hz, 1H), 8.82 (dd, *J* = 4.1, 1.7 Hz, 1H); ^{13}C NMR (125 MHz, $CDCl_3$) δ 52.2, 55.2, 120.3, 121.5, 123.8 (2C), 124.0, 126.9, 129.1, 132.0, 134.6, 136.1, 140.6, 148.9, 164.3, 167.2; IR (neat) ν 3063, 2976, 1749, 1716, 1475, 1372, 1077, 913, 827, 792, 716; MS (ESI) (*m*/*z*): $[M+H]^+$ calcd for $C_{20}H_{14}N_3O_3$ 344.1, found 344.1.

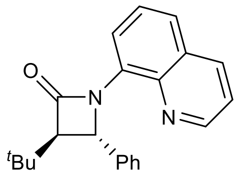

3g

Compound **3g**, yellow oil, yield: 78%. ^{1}H NMR (500 MHz, $CDCl_3$) δ 1.20 (s, 9H), 3.08 (d, *J* = 2.5 Hz, 1H), 6.07 (d, *J* = 2.5 Hz, 1H), 7.10-7.14 (m, 1H), 7.17-7.21 (m, 2H), 7.23-7.26 (m, 1H), 7.37 (d, *J* = 7.3 Hz, 2H), 7.47-7.50 (m, 2H), 7.97 (dd, *J* = 8.2, 1.7 Hz, 1H), 8.34-8.37 (m, 1H), 8.71 (dd, *J* = 4.1, 1.7 Hz, 1H); ^{13}C NMR (125 MHz, $CDCl_3$) δ 27.6, 32.2, 62.3, 72.2, 121.3, 121.9, 124.2, 126.2, 126.6, 127.4, 128.6, 129.1, 133.4, 135.7, 141.1, 141.5, 148.8, 168.9; IR (neat) ν 2957, 1742, 1504, 1473, 1399, 1342, 1143, 825, 789, 745, 697; MS (ESI) (*m*/*z*): $[M+H]^+$ calcd for $C_{22}H_{23}N_2O$ 331.2, found 331.2. The relative configuration was determined by NOESY analysis of this compound.

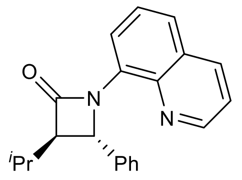

3h

Compound **3h**, yellow oil, yield: 61%. ^{1}H NMR (500 MHz, $CDCl_3$) δ 1.17 (d, *J* = 6.7 Hz, 3H), 1.22 (d, *J* = 6.7 Hz, 3H), 2.30-2.38 (m, 1H), 3.05 (dd, *J* = 8.2, 2.5 Hz, 1H), 6.00 (d, *J* = 2.4 Hz, 1H), 7.10-7.15 (m, 1H), 7.18-7.22 (m, 2H), 7.25-7.28 (m, 1H), 7.37 (d, *J* = 7.0 Hz, 2H), 7.48-7.52 (m, 2H), 7.99 (dd, *J* = 8.3, 1.7 Hz, 1H), 8.33-8.36 (m, 1H), 8.71 (dd, *J* = 4.1, 1.7 Hz, 1H); ^{13}C NMR (125 MHz, $CDCl_3$) δ 20.4, 20.7, 29.1, 63.9, 68.2, 121.3, 121.9, 124.2, 126.2, 126.7, 127.5, 128.6, 129.1, 133.5, 135.8, 141.0, 141.3, 148.8, 169.4; IR (neat) ν 2958, 1743, 1504, 1473, 1399, 1336, 1148, 825, 789, 749, 697; MS (ESI) (*m*/*z*): $[M+H]^+$ calcd for $C_{21}H_{21}N_2O$ 317.2,

found 317.2. The relative configuration was determined by NOESY analysis of this compound.

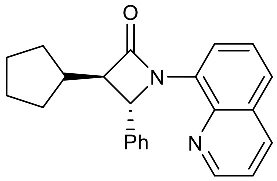

3i

Compound **3i**, yellow oil, yield: 65%. ^{1}H NMR (500 MHz, $CDCl_3$) δ 1.44-1.74 (m, 6H), 1.95-2.05 (m, 2H), 2.46-2.55 (m, 1H), 3.19 (dd, J = 8.5, 1.5 Hz, 1H), 5.97 (d, J = 1.4 Hz, 1H), 7.10-7.16 (m, 1H), 7.18-7.22 (m, 2H), 7.25-7.28 (m, 1H), 7.36 (d, J = 7.2 Hz, 2H), 7.48-7.52 (m, 2H), 7.79-8.01 (m, 1H), 8.34-8.37 (m, 1H), 8.69-8.72 (m, 1H); ^{13}C NMR (125 MHz, $CDCl_3$) δ 25.1, 25.5, 30.6 (2C), 40.0, 64.8, 66.0, 121.3, 121.9, 124.2, 126.1, 126.7, 127.5, 128.6, 129.1, 133.5, 135.8, 141.0, 141.3, 148.7, 169.7; IR (neat) ν 3356, 2951, 1633, 1525, 1485, 1323, 1159, 915, 825, 791, 740, 699; MS (ESI) (m/z): $[M+H]^+$ calcd for $C_{23}H_{23}N_2O$ 343.2, found 343.2. The relative configuration was determined by NOESY analysis of this compound.

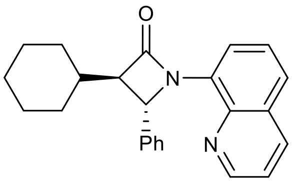

3j

Compound **3j**, yellow oil, yield: 70%. ^{1}H NMR (500 MHz, $CDCl_3$) δ 1.17-1.37 (m, 5H), 1.67-1.73 (m, 1H), 1.75-1.80 (m, 2H), 1.92-1.97 (m, 1H), 2.00-2.07 (m, 1H), 2.13-2.18 (m, 1H), 3.08 (dd, J = 8.3, 2.5 Hz, 1H), 6.03 (d, J = 2.4 Hz, 1H), 7.09-7.13 (m, 1H), 7.17-7.21 (m, 2H), 7.24-7.27 (m, 1H), 7.34-7.37 (m, 2H), 7.48-7.51 (m, 2H), 7.98 (dd, J = 8.3, 1.7 Hz, 1H), 8.34-8.37 (m, 1H), 8.70 (dd, J = 4.1, 1.7 Hz, 1H); ^{13}C NMR (125 MHz, $CDCl_3$) δ 26.1, 26.2, 26.5, 31.0, 31.2, 39.7, 63.8, 67.3, 121.3, 121.8, 124.1, 126.2, 126.7, 127.4, 128.6, 129.1, 133.5, 135.8, 141.0, 141.4, 148.7, 169.4; IR (neat) ν 2924, 2852, 1742, 1504, 1400, 1343, 1152, 1103, 825, 789, 735, 697; MS (ESI) (m/z): $[M+H]^+$ calcd for $C_{24}H_{25}N_2O$ 357.2, found 357.2. The relative configuration was determined by NOESY analysis of this compound.

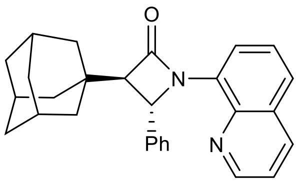

3k

Compound **3k**, yellow solid, yield: 68%. ^{1}H NMR (500 MHz, CDCl$_3$) δ 1.74-1.79 (m, 9H), 1.92-1.97 (m, 3H), 2.02-2.06 (m, 3H), 2.95 (d, J = 2.6 Hz, 1H), 6.15 (d, J = 2.5 Hz, 1H), 7.09-7.13 (m, 1H), 7.16-7.20 (m, 2H), 7.24-7.27 (m, 1H), 7.36 (d, J = 7.0 Hz, 2H), 7.48-7.51 (m, 2H), 7.99 (dd, J = 8.3, 1.7 Hz, 1H), 8.37-8.40 (m, 1H), 8.72 (dd, J = 4.1, 1.8 Hz, 1H); ^{13}C NMR (125 MHz, CDCl$_3$) δ 28.5, 34.3, 37.1, 40.3, 60.5, 72.8, 121.3, 121.6, 124.0, 126.2, 126.7, 127.3, 128.6, 129.1, 133.5, 135.7, 141.0, 141.8, 148.8, 168.7; IR (neat) ν 2903, 2848, 1740, 1504, 1399, 1343, 1131, 910, 825, 788, 733, 697; MS (ESI) (*m*/*z*): [M+H]$^+$ calcd for $C_{28}H_{29}N_2O$ 409.2, found 409.2. The relative configuration was determined by NOESY analysis of this compound.

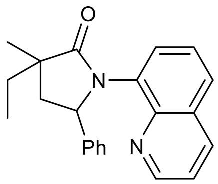

4a1

Compound **4a1**, yellow oil, yield: 83%. ^{1}H NMR (500 MHz, CDCl$_3$, a mixture of diastereomers in ratio 1.1:1, the minor one is marked with an *) δ 1.14 (t, J = 7.4 Hz, 3H), 1.54 (s, 3H), 1.90-1.97 (m, 2H), 2.00-2.08 (m, 1H), 2.28 (dd, J = 12.7, 6.2 Hz, 1H), 5.28-5.34 (m, 1H), 7.18-7.24 (m, 5H), 7.26-7.29 (m, 1H), 7.30-7.33 (m, 1H), 7.41-7.46 (m, 2H), 8.03-8.06 (m, 1H), 8.89-8.91 (m, 1H); 1.23 (t, J = 7,4 Hz, 3H)*, 1.57 (s, 2H)*, 1.90-1.97 (m, 1H)*, 2.00-2.08 (m, 1H)*, 2.15 (dd, J = 12.6, 10.3 Hz, 1H)*, 2.58 (dd, J = 12.9, 6.7 Hz, 1H)*, 5.28-5.34 (m, 1H)*, 7.18-7.24 (m, 5H)*, 7.26-7.29 (m, 1H)*, 7.30-7.33 (m, 1H)*, 7.41-7.46 (m, 2H)*, 8.03-8.06 (m, 1H)*, 8.89-8.91 (m, 1H)*; ^{13}C NMR (125 MHz, CDCl$_3$) δ 9.1, 24.4, 31.5, 44.4, 45.5, 80.2, 119.9, 120.9, 122.6, 125.6, 126.6, 127.9, 128.4, 129.2, 135.9, 140.9, 141.7, 146.4, 149.2, 168.7; 9.2*, 24.6*, 32.2*, 45.4*, 45.8*, 80.2*, 120.0*, 120.9*, 122.6*, 125.7*, 126.6*, 127.9*, 128.4*, 129.2*, 135.9*, 141.3*, 141.8*, 146.6*, 149.3*, 168.8*; IR (neat) ν 2967, 1767, 1705, 1496, 1462, 1372, 1188, 1020, 940, 821, 794, 756, 699; MS (ESI) (*m*/*z*): [M+H]$^+$ calcd for $C_{22}H_{23}N_2O$ 331.2, found 331.2.

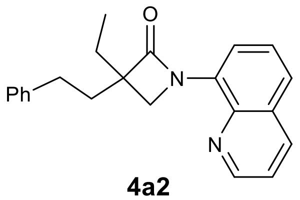

4a2

Compound **4a2**,[3] pale yellow oil, yield: 8%. ^{1}H NMR (500 MHz, $CDCl_3$) δ 1.10 (t, *J* = 7.5 Hz, 3H), 1.85-1.94 (m, 2H), 2.04-2.14 (m, 2H), 2.70-2.92 (m, 2H), 4.38 (dd, *J* = 15.0, 7.5 Hz, 2H), 7.15-7.19 (m, 1H), 7.20-7.28 (m, 4H), 7.36 (dd, *J* = 8.3, 4.1 Hz, 1H), 7.46-7.51 (m, 2H), 8.08 (dd, *J* = 8.3, 1.8 Hz, 1H), 8.48-8.53 (m, 1H), 8.81 (dd, *J* = 4.1, 1.8 Hz, 1H).

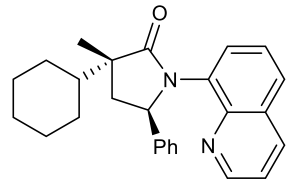

4b1

Compound **4b1**, yellow oil, yield: 72%. ^{1}H NMR (500 MHz, $CDCl_3$) δ 1.14-1.50 (m, 5H), 1.55 (s, 3H), 1.73-1.79 (m, 1H), 1.86-1.97 (m, 4H), 2.12-2.21 (m, 2H), 2.73-2.78 (m, 1H), 5.24-5.29 (m, 1H), 7.19-7.25 (m, 5H), 7.28-7.31 (m, 1H), 7.33-7.36 (m, 1H), 7.43-7.47 (m, 2H), 8.06-8.09 (m, 1H), 8.92-8.94(m, 1H); ^{13}C NMR (125 MHz, $CDCl_3$) δ 24.6, 26.8 (2C), 26.9, 27.7, 29.0, 42.8, 45.9, 48.8, 80.9, 120.1, 121.0, 122.6, 125.5, 126.7, 127.8, 128.5, 129.2, 136.21, 141.8, 142.0,146.5, 149.2, 169.5; IR (neat) *v* 2927, 2852, 1706, 1495, 1450, 1217, 1024, 909, 792, 756, 732, 699; MS (ESI) (*m*/*z*): $[M+H]^+$ calcd for $C_{26}H_{29}N_2O$ 385.2, found 385.2. The relative configuration was determined by NOESY analysis of this compound.

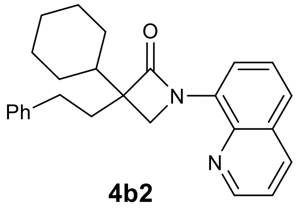

4b2

Compound **4b2**, pale yellow oil, yield: 17%. ^{1}H NMR (500 MHz, $CDCl_3$) δ 1.09-1.21 (m, 2H), 1.22-1.34 (m, 3H), 1.66-1.72 (m, 1H), 1.76-1.87 (m, 4H), 1.96-2.01

(m, 1H), 2.06-2.11 (m, 2H), 2.74-2.89 (m, 2H), 4.33 (d, *J* = 7.6 Hz, 1H), 4.44 (d, *J* = 7.6 Hz, 1H), 7.14-7.19 (m, 1H), 7.21-7.28 (m, 4H), 7.36-7.40 (m, 1H), 7.47-7.52 (m, 2H), 8.08-8.12 (m, 1H), 8.50-8.53 (m, 1H), 8.81-8.83 (m, 1H); ^{13}C NMR (125 MHz, $CDCl_3$) δ 26.5, 26.6 (2C), 27.9, 28.3, 31.1, 33.5, 40.76, 54.0, 63.3, 119.5, 121.3, 122.9, 126.0, 126.9, 128.5 (2C), 129.2, 134.8, 136.1, 140.6, 142.3, 148.6, 172.3; IR (neat) ν 3453, 2927, 2852, 1737, 1504, 1474, 1402, 1346, 1151, 909, 825, 789, 699, 626; MS (ESI) (*m*/*z*): $[M+H]^+$ calcd for $C_{26}H_{29}N_2O$ 385.2, found 385.2.

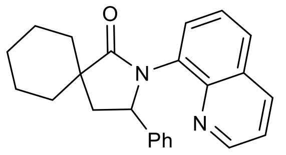

4c

Compound **4c**, yellow oil, yield: 86%. ^{1}H NMR (500 MHz, $CDCl_3$) δ 1.26-1.34 (m, 1H), 1.40-1.54 (m, 2H), 1.69-1.74 (m, 1H), 1.80-1.90 (m, 3H), 1.96-2.03 (m, 3H), 2.23-2.31 (m, 1H), 2.73 (dd, J = 12.9, 6.4 Hz, 1H), 5.32 (dd, J = 9.8, 6.4 Hz, 1H), 7.20-7.26 (m, 5H), 7.31-7.38 (m, 2H), 7.42-7.49 (m, 2H), 8.10 (dd, J = 8.3, 1.4 Hz, 1H), 8.95-8.98 (m, 1H); ^{13}C NMR (125 MHz, $CDCl_3$) δ 22.2, 22.3, 25.4, 31.7, 34.3, 42.4, 45.4, 78.1, 110.3, 116.2, 121.5, 125.4, 127.5, 128.4, 128.8, 130.2, 136.2, 138.5, 140.0, 144.0, 147.5, 181.4; IR (neat) ν 3370, 2931, 2856, 1763, 1704, 1505, 1372, 1189, 1022, 821, 791, 746, 699; MS (ESI) (*m*/*z*): $[M+H]^+$ calcd for $C_{24}H_{25}N_2O$ 357.2, found 357.2.

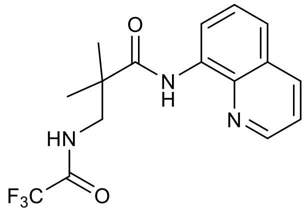

5a

Compound **5a**, colorless oil, yield: 29%. ^{1}H NMR (500 MHz, $CDCl_3$) δ 1.49 (s, 6H), 3.58 (d, *J* = 6.1 Hz, 2H), 7.46-7.55 (m, 3H), 7.74 (brs, 1H), 8.17 (dd, *J* = 8.3, 1.4 Hz, 1H), 8.68 (dd, *J* = 8.8, 4.4 Hz, 1H), 8.82 (dd, *J* = 4.1, 1.5 Hz, 1H), 10.28 (brs, 1H); ^{13}C NMR (125 MHz, $CDCl_3$) δ 24.1, 44.0, 47.7, 116.2 (q, J_{CF} = 288 Hz), 116.8, 121.9, 122.3, 127.3, 128.1, 133.9, 136.6, 138.8, 148.7, 157.7 (q, J_{CCF} = 36.8 Hz), 175.9; ^{19}F NMR (471 MHz, $CDCl_3$) δ -75.9; IR (neat) ν 3344, 2972, 2668, 1720,

1668, 1538, 1488, 1232, 1122, 915, 827, 790, 756; MS (ESI) (*m*/*z*): $[M+H]^+$ calcd for $C_{16}H_{17}F_3N_3O_2$ 340.1, found 340.1.

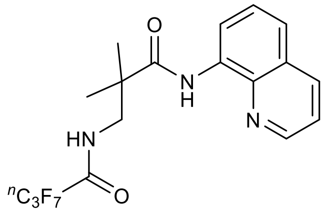

5b

Compound **5b**, colorless oil, yield: 64%. ^1H NMR (500 MHz, $CDCl_3$) δ 1.49 (s, 6H), 3.61 (d, J = 6.1 Hz, 2H), 7.46-7.56 (m, 3H), 7.88 (brs, 1H), 8.18 (dd, J = 8.3, 1.6 Hz, 1H), 8.67-8.71 (m, 1H), 8.83 (dd, J = 4.2, 1.6 Hz, 1H), 10.29 (brs, 1H); ^{13}C NMR (125 MHz, $CDCl_3$) δ 24.1, 44.0, 47.9, 105.8-111.2 (m, 2C), 116.8, 117.5 (qt, J_{CF} = 288 Hz, J_{CCF} = 33.7 Hz, 1C), 121.9, 122.3, 127.4, 128.1, 133.9, 136.6, 138.8, 148.7, 158.0 (t, J_{CCF} = 25.7 Hz, 1C), 176.0; ^{19}F NMR (471 MHz, $CDCl_3$) δ -80.5 (3F), -120.7 (2F), -126.8 (2F); IR (neat) ν 3348, 3068, 2971, 2877, 1717, 1669, 1539, 1489, 1232, 1122, 921, 826, 791, 756; MS (ESI) (*m*/*z*): $[M+H]^+$ calcd for $C_{18}H_{17}F_7N_3O_2$ 440.1, found 440.1.

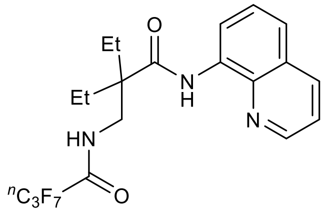

5c

Compound **5c**, colorless oil, yield: 53%. ^1H NMR (500 MHz, $CDCl_3$) δ 1.04 (t, J = 7.5 Hz, 6H), 1.80-1.94 (m, 4H), 3.68 (d, J = 6.0 Hz, 2H), 7.46-7.59 (m, 3H), 7.84 (brs, 1H), 8.19 (dd, J = 8.3, 1.5 Hz, 1H), 8.70-8.74 (m, 1H), 8.83 (dd, J = 4.2, 1.6 Hz, 1H), 10.26 (brs, 1H); ^{13}C NMR (125 MHz, $CDCl_3$) δ 8.6, 27.7, 43.2, 51.0, 105.8-111.2 (m, 2C), 116.9, 117.6 (qt, J_{CF} = 286 Hz, J_{CCF} = 33.2 Hz, 1C), 121.9, 122.2, 127.5, 128.2, 133.9, 136.6, 138.9, 148.7, 157.8 (t, J_{CCF} = 25.7 Hz, 1C), 175.4; ^{19}F NMR (471 MHz, $CDCl_3$) δ -80.5 (3F), -120.7 (2F), -126.9 (2F); IR (neat) ν 3348, 2972, 2931, 2884, 1725, 1668, 1538, 1487, 1232, 960, 904, 826, 791, 757; MS (ESI) (*m*/*z*): $[M+H]^+$ calcd for $C_{20}H_{21}F_7N_3O_2$ 468.2, found 468.2.

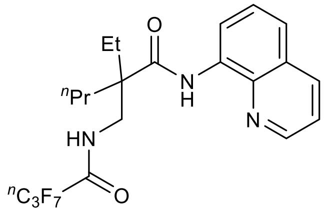

5d

Compound **5d**, pale yellow oil, yield: 50%. ^{1}H NMR (500 MHz, $CDCl_3$) δ 0.96 (t, J = 7.2 Hz, 3H), 1.04 (t, J = 7.5 Hz, 3H), 1.39-1.49 (m, 2H), 1.69-1.93 (m, 4H), 3.64-3.72 (m, 2H), 7.47-7.50 (m, 1H), 7.54-7.58 (m, 2H), 7.88 (brs, 1H), 8.19 (dd, J = 8.3, 1.5 Hz, 1H), 8.68-8.73 (m, 1H), 8.84 (dd, J = 4.2, 1.5 Hz, 1H), 10.27 (brs, 1H); ^{13}C NMR (125 MHz, $CDCl_3$) δ 8.6, 14.7, 17.6, 28.2, 37.5, 43.5, 50.8, 106.0-111.2 (m, 2C), 116.9, 117.6 (qt, J_{CF} = 286 Hz, J_{CCF} = 33.6 Hz, 1C), 121.9, 122.2, 127.4, 128.2, 133.9, 136.6, 138.9, 148.7, 157.8 (t, J_{CCF} = 25.6 Hz, 1C), 175.5; ^{19}F NMR (471 MHz, $CDCl_3$) δ -80.5 (3F), -120.7 (2F), -126.9 (2F); IR (neat) ν 3348, 2965, 2936, 1725, 1668, 1539, 1488, 1233, 1122, 964, 908, 826, 791, 757; MS (ESI) (m/z): $[M+H]^+$ calcd for $C_{21}H_{23}F_7N_3O_2$ 482.2, found 482.2.

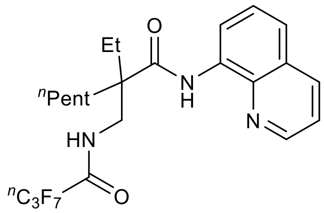

5e

Compound **5e**, pale yellow oil, yield: 45%. ^{1}H NMR (500 MHz, $CDCl_3$) δ 0.85 (t, J = 7.1 Hz, 3H), 1.04 (t, J = 7.5 Hz, 3H), 1.27-1.35 (m, 4H), 1.37-1.45 (m, 2H), 1.73-1.93 (m, 4H), 3.63-3.72 (m, 2H), 7.47-7.51 (m, 1H), 7.54-7.59 (m, 2H), 7.88 (brs, 1H), 8.19 (dd, J = 8.3, 1.7 Hz, 1H), 8.68-8.72 (m, 1H), 8.83 (dd, J = 4.2, 1.7 Hz, 1H), 10.26 (brs, 1H); ^{13}C NMR (125 MHz, $CDCl_3$) δ 8.6, 14.1, 22.5, 23.8, 28.3, 32.5, 35.2, 43.6, 50.7, 105.8-111.2 (m, 2C), 116.9, 117.7 (qt, J_{CF} = 286 Hz, J_{CCF} = 33.2 Hz, 1C), 122.0, 122.2, 127.5, 128.1, 133.9, 136.6, 138.9, 148.7, 157.7 (t, J_{CCF} = 25.7 Hz, 1C), 175.6; ^{19}F NMR (471 MHz, $CDCl_3$) δ -80.5 (3F), -120.7 (2F), -126.9 (2F); IR (neat) ν 3348, 2934, 2872, 1725, 1668, 1533, 1487, 1232, 1122, 906, 826, 791, 757; MS (ESI) (m/z): $[M+H]^+$ calcd for $C_{23}H_{27}F_7N_3O_2$ 510.2, found 510.2.

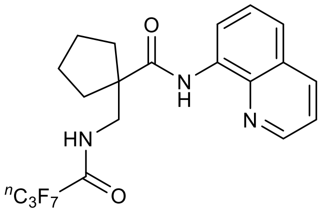

5f

Compound **5f**, colorless oil, yield: 57%. ^{1}H NMR (500 MHz, $CDCl_3$) δ 1.85-1.99 (m, 4H), 2.01-2.09 (m, 2H), 2.15-2.21 (m, 2H), 3.64 (d, J = 6.0 Hz, 2H), 7.46-7.50 (m, 1H), 7.53-7.58 (m, 2H), 7.88 (brs, 1H), 8.18 (dd, J = 8.3, 1.7 Hz, 1H), 8.66-8.70 (m, 1H), 8.82 (dd, J = 4.2, 1.7 Hz, 1H), 10.20 (brs, 1H); ^{13}C NMR (125 MHz, $CDCl_3$) δ 25.8, 35.6, 45.8, 55.2, 105.8-111.2 (m, 2C), 116.7, 117.6 (qt, J_{CF} = 286 Hz, J_{CCF} = 33.1 Hz, 1C), 121.9, 122.1, 127.4, 128.1, 134.1, 136.6, 138.8, 148.7, 158.1 (t, J_{CCF} = 25.8 Hz, 1C), 176.8; ^{19}F NMR (471 MHz, $CDCl_3$) δ -80.5 (3F), -120.8 (2F), -126.8 (2F); IR (neat) v 3344, 2959, 2876, 1724, 1669, 1532, 1488, 1231, 1122, 965, 905, 826, 791, 756; MS (ESI) (m/z): $[M+H]^+$ calcd for $C_{20}H_{19}F_7N_3O_2$ 466.1, found 466.2.

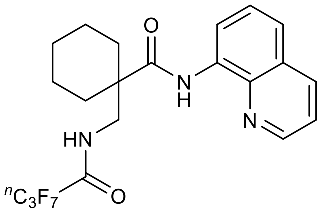

5g

Compound **5g**, colorless oil, yield: 47%. ^{1}H NMR (500 MHz, $CDCl_3$) δ 1.55-1.78 (m, 8H), 2.06-2.13 (m, 2H), 3.68 (d, J = 5.7 Hz, 2H), 7.47-7.51 (m, 1H), 7.54-7.58 (m, 2H), 7.70 (brs, 1H), 8.19 (dd, J = 8.3, 1.6 Hz, 1H), 8.72-8.76 (m, 1H), 8.84 (dd, J = 4.2, 1.6 Hz, 1H), 10.44 (brs, 1H); ^{13}C NMR (125 MHz, $CDCl_3$) δ 22.1, 25.7, 32.0, 45.2, 47.5, 105.8-111.2 (m, 2C), 117.1, 117.6 (qt, J_{CF} = 286 Hz, J_{CCF} = 33.7 Hz, 1C), 121.9, 122.4, 127.4, 128.1, 133.9, 136.6, 139.0, 148.7, 157.8 (t, J_{CCF} = 25.9 Hz, 1C), 175.2; ^{19}F NMR (471 MHz, $CDCl_3$) δ -80.6 (3F), -120.7 (2F), -126.9 (2F); IR (neat) v 3349, 2933, 2859, 1723, 1662, 1532, 1487, 1231, 1122, 916, 826, 791, 756; MS (ESI) (m/z): $[M+H]^+$ calcd for $C_{21}H_{21}F_7N_3O_2$ 480.2, found 480.2.

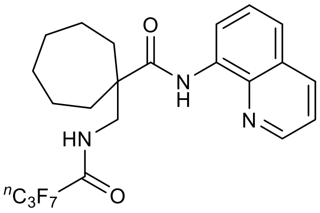

5h

Compound **5h**, pale yellow oil, yield: 52%. ^{1}H NMR (500 MHz, $CDCl_3$) δ 1.63-1.86 (m, 10H), 2.13-2.19 (m, 2H), 3.63 (d, J = 5.9 Hz, 2H), 7.47-7.50(m, 1H), 7.54-7.60 (m, 2H), 7.84 (brs, 1H), 8.19 (dd, J = 8.3, 1.7 Hz, 1H), 8.68-8.72 (m, 1H), 8.84 (dd, J = 4.2, 1.7 Hz, 1H), 10.39 (brs, 1H); ^{13}C NMR (125 MHz, $CDCl_3$) δ 24.0, 30.8, 34.7, 46.4, 50.7, 105.8-111.2 (m, 2C), 116.9, 117.6 (qt, J_{CF} = 286 Hz, J_{CCF} = 33.7 Hz, 1C), 121.9, 122.2, 127.4, 128.1, 134.2, 136.6, 138.9, 148.7, 157.9 (t, J_{CCF} = 25.7 Hz, 1C), 176.8; ^{19}F NMR (471 MHz, $CDCl_3$) δ -80.5 (3F), -120.7 (2F), -126.8 (2F); IR (neat) ν 3347, 2929, 2858, 1723, 1668, 1532, 1487, 1232, 1122, 907, 826, 791, 756; MS (ESI) (*m*/*z*): $[M+H]^+$ calcd for $C_{22}H_{23}F_7N_3O_2$ 494.2, found 494.2.

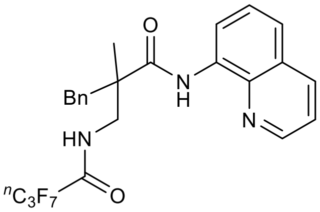

5i

Compound **5i**, yellow oil, yield: 66%. ^{1}H NMR (500 MHz, $CDCl_3$) δ 1.49 (s, 3H), 2.99 (d, J = 13.7 Hz, 1H), 3.18 (d, J = 13.7 Hz, 1H), 3.60 (dd, J = 13.5, 6.0 Hz, 1H), 3.71 (dd, J = 13.5, 6.0 Hz, 1H), 7.13-7.21 (m, 5H), 7.44-7.47 (m, 1H), 7.55-7.59 (m, 2H), 7.91 (brs, 1H), 8.17 (dd, J = 8.3, 1.5 Hz, 1H), 8.71-8.74 (m, 2H), 10.15 (brs, 1H); ^{13}C NMR (125 MHz, $CDCl_3$) δ 21.1, 44.0, 47.0, 48.3, 105.8-111.2 (m, 2C), 116.9, 117.6 (qt, J_{CF} = 286 Hz, J_{CCF} = 33.5 Hz, 1C), 121.9, 122.4, 127.2, 127.4, 128.1, 128.6, 130.2, 133.7, 135.7, 136.5, 138.8, 148.6, 158.0 (t, J_{CCF} = 25.8 Hz, 1C), 174.7; ^{19}F NMR (471 MHz, $CDCl_3$) δ -80.5 (3F), -120.7 (2F), -126.8 (2F); IR (neat) ν 3344, 3064, 3030, 2971, 1723, 1668, 1538, 1488, 1232, 1122, 908, 826, 791, 738, 701; MS (ESI) (*m*/*z*): $[M+H]^+$ calcd for $C_{24}H_{21}F_7N_3O_2$ 516.2, found 516.1.

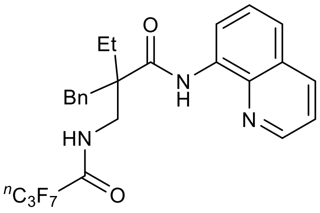

5j

Compound **5j**, yellow oil, yield: 55%. ^{1}H NMR (500 MHz, $CDCl_3$) δ 1.14 (t, J = 7.5 Hz, 3H), 1.82-1.90 (m, 1H), 1.92-2.00 (m, 1H), 3.11 (s, 2H), 3.64-3.76 (m, 2H), 7.14-7.19 (m, 5H), 7.44-7.47 (m, 1H), 7.54-7.59 (m, 2H), 7.91 (brs, 1H), 8.18 (dd, J = 8.3, 1.7 Hz, 1H), 8.70-8.74 (m, 2H), 10.15 (brs, 1H); ^{13}C NMR (125 MHz, $CDCl_3$) δ 8.7, 28.1, 41.6, 44.0, 51.7, 105.8-111.2 (m, 2C), 116.9, 117.7 (qt, J_{CF} = 286 Hz, J_{CCF} = 33.3 Hz, 1C), 121.9, 122.3, 127.2, 127.4, 128.1, 128.6, 130.3, 133.6, 135.6, 136.5, 138.8, 148.6, 157.8 (t, J_{CCF} = 25.8 Hz, 1C), 174.5; ^{19}F NMR (471 MHz, $CDCl_3$) δ -80.5 (3F), -120.6 (2F), -126.7 (2F); IR (neat) v 3346, 3063, 2970, 2930, 1725, 1669, 1532, 1487, 1232, 1122, 970, 907, 826, 791, 740, 701; MS (ESI) (m/z): $[M+H]^+$ calcd for $C_{25}H_{23}F_7N_3O_2$ 530.2, found 530.2.

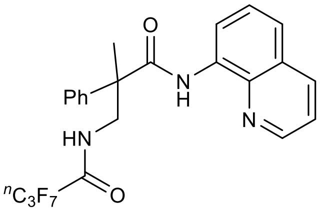

5k1

Compound **5k1**, yellow oil, yield: 32%. ^{1}H NMR (500 MHz, $CDCl_3$) δ 1.89 (s, 3H), 3.82-3.93 (m, 2H), 7.33-7.37 (m, 1H), 7.37-7.43 (m, 3H), 7.44-7.47 (m, 2H), 7.51-7.58 (m, 2H), 7.87 (brs, 1H), 8.12 (dd, J = 8.3, 1.6 Hz, 1H), 8.61 (dd, J = 4.2, 1.7 Hz, 1H), 8.73 (dd, J = 7.4, 1.5 Hz, 1H), 9.87 (brs, 1H); ^{13}C NMR (125 MHz, $CDCl_3$) δ 21.6, 48.5, 52.6, 105.8-111.2 (m, 2C), 116.7, 117.6 (qt, J_{CF} = 286 Hz, J_{CCF} = 33.4 Hz, 1C), 121.8, 122.3, 126.7, 127.3, 128.1, 128.3, 129.3, 134.0, 136.4, 138.7, 140.3, 148.6, 157.8 (t, J_{CCF} = 25.8 Hz, 1C), 175.2; ^{19}F NMR (471 MHz, $CDCl_3$) δ -80.5 (3F), -120.7 (2F), -126.8 (2F); IR (neat) v 3335, 3060, 2977, 2928, 1726, 1674, 1532, 1487, 1232, 1122, 905, 826, 791, 758, 700; MS (ESI) (m/z): $[M+H]^+$ calcd for $C_{23}H_{19}F_7N_3O_2$ 502.1, found 502.1.

Deuterium Labeling Experiment

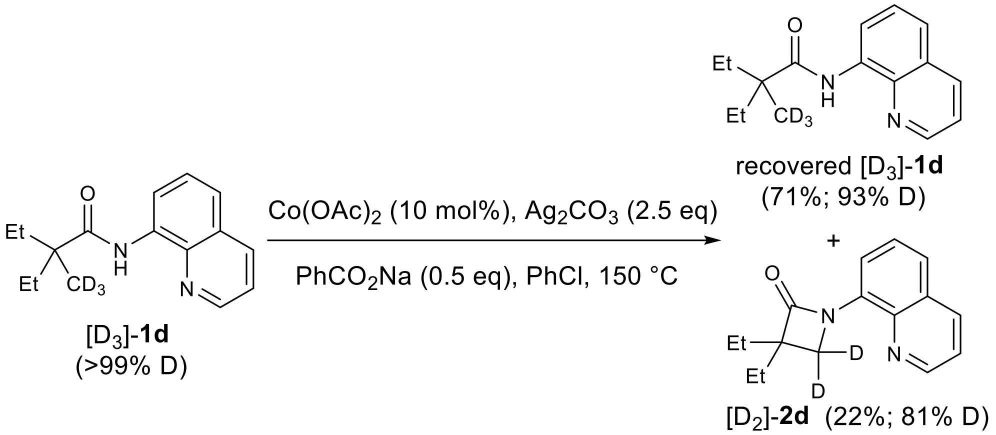

A 20 mL tube was charged with $[D_3]$-**1d** (77.8 mg, 0.30 mmol), $Co(OAc)_2$ (5.3 mg, 0.030 mmol), Ag_2CO_3 (207 mg, 0.75 mmol), $PhCO_2Na$ (21.6 mg, 0.15 mmol) and 0.60 mL of PhCl. The reaction mixture was stirred rigorously open to the air at 150 ˚C for 12 h. Then the mixture was cooled to room temperature, diluted with EtOAc (2 mL), filtered through a celite pad, and concentrated in vacuo. The residue was purified by flash chromatography on silica gel (gradient eluent of 2% EtOAc in hexanes, v/v) to give the desired product $[D_2]$-**2d** and recovered $[D_3]$-**1d**. The ratio of deuterium was determined by ^1H NMR.

$[D_2]$-**2d**,[2] colorless oil, yield: 22%. ^1H NMR (500 MHz, $CDCl_3$) δ 1.07 (t, J = 7.5 Hz, 6H), 1.78-1.85 (m, 4H), 4.28-4.32 (m, 0.38H), 7.36-7.39 (m, 1H), 7.47-7.51 (m, 2H), 8.10 (dd, J = 8.3, 1.7 Hz, 1H), 8.48-8.51 (m, 1H), 8.81 (dd, J = 4.1, 1.8 Hz, 1H).

Recovered $[D_3]$-**1d**,[2] colorless oil, yield: 71%. ^1H NMR (500 MHz, $CDCl_3$) δ 0.94 (t, J = 7.5 Hz, 6H), 1.31-1.36 (m, 0.21H), 1.59-1.67 (m, 2H), 1.84-1.93 (m, 2H), 7.42-7.55 (m, 3H), 8.15 (dd, J = 8.3, 1.6 Hz, 1H), 8.80-8.84 (m, 2H), 10.22 (brs, 1H).

Parallel KIE Experiment

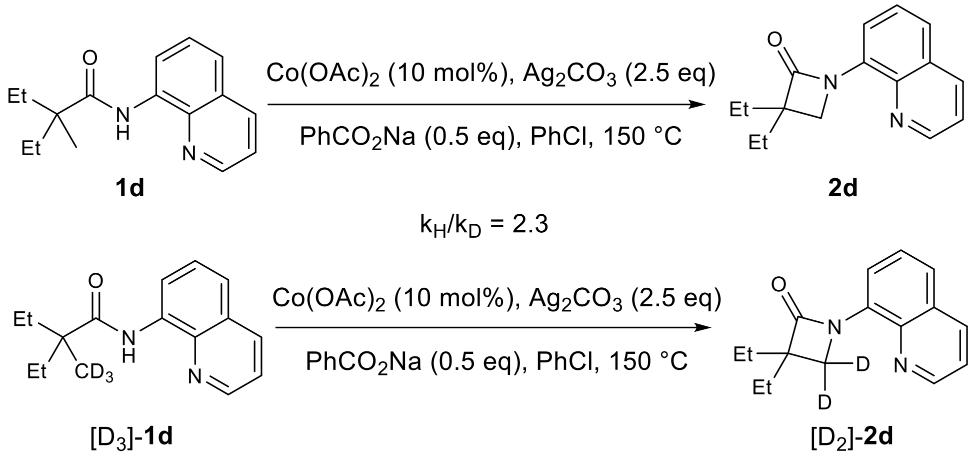

A 20 mL Schlenk tube was charged with **1d** (76.8 mg, 0.30 mmol) or [D_3]-**1d** (77.8 mg, 0.30 mmol), $Co(OAc)_2$ (5.3 mg, 0.030 mmol), Ag_2CO_3 (207 mg, 0.75 mmol), $PhCO_2Na$ (21.6 mg, 0.15 mmol) and 0.60 mL of PhCl. The reaction mixture was stirred rigorously open to the air at 150 °C. The reaction was stopped by rapid cooling in the indicated reaction period, and analyzed by GC using benzophenone as the internal standard. The GC yield was calculated after calibrating the response of GC.

Time (min)	10	20	30	40	50	60	70	80	90
Yield of **2d** (%)	0.6	2.1	4.4	4.7	6.0	7.3	9.6	12.0	12.8
Yield of [D_2]-**2d** (%)	0.1	0.6	1.0	2.2	1.7	3.3	3.4	4.0	5.9

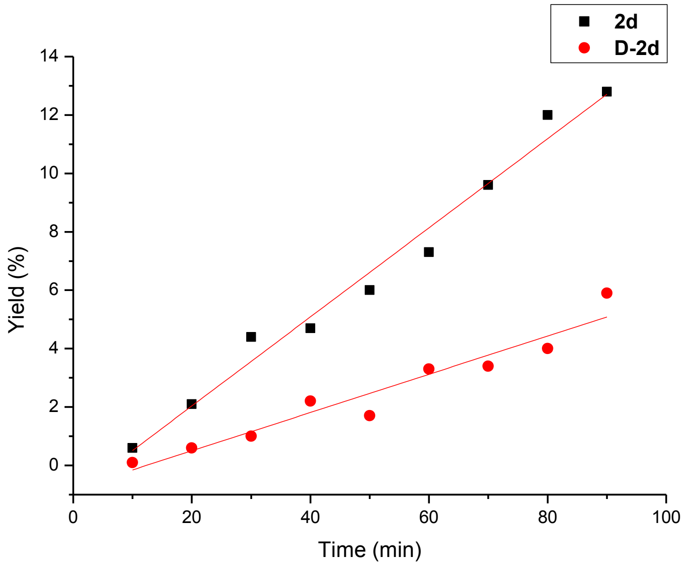

Equation for **2d**: $y = 0.1525x – 1.0139$ $R^2 = 0.9790$

Equation for [D_2]-**2d**: $y = 0.0655x – 0.8083$ $R^2 = 0.9230$

$k_H/k_D = 0.1525/0.0655 \approx 2.3$

KIE value determined from parallel reactions is 2.3.

General Procedure for Removal of the Quinolyl Group[2,3]

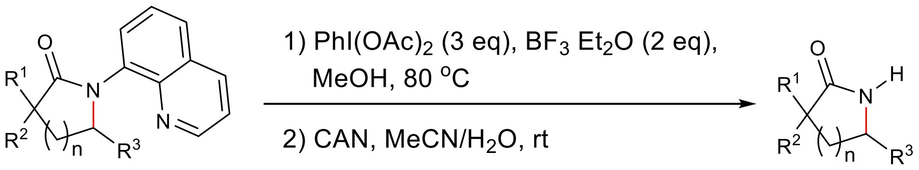

2, n = 0 or **4**, n = 1 **6** or **7**

$PhI(OAc)_2$ (145 mg, 0.45 mmol) was added to a stirred solution of lactam **2** or **4** with quinolyl group (0.15 mmol) and $BF_3 \cdot Et_2O$ (0.038 mL, 0.30 mmol) in MeOH (0.5 mL) at room temperature. After stirring at room temperature for 30 mins, the reaction mixture was refluxed at 80 ˚C for 3-8 hrs. Then the mixture was quenched with saturated aqueous $NaHCO_3$ at 0 °C, extracted with ethyl acetate (10 mL x 3). The combined organic layers were washed with brine, dried over Na_2SO_4, and then evaporated in vacuo to give the crude methoxylated product.

The crude methoxylated product was dissolved in a mixture of CH_3CN/H_2O (2.0 mL/0.4 mL) at room temperature. After the addition of CAN (247 mg, 0.45 mmol), the reaction mixture was stirred at room temperature for 6 hrs. Then the reaction mixture was diluted with water (5 mL), extracted with ethyl acetate, washed with water, saturated aqueous $NaHCO_3$ and brine. The organic layer was dried over anhydrous Na_2SO_4. After removal of the solvent, the residue was purified by flash chromatography on silica gel (gradient eluent of 20% EtOAc in hexanes, v/v) to give the deprotected lactam **6** or **7**.

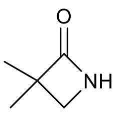

6a

Compound **6a**,[2] pale yellow oil, yield: 60%. ^1H NMR (500 MHz, $CDCl_3$) δ 1.31 (s, 6H), 3.12 (s, 2H), 5.81 (brs, 1H).

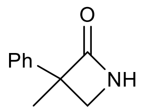

6b

Compound **6b**,[3] yellow oil, yield: 62%. ^{1}H NMR (500 MHz, $CDCl_3$) δ 1.70 (s, 3H), 3.46 (d, J = 5.2 Hz, 1H), 3.60 (d, J = 5.2 Hz, 1H), 5.80 (brs, 1H), 7.25-7.29 (m, 1H), 7.33-7.38 (m, 2H), 7.41-7.44 (m, 2H).

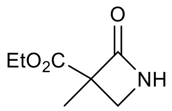

6c

Compound **6c**, yellow oil, yield: 51%. ^{1}H NMR (500 MHz, $CDCl_3$) δ 1.30 (t, J = 7.1 Hz, 3H), 1.62 (s, 3H), 3.16 (d, J = 5.4 Hz, 1H), 3.78 (d, J = 5.4 Hz, 1H), 4.24 (q, J = 7.1 Hz, 2H), 5.80 (brs, 1H); ^{13}C NMR (125 MHz, $CDCl_3$) δ 14.3, 17.2, 475, 61.4, 61.9, 167.2, 169.9; IR (neat) *v* 3284, 2980, 2934, 1769, 1729, 1458, 1387, 1290, 1175, 1022; MS (ESI) (*m*/*z*): $[M+H]^+$ calcd for $C_7H_{12}NO_3$ 158.1, found 158.1.

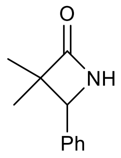

6d

Compound **6d**, yellow oil, yield: 55%. ^{1}H NMR (500 MHz, $CDCl_3$) δ 0.78 (s, 3H), 1.47 (s, 3H), 4.51 (s, 1H), 6.00 (brs, 1H), 7.25 (d, J = 7.0 Hz, 2H), 7.29-7.33 (m, 1H), 7.36-7.40 (m, 2H); ^{13}C NMR (125 MHz, $CDCl_3$) δ 18.3, 22.7, 57.2, 63.4, 126.2, 128.1, 128.7, 138.0, 174.9; IR (neat) *v* 3249, 2961, 2925, 1750, 1462, 1389, 1366, 1211, 1134, 1005, 978, 750, 700, 667; MS (ESI) (*m*/*z*): $[M+H]^+$ calcd for $C_{11}H_{14}NO$ 176.1, found 176.1.

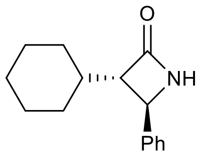

6e

Compound **6e**, yellow oil, yield: 59%. ^{1}H NMR (500 MHz, $CDCl_3$) δ 1.07-1.34 (m, 5H), 1.65-1.87 (m, 5H), 2.04 (m, 1H), 2.87 (dd, J = 8.1, 2.0 Hz, 1H), 4.48 (d, J = 2.3 Hz, 1H), 6.16 (brs, 1H), 7.28-7.32 (m, 1H), 7.34-7.39 (m, 4H); ^{13}C NMR (125 MHz, $CDCl_3$) δ 26.0, 26.1, 26.4, 30.8, 31.1, 38.1, 55.4, 67.7, 125.8, 128.1, 129.0, 140.6, 170.9; IR (neat) ν 3253, 2924, 2852, 1750, 1449, 1362, 1280, 1173, 990, 911, 732, 698; MS (ESI) (*m*/*z*): $[M+H]^+$ calcd for $C_{15}H_{20}NO$ 230.2, found 230.1. The relative configuration was determined by NOESY analysis of this compound.

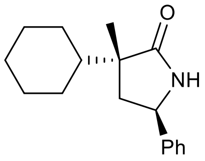

7

Compound **7**, yellow oil, yield: 56%. ^{1}H NMR (500 MHz, $CDCl_3$) δ 0.99-1.08 (m, 1H), 1.12-1.34 (m, 7H), 1.56-1.63 (m, 1H), 1.69-1.74 (m, 1H), 1.79-1.87 (m, 4H), 1.90-1.95 (m, 1H), 2.69 (dd, J = 13.4, 7.2 Hz, 1H), 5.28 (dd, J = 9.0, 7.4 Hz, 1H), 7.28-7.43 (m, 6H); ^{13}C NMR (125 MHz, $CDCl_3$) δ 24.3, 26.6, 26.7, 26.8, 27.6, 28.8, 43.0, 45.2, 48.2, 79.6, 125.5, 128.1, 141.5, 178.9; IR (neat) ν 3285, 2926, 2852, 1684, 1450, 1339, 1255, 1058, 931, 854, 758, 698; MS (ESI) (*m*/*z*): $[M+H]^+$ calcd for $C_{17}H_{24}NO$ 258.2, found 258.2. The relative configuration was determined by NOESY analysis of this compound.

Supplementary References

1. a) R. Shang, L. Ilies, A. Matsumoto, E. Nakamura, *J. Am. Chem. Soc.* **2013**, *135*, 6030; b) X.-S. Wu, Y. Zhao, H.-B. Ge, *J. Am. Chem. Soc.* **2014**, *136*, 1789.
2. X.-S. Wu, Y. Zhao, G.-W. Zhang, H.-B. Ge, *Angew. Chem. Int. Ed.* **2014**, *53*, 3706.
3. X.-S. Wu, Y. Zhao, H.-B. Ge, *Chem. Eur. J.* **2014**, *20*, 9530.
4. L. D. Tran, O. Daugulis, *Angew. Chem. Int. Ed.* **2012**, *51*, 5188.
5. F. Hoffmann-Emery, H. Hilpert, M. Scalone, P. Waldmeier, *J. Org. Chem.*, **2006**, *71*, 2000.
6. Y. Aihara, N. Chatani, *J. Am. Chem. Soc.* **2014**, *136*, 898.
7. G. Athanasellis, A. Detsi, K. Prousis, O. Igglessi-Markopoulou, J. Markopoulos, *Synthesis* **2003**, 2015.
